# Non-Aqueous Binary and Ternary *n*HF·Base Fluoride Reagents: Characterization of Structure, Properties, and Reactivity

**DOI:** 10.1021/jacs.5c05472

**Published:** 2025-05-26

**Authors:** Stephen G. Sweeting, Alastair J. J. Lennox

**Affiliations:** School of Chemistry, 1980 University of Bristol, Cantock’s Close, Bristol, Avon BS8 1TS, U.K.

## Abstract

Binary and ternary *n*HF·base mixtures are an important class of nucleophilic fluorinating reagents used in myriad fluorination reactions. These reagents are soluble in organic media, and by varying *n*, the reactivity of fluoride can be controlled and tuned. Of particularly frequent utility are the ternary mixtures of *n*HF·amine, in which the binary 9HF·py and 3HF·NEt_3_ mixtures are combined, the ratio (*n*) of which has a strong influence on the reaction yields and selectivity. The structure, properties, and reactivity of these non-aqueous ionic liquid mixtures vary considerably with *n*. Herein, we disclose a combined experimental and theoretical study aimed at characterizing binary and ternary *n*HF·base mixtures. We have measured the concentration of components, their Hammett acidity *H*_0_, nucleophilicity, and basicity, while using theory to calculate the lowest energy size and structure of the clusters formed at different ratios of HF to base and analyzed the noncovalent interactions present. The quantification of properties and enhanced understanding presented should facilitate the further development and use of this important family of fluorination reagents.

## Graphical abstract

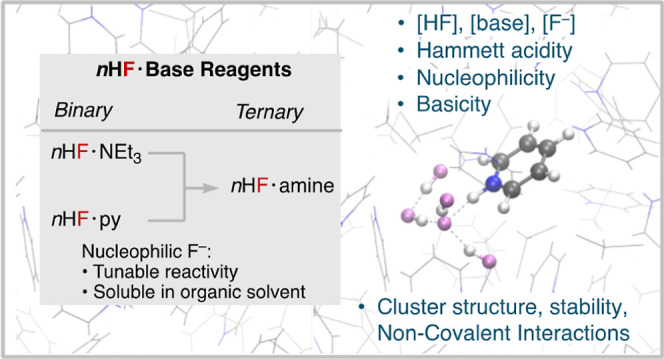

## Introduction

The ever-increasing ability of synthetic chemists to selectively incorporate fluorine into organic molecules has been an essential factor in the growth of many fields and applications, ranging from crystal engineering, battery technology, agriculture, and medicinal chemistry.^1–12^ For example, incorporation of fluorine into pharmacophores can enhance potency or help to tune pharmacokinetics,^8^ rationalizing why 34% of commercial pharmaceuticals contain at least one fluorine atom.^13^ The high charge density of fluorine and very strong bonds with carbon provides the basis for the plethora of these benefits.^14^

The development of different electrophilic^15–17^ and nucleophilic^18–22^ fluorination reagents for diverse reactivity has been the focus of intensive research over the past several decades. Great advances have been made to improve selectivity, reactivity, cost, and sustainability, with ever more novel or improved transformations being discovered.^23–28^

Within the realm of nucleophilic fluorination, the reactivity of the fluoride anion varies very significantly depending on the environment, with small changes being able to drastically affect the outcome of reactions. A large range of reactivity, nucleophilicity, and basicity can be accessed with different fluoride reagents; more ‘naked’ fluoride ions are highly reactive and are more intrinsically basic and nucleophilic, compared to a highly hydrogen-bonded fluoride ion.^29^ While the purity, vendor, conditions of storage, and handling are all important, the identity of the countercation is the most influential factor in determining the comparative solubility and reactivity profile between these reagents, Figure 1A. Controlling the reactivity of fluoride salts has been a long-standing challenge in the field,^30^ and specifically, quantifying the balance between basicity and nucleophilicity has been the subject of much focus.^21,23,31–34^

**Figure 1:**
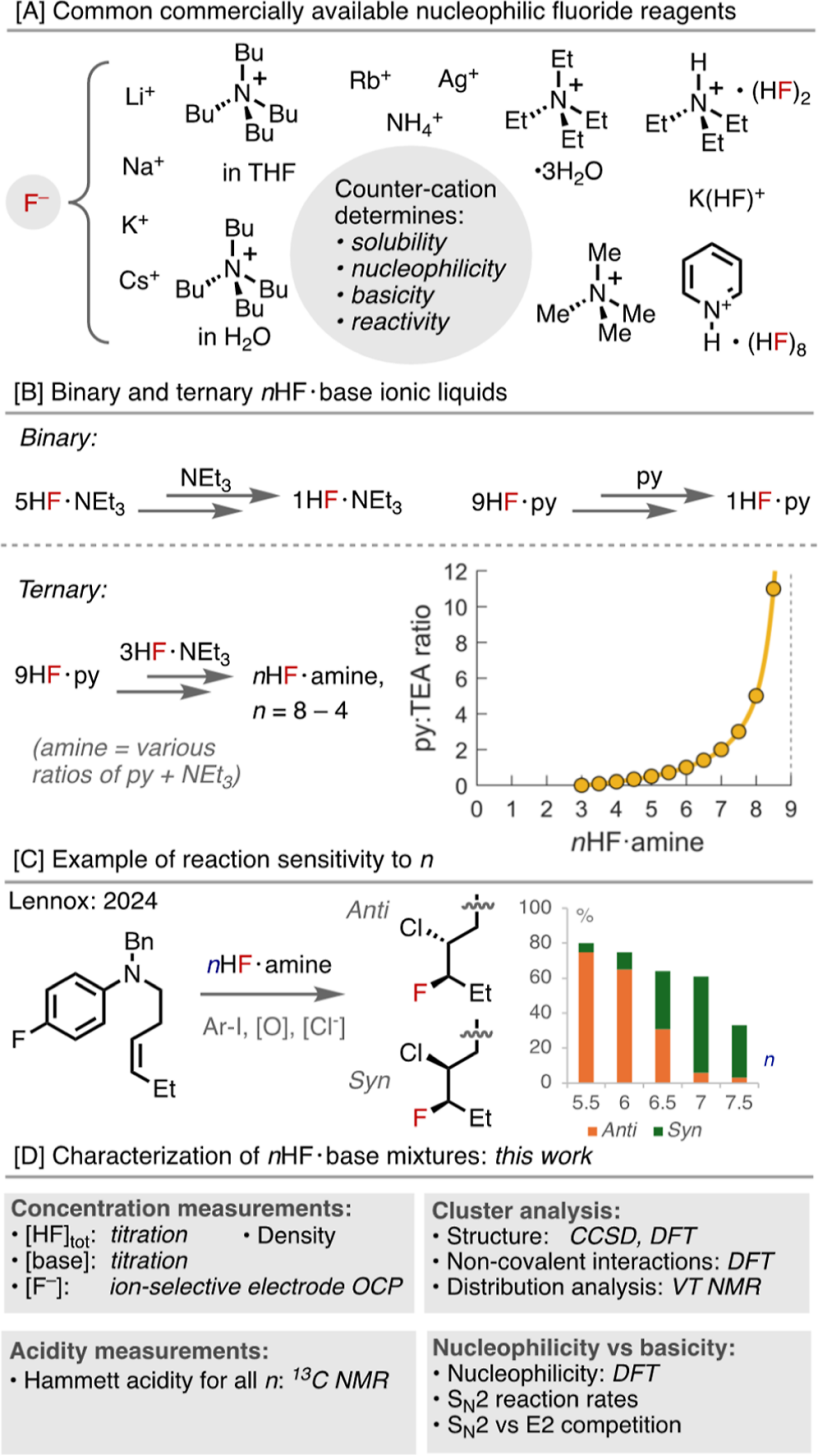
[A] Nucleophilic fluorination reagents couple a fluoride ion with a countercation; [B] binary and ternary *n*HF·base mixtures, the plot shows how the ratio of py:TEA (“amine”) varies with *n* in *n*HF·amine; [C] selected example of the sensitivity of diastereoselectivity to *n*; and [D] a summary of this work.

*n*HF·base reagents are a family of nucleophilic fluorinating reagents that exhibit a broad spectrum of utility,^35–41^ as they offer several unique characteristics compared to other reagents. 9HF·py (py = pyridine) or “Olah’s reagent”,^42^ which is 70% w/w HF with 30% w/w pyridine, and 3HF·TEA (TEA = triethylamine) or “Franz’s reagent”, which is 37% w/w HF with 63% w/w triethylamine,^43^ are both widely commercially available and have thus become the most prominent reagents used from this family. These reagents are ionic liquids at room temperature, readily soluble in organic solvents, and have relatively low hygroscopicity, low cost, and a lower, controllable nucleophilicity of fluoride. This collection of properties is distinctive and is not present in metal or ammonium fluorides. However, their acidic nature renders them more hazardous, and strict safety protocols are required for their handling, see Supporting Information for details. This property limits their use to reactions in which alternative fluoride sources do not work or perform inferiorly. Nevertheless, their set of unique properties still necessitate their use broadly in synthesis.^44,45^

In contrast to other nucleophilic fluoride sources, the reactivity of *n*HF·base reagents can be easily tuned. This can be performed by varying *n*, i.e., the ratio between HF and the base, Figure 1B, by simply diluting the same base into the commercially available reagents to generate binary *n*HF·base mixtures.^45–48^ Alternatively, as recently introduced by Gilmour,^49^ 9HF·py is diluted with 3HF·TEA to generate ternary “*n*HF·amine” mixtures, where amine is a combination of py and TEA. In these mixtures, the ratio of pyridine to NEt_3_ changes with *n*, Figure 1B, with richer ratios of pyridine at higher values of *n*. This strategy has emerged as the most popular way to adjust the reactivity of these reagents.^49–67^

The reactivity of fluoride with different *n* values changes the characteristics of the medium and the reactivity of fluoride to a very large extent. Many fluorination reactions, particularly those mediated by hypervalent iodine, show a high sensitivity to both *n* and the base, with yields, stereo-, chemo-, and regio-selectivity all being strongly affected (see Figure S1 for examples).^49–67^ A recent example published from our lab demonstrated the diastereoselectivity of an alkene chlorofluorination reaction could be entirely switched from *anti* to *syn* upon simply moving from 5.6HF·amine to 7.0HF·amine,^63^ Figure 1C. While extensive mechanistic studies were able to elucidate a specific rationale for this diastereoselectivity switch, the fundamental properties of ternary *n*HF·amine and binary *n*HF·py or *n*HF·TEA mixtures have not been studied. For example, it has not been characterized how the acidity of the medium changes or how the concentration and nucleophilicity of fluoride vary with *n*. Consequently, it remains challenging to predict optimal conditions or rationalize optimization data.

Herein, we present the results of a study aimed at characterizing the properties and structures of the binary *n*HF·py (*n* = 1–9), *n*HF·TEA (*n* = 1–5), and ternary *n*HF·amine (*n* = 4–8) mixtures that can be made from the commercially available reagents, Figure 1D. For each *n*, we have measured the concentrations of HF, fluoride, and base. We have conducted computational and VT NMR studies to characterize the size, structure, noncovalent interactions, and nucleophilicity of the lowest energy clusters in these nonaqueous ionic liquids, modeling the solvent with a bespoke implicit model. The Hammett acidity function *H*_0_, the nucleophilicity of fluoride, and the balance with basicity have been measured for each mixture. This study quantifies and compares the parameters and reactivity of this important family of *n*HF·base fluoride reagents, and it contrasts previous studies that have focused on a single parameter or reagent.^68–75^

## Results and Discussion

### Concentration Measurements

The total concentration of HF ([HF]_tot_) for each of the mixtures was measured via titration with KOH_(aq)_, and the KF produced and base (either TEA or py) were quantified (^19^F and ^1^H NMR against an internal standard),^68^ Figure 2A–C. The concentration and weight % of HF increased with *n* for all mixtures, Figure 2A,B. The concentration of HF was found to be greatest for *n*HF·py, followed by *n*HF·amine and *n*HF·TEA for each *n*, which is consistent with the molar concentrations of the two bases (py = 12.4 M vs TEA = 7.17 M). As the concentration of HF increases with *n*, the concentration of the base decreases, Figure 2C.

As the base in these mixtures is protonated, there is a corresponding fluoride counteranion present. The fluoride anion is stabilized by hydrogen-bonding HF molecules that form an array of clusters, the average size, structure and distribution of which depend on the precise composition of the mixture, vida infra. To measure the concentration of these fluoride anions in each of the mixtures, we used an electrochemical technique.^76^ A fluoride ion-selective electrode (Ag|AgF) was prepared, and open-circuit potential (OCP) measurements were calibrated to known concentrations of tetramethylammonium fluoride. The OCP of each *n*HF·base mixture was measured in a dilute solution to avoid solvation effects that might perturb the measurement, and the concentration in the neat solution was back calculated (see Supporting Information, Tables S5–S7). As *n* increases, the concentration of fluoride decreased, Figure 2D. For a given *n*, the concentration of fluoride anions is greatest in *n*HF·py, then *n*HF·amine, and then *n*HF·TEA. The concentration of fluoride in these *n*HF·base mixtures is significantly higher than that previously measured in anhydrous HF,^77,78^ which demonstrates the considerable influence of a base.

The concentrations of the base and fluoride correlate linearly, Figure 2E, which is expected considering the p*K*_a_ of both bases. Only a small deviation from equimolarity was observed; the concentration of fluoride is ca. 1.02 times higher than the base, which should be due to the autoionization of HF, vide infra.

Density measurements were conducted for each *n* in each type of mixture, Figure 2F (see Supporting Information, Tables S8–S10). For a given *n*, the density decreased from *n*HF·py to *n*HF·amine to *n*HF·TEA, which is a trend that aligns with, and can rationalize, how [HF]_tot_ varies for a given *n*. For *n*HF·py, *n* = 1–4, the density increases, but from *n* = 5–9, it then decreases. This interesting shape reflects the changing structure and distribution of the protonated base and anionic fluoride clusters, vide infra.

### Computational Studies on *n*HF·Base Clusters

To gain a deeper understanding of the lowest energy structures of the ionic clusters present in each of the binary *n*HF·py and *n*HF·TEA mixtures, we performed a range of computational studies. While there will be a distribution of clusters present at each *n*, a theoretical structural analysis of the individual lowest energy clusters provides insights into their observed reactivity.

Conformational sampling was performed using the Conformer-Rotamer Ensemble Sampling Tool (CREST),^79^ which allows different conformations to be examined at the GFN2-xTB level of theory.^80^ Although several are accessible, the lowest energy conformers found in each system were then used as initial inputs for geometry optimizations by using Density Functional Theory (DFT). All DFT optimizations used the Ahlrichs valence triple-ζ basis set, with two sets of polarization functions and a set of diffuse functions (def2-TZVPPD) as implemented in ORCA 6.0,^81,82^ using tight convergence criteria throughout. To determine a suitable DFT functional, benchmarking studies were performed against the Domain-based Local Pair Natural Orbital Coupled Cluster with Single and Double excitation method (DLPNO–CCSD)^83^ calculations as implemented in ORCA 6.0 (see Supporting Information for full details, Figures S6–S17).^81^ By comparing functionals known for modeling hydrogen bonding, it was established that the range-separated hybrid functional LC-PBE performed the best. Hence, all subsequent calculations adopted the LC-PBE/def2-TZVPPD level of theory.

**Figure 2:**
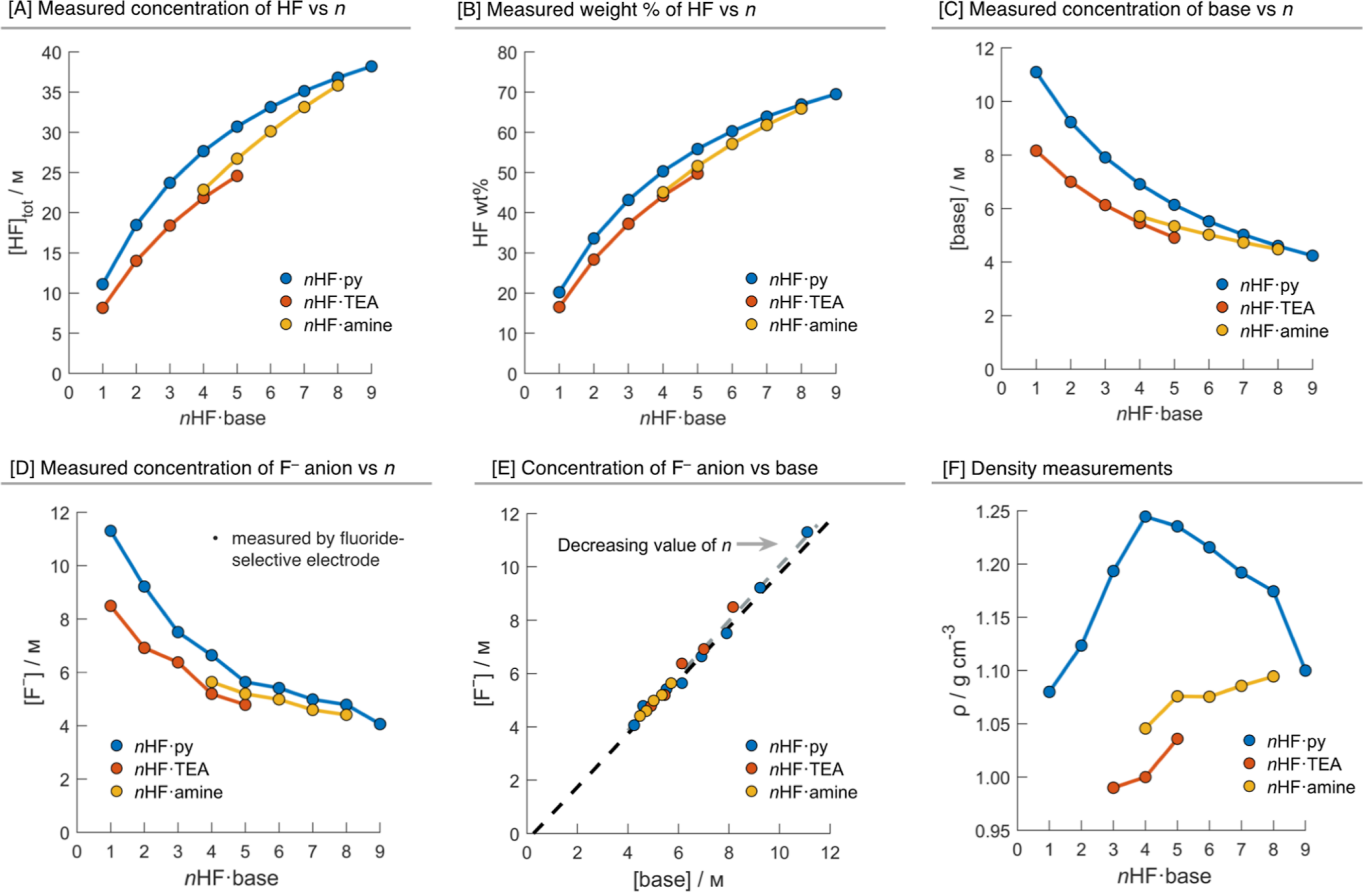
(A) The concentration of [HF] vs *n* in each mixture. (B) The weight percentage of HF vs *n* in each mixture. (C) The concentration of the base measured in each mixture. (D) Concentration of the fluoride anion measured from the open-circuit potential (OCP) using a fluoride ion-selective electrode plotted against *n*. (E) Concentration of the fluoride anion against the concentration of the base. (F) The density of *n*HF·base ionic liquids. *n*HF·TEA, where *n* = 1 and 2 are solids and therefore could not be measured.

We developed an implicit solvent model to account for the *n*HF·base mixtures, as other solvent models are not adequate to describe these environments. By calculating the static dielectric constant and refractive index for each *n*HF·base mixture, we built conductor-like polarizable continuum models (CPCMs)^84,85^ for each mixture (*n* and base) (see Supporting Information for full details, Figure S18). We optimized the clusters in the least polar and most polar implicit solvent systems, i.e., low and high dielectrics, respectively, as the spread of effects due to the change in polarity should be captured within these two limiting scenarios.

The optimized clusters from *n* = 1–9 (py) and 1–5 (TEA) demonstrate how additional HF molecules interact with the base·HF salt, Figure 3A and B. The first two additional HF molecules (*n* = 2–3) solvate fluoride to form a primary solvation shell. The next 2 HF molecules (*n* = 4 and 5) interact with the solvating HFs to form a secondary solvation shell. With py as a base, from *n* = 6, the fluoride is solvated by a third HF molecule, after which (*n* = 7–9) secondary and tertiary solvation shells are formed. Interestingly, no structures were optimized in which the fluoride ion was coordinated to 4 HF molecules in its primary solvation shell.

To investigate why four strong hydrogen bonds are not formed around the fluoride anion, we considered the anionic clusters in isolation to assess their stability. Geometry optimizations were performed using Couple Cluster Singles and Doubles (CCSD) level of theory with the augmented correlation-consistent polarized valence triple-ζ basis set (aug-cc-pVTZ)^86^ as implemented in ORCA 6.0 to calculate the stabilization energy (*E*_stab_) and binding energy. The stabilization increased when more HF molecules are introduced to the linear bifluoride anion, as the negative charge on fluoride is distributed over a larger system; see Supporting Information, Figure S19. However, with four coordinating HFs to fluoride in a tetrahedral cluster, the stabilization reduced due to the increase in steric strain in the primary solvation shell. Hence, formation of the tetrahedral cluster is unfavorable at 298 K. A tetrahedral anion [F­(HF)_4_]^−^ has, however, been detected at a low temperature (130 K, NMR) by both Olah and Limback,^37,42,87^ at which stabilization from the stronger hydrogen bonds should overcome the steric repulsion.

### Noncovalent Interaction Analysis

The noncovalent interactions (NCIs) present in these clusters were analyzed to further characterize their structure. In the first instance, reduced density gradient (RDG) calculations using MultiWFN 3.8 ^88^ were performed to determine the strength and nature of the NCIs present, see Figure 3C for representative plots (see Supporting Information for full details including RDG-NCI maps, Figures S19–S32). The ionic hydrogen bonds in the primary solvation shell of the fluoride anion and the neutral hydrogen bonds in the secondary and tertiary solvation shells are all confirmed to be strong NCIs in this analysis for all *n*HF·py and *n*HF·TEA clusters. Weak attractive interactions between the π-hole of the pyridinium cation and HF molecules are present in the 8HF·py and 9HF·py clusters. Weak attractive interactions are also found between HF and the methylene carbons on the triethylammonium cation in all of the *n*HF·TEA clusters.

The strength of the NCI between the protonated base and the anionic cluster (blue arrow, Figure 3A and B) was quantified, Figure 3D, using the Quantum Theory of Atoms In Molecules (QTAIM), which is a more quantitative technique to assess NCIs. The interaction energy decreases with increasing *n* in both the *n*HF·py and *n*HF·TEA clusters, indicating increasingly weaker interactions between these two entities. The lower charge density associated with the anionic clusters with more HFs results in a smaller interaction with the protonated base. From *n* = 1–5, this weakening is rapid, but from 6–9, it is very gradual. This change aligns with the NCI of interest (blue arrow) shifting from one that involves an anionic hydrogen bond to the fluoride anion to the one that involves a neutral hydrogen bond to a solvating HF. These analyses have revealed that the interaction between the protonated base and anionic fluoride cluster decreases with *n*. For example, when *n* > 5, the fluoride anion is separated from the protonated base and does not significantly interact with it. This reduction in interaction should serve to increase the volume and therefore can rationalize the decrease in density that is experimentally observed, as shown in Figure 2F.

The degree of ionic vs covalent character in these hydrogen bonds (blue arrows) was analyzed by assessing the ratio between the electron density (ρ_BCP_) and the total electronic density (*H*_BCP_), see Supporting Information for details, Tables S11–S24. As *n* increases in both the *n*HF·py and *n*HF·TEA clusters, the interaction was found to become more ionic in nature, although it does not become fully ionic as a small amount of covalent character remains.

QTAIM was then used to analyze how the strength of each hydrogen bond (interaction energy) changes as HFs are introduced into the complex with increasing *n*. The hydrogen bonds being discussed are assessed by consideration of which solvation shell they are contained in, Figure 3E. The addition of HF to a cluster either strengthens the hydrogen bond of interest, i.e., is cooperative, or weakens it, i.e., is anti-cooperative. Computational work by Deshmukh showed only cooperative effects when considering both isolated linear and cyclic hydrogen fluoride clusters that did not involve a base.^89^

The hydrogen bond in the primary solvation shell weakens when HFs are added to this primary solvation shell, i.e., the addition is anti-cooperative, Figure 3E-i. The reduced charge density of the fluoride anion and a reduction in the optimal orbital overlap result in this weakening. However, the ionic hydrogen bonds of the primary solvation shell are strengthened when the secondary solvation and, to a lesser extent, the tertiary solvation shell are built up, i.e., the addition of HF is cooperative. In this instance, the more disperse electron density increases the strength of the ionic hydrogen bond.

The hydrogen bonds in the secondary solvation shell also weaken when HFs are added to the secondary solvation shell, i.e., the addition is anti-cooperative, Figure 3E-ii, due to the greater dispersion of electron density. These hydrogen bonds, Figure 3E-ii, as well as those in the tertiary shell, Figure 3E-iii, however, become strengthened with the development of the tertiary solvation shell in the 8HF·py and 9HF·py clusters. This effect is due to interactions between the π-hole and tertiary shell HF molecules (see green ellipsoids in Figure 3D), which provides more electron density to form stronger hydrogen bonds in the secondary and tertiary solvation shells. This strengthening effect should be unique to 8HF·py and 9HF·py clusters, as nonaromatic bases will not feature a π-hole in their structure.

**Figure 3:**
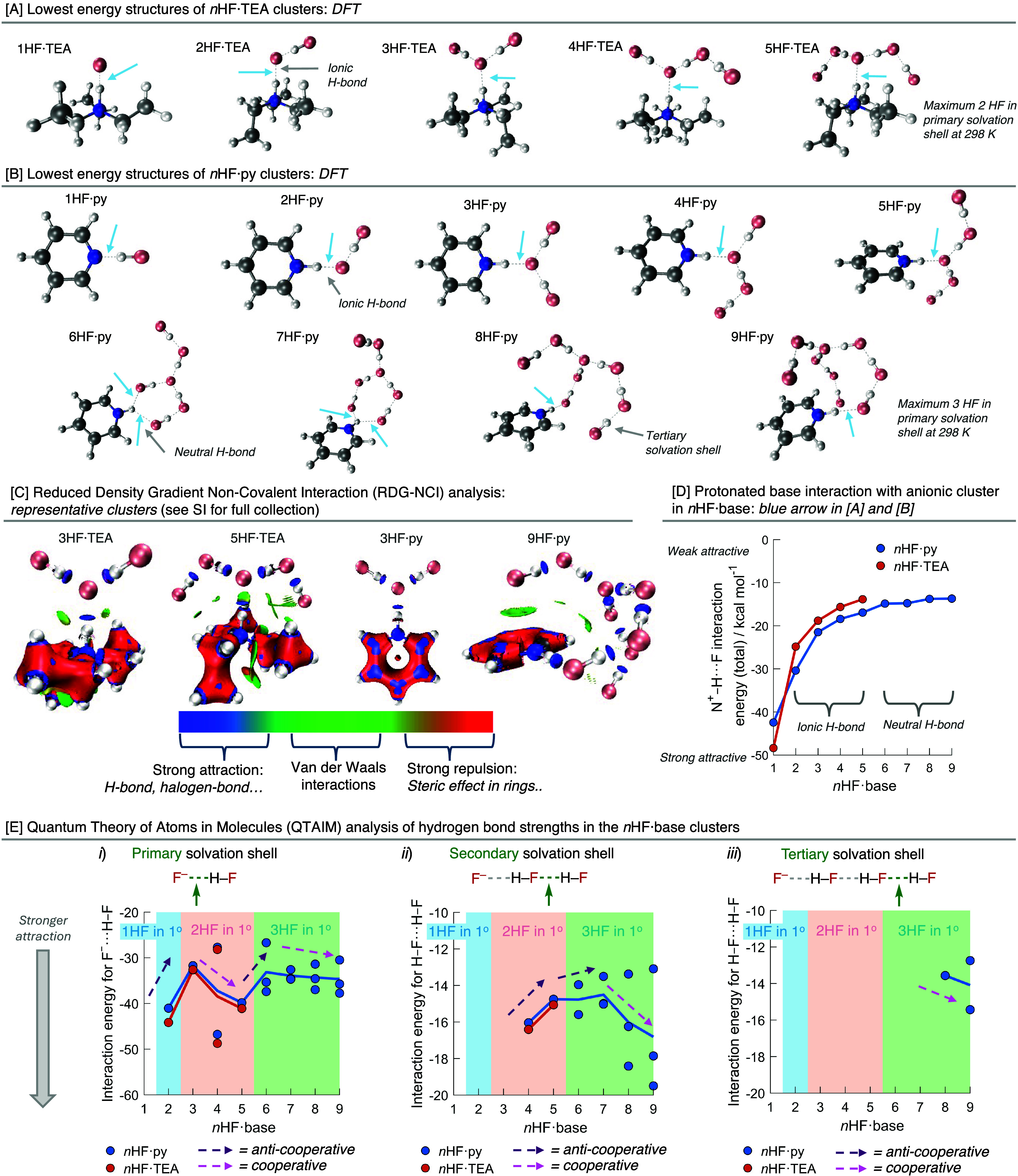
Calculated structures of (A) *n*HF·TEA and (B) *n*HF·py clusters using DFT/LC-PBE/def2-TZVPPD + (custom). Blue arrows indicate the noncovalent interaction assessed in part (D); (C) Reduced Density Gradient Non-Covalent Interaction (RDG-NCI) analysis; (D) interaction energy plot for the noncovalent interaction between the protonated base and the anionic cluster, see the blue arrow in part [A] and [B]; and (E) Quantum Theory of Atoms in Molecules (QTAIM) analysis of hydrogen-bond strengths in the *n*HF·base clusters using results obtained from DFT/LC-PBE/def2-TZVPPD + (custom).

### Average Cluster Size Analysis at Each *n* by NMR

Each anionic cluster [F­(HF)_*x*_]^−^ should have two associated peaks in the ^19^F NMR spectrum: one for the fluoride anion and another for the hydrogen-bonded HF molecules. To gain some further insights into the fluoride/HF clusters ([F­(HF)_*x*_]^−^) formed in the *n*HF·base mixtures, both room-temperature and variable-temperature (VT) NMR studies were performed. ^19^F and ^1^H NMR spectra were recorded, first as neat liquids at 298 K and then at lower temperatures in DCM. Similar analyses have been performed on related (neat) HF reagents.^75,87,90^

Only a single peak for the HF was observed at all temperatures, as the peak for the fluoride anion could not be detected due to the low intensity relative to that of HF. For every *n*, there will be a distribution of cluster sizes that depend on a variety of factors, including *n* and the solvent polarity. For example, a smaller distribution is expected at low and high values of *n*.^91^ The chemical shift of the HF peak represents an average shift for all of the HF molecules in the distribution of clusters present in each mixture.

For each neat *n*HF·base mixture, the ^19^F NMR signal shifted upfield with *n*, Figure 4A. However, across different bases for the same *n*, the observed peak is relatively similar, which suggests a similar distribution of cluster sizes are present. The upfield shift with increasing *n* aligns with an increase in the size of the clusters, i.e., x increases in [F­(HF)_*x*_]^−^. With more HFs interacting with the fluoride anion, the ionic hydrogen bonds lengthen as the orbital overlap between fluoride and the σ* orbital of the HF decreases, leading to greater shielding of the fluorine in HF and an upfield shift in the NMR.

**Figure 4:**
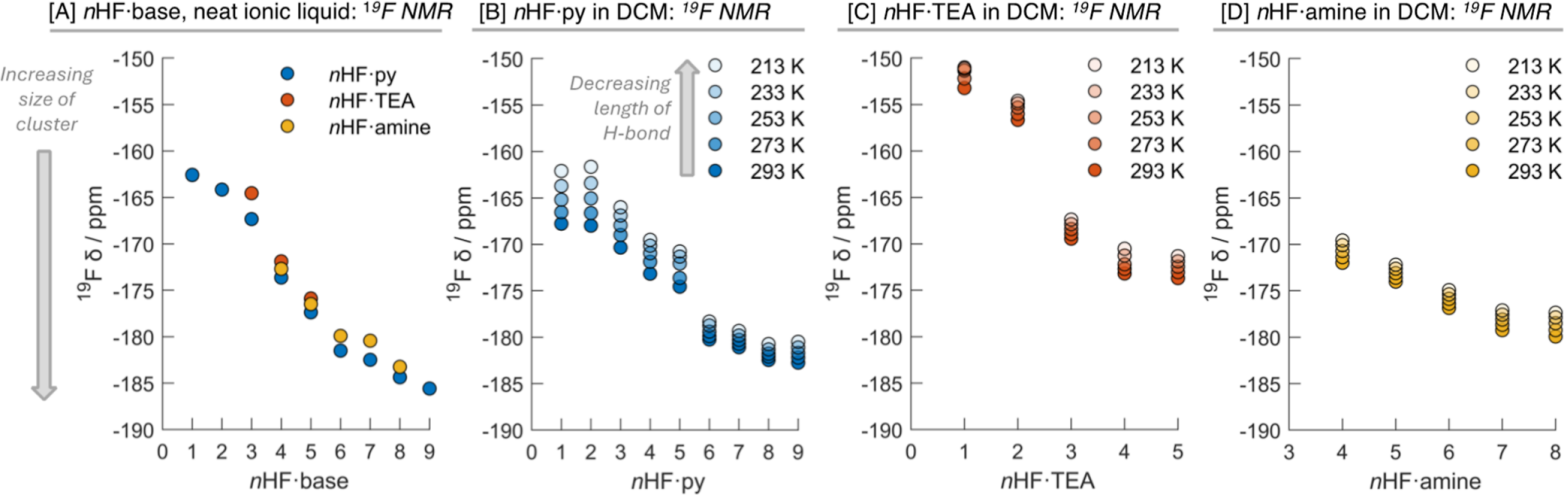
^19^F NMR shift of the fluoride anion solvating HF molecules are plotted against *n*. (A) Recorded neat at 298 K and (B–D) recorded in a solution of DCM (3:1 *n*HF·base:DCM) from 213 to 298 K.

The VT measurements in DCM showed the ^19^F NMR signals for each mixture shifting downfield as the temperature decreases, Figure 4B–D.^75,92^ The length of the hydrogen bonds will decrease, which reduces the shielding of the fluorine in the coordinating HF. For each *n*, the chemical shift is similar for each base mixture, indicating a similar distribution of clusters. For solutions of *n*HF·py, the variation in the ^19^F NMR shift with temperature is observed to be much larger for lower *n* compared to higher *n*, for example, there is a spread of ca. 7 ppm for *n* = 1 and 2 compared to a spread of ca. 2 ppm for *n* = 7–9. This difference reflects a larger change in the length of hydrogen bonds at lower *n* compared to higher *n*, i.e., the larger clusters are less sensitive to temperature changes than the smaller clusters. Another notable observation from these measurements is the upfield change in the NMR signal for *n*HF·py and *n*HF·TEA after *n* = 5 and 2, respectively. This represents a greater change in the distribution of clusters compared with other changes in *n*. It is of note that the computational calculations predict an additional hydrogen bond directly to the fluoride anion after *n* = 5 and *n* = 2 for *n*HF·py and *n*HF·TEA, respectively, Figure 3A and B.

### Acidity Determination

To establish the acidity of each binary and ternary *n*HF·base mixture, we measured their Hammett acidity functions (*H*_0_). The pH scale is not suitable for systems where the acidic species are present at high concentrations in solutions or when considering non-aqueous solvents. The Hammett acidity (*H*_0_) function has the form of eq 1, where B is a basic indicator and p*K*_BH^+^_ is −log­(*K*) for the dissociation of the protonated base.^93^

1 $$H_{0}={pK}_{{BH}^{+}}+\log_{10}\left(\frac{\left[B\right]}{\left[{BH}^{+}\right]}\right)$$

Thibaudeau and co-workers have recently used a series of novel *N*-trifluoromethylaniline bases to measure *H*_0_ for *n*HF·py, *n* > 6.2 with ^19^F NMR.^69^ As these aniline bases are not suitable for measuring solutions of lower acidity, this method could not be used to measure the acidity of the broader range of *n*HF·base mixtures being studied herein, namely, *n*HF·py (*n* = 1–9), *n*HF·TEA (*n* = 3–5), and *n*HF·amine (*n* = 4–8). Several other reported methods, which are based on a variety of techniques including UV–vis spectroscopy,^94,95^ IR spectroscopy,^74,96^ or NMR with alternative indicators,^97,98^ were considered or tested; however, none were found to be compatible or suitable for HF-containing mixtures. Hence, a new approach was sought.

We identified a series of Hammett bases with known p*K*_BH^+^_ that would indicate in the region of relevance, *H*_0_ = ca. −6 to 2, Figure 5A. Rather than using absorption spectroscopy to detect their ionization,^94,95^ we turned to ^13^C NMR spectroscopy. A single peak is produced per carbon for the equilibrium, the shift of which is proportional to the degree of ionization. Hence, the Hammett acidity can be calculated using eq 2:

2 $$H_{0}={pK}_{{BH}^{+}}-\log_{10}\left(\frac{\delta_{B}-\delta_{obs}}{\delta_{obs}-\delta_{{BH}^{+}}}\right)$$

where δ_B_ and δ_BH^+^_ are the ^13^C NMR chemical shifts of the Hammett base and conjugate acid, respectively, and δ_obs_ is the observed chemical shift of the base in the solution being measured, which is the weighted average of the δ_B_ and δ_BH^+^_ indicator shifts, eq 3. Reference δ_B_ and δ_BH^+^_ values for each indicator were recorded in both DCM and anhydrous hydrogen fluoride, respectively.

3 $$\delta_{obs}=\frac{\delta_{{BH}^{+}}\left[{BH}^{+}\right]+\delta_{B}\left[B\right]}{\left[{BH}^{+}\right]+\left[B\right]}$$

We measured *H*_0_ for *n*HF·py (*n* = 1–9), *n*HF·TEA (*n* = 3–5), and *n*HF·amine (*n* = 4–8) ionic liquids, Figure 5B. The acidity was found to increase as the concentration of HF increases within each series, e.g., for *n*HF·py *n* = 1–9, the *H*_0_ increases from ca. 0.9 to −5.1. When plotted against the wt % of HF, Figure 5C, the acidity observed increases gradually up to ca. 50% HF, at which point there is a point of inflection, and the acidity increases more substantially with increasing HF concentration. Although the acidities are lower, this monotonic relationship is similar to that observed in aqueous hydrofluoric acid solutions.^70^ The increase in acidity below 50 wt % should be due to an increase in the stability of the anionic conjugate base, eq 4, with more HF solvating the fluoride and larger anionic clusters formed.^99–101^ In turn, this facilitates the dissociation of the protonated base salt, which increases the acidity.

4 $$Base\cdot HF+xHF⇌{\left[base\cdot H\right]}^{+}+\left[F{\left(HF\right)}_{x}\right]$$

**Figure 5:**
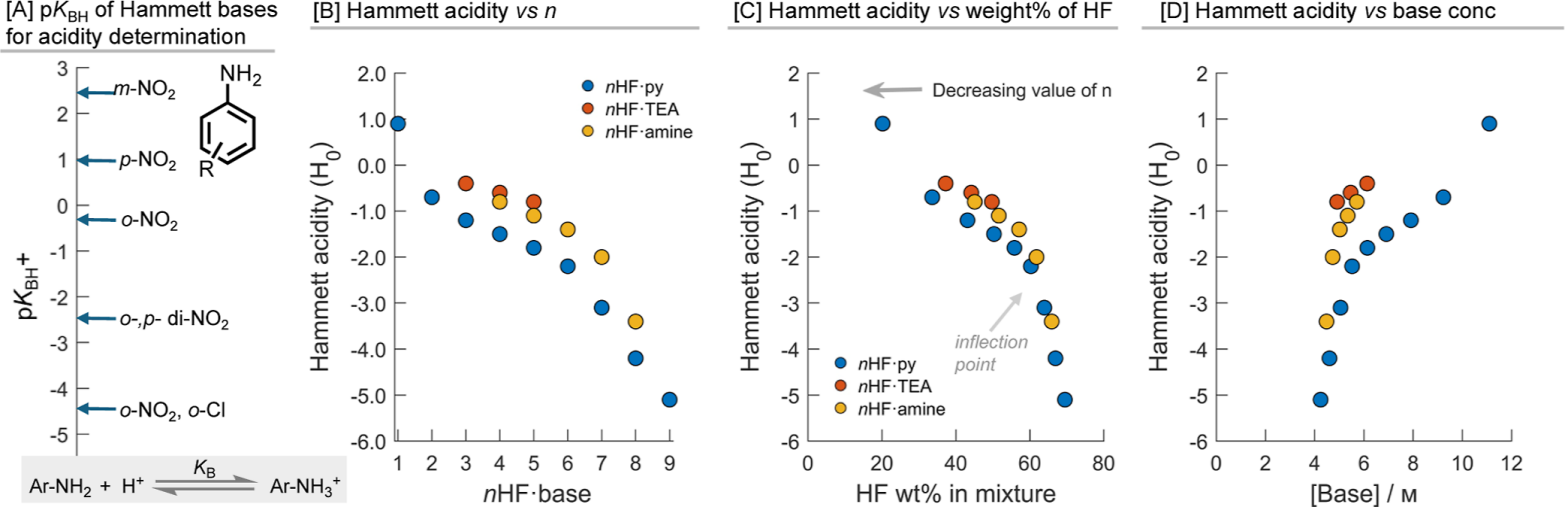
(A) Hammett acidity functions (*H*_0_) of each *n* in each *n*HF·base mixture measured using the Hammett bases with ^13^C NMR. *H*_0_ plotted against (B) *n* and (C) HF wt % at 0 °C. Note, 1HF·TEA and 2HF·TEA are hygroscopic solids and therefore could not be measured.

When the wt % of HF rises over 50%, Figure 5C, or the base concentration decreases below 6 M, Figure 5D, the acidity of the ionic liquids increases more rapidly, as can be observed for both *n*HF·py and *n*HF·amine. We propose that both homoassociation and autoionization of HF in the larger anionic clusters contribute to this increase in acidity after 50 wt % [HF], eq 5, which would be consistent with that reported for aqueous solutions of HF.^102–104^ The bifluoride [F_2_H]^−^ and trifluoride [F_3_H_2_]^−^ anions are modeled to increase in concentration as the HF concentration increases, which has also been observed experimentally.^102,103^

5 $${\left[F{\left(HF\right)}_{x}\right]}^{-}⇌{\left[F\left({\left(HF\right)}_{x-2}\right)\right]}^{-}+{\left[F\left(HF\right)\right]}^{-}+H^{+}$$

For each given *n*, *n*HF·py is more acidic than *n*HF·amine, which is more acidic than *n*HF·TEA. Additionally, when comparing mixtures with different bases but equal concentrations of HF, the acidity is different. For example, the concentration of HF is comparable in 3HF·py and 5HF·TEA (ca. 24 mM) (see Supporting Information, Tables S85–S87); however, the *H*_0_ is −1.2 and −0.8, respectively. This difference is related to the relative acidity of the protonated base salt. We measured the p*K*_a_ of both pyridinium fluoride and triethylammonium fluoride in DCM, to be 5.21 and 10.74, respectively, and a 1:1 mixture of the two salts to represent an effective p*K*_a_ for an “amine” mixture was found to be 6.41.^105^ As expected, the stronger base reduces the acidity of the ionic liquids, and so the combination of bases in “amine” can be used to tune the acidity of the mixtures.

Our methodology has expanded the range of acidity that is possible to measure compared to the known methods,^69,74,93–98^ allowing for the acidity of the full range of *n*HF·TEA, *n*HF·amine, and *n*HF·py ionic liquids to be measured for the first time.

### Quantification of Fluoride Nucleophilicity

A combined computational and experimental approach was applied to quantify the nucleophilicity of the fluoride anion in each of the binary and ternary *n*HF·base mixtures. DFT calculations were conducted to probe the theoretical trends for the individual clusters, while the nucleophilicity of fluoride contained in the distributions of these clusters in each of the *n*HF·base mixtures was measured in a model S_N_2 reaction. Such studies have not previously been reported on HF reagents.

To directly quantify the nucleophilicity of the fluoride anion in each of the *n*HF·base mixtures, we measured rates of nucleophilic substitution in a model S_N_2 reaction.^106^ The criteria for such a reaction are that (a) there is no competing elimination to give an alkene and (b) there is no competing S_N_1, which would otherwise not accurately reflect the nucleophilicity of the fluoride anion. Several reactions were considered, but the simple substitution of 4-nitrobenzyl bromide **1** by fluoride to **2** was found to qualify both criteria, Figure 6A. To confirm that the reaction was entirely S_N_2, enantiomerically enriched deuterated 4-nitrobenzyl bromide ([^2^H]-**1**) was prepared and subjected to the reaction conditions. Complete inversion of the configuration was observed when using either enantiomer, and only a very small secondary kinetic isotope effect was observed, which is a feature associated with a stereospecific S_N_2 substitution reaction.

Substitution reactions were monitored by analyzing the decay of **1** by ^1^H NMR. Despite being under pseudo-first-order conditions with a large excess of fluoride, the reaction was found to reach equilibrium, as the liberated bromide ion is a stronger nucleophile under the protic conditions. Second-order rate constants were determined both by using the initial rate method, Figure 6B, and extracting the rate constants from an S_N_2 substitution equilibrium model in COPASI, see Supporting Information, Tables S89–S109. Both methods revealed a decline in the second-order rate constant and therefore the nucleophilicity of fluoride,^107^ with increasing *n* in all mixtures, from ca. 2 to 0.25 × 10^–3^ M^–1^s^–1^. For context, the corresponding rate with tetrabutylammonium fluoride (TBAF) was measured to be at least 5 × 10^–3^ M^–1^ s^–1^ (see Supporting Information entry S79 for details).

The nucleophilicity of fluoride in each of the clusters, Figure 3A and B, was calculated to rationalize why the decline in nucleophilicity was very steep from *n* = 1 to 4 but much lower from *n* = 5 to 9. There are several approaches of representing nucleophilicity with a theory,^108^ including the ionization potential (*I*) predicted using Koopman’s Theorem,^109^ the local ionization potentials (*I̅*(**r**)), the local electron affinity (*A*_E_), the local electron attachment energy (*E*_att_(**r**)), or molecular electrostatic potentials. We tested each approach^109–112^ (see Supporting Information for full details, Tables S26–S76) but found the average local ionization potential (*I̅*(**r**)) at the fluoride anion, combined with the local electron attachment energy (*E*_att_(**r**)), was the most appropriate for these clusters, eq 6.

6 $$N^{\prime }=\frac{2\left(\mathrm{I̅}\left(r\right)-E_{att}\left(r\right)\right)}{{\left(\mathrm{I̅}\left(r\right)+E_{att}\left(r\right)\right)}^{2}}$$

Through this approach (DFT, custom implicit solvent), as expected, the nucleophilicity of the fluoride anion dropped with an increasing size of the anionic cluster, from ca. 0.54 to 0.47 eV, Figure 6C. The larger and higher stability clusters are harder; i.e., they are more resistant to exchange electron density, and therefore the nucleophilicity is dampened. For context, the corresponding nucleophilicity for tetramethylammonium fluoride (TMAF) was calculated to be 0.67 eV.

These computational studies, Figure 6C, revealed that when there is less than 2HF in the primary shell, the development of the primary and secondary solvation shells has a very large impact on dampening the nucleophilicity of the fluoride anion. However, when 3 HFs are present in the primary solvation shell, i.e., *n* > 6, the development of the secondary and tertiary solvation shells has only a very minor dampening effect on the nucleophilicity. These findings mirror the plateauing observed in the experimental rate constants measured, Figure 6B, and hence, by extension, the nucleophilicity is consistent with the cluster analyses.

The nucleophilicity of *n*HF·TEA was found to be greater than that of *n*HF·amine, which was greater than that of *n*HF·py, at the same *n*. Our VT NMR studies of the neat *n*HF·base mixtures predict that the distribution of clusters at each *n* is broadly the same for each base. Hence, this difference in nucleophilicity is due to the basicity of the base and the resulting influence of the protonated salt; cf. when *n* = 1, triethylammonium fluoride is a more nucleophilic source of fluoride than pyridinium fluoride, Figure 6B. The stronger TEA base produces a more stable protonated ammonium salt that dampens the nucleophilicity of the fluoride anion less than the corresponding pyridinium salt, with amine in between.

**Figure 6:**
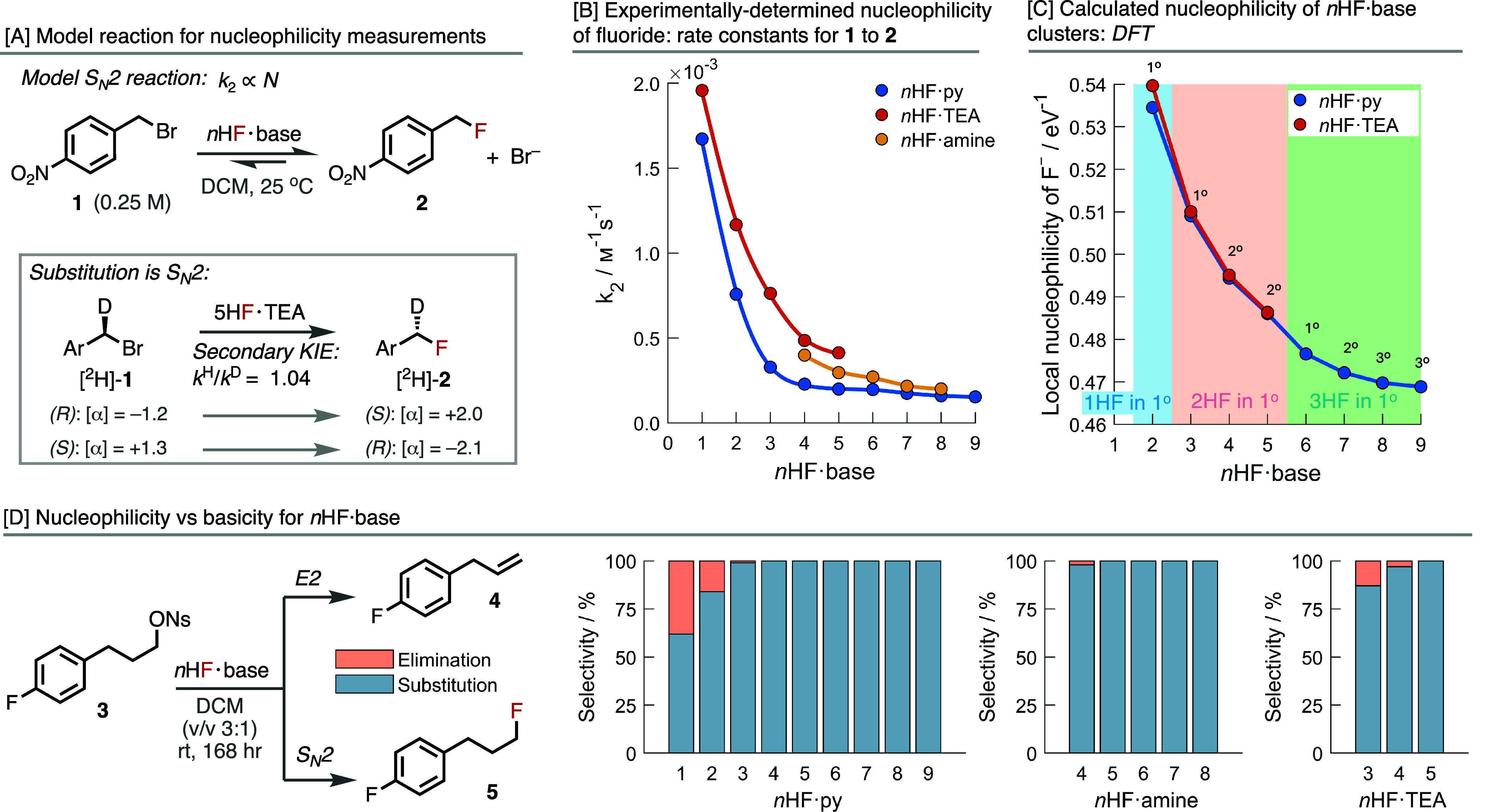
(A) Model reaction used for determination of fluoride nucleophilicity. Optical rotations given in deg mL g^–1^ dm^–1^ and measured at 20 °C using the sodium D line. KIE measured from independent rate measurements of [^2^H]-**1** and **1** with 5HF·TEA. (B) Experimentally determined nucleophilicity of fluoride in each *n*HF·base mixture in DCM (0.25 M) at 298 K. Second-order rate constants shown were extracted using the initial rate method. (C) The calculated nucleophilicity of *n*HF·base using eq 6 [DFT/LC-PBE/def2-TZVPPD + (custom)]. Labels next to data points indicate the solvation shell into which the last HF molecule has added into, 1° = primary, 2° = secondary, and 3° = tertiary. (D) Nucleophilicity vs basicity (S_N_2 vs E2) measured for all mixtures.

To investigate the balance between the basicity and nucleophilicity of the fluoride anion in each mixture, we tested the reactivity of each mixture on a substrate capable of both E2 and S_N_2. An analysis of selectivity between the two pathways confirms the fluoride is more nucleophilic than basic in all mixtures, Figure 6D. Only for low values of *n* (<4) is any basic behavior observed, which is still surprising considering the acidity of the media. The ability of fluoride to interact with acidic protons is directly suppressed through hydrogen bonding with HF in the larger clusters. The distortion energy for the S_N_2 reaction increases less than the distortion energy for the elimination reaction when *n* and the cluster size increases.^113–115^ Using our computed *n*HF·base clusters, Figure 3, we calculated that the intrinsic basicity decreases with increasing values of *n* and found that the trend obtained matched that observed experimentally (see Supporting Information for details, Tables S77–S80).

## Conclusion

In summary, we have conducted a combined experimental and theoretical investigation into the properties, structure, and reactivity of binary and ternary *n*HF·base ionic liquids. The key findings can be summarized by the following:

- The concentration of HF increases with *n*, while the concentration of anionic fluoride and base decreases with *n.* For each *n*, the concentration of HF, fluoride, and base decreases from *n*HF·py to *n*HF·amine to *n*HF·TEA, which is due to the different molar concentrations of the base.
- The density increases with *n* for each mixture, except from *n* = 5 to 9 for *n*HF·py, which we propose to be due to the formation of extended secondary and tertiary solvation shells in the clusters that serve to increase the volume.
- Computational studies on the individual calculated lowest energy clusters revealed that with increasing *n* and the size of the anionic cluster, HF solvates fluoride either in a primary, secondary, or tertiary shell, depending on *n.* A maximum of two HFs solvate fluoride in the primary solvation shell for *n*HF·TEA and 3 for *n*HF·py, at 298 K. The addition of each HF to the cluster has either cooperative or anticooperative effects on the strength of other hydrogen bonds in the cluster. The interaction between the protonated base and anionic cluster decreases in strength with increasing *n* and becomes more ionic than covalent in nature.
- The distribution of the clusters present at each *n* is broadly similar across the different base mixtures, when neat or diluted in DCM, according to NMR.
- The Hammett acidity (*H*_0_) increases with *n* and the wt % HF, due to stabilization of the fluoride and greater dissociation of the protonated base. An inflection point is observed around 50% HF, which we propose reflects initiation of HF autoionization. For each *n*, the acidity decreases from *n*HF·py to *n*HF·amine to *n*HF·TEA.
- The nucleophilicity of fluoride was measured in each *n*HF·base mixture, which confirmed a sharp decline until *n* = 4, after which only a minor further decline was observed with increasing *n.* This trend reflects the nucleophilicity calculated from the increasing size of the clusters and development of tertiary solvation shells around fluoride.
- The balance of nucleophilicity vs basicity for fluoride in the *n*HF·base mixtures was measured and found to be far more nucleophilic than basic for all mixtures, with only *n* = 1 and 2 for *n*HF·py, 4 for *n*HF·amine, and 3 for *n*HF·TEA displaying any basic behavior at all.

The addition of solvents will serve to dilute the *n*HF·base mixtures and reduce the HF concentration and the acidity. While our NMR analysis in DCM showed that the average cluster size does not significantly change upon dilution in DCM,^49–67^ we expect that other more polar solvents will disrupt the structure and distribution of clusters to a greater degree. The addition of less polar solvents should serve to increase the nucleophilicity and basicity of the fluoride anion.

With the properties and reactivity of the binary and ternary *n*HF·base ionic liquids quantified, data on reaction outcomes with their use can be better rationalized and their reactivity can be more easily predicted. Characterization of the lowest energy structure and size of the clusters with theory, and the noncovalent interactions present, creates a critical link between structure and reactivity that improves our understanding of this family of reagents. As an example, when *n* > 4 for *n*HF·py mixtures, the nucleophilicity and basicity of fluoride do not change very substantially, which is in direct contrast to the acidity of the medium that increases substantially when *n* > 4. These trends can be directly linked to the structure of the clusters that more readily form secondary and tertiary solvation shells when *n* > 4. The outer solvation shells have a relatively minor dampening effect on the nucleophilicity of the fluoride anion. However, the higher concentration of HF increases the acidity of the medium through HF autoionization and formation of the stable [F_2_H]^−^ and [F_3_H_2_]^−^ anions.^116^

The large difference in acidity but relative similarity of nucleophilicity and basicity for *n* > 4 should help to rationalize the outcome of fluorination reactions that are mediated by distinct hypervalent iodine reagents. For example, it can now be elucidated that the diastereoselectivity for alkene chlorofluorination, Figure 1C, requires a higher acidity medium to activate the hypervalent iodine reagent that is responsible for *syn*-chlorofluorination compared to the reagent responsible for *anti*-chlorofluorination, which is more reactive.^63^ Or more generally, variation in reaction optimization data for *n* > 4 can be assigned to differences in the acidity, rather than fluoride nucleophilicity. Hence, applications that require the use of *n*HF·base ionic liquids can now be developed with greater understanding and control of reactivity and provide the potential for prediction.

## Supplementary Material



## Data Availability

The data underlying this study are available in the published article and its Supporting Information.

## Notes

Experimental and computational procedures, characterization data for known and new compounds, and spectra for new compounds (PDF)
