# Supplementary material for: Non-Aqueous Binary and Ternary nHF·Base Fluoride Reagents: Characterization of Structure, Properties, and Reactivity
Source: J Am Chem Soc. 2025 May 26;147(22):19329–41. doi: 10.1021/jacs.5c05472 (PMC12147121; doi:10.1021/jacs.5c05472)
Supplement: Supplementary file 1 [file ja5c05472_si_001.pdf]

Non-Aqueous Binary and Ternary *n*HF·base Fluoride Reagents: Characterization of Structure, Properties and Reactivity

Stephen G. Sweeting and Alastair J. J. Lennox*

*a.lennox@bristol.ac.uk

School of Chemistry, University of Bristol, Cantock's Close, Bristol, BS8 1TS, UK

Supporting Information

Contents

General Experimental **4**

1. Recent Literature Examples of *n*HF·base Being Used **7**

2. Preparation of *n*HF·base Mixtures **8**

2.1. Determining the Mass Percentage of HF in nHF·base mixtures 8

2.2 Preparing the nHF·base mixtures 9

3. Fluoride Measurements for *n*HF·base Mixtures **12**

3.1. Synthesis and Calibration of Fluoride Ion Selective Electrode (ISE) 12

3.2. Measuring Fluoride Concentrations of nHF·base Mixtures 14

4. Density Measurements for *n*HF·base Mixtures **16**

5. Computational Chemistry **18**

5.1 Optimisation of nHF·base Clusters and Benchmarking 18

5.2 Development of Parameters for CPCM Modelling 55

5.3 Optimisation of Anionic Clusters $[F(HF)_x]^-$ (x = 1-4) 58

5.4 Stability of Anionic Clusters $[F(HF)_x]^-$ (x = 1-4) 66

5.5 Non-Covalent Interaction Analysis of nHF·py and nHF·TEA clusters 68

5.6 Nucleophilicity of anionic clusters 83

5.7 Nucleophilicity of nHF·base clusters 84

5.8 Basicity of the Fluoride Anion in nHF·py and nHF·TEA clusters 136

6. VT-NMR for *n*HF·base Mixtures **143**

7. Determining H_0 for *n*HF·base Mixtures **160**

8. Kinetics **165**

9. Mechanistic Investigations for the Reaction of 4-Nitrobenzyl Bromide in *n*HF·base Mixtures **187**

9.1 Synthesis of chiral 1-(bromomethyl-d)-4-nitrobenzenes 187

9.1.1 Synthesis of (4-nitrophenyl)methan-d-ol 187

9.1.2 Synthesis of 1-(bromomethyl-d)-4-nitrobenzene 189

9.2 Synthesis of chiral 1-(fluoromethyl-d)-4-nitrobenzenes 191

9.2.1 Synthesis of (4-nitrophenyl)methyl-d methanesulfonate 191

9.2.2 Synthesis of 1-(fluoromethyl-d)-4-nitrobenzene 194

9.3 Polarimetry measurements 196

9.4 Kinetic isotope effect measurement 197

10. pK_a Measurements of Bases in *n*HF·base Mixtures 198

11. Comparing Basicity and Nucleophilicity of Fluoride Anion 199

11.1 Synthesis of 3-(4-fluorophenyl)propyl 4-nitrobenzenesulfonate 199

11.2 Selectivity reaction 201

12. Experiments Involving Tetramethylammonium Fluoride (TMAF) 203

12.1 Nucleophilicity of fluoride anion in TMAF 203

12.2 Basicity of fluoride anion in TMAF 207

13. Cost Analysis of Nucleophilic Fluorination Reagents 208

14. NMR Spectra for Novel Compounds 209

15. References 225

General Experimental

Solvents

Deuterated water, deuterated dichloromethane (DCM) and deuterated chloroform were purchased from Merck Life Sciences Ltd. Non-deuterated chloroform, pentane, petroleum ether and ethyl acetate were purchased from Merck Life Sciences Ltd. Solvents were used without further purification. Tetrahydrofuran (THF) and DCM was dried using an Anhydrous Engineering alumina column drying system situated in the University of Bristol's chemistry department. All solvents were collected using Strauss flasks using a gastight J. Youngs valve.

Reagents

Olah's reagent (9HF·py), ethyl trifluoroacetate, trichlorofluoromethane, silver triflate, propylene carbonate, silver wire (⌀ = 0.5 mm), tetramethylammonium fluoride (TMAF), 2-nitroaniline, 3-nitroaniline, 4-nitroaniline, 2-chloro-6-nitroaniline 2,4-dinitroaniline, (1*R*,2*R*)-(−)-N-*p*-Tosyl-1,2-diphenylethylenediamine, (1*S*,2*S*)-(−)-N-*p*-tosyl-1,2-diphenylethylenediamine, (cymene)ruthenium dichloride dimer, 18-crown-6, sodium borohydride, 4-nitrobenzaldehyde, 4-(dimethylamino)pyridine, caesium fluoride, 4-nitrobenzenesulfonyl chloride, isopropyl alcohol and ethanol were purchased from Merck Life Sciences Ltd. Potassium hydroxide, sodium hydrogen carbonate, magnesium sulfate, deuterated formic acid, triethylamine and methanesulfonyl chloride purchased from ThermoFisher Scientific. Pyridine and tetraethylammonium perchlorate (TEAP) with 10% water were purchased from Alfa Aesar. Triethylamine pentahydrofluoride was purchased from Apollo Scientific Ltd. Triethylamine trihydrofluoride was purchased from FluoroChem. 4-Nitrobenzyl bromide, phosphorous tribromide, *tert*-butanol, methanol and potassium fluoride were purchased from Acros. Tetramethylsilame was purchased from Fluka. Silica gel (40 – 63 µm) was purchased from VWR international, and hydrogen fluoride gas (water < 200 ppm) was obtained from Air Liquide. Reagents were used without further purification.

Techniques

Unless stated otherwise, reactions were conducted under an inert atmosphere, where procedures described were performed using traditional Schlenk line techniques. Oven-dried glassware was dried in an oven overnight at 180 °C and then allowed to cool to room temperature under dynamic vacuum (pressures measured were approximately 0.1 mmHg).

Molecular sieves were activated by drying overnight in an oven at 180 °C and then with a flame under dynamic vacuum (on a vacuum line, at pressures up to ~0.1 mmHg). Solvents were removed under vacuum using a rotary evaporator with water bath temperatures up to 40 °C and pressures up to approximately 10 mmHg (diaphragm pump).

TEAP was dried in a vacuum oven from Shel lab (model number: 1425) by heating the sample to 70 °C for 24 hours under vacuum. A Schlenk tube was dried at 180 °C in an oven and allowed to cool under vacuum using Schlenk line apparatus under a flow of dry nitrogen. Transferring the anhydrous TEAP to the dry Schlenk tube was done quickly to minimise moisture exposure against a flow of dry nitrogen. Sample was then stored under vacuum to allow transferal to a glovebox for long-term storage.

Chromatography

Thin layer chromatography (TLC) was performed using aluminium-backed silica gel 60 F_{254} plates and reagent grade solvents. Visualisation was achieved by UV fluorescence (254 nm), and/or basic potassium permanganate stain. Flash column chromatography (FCC) was performed manually, using silica gel (40-63 µm, 230-400 mesh), or using a Biotage Selekt system with Biotage Sfär Silica (60 µm) pre-packed columns. Reagent grade solvents were used in both cases.

Analysis

When investigating the acidity of HF mixtures, carbon NMR experiments were conducted on the Bruker Avance III HD NMR spectrometer (500 MHz) fitted with a 5mm DCH cryo-probe. VT-NMR experiments were conducted on the Jeol ECS300 NMR spectrometer (300 MHz). When characterising novel compounds, proton, carbon, fluorine, and deuterium NMR experiments were conducted on the Bruker Avance III HD NMR spectrometer (400 MHz). All chemical shifts are given in parts per million (ppm) and are all referenced to an internal standard. For proton NMR, the internal standard is the deuterated solvent; and for fluorine NMR the internal standard is trichlorofluoromethane. All measurements were repeated three times with a new HF mixture to minimise errors in producing the mixtures. For deuterium NMR, protonated solvents were used, followed by 80 µL of deuterated solvent. This allows for the deuterium NMR to be referenced, without losing the signal(s) associated with the deuterated product. Coupling constants (*J*) are given in Hertz (Hz). Multiplicities are abbreviated as: s (singlet), d (doublet), t (triplet), q (quartet), m (multiplet) or a combination thereof.

All experiments that involve the ISE were performed on the AutoLab M101 from AutoLab. The potentiostat is controlled using the *Nova 2.1.5* software, provided by AutoLab. All measurements were repeated thrice with a new HF mixture to minimise errors in producing the mixtures as well as to minimise the error in the open circuit potential reading. The open circuit potential was determined when the $\frac{dE}{dt}$ limit reached 1 μVs^{-1}.

Novel compounds were further characterised with the use of mass spectrometry, Infra-red spectroscopy, and polarimetry when the compound feature chirality. Mass spectra were recorded using the electron impact method for ionisation using a Thermo Scientific QExactive gas chromatography mass spectrometer. Infra-red measurements were performed using a Perkin Elmer Spectrum Two FT-IR spectrometer.

Warning: Use of HF reagents

The hazards associated with the handling of solutions containing hydrogen fluoride are well-documented and can be viewed in the MSDS for these mixtures (example from Sigma Aldrich; https://www.sigmaaldrich.com/catalog/product/aldrich/184225). Therefore, personal protection is of utmost importance when handling these mixtures. Standard procedure when using these mixtures involves wearing two pairs of gloves when handling them – a pair of rubber gloves followed by a pair of disposable nitrile gloves. If the gloves encounter HF at any moment, gloves are removed immediately, and the area affected washed thoroughly with Hexafluorine solution™. Calcium glucanoate gel is applied to the affected area and medical attention is sought as soon as possible. It is advised that Hexafluorine solution™ and calcium glucanoate gel is kept nearby for personal use.

Warning: Handling of hydrogen fluoride gas *must* be done by experienced chemists with all the necessary safety arrangements in place.

1. Recent Literature Examples of *n*HF·base Being Used

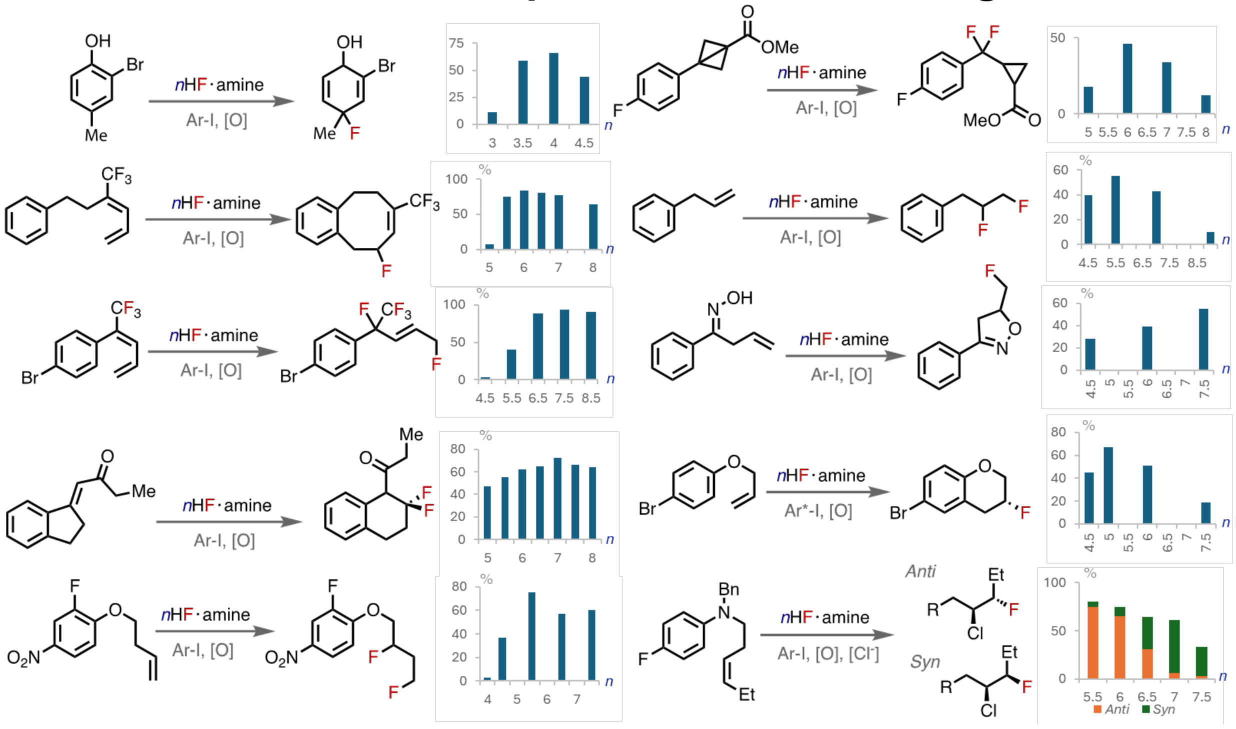

Figure S1: Recent examples of *n*HF·base being used in the literature, along with the distribution of yields obtained for different values of *n*.[1–10]

As can be seen by the distribution of yields for each reaction, the ratio of HF to amine needs to be optimised for these fluorinations. Common optimised ratios tend to be between 4HF·amine and 6HF·amine, which is in accordance with our manuscript, gives an acidity value of -0.8 > H_0 > -1.4. This range also correlated with medium sized fluoride anionic clusters, showing how the nucleophilicity is maximised for these reactions. The acidity is essential for activation of the hypervalent iodine species. For electron-poor substrates, the HF·amine ratio seems to be higher than when electron donating groups are present. The greater acidity of the mixture likely helps with the stabilisation of the hypervalent iodine species. For a given reaction, if the HF·amine ratio is too low, then there is insufficient Brønsted activation or stabilisation of the hypervalent iodine reagent. If the HF·amine mixture is too high, then the reactivity of the fluoride anion is dampened, resulting in diminished yields.

2. Preparation of *n*HF·base Mixtures

2.1. Determining the Mass Percentage of HF in *n*HF·base mixtures

This procedure has been previously described in the literature.[11] A solution of KOH (1.96 g) in deuterated water was produced (10 ml, 3.5 mol dm^{-3}). A 0.5 mL aliquot of this solution was added to a 25 mL HDPE vial equipped with a stir bar. The aliquot was then cooled to 0 °C before adding 25 μL of the HF reagent and stirred for five minutes. To this, 15 μL of ethyl trifluoroacetate was added. The solution was then transferred to a fluorinated ethylene propylene (FEP) NMR tube liner, which was inserted into an NMR tube charged with 0.1 mL trichlorofluoromethane, which was used as the internal reference. ^{1}H and ^{19}F NMR were conducted to determine the mass percentage of both hydrogen fluoride and base present, with typical hydrogen fluoride mass percentages shown in **Table S1**. This procedure was repeated throughout the study, as other HF reagents are known to lose HF overtime through evaporation. By constantly monitoring the concentrations of HF, accurate ratios could be obtained for experimental investigations.

Table S1: Concentration of HF determined for commercially available mixtures of 9HF·py, 5HF·TEA and 3HF·TEA.

Commercially available HF reagent	Supplier	Typical concentration of HF (wt %)
9HF·py	Sigma-Aldrich	69.5
5HF·TEA	Apollo Scientific	49.7
3HF·TEA	FluoroChem	37.2

2.2 Preparing the *n*HF·base mixtures

*n*HF·py and *n*HF·TEA

Base, either pyridine or triethylamine, is added to either 9HF·py or 5HF·TEA, respectively to produce different *n*HF·py and *n*HF·TEA mixtures. When producing these mixtures, first the base was added to a 25 mL HDPE vial, and cooled to 0 °C. The addition of the commercially available *n*HF·base mixtures was added *slowly*, due to the large exotherm generated through this process. Once all the HF reagent was added to the HDPE vial, the solution was allowed to stir for 30 minutes at 0 °C, producing a homogenous solution. A rubber suba-seal was placed over the opening of the HDPE vial to prevent loss of HF. The volumes of base and HF reagent for *n*HF·py and *n*HF·TEA are recorded in **Table S2** and **Table S3**, respectively.

Table S2: Volumes of 9HF·py and pyridine used to produce different *n*HF·py solutions.

*n*HF·py	Volume of 9HF·py / mL	Volume of pyridine / mL
9	1.000	0.000
8	1.000	0.025
7	1.000	0.079
6	1.000	0.151
5	1.000	0.252
4	1.000	0.404
3	1.000	0.656
2	1.000	1.161
1	1.000	2.677

Table S3: Volumes of 5HF·TEA and triethylamine used to produce different *n*HF·TEA solutions.

*n*HF·TEA	Volume of 5HF·TEA / mL	Volume of TEA / mL
5	1.000	0.000
4	1.000	0.171
3	1.000	0.457
2	1.000	1.027
1	1.000	2.740

***n*HF·amine**

Analogous methodology was used to produce different *n*HF·amine mixtures. For a given volume of 9HF·py (V_{Olah}) and a given volume of 3HF·TEA ($V_{TREAT\text{-}HF}$), the mass of HF, pyridine and triethylamine can be determined from the density (ρ_{Olah} and $\rho_{TREAT\text{-}HF}$; available from the supplier), and through knowing the weight percentage of HF in each reagent ($wt\%_{Olah}$ and $wt\%_{TREAT\text{-}HF}$). The total number of moles of HF can then be calculated, followed by the total moles of base present in the mixture, allowing for the value of *n* to be calculated for *n*HF·amine, as shown in **Equation S1**:

$$n = \frac{\left(\frac{wt\%_{Olah}\ V_{Olah}\ \rho_{Olah}}{20.01}\right) + \left(\frac{wt\%_{TREAT\text{-}HF}\ V_{TREAT\text{-}HF}\ \rho_{TREAT\text{-}HF}}{20.01}\right)}{\left(\frac{V_{Olah}\ \rho_{Olah}(1 - wt\%_{Olah})}{79.1}\right) + \left(\frac{V_{TREAT\text{-}HF}\ \rho_{TREAT\text{-}HF}(1 - wt\%_{TREAT\text{-}HF})}{101.19}\right)} \qquad \textbf{Equation S1}$$

When preparing the *n*HF·amine mixtures, the volume of 3HF·TEA required was first added to the 25 mL HDPE vial and cooled to 0 °C, followed by the *slow* addition of 9HF·py. Solutions were allowed to stir for 30 minutes at 0 °C to obtain a homogenous solution, with the HDPE vial capped using a rubber suba-seal as described previously. The volumes of 9HF·py and 3HF·TEA to produce *n*HF·amine are shown in **Table S4**.

Table S4: Volumes of 9HF·py and 3HF·TEA used to produce different nHF·amine solutions.

*n*HF·amine	Volume of 9HF·py / mL	Volume of 3HF·TEA / mL
8	1.000	0.082
7	1.000	0.281
6	1.000	0.613
5	1.000	1.277
4	1.000	3.027

We validated our mixtures by determining the mass percentage in our mixtures using the method described in *section 1.1*. The plot shown in **Figure S2** show that our mixtures were in line with what the theoretical values of n would be for any mixture, confirming our methodology to be correct.

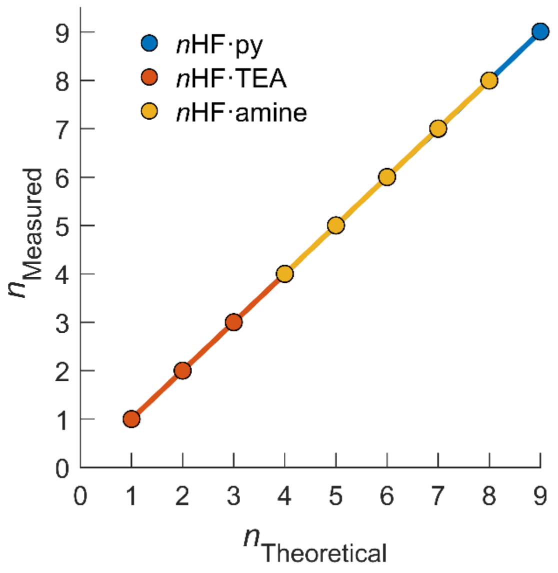

Figure S2: The measured *n* vs the calculated *n* used to prepare the mixtures.

3. Fluoride Measurements for *n*HF·base Mixtures

3.1. Synthesis and Calibration of Fluoride Ion Selective Electrode (ISE)

This method has been reported previously for HF mixtures.[12] The open circuit potential (V_{ocp}) was measured using a Ag/AgOTf double-junction reference electrode, previously described in the literature to prevent the precipitation of fluoride salts.[13] A diagram for this reference electrode is shown in **Figure S3**. When the reference electrode is not in use, it is stored in a 0.1M tetraethylammonium perchlorate (TEAP) solution in propylene carbonate (PC).

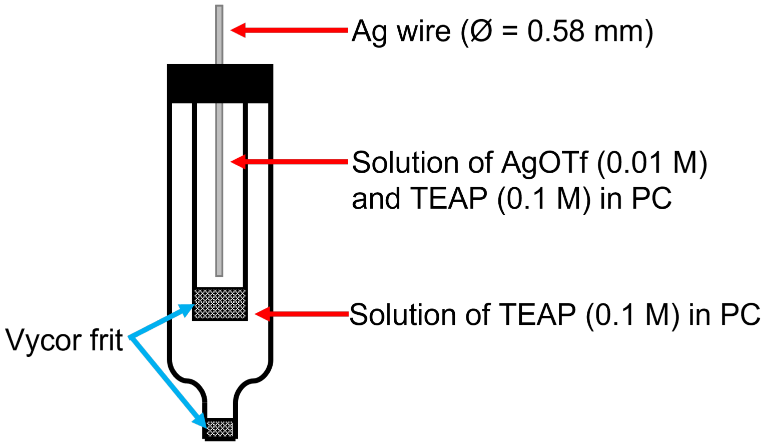

Figure S3: A diagram of the Ag/AgOTf reference electrode that was used for all the fluoride measurements.

For the fluoride ISE, silver wire was submerged in 9HF·py for five days. The wire was held in place by pushing it through a suba-seal, which was able to cover the top of a 25 ml-HDPE vial containing the HF mixture. After five days, a brown-black precipitate was present on the surface of the wire (**Figure S4**), indicating the synthesis of silver fluoride upon the surface of the wire.

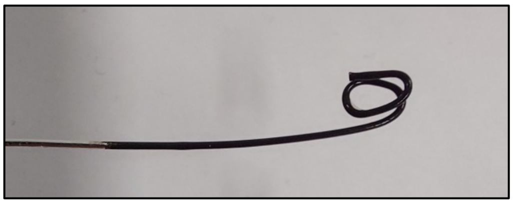

Figure S4: The Ag|AgF ISE synthesised by subjecting silver wire to Olah's reagent for five days.

The calibration curve was determined for fluoride concentrations in the range 10^{-2}M to 10^{-4}M, as this has been determined to be the range that the ISE can function successfully.[13] The

calibration curve (**Figure S5**) was produced through serial dilution of a 10^{-2} M tetramethylammonium fluoride solution with a 10^{-1} M tetraethylammonium perchlorate in PC.

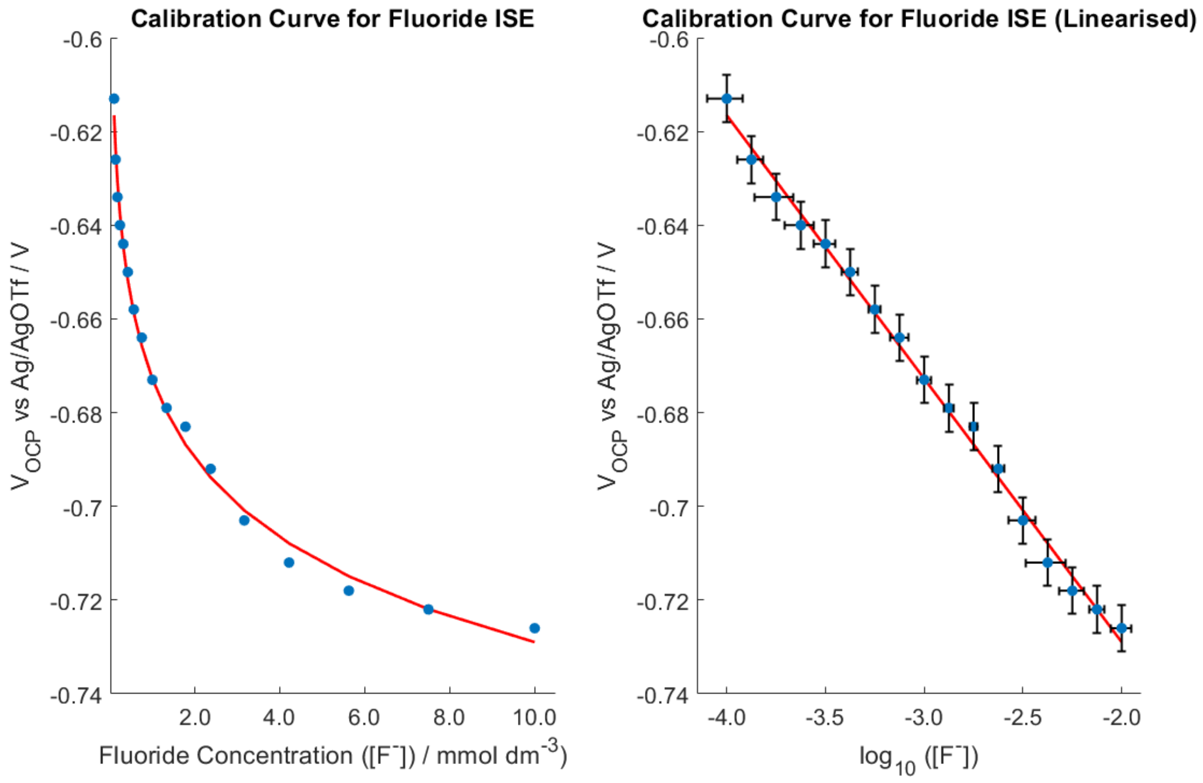

Figure S5: The calibration curve for the fluoride measurements when considering solutions with concentrations between 10^{-4} M and 10^{-2} M. **a**) The collected data along with the curve of best fit. **b**) The linearised form of the same data, where the equation of the line is given in **Equation S1**.

Data fitting was performed via MATLAB software using the function described in **Equation S1**, where $[F^-]$ refers to the concentration of fluoride within the solution.

$$V_{OCP} = -0.8415 - 0.0562 \log_{10}[F^-]$$ **Equation S1**

3.2. Measuring Fluoride Concentrations of *n*HF·base Mixtures

To measure the fluoride concentrations, a 150 µL aliquot of the *n*HF·base mixture was mixed with 5 ml of propylene carbonate (PC) for five minutes. The V_{OCP} (vs the Ag/AgOTf reference electrode) was then measured using the AutoLab M101 potentiostat. The fluoride concentrations are recorded in **Table S5 – S7**.

Table S5: The measured open circuit potential (V_{OCP}) for *n*HF·py mixtures, along with the determined fluoride anion concentrations according to **Equation S1**.

*n*HF·py	V_{OCP} of solution / V	[F$^-$] in PC solution / M	[F$^-$] in neat *n*HF·base mixture / M
1	-0.732	1.13E-02	11.3
2	-0.727	9.21E-03	9.22
3	-0.722	7.50E-03	7.51
4	-0.719	6.63E-03	6.64
5	-0.715	5.63E-03	5.64
6	-0.714	5.41E-03	5.41
7	-0.712	4.98E-03	4.99
8	-0.711	4.78E-03	4.79
9	-0.707	4.06E-03	4.06

Table S6: The measured open circuit potential (V_{OCP}) for *n*HF·TEA mixtures, along with the determined fluoride anion concentrations according to **Equation S1**.

*n*HF·TEA	V_{OCP} of solution / V	[F$^-$] in PC solution / M	[F$^-$] in neat *n*HF·base mixture / M
3	-0.722	7.50E-03	7.51
4	-0.719	6.63E-03	6.64
5	-0.715	5.63E-03	5.64

Table S7: The measured open circuit potential (V_{OCP}) for *n*HF·amine mixtures, along with the determined fluoride anion concentrations according to **Equation S1**.

*n*HF·amine	V_{OCP} of solution / V	[F⁻] in PC solution / M	[F⁻] in neat *n*HF·base mixture / M
4	-0.719	6.63E-03	6.64
5	-0.715	5.63E-03	5.64
6	-0.714	5.41E-03	5.41
7	-0.712	4.98E-03	4.99
8	-0.711	4.78E-03	4.79

4. Density Measurements for *n*HF·base Mixtures

To determine the density of the different *n*HF·base mixtures, gravimetric techniques were used. Fresh *n*HF·base mixtures were used for all analyses, which were produced on a 5 mL scale. An HDPE vial with lid and parafilm was placed on a balance, and the balance was tared. Using a syringe fitted with a long needle, 0.5 mL of the *n*HF·base mixture was transferred to the HDPE vial, which was then closed and sealed using parafilm. The mass of the sealed HDPE vial featuring the aliquot of *n*HF·base was then recorded. This procedure was repeated three times for each mixture, and the average mass of the *n*HF·base mixture aliquot was determined. From the average mass ($\bar{m}$), the density (ρ) could be determined using **Equation S2**:

$$\rho = \frac{\bar{m}}{V}$$ **Equation S2**

where V is the volume of the solution being measured (0.5 mL). Densities are shown in **Tables S8 – S10**.

Table S8: The measured masses of different *n*HF·py mixtures, along with the calculated average mass and associated density.

*n*HF·py mixture	Mass 1 / g	Mass 2 / g	Mass 3 / g	Average mass ($\bar{m}$) / g	Density (ρ) / g cm^{-3}
1	0.54001	0.53995	0.54035	0.54010	1.08
2	0.56175	0.56168	0.56170	0.56171	1.12
3	0.59670	0.59670	0.59670	0.59670	1.19
4	0.62230	0.62221	0.62235	0.62229	1.24
5	0.61775	0.61755	0.61786	0.61772	1.24
6	0.60790	0.60775	0.60788	0.60784	1.22
7	0.59602	0.59600	0.59567	0.59590	1.19
8	0.57820	0.57820	0.57820	0.57820	1.16
9	0.55003	0.55003	0.55003	0.55003	1.10

Table S9: The measured masses of different *n*HF·TEA mixtures, along with the calculated average mass and associated density.

*n*HF·TEA mixture	Mass 1 / g	Mass 2 / g	Mass 3 / g	Average mass ($\bar{m}$) / g	Density (ρ) / g cm^{-3}
3	0.49505	0.49481	0.49594	0.49527	0.99
4	0.50055	0.50013	0.49752	0.49940	1.00
5	0.51891	0.51725	0.51807	0.51808	1.04

Note: 1HF·TEA and 2HF·TEA are solids at room temperature, and therefore their densities cannot be determined with this methodology.

Table S10: The measured masses of different *n*HF·amine mixtures, along with the calculated average mass and associated density.

*n*HF·amine mixture	Mass 1 / g	Mass 2 / g	Mass 3 / g	Average mass ($\bar{m}$) / g	Density (ρ) / g cm^{-3}
4	0.52280	0.52280	0.52280	0.52280	1.05
5	0.53865	0.53832	0.53878	0.53858	1.08
6	0.53800	0.53811	0.53769	0.53793	1.08
7	0.54287	0.54251	0.54296	0.54278	1.09
8	0.54727	0.54749	0.54759	0.54745	1.09

5. Computational Chemistry

5.1 Optimisation of *n*HF·base Clusters and Benchmarking

Computational studies of all *n*HF·base were initially generated using the Conformer-Rotamer Ensemble Sampling Tool (CREST) software (Version 3.0).[14] The non-covalent interaction conformational sampling tool was used within the CREST software to generate the ensembles, using the GFN2-xTB level of theory.[15]

With the best ensembles recognised, the geometries of each ensemble were used for both density functional theory (DFT) and domain-based local pair natural orbital coupled-cluster (DLPNO-CCSD) level calculations, both available through ORCA 6.0.[16] When performing optimisation calculations, a range of different functionals were investigated for benchmarking studies. Functionals that were investigated in this study include B2-PLYP-D4,[17–19] B3LYP,[20,21] B3LYP-D4,[18–21] BP86,[22,23] cam-B3LYP,[24] cam-B3LYP-D4,[18,19,24] LC-PBE,[25–28] M06-2X,[29] RevDSD-PBEP86-D4,[30] RevPBE,[31] RevPBE0,[32] RevPBE0-D4,[18,19,32] RevPBE38,[32–36] RevPBE38-D4,[18,19,32–36] RevPBE-D4 [18,19,31] and ωB97X-D4.[18,19,37] All calculations made use of the def2-TZVPPD basis set,[38,39] and implicit solvent was modelled using the conductor polarisable continuum model (CPCM)[40] framework (see section 6.2 for further details). Optimisation calculations were performed on all *n*HF·base ensembles. Stationary points after optimisation calculations were characterised through their harmonic vibrational frequencies as minima, where ground states can be identified through a lack of imaginary vibrational frequencies.

When calculating the DLPNO-CCSD values, single point energy calculations were performed on all *n*HF·base ensembles. Geometries optimised at the DFT level were used as inputs for the DLPNO-CCSD calculations. To determine the most suitable functional, we analysed three metrics: the largest absolute error (LAE; **Equation S3**), the mean absolute error (MAE; **Equation S4**) and the root mean square deviation (RMSD; **Equation S5**). Plots of these metrics are shown in **Figure S6 – S17** and show that the LC-PBE functional is most suitable for describing the *n*HF·py and nHF·TEA clusters, and their coordinates are given within.

$$\mathrm{LAE} = \max_{n=i}\left|x_{\mathrm{DFT},\,i} - x_{\mathrm{DLPNO\text{-}CCSD},\,i}\right| \qquad \textbf{Equation S3}$$

$$\mathrm{MAE} = \frac{\sum_{i=1}^{N}\left|x_{\mathrm{DFT},\,i} - x_{\mathrm{DLPNO\text{-}CCSD},\,i}\right|}{N} \qquad \textbf{Equation S4}$$

$$\mathrm{RMSD} = \sqrt{\frac{\sum_{i=1}^{N}\left(x_{\mathrm{DFT},\,i} - x_{\mathrm{DLPNO\text{-}CCSD},\,i}\right)^2}{N}} \qquad \textbf{Equation S5}$$

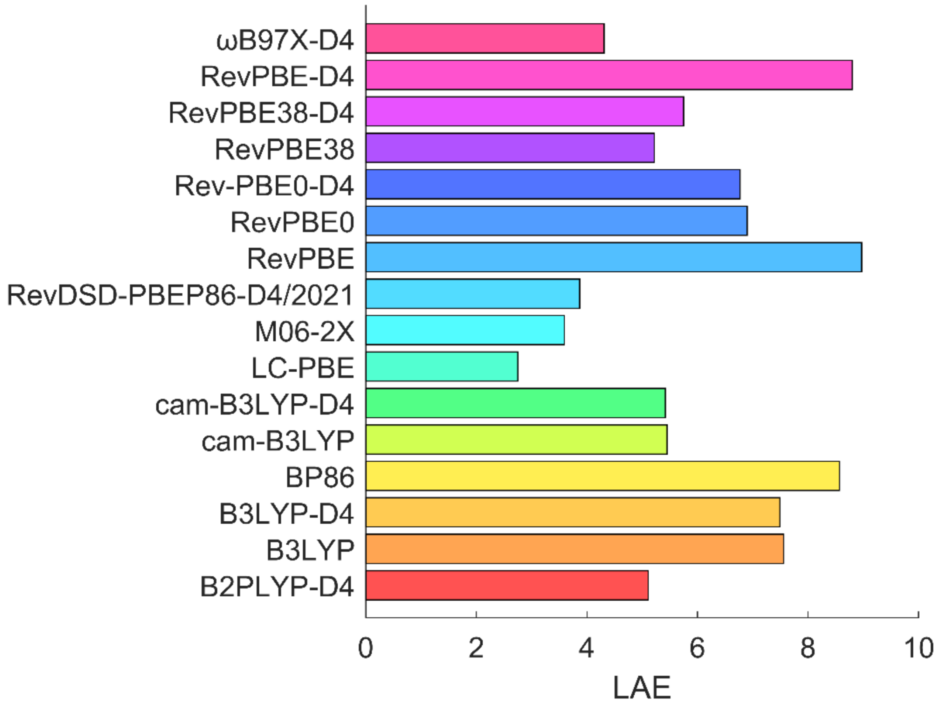

Figure S6: The LAE for all DFT functionals being considered within the study when compared to DLPNO-CCSD calculations for nucleophilicity using **Equation S22**.

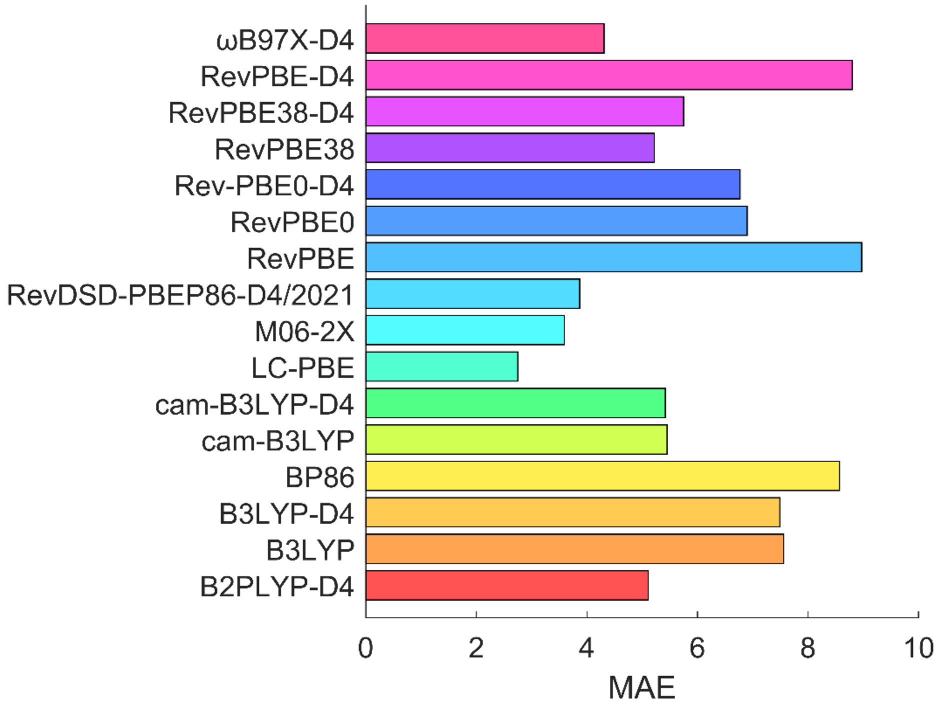

Figure S7: The MAE for all DFT functionals being considered within the study when compared to DLPNO-CCSD calculations for nucleophilicity using **Equation S22**.

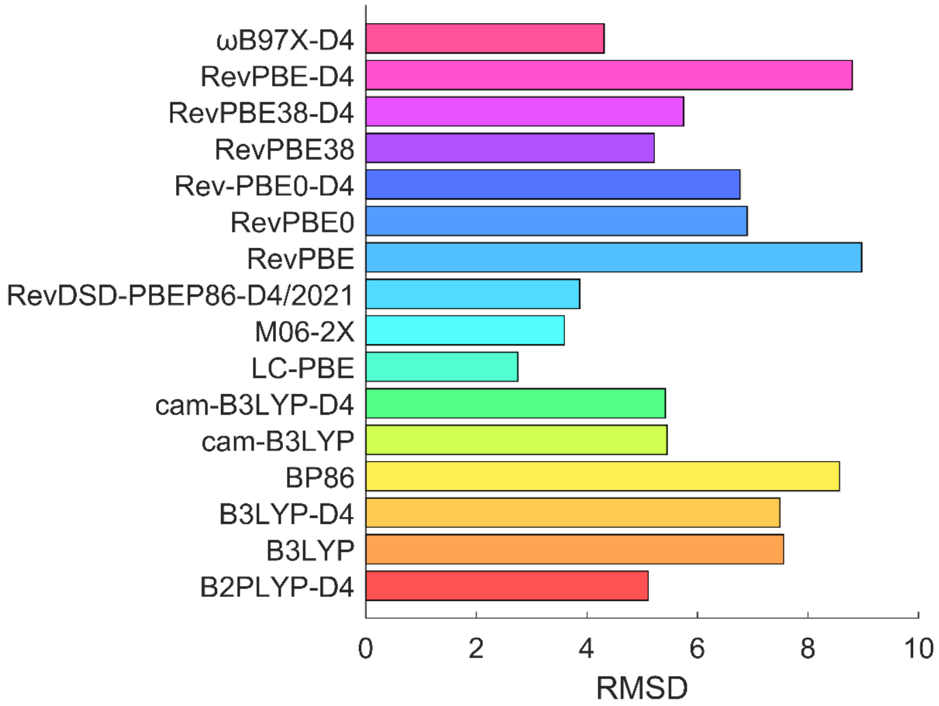

Figure S8: The RMSD for all DFT functionals being considered within the study when compared to DLPNO-CCSD calculations for nucleophilicity using **Equation S22**.

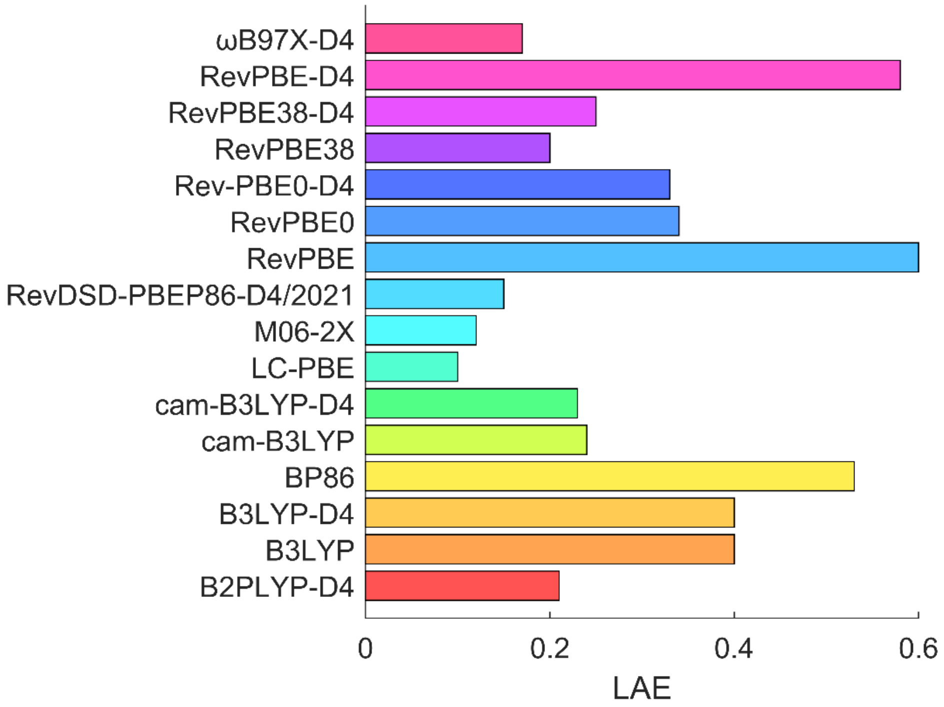

Figure S9: The LAE for all DFT functionals being considered within the study when compared to DLPNO-CCSD calculations for nucleophilicity using **Equation S23**.

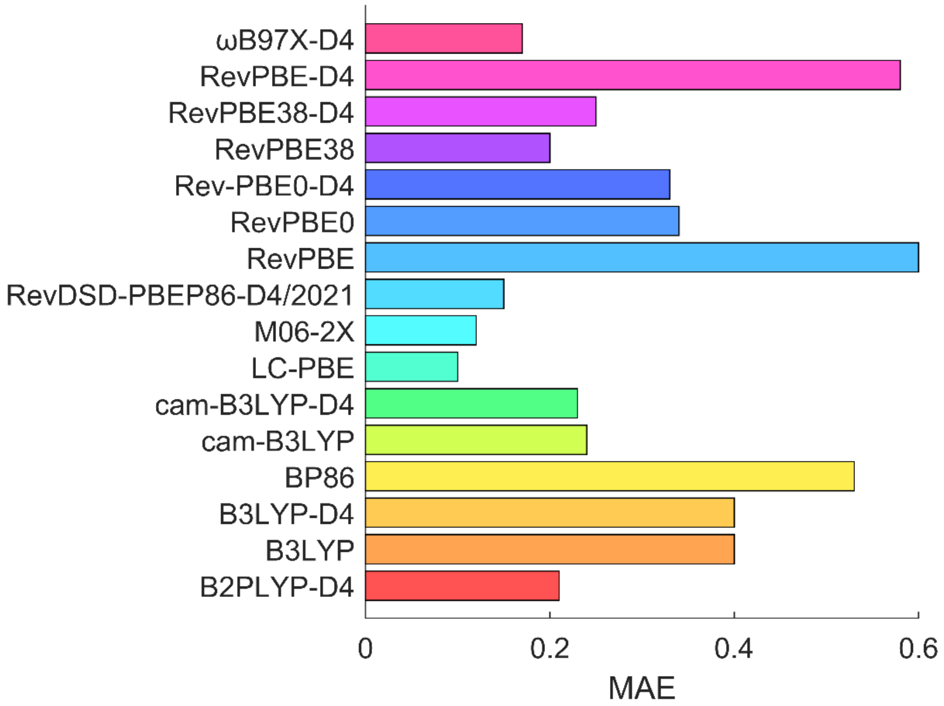

Figure S10: The MAE for all DFT functionals being considered within the study when compared to DLPNO-CCSD calculations for nucleophilicity using **Equation S23**.

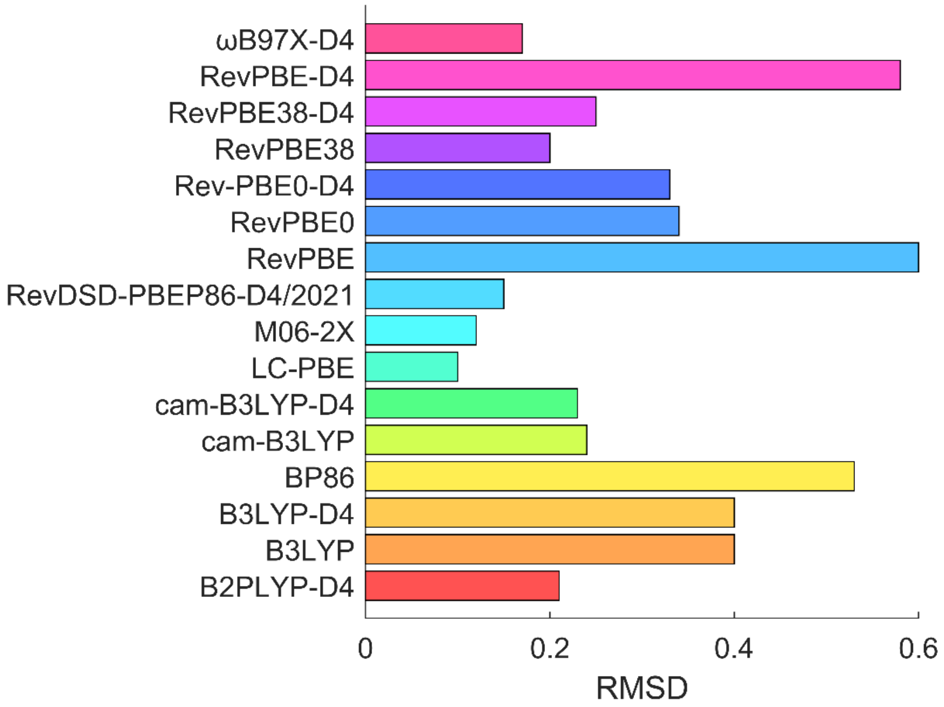

Figure S11: The RMSD for all DFT functionals being considered within the study when compared to DLPNO-CCSD calculations for nucleophilicity using **Equation S23**.

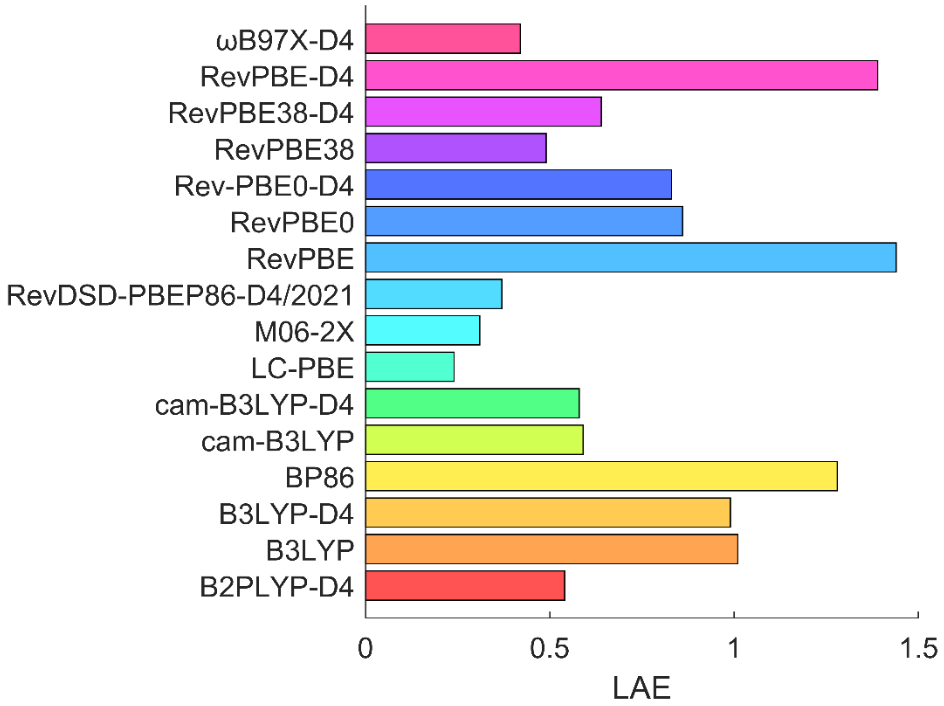

Figure S12: The LAE for all DFT functionals being considered within the study when compared to DLPNO-CCSD calculations for nucleophilicity using **Equation S24**.

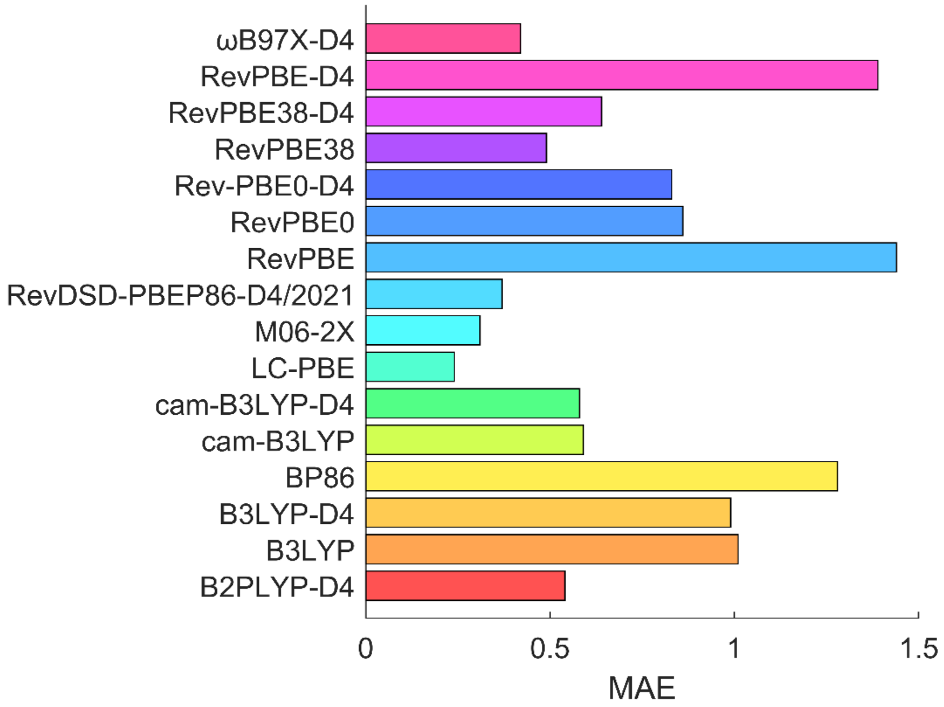

Figure S13: The MAE for all DFT functionals being considered within the study when compared to DLPNO-CCSD calculations for nucleophilicity using **Equation S24**.

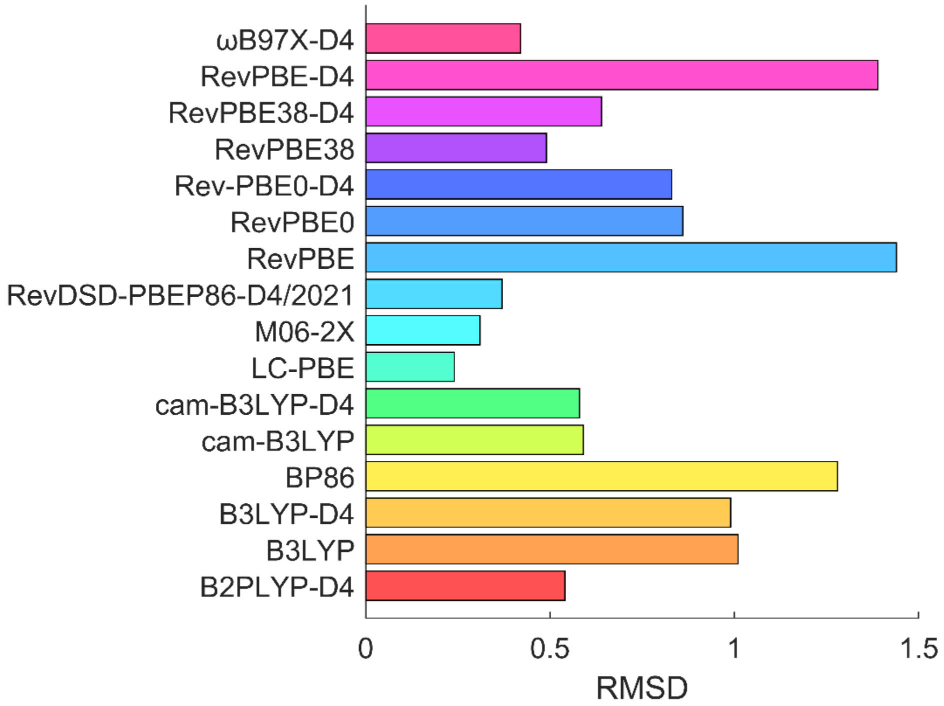

Figure S14: The RMSD for all DFT functionals being considered within the study when compared to DLPNO-CCSD calculations for nucleophilicity using **Equation S24**.

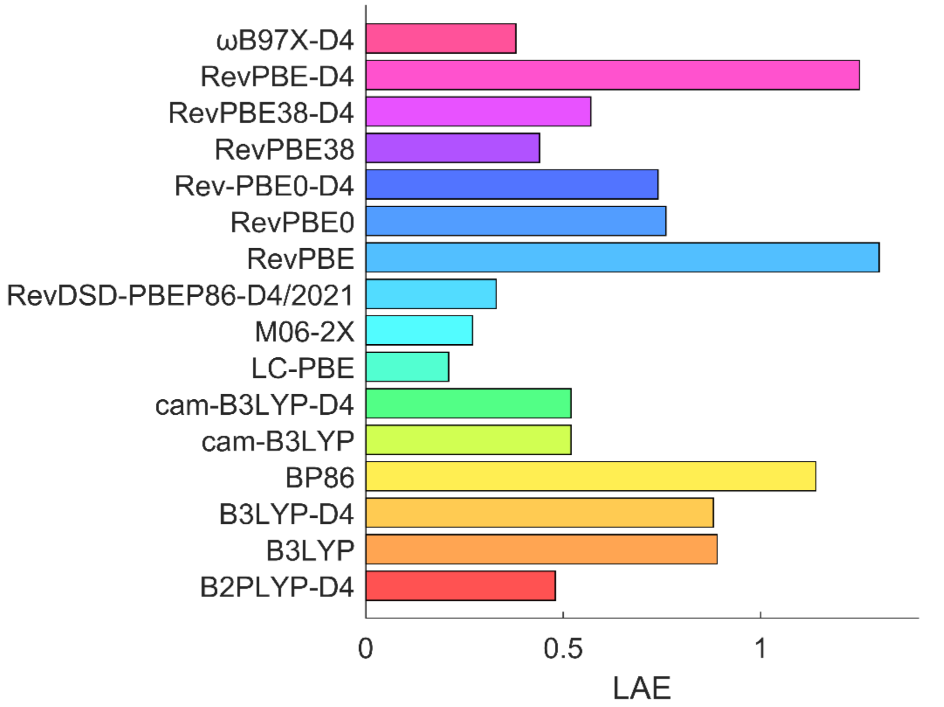

Figure S15: The LAE for all DFT functionals being considered within the study when compared to DLPNO-CCSD calculations for nucleophilicity using **Equation S25**.

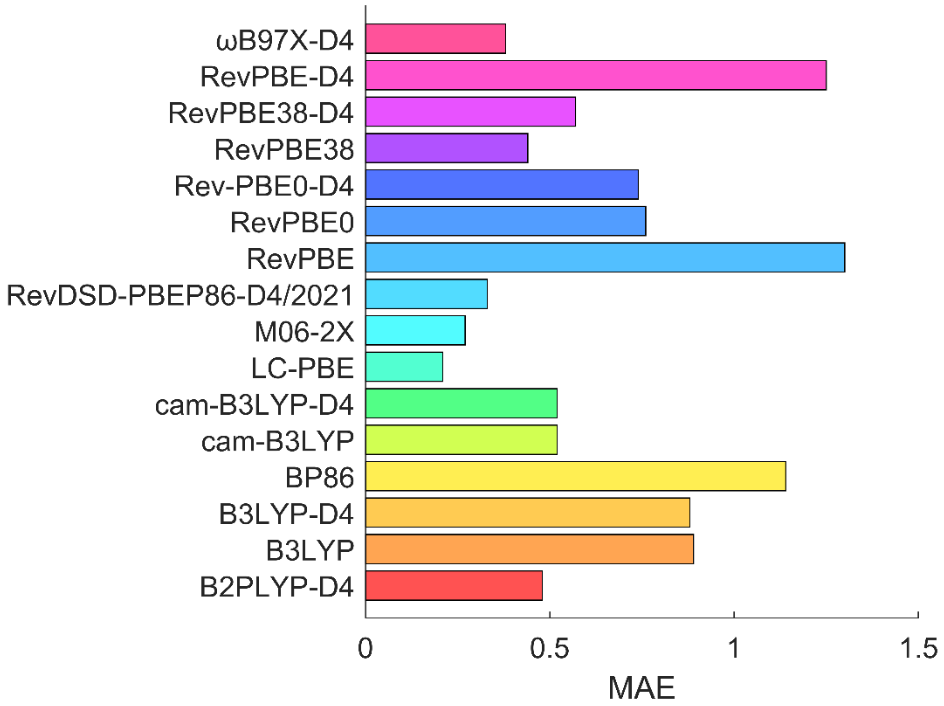

Figure S16: The MAE for all DFT functionals being considered within the study when compared to DLPNO-CCSD calculations for nucleophilicity using **Equation S25**.

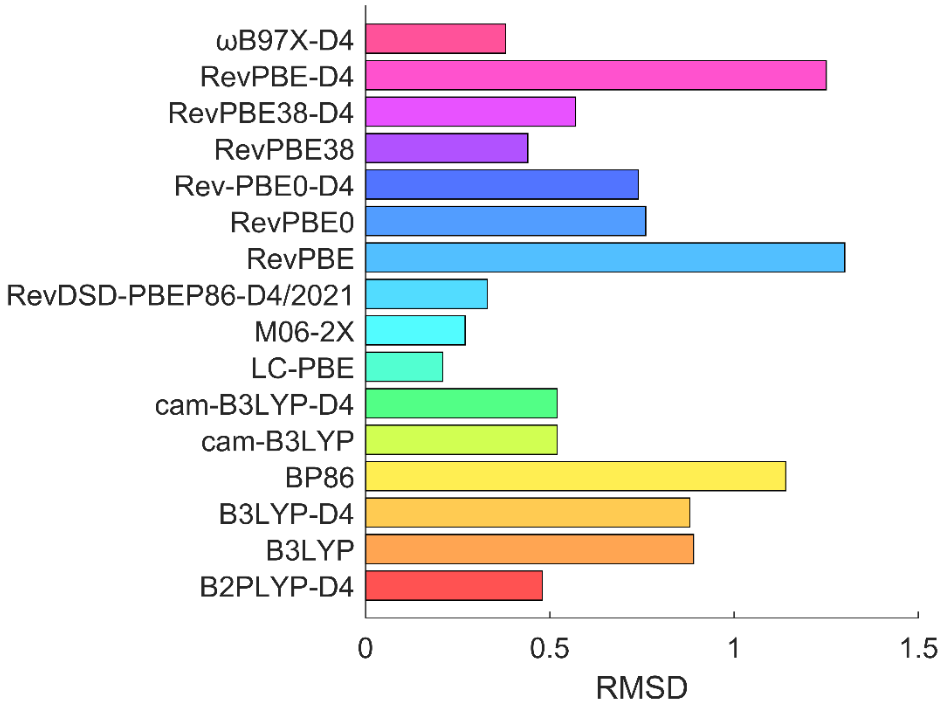

Figure S17: The RMSD for all DFT functionals being considered within the study when compared to DLPNO-CCSD calculations for nucleophilicity using **Equation S25**.

Cartesian coordinates

1HF·py (DFT/LC-PBE/def2-TZVPPD+CPCM(High dielectric))

C	-2.12987495629253	-0.00007330494442	-0.00006034852074
C	-1.43553106359174	1.18556259556430	-0.00002885834710
C	-0.06676064912835	1.14188353658952	0.00002700470372
C	-0.06665744336622	-1.14184881589010	0.00001727914859
C	-1.43542734222036	-1.18564887913268	-0.00004114994405
H	-3.21376657349200	-0.00012012055427	-0.00009993412383
H	-1.94613850699915	2.13975916545204	-0.00004377215799
H	0.53023898811129	2.04752269813014	0.00004802183903
H	0.53042424469906	-2.04743520705539	0.00004055796361
H	-1.94594627489510	-2.13989334933447	-0.00006769209134
N	0.59545261145882	0.00004670444950	0.00004763498020
F	3.01629977808658	0.00008721127215	-0.00005246750587
H	1.96195056722971	0.00008268185369	0.00005383965578

2HF·py (DFT/LC-PBE/def2-TZVPPD+CPCM(High dielectric))

C	-2.80465060965018	0.46251263275051	-0.00850968938774
C	-1.81764513872203	1.41972296636010	0.00682826199291
C	-0.51434108710513	1.01519408088813	0.01619365040808
C	-1.14542198869563	-1.21022774824768	-0.00402913216951
C	-2.46706176945119	-0.87039113179268	-0.01400275902579
H	-3.84731773245345	0.75833198021234	-0.01620485917195
H	-2.05332849299264	2.47537913690971	0.01146924521639
H	0.32017241784743	1.70547262918483	0.02824426051285
H	-0.79786548015241	-2.23592750965521	-0.00741644481897
H	-3.22159244141137	-1.64527224044849	-0.02593570029676
N	-0.21336968406378	-0.27245205606005	0.01056931554056
F	2.17710531157881	-0.96668508985125	0.02701307777050
H	0.83206096262328	-0.56767252764835	0.01792187531236
F	3.82151933586927	0.63796948420843	-0.03682452489211
H	3.08588378157901	-0.07343572421035	-0.00862203259081

3HF·py (DFT/LC-PBE/def2-TZVPPD+CPCM(High dielectric))

C	-3.20849171976328	-0.00005568521580	-0.00063983937071
C	-2.52121814911738	1.19114949706388	-0.00134884059374
C	-1.15795161236700	1.16010154888248	-0.00082179074569
C	-1.15781626425229	-1.15992784184501	0.00105925898922
C	-2.52107206879086	-1.19117175636126	0.00057668247431
H	-4.29226851862456	-0.00011570099575	-0.00105618605594
H	-3.03660417812768	2.14204076430166	-0.00230838790691
H	-0.54099146764731	2.04991664227551	-0.00131455661525
H	-0.54074323769977	-2.04966042380374	0.00202217112226
H	-3.03633408894460	-2.14213232265951	0.00115803903491
N	-0.52085099452092	0.00012194689495	0.00034490177124
F	2.06885579395289	0.00000301557465	0.00107572031164
H	0.53256220558846	0.00013860931558	0.00066626035526
F	3.25620857574388	2.03166602209687	0.00937545949135
H	2.76195164686331	1.17752533119075	0.00577839389707
H	2.76170680117443	-1.17768054966256	-0.00233949235518
F	3.25574990506561	-2.03193562596308	-0.00484742132297

4HF·py (DFT/LC-PBE/def2-TZVPPD+CPCM(High dielectric))

C	-3.38417912164437	0.62669971797807	0.19477337323776
C	-2.77019233790840	-0.42393651229221	0.83577331704159
C	-1.40950933891555	-0.50283562411489	0.80744806801037
C	-1.27181188880458	1.44202078278103	-0.45522703106199
C	-2.62880604941476	1.57182275814408	-0.45933452297612
H	-4.46472606656425	0.71025020755210	0.20541341067036
H	-3.34066127738037	-1.18069387156244	1.35692926915575
H	-0.84576389656172	-1.29424851990530	1.28496005735275
H	-0.60246121994648	2.13912914276403	-0.94321931876582
H	-3.08646867246464	2.40757488850028	-0.97103216520957
N	-0.70711169156485	0.42126626272448	0.17094134147888
F	1.92785496023199	0.26279202534696	0.14451886141494
H	0.33351563480571	0.34425067429491	0.16167975879215
F	2.88652491220884	-1.77735420688233	-0.17629541790789
H	2.44326605679518	-0.83454322984697	-0.02890685728484
H	2.72873468665255	1.41870109029034	-0.01665415220594
F	3.26804572750521	2.22152924879566	-0.12400814026326
F	1.38648907905221	-3.60338597175102	-0.62674065201693
H	1.97989864603312	-2.86118628770702	-0.44286035057731

5HF·py (DFT/LC-PBE/def2-TZVPPD+CPCM(High dielectric))

C	-3.62824190971959	0.02264236365766	0.01423282826709
C	-2.94720253716091	0.01571881875948	1.20924083610936
C	-1.58435936306097	0.00640105639501	1.18594686251486
C	-1.57309194709068	0.01050433907203	-1.13763583101367
C	-2.93564241854321	0.01994605533798	-1.17412365961207
H	-4.71196463506605	0.03039998944358	0.00894609191879
H	-3.46726712374158	0.01770904370143	2.15751881241808
H	-0.97039058967973	0.00078492831416	2.07757546608932
H	-0.95045713245760	0.00805382311370	-2.02325109755058
H	-3.44649417362183	0.02530818507884	-2.12738095270443
N	-0.94379200979871	0.00410989995943	0.02721001314416
F	1.72313926333890	-0.01031891641487	0.03259360079166
H	0.09521251497448	-0.00204042707991	0.03186846412469
F	2.89891391015622	-1.99054891652148	0.02596862976228
H	2.37746578350862	-1.11111229528217	0.02804515577858
H	2.12942157280649	3.18585077219352	-0.05413698732222
F	1.63095157800111	4.00650243557058	-0.11464121972633
F	1.58525552085711	-4.02626461058236	-0.12922422915037
H	2.09304136253820	-3.21145431581252	-0.06742446752498
F	2.92167100016694	1.95616745918987	0.03754185964222
H	2.39094700642549	1.08235204783395	0.03448283479230

6HF·py (DFT/LC-PBE/def2-TZVPPD+CPCM(High dielectric))

C	-4.24246463357458	1.13883577281946	0.28201588528334
C	-3.65047840155587	0.04187409120656	0.86338506063627
C	-2.29256238500033	-0.06708572495185	0.82473196150875
C	-2.11501126654105	1.94313410172547	-0.33517222679781
C	-3.46864178585194	2.10094628970714	-0.32427987061968
H	-5.32083581296524	1.24571575491294	0.30111868031790
H	-4.23609758228668	-0.72890806257176	1.34556278662698
H	-1.73922610223820	-0.89239516113509	1.25234441678758
H	-1.42750311644614	2.64489987996184	-0.78744582835974
H	-3.90998044534861	2.97226055576562	-0.78871905843127
N	-1.57541382398008	0.87628001806425	0.23400974288093
F	2.87144216960212	-0.87077111082190	0.19241200836874
H	-0.55582422659695	0.77103820046763	0.21604272261878
F	3.62811334985746	-2.91564868160065	-0.62021982066996
H	3.29847809913745	-2.03264202527178	-0.27277090592517
H	1.53836320491551	-0.74552853332534	0.69408292974551
F	0.62259011204350	-0.62712067260950	0.99695570105692
F	2.17034540243569	-4.83121720232712	-0.20238529610512
H	2.73345561200998	-4.07128772024990	-0.35962393941047
F	3.02578050664744	1.10507410340895	-1.00353348304745
H	3.01211905448115	0.22271102462415	-0.50526336539083
F	0.81137525755640	2.01364100414951	-0.67512996045043
H	1.70519324806656	1.67413129897814	-0.81969013378327

7HF·py (DFT/LC-PBE/def2-TZVPPD+CPCM(High dielectric))

C	-4.35681470191017	-0.39139913415911	0.04305170710399
C	-3.70076168889566	-0.53273394428602	-1.15770972264828
C	-2.38265905462201	-0.87886862064373	-1.14763722261108
C	-2.36744917386032	-0.94325430410255	1.17917672732671
C	-3.68530151203564	-0.59890257092124	1.22545827082833
H	-5.40504373131300	-0.11613676557284	0.05742573669185
H	-4.20597489535196	-0.37525607536835	-2.10083922567642
H	-1.78375923891536	-1.01335118287736	-2.03836965720761
H	-1.75690608377763	-1.12564598517181	2.05328869220425
H	-4.17813761179671	-0.49425035883164	2.18237628603188
N	-1.76450525093245	-1.07135343029132	0.00737743986097
F	3.09513341798335	0.83478691885196	-0.00400030004233
H	-0.77219038192053	-1.32830761559135	-0.00730592519993
F	2.32512046979889	-0.16803612939210	1.95752589033163
H	2.66235777021964	0.27473252774891	1.12415165699456
H	3.18844389752746	2.18089841539823	-0.08157380966053
F	3.26761702316392	3.17050587971240	-0.14113325123557
F	0.50036868464632	-1.70589970186285	1.52790368826804
H	1.22676077099378	-1.09258302022719	1.69250258604584
F	2.43035520615442	-0.37279844733903	-1.88561257683222
H	2.71878355105109	0.15543447472941	-1.08364552996797
F	1.14960052178866	4.40539628640793	-0.09718054849134
H	1.96873299170630	3.91107533477625	-0.11134826545591
F	0.44993128196168	-1.72910745114206	-1.55278596410161
H	1.23779850173024	-1.18492913973760	-1.67330996598311

8HF·py (DFT/LC-PBE/def2-TZVPPD+CPCM(High dielectric))

C	3.47326222256451	1.94995678042172	0.25828118179588
C	3.13287189047990	0.80657223699847	-0.41113185599601
C	2.16220910054852	0.00377696991074	0.11973723099693
C	1.87724426226121	1.43738284430888	1.93497188179122
C	2.83926711034588	2.27417670547474	1.44561718051773
H	4.24157118689378	2.60210169370648	-0.14972570064282
H	3.61560644290084	0.53243212126772	-1.34432554560229
H	1.83186838117188	-0.91505088671309	-0.33887614762661
H	1.32807851689133	1.61362436348998	2.85906794709036
H	3.08820527978678	3.16995948139149	1.99218134782891
N	1.57125453775604	0.34223751499758	1.26608847456317
F	-2.86307346740011	-0.25862019269818	-0.68946905666901
H	0.83797867477303	-0.27730078715236	1.64780284252922
F	-2.61442065892177	2.04808960312927	-1.11180583817271
H	-2.71194505819888	1.07718995899938	-0.93308065237555
H	-2.71360699092414	-0.68991167252558	0.55621077779824
F	-2.61338310050577	-1.00993052610250	1.49966222472485
F	-0.36859873472153	3.00840715247440	-0.75447786810592
H	-1.24037881266714	2.61597036217280	-0.89095730531964
F	-2.49944988717866	-1.77439338496777	-2.42127756555738
H	-2.65218832054100	-1.10897661718122	-1.65955293542420
F	1.31828093416315	-2.77265742716608	-1.27171711299276
H	0.43653820331805	-3.03032367915793	-1.55237026067647
F	-0.91982206474895	-3.47589106376872	-1.97841808415843
H	-1.56621997254635	-2.75996504362042	-2.14436258818280
F	-0.37167472328851	-1.29816834000146	2.29756604057881
H	-1.26799844080864	-1.17979067489947	1.94921409648254

9HF·py (DFT/LC-PBE/def2-TZVPPD+CPCM(High dielectric))

C	3.63816705191273	-1.10302170933285	0.10618995406682
C	3.47321300525325	-1.25278325370274	1.46340950571020
C	2.34655551730327	-0.75339668458767	2.04491330232870
C	1.57042952187666	0.02033058869379	-0.00586054209597
C	2.67701293164699	-0.46062002435170	-0.63901713713697
H	4.52659138372486	-1.49252230317676	-0.37721555111645
H	4.21314105728977	-1.75443116009102	2.07205121884539
H	2.13519864509138	-0.82669768140631	3.10403620348351
H	0.76733877550177	0.53919940044279	-0.51309181382631
H	2.78200662910646	-0.33009045536662	-1.70748992528633
N	1.44019035263729	-0.13654675523933	1.30248480022163
F	-1.81615534169823	-0.60044529700649	-1.56142991997242
H	0.59749854098389	0.23549602076205	1.76580619156482
F	-0.96719752095160	-2.79156447932602	-1.32366097166545
H	-1.31601085842421	-1.87291881389452	-1.41712354695138
H	-2.79569491567496	-0.20521721888058	-0.80792414674152
F	-3.56586834328729	0.10122698187716	-0.22001411694685
F	-0.72981709307826	0.78578140889623	2.69440503934543
H	-1.61386850836858	0.89550221958514	2.31471257061679
F	-0.80222004048211	0.91625714055452	-3.00303938235090
H	-1.24345213819635	0.26025193801596	-2.37758506305490
F	0.52589722986548	-3.23459032166449	0.58099429233796
H	-0.05502000860404	-3.04230648804146	-0.15259144813420
F	0.44515715627427	3.74824550193421	-0.26527564079495
H	-0.04895805648842	3.51352518690051	-1.04686211090678
F	-2.96106531109785	1.09585473474076	1.82895626879922
H	-3.19015162573795	0.68113019530472	0.95912006576613
F	-0.84889469384606	3.18465486257402	-2.29017535974738
H	-0.83334489344828	2.24414671303736	-2.57011315247988

1HF·TEA (DFT/LC-PBE/def2-TZVPPD+CPCM(High dielectric))

N	0.00087570709053	-0.00031708571697	-0.29273475200725
C	-1.23284959045887	-0.63598419296925	-0.75775294621469
H	-1.13147857428289	-0.81465774975658	-1.83244542046589
H	-2.04094071694105	0.08048509199089	-0.62348048936366
C	1.16811366387965	-0.75040371438590	-0.75927328169269
H	1.26819885436594	-0.57481929273920	-1.83457862826664
H	0.95290568205312	-1.80849091517226	-0.62253893834489
C	0.06826173308629	1.38710850105673	-0.75394057621242
H	1.09075923084673	1.72994364963405	-0.60718738621911
H	-0.12529480331012	1.39057194835262	-1.83081704722040
C	-0.87167583980717	2.30374461197281	-0.02718418565556
H	-1.91835922811898	2.06402372777929	-0.20974549843826
H	-0.69114161751065	2.26744221356792	1.04663339231768
H	-0.70674486439984	3.32516388496473	-0.36814439803046
C	-1.56400250047839	-1.90114252693199	-0.02195012295514
H	-1.63909118381612	-1.71481069052797	1.04890142344687
H	-2.52472452190866	-2.27593956747637	-0.37313305076103
H	-0.82792314255781	-2.68726061477175	-0.18463681178095
C	2.43207983058861	-0.40302671638882	-0.02911545941449
H	2.74501836952358	0.62664333301302	-0.19655760658443
H	2.31263878224871	-0.55825882958495	1.04263031875070
H	3.23508102257946	-1.04946829211981	-0.38130439589056
F	0.00056436747139	0.00134292483673	2.12273904382906
H	0.00072159025655	-0.00103869982695	0.89414527337421

2HF·TEA (DFT/LC-PBE/def2-TZVPPD+CPCM(High dielectric))

N	0.60654479517997	0.19259160173634	-0.06686520541623
C	0.35425941974218	-0.61286157960324	1.13537062525327
H	-0.55333449634389	-0.21782753989202	1.58729160069633
H	1.17459761445534	-0.43831010286198	1.83284300635293
C	0.76477577956286	1.61806732341641	0.25621797870330
H	1.03599040022055	2.11454510435966	-0.67549780684101
H	1.60238582585071	1.71530579654678	0.94685690098890
C	1.72831030239448	-0.30448508044003	-0.87910442384235
H	1.43828100148860	-1.28382225542556	-1.25655205355095
H	1.80070521833714	0.36411408524329	-1.73668409002532
C	3.03236838157459	-0.37957081567357	-0.14516723070274
H	2.99336794039218	-1.08163903417222	0.68733520319006
H	3.79681144268418	-0.73159575256819	-0.83635817334162
H	3.35063757284395	0.59160011792170	0.23138664843108
C	0.18056211204235	-2.06976455503331	0.83113358193161
H	1.10986187764736	-2.54299824014385	0.51656062294635
H	-0.16273666694634	-2.57798845215741	1.73089871191109
H	-0.56908673299172	-2.22031409195837	0.05326218480462
C	-0.48129217527390	2.22744126018522	0.82601815931268
H	-0.69431144207701	1.87759314664257	1.83448007898895
H	-0.35036239155793	3.30747143693602	0.87455135510541
H	-1.34362490558802	2.02015802129739	0.19148111639522
F	-1.45249952548486	-0.03707839829721	-1.56793245431526
H	-0.25933438427830	0.10583280151276	-0.68491407663942
F	-3.45816824962842	-0.38918723646000	-0.52789385646096
H	-2.54822779094605	-0.22865714341118	-0.99937805187594

3HF·TEA (DFT/LC-PBE/def2-TZVPPD+CPCM(High dielectric))

N	0.61066127403496	0.39148344424542	0.27193972453363
C	0.61588114018741	0.07386772169363	1.70970206146516
H	-0.25864962820512	0.57374613741011	2.12603509070197
H	1.49957908740017	0.53882135775632	2.14669909568082
C	0.60943091798694	1.85327355690400	0.09428682930576
H	1.49415580378294	2.24711742272486	0.59428155532469
H	-0.26343548864986	2.21661816328262	0.63664875603397
C	1.66948406638564	-0.29353902405604	-0.48762824385672
H	1.50020156790009	-1.36176433676998	-0.36483552216229
H	1.49771964242444	-0.06501094945706	-1.53782726533852
C	3.05499274245542	0.08801254304120	-0.06870174972810
H	3.76469735783241	-0.46452902321221	-0.68251822489907
H	3.25031095841799	1.15022031501630	-0.21444327094129
H	3.25315340409466	-0.16471588057046	0.97268148361875
C	0.56622532729478	-1.39627458095374	1.98932212191327
H	0.38908539106449	-1.54227755639912	3.05377328881756
H	-0.24863552697267	-1.87490296597109	1.44454515816051
H	1.49947993073977	-1.89835541423570	1.73787874515613
C	0.55075971780030	2.27256890665395	-1.34167607843636
H	-0.26700659734397	1.77587366047060	-1.86548659056171
H	0.37250700200109	3.34599100174363	-1.38357990954162
H	1.48088856352895	2.07087449812832	-1.87141140478695
F	-1.70610507376296	-0.50960864546960	-0.64114393062338
H	-0.29907829641344	0.03836198129709	-0.11000523921955
F	-1.72369599336086	-2.59521565338200	-1.71698067572614
F	-3.70453181731871	0.39134022701340	0.19525819773390
H	-1.72376932643464	-1.71808252308141	-1.25765181330617
H	-2.86404366178160	0.00274159366171	-0.15561390561912

4HF·TEA (DFT/LC-PBE/def2-TZVPPD+CPCM(High dielectric))

N	-0.99521124172710	0.42524920526794	-0.23005919579480
C	-2.16794174724894	0.41139348918339	0.66486293374888
H	-1.79093585454358	0.51116470477188	1.68127183080763
H	-2.60304830679857	-0.58347602830172	0.57464496679855
C	-1.35674784479867	0.03576365335996	-1.60518477742425
H	-1.93329283464351	0.85276892986733	-2.03750712635137
H	-2.01028379810072	-0.83177084794812	-1.51546853087304
C	-0.25471392025037	1.69824315029770	-0.20363486643902
H	0.63235659947398	1.55132942019803	-0.81589069047157
H	-0.87745630858331	2.44640812282554	-0.69429824404302
C	0.13562855222178	2.11625571981647	1.18073322323066
H	0.65955114600572	1.31381593130117	1.70204492694338
H	0.81278140336921	2.96593962610740	1.10967697856191
H	-0.72068782339917	2.42261846494595	1.77996099032986
C	-3.17900966955046	1.47240944256013	0.35866763242890
H	-3.58091276446770	1.38074979286228	-0.64934378359423
H	-4.00890754498993	1.36282377811308	1.05518878174232
H	-2.77397644168934	2.47586527309649	0.48544450205190
C	-0.16118326343781	-0.29542987934835	-2.44648705466953
H	0.46889854636518	-1.04026278032373	-1.95875728038611
H	-0.50729479734628	-0.71606761087914	-3.38939317772137
H	0.44342601741707	0.57915449454545	-2.67960776916767
F	0.60273617869830	-1.53047090404636	0.76779331563144
H	-0.35596066259475	-0.31012141272497	0.13470410554250
F	0.30223076346735	-3.72201133271907	-0.12184869654782
F	2.67882119194527	-0.93024288514337	1.46874420973931
H	0.43480451048215	-2.82739597133153	0.24167608961246
H	1.71028701680214	-1.21297012110439	1.13880373000977
F	3.67378651303773	0.95229000652695	0.36171663478579
H	3.26098407537182	0.19375584510866	0.80243803831369

5HF·TEA (DFT/LC-PBE/def2-TZVPPD+CPCM(High dielectric))

N	-0.37139206136773	1.03123919063174	-0.01946718139135
C	0.85590129657821	1.84555605020617	-0.00789191061103
H	1.58937619935492	1.28985218935300	0.57267035421069
H	0.62834198624273	2.76613463355303	0.52981518226696
C	-0.87164170588041	0.77892219955570	1.34445249702534
H	-1.81471573079188	0.24508487778209	1.22836441815849
H	-1.07837744883297	1.74589846523273	1.80124821355750
C	-1.43133483445014	1.57397290901567	-0.89151684181331
H	-1.05668457220335	1.52633991928688	-1.91256818638974
H	-2.26659926962902	0.87806836224224	-0.81715301338377
C	-1.85367063992125	2.96607676426378	-0.53791303005054
H	-2.24664724071540	3.03441025934216	0.47534931012623
H	-1.04014761935111	3.68234173007728	-0.64850074756749
H	-2.64880797290730	3.26383297175427	-1.21982500468178
C	1.38750174207201	2.11372338960424	-1.38169100397037
H	2.36776169493890	2.57893570364139	-1.29070186971151
H	1.50844433965411	1.18672924124045	-1.94426949586094
H	0.75065035557556	2.78861277199722	-1.95189579645743
C	0.08442163696706	-0.02383103399509	2.17402532241020
H	0.39351462461132	-0.93192176609391	1.65424165278328
H	0.97226445853753	0.53997695967080	2.45395031171003
H	-0.42046073196407	-0.32072580660805	3.09207091059311
F	0.18955781279672	-1.48242770201635	-1.02394670851503
H	-0.12371070481004	0.10757145426591	-0.41971186463176
F	2.42520135327675	-1.92522856287240	-0.73213474358638
F	-3.17840018061252	-1.93338362782655	1.01940704476936
H	1.42281673538716	-1.73933907867181	-0.86074838975435
H	-2.45550059159022	-2.33628277873739	0.52578368284699
F	-1.36899757816291	-2.96608237512697	-0.21790781928811
H	-0.66388508152186	-2.31013645779355	-0.57675758750397
F	3.59423949608271	-0.33404438202615	0.67212079550519

H	3.11999149227279	-0.96352145245191	0.11767995542342

1HF·py (DFT/LC-PBE/def2-TZVPPD+CPCM(Low dielectric))

C	-2.13336721768510	-0.00007435078054	-0.00005727941051
C	-1.43859555852271	1.18517755140069	-0.00003073986112
C	-0.06947547540578	1.14083551726468	0.00001938802842
C	-0.06936893649996	-1.14079439966965	0.00001279427156
C	-1.43848802358756	-1.18526263787904	-0.00004378166232
H	-3.21724177536412	-0.00012526157294	-0.00009007498741
H	-1.94868385055580	2.13964875576044	-0.00004188250503
H	0.52635902947918	2.04731954497449	0.00004121982283
H	0.52655190109338	-2.04722311214926	0.00003198513686
H	-1.94848885997987	-2.13978066540636	-0.00006542473229
N	0.59386254965214	0.00005132154909	0.00004106507027
F	3.02556161040532	0.00007363165600	-0.00003942514790
H	1.98563798657088	0.00007902125242	0.00006227157665

2HF·py (DFT/LC-PBE/def2-TZVPPD+CPCM(Low dielectric))

C	-2.79276295371410	0.46848415252135	-0.00529395627041
C	-1.79704376425228	1.41657836792305	0.00526302084114
C	-0.49709126624900	0.99946075877799	0.01042292119924
C	-1.14729143404689	-1.21812425202124	-0.00473032366007
C	-2.46658187502305	-0.86730614981533	-0.01037439892523
H	-3.83277062823301	0.77349861237220	-0.00960777499793
H	-2.02307190903937	2.47434006186555	0.00944263317646
H	0.34364972566446	1.68251882101796	0.01859620941169
H	-0.80936065506358	-2.24713491023621	-0.00812865458620
H	-3.22793189408505	-1.63553864790115	-0.01864616537133
N	-0.20619144122438	-0.29001240365275	0.00536143122296
F	2.15900186378560	-0.97961267647390	0.01412863549935
H	0.85050080528221	-0.59604148074793	0.00942770758116
F	3.74883357222308	0.69456736562024	-0.01621697804900
H	3.05225923877535	-0.04315873664983	-0.00294976267183

3HF·py (DFT/LC-PBE/def2-TZVPPD+CPCM(Low dielectric))

C	-3.17801970830428	0.00038055843231	0.00308985608200
C	-2.49032670837857	1.19123082549804	0.00247836021254
C	-1.12689004316665	1.15925428024502	-0.00193942308896
C	-1.12765402627024	-1.15984218111371	-0.00510840657565
C	-2.49111685536578	-1.19092162589065	-0.00077876701140
H	-4.26180476248977	0.00074105315032	0.00669352924911
H	-3.00502329569796	2.14245896339277	0.00548727809033
H	-0.50857147948288	2.04825400728641	-0.00265060512027
H	-0.50994417556352	-2.04925837214987	-0.00823998294832
H	-3.00644826245563	-2.14181055510435	-0.00037745954110
N	-0.49012407173685	-0.00050149662605	-0.00558803729681
F	2.07588651300685	-0.00050858167277	-0.01115067716506
H	0.56836801101892	-0.00078112863700	-0.00848622916209
F	3.19052395498723	2.07951373469707	0.01160654408796
H	2.73765987660848	1.20647839315709	0.00227954184723
H	2.73990862308683	-1.20632720585695	0.00374708154104
F	3.19475491820385	-2.07839730430766	0.01413189619944

4HF·py (DFT/LC-PBE/def2-TZVPPD+CPCM(Low dielectric))

C	-3.29682971713645	0.52810719690395	0.17401065239276
C	-2.64163822845573	-0.67045025725634	0.33097085647425
C	-1.27823144382925	-0.68514388588183	0.28383304285977
C	-1.21976864601055	1.61246014224347	-0.06420994693311
C	-2.58023630552993	1.68512109488185	-0.02719744618811
H	-4.37957171714883	0.56227815861161	0.20779946590470
H	-3.18154808014251	-1.59425359452174	0.48959289198351
H	-0.68075303858379	-1.58416204961622	0.39579099357629
H	-0.57884544272556	2.47162111853798	-0.21688485190289
H	-3.07038919602619	2.64054712336627	-0.15437065304221
N	-0.61543926642432	0.44479982508192	0.09064227878320
F	2.01702521505860	0.35779090379897	0.04914627326424
H	0.42862603396549	0.41505799214909	0.06160489254799
F	2.70589885945431	-1.81067892630731	0.13080395666679
H	2.40915652385919	-0.80016104296075	0.08675867318685
H	2.79572729458951	1.32581821438833	-0.64999867300922
F	3.31380075940873	1.99795321024209	-1.12041252517436
F	0.77769973234156	-3.18864628241416	0.49307909885424
H	1.57397151463831	-2.65158991388954	0.34502997929564

5HF·py (DFT/LC-PBE/def2-TZVPPD+CPCM(Low dielectric))

C	-3.57675654436373	0.02363573954276	0.04627187579293
C	-2.91176535812306	0.01640199050271	1.25020955880204
C	-1.54864261194930	0.00632944576068	1.24485224734817
C	-1.50647380862246	0.01037523420185	-1.07777294364167
C	-2.86851184712451	0.02051646235012	-1.13267133889664
H	-4.66030726173464	0.03192189923038	0.02644782902637
H	-3.44442106540792	0.01867649568056	2.19145750179898
H	-0.94637091534582	0.00030797471809	2.14445486817163
H	-0.87177030036648	0.00745853362489	-1.95482239922452
H	-3.36658549821707	0.02607788451524	-2.09264608452978
N	-0.89259020879251	0.00363196690089	0.09499910605762
F	1.75361073930602	-0.01177603047942	0.12351116642340
H	0.14905319230660	-0.00328858351974	0.11237602970501
F	2.87642129792895	-2.02657616403249	0.06154339352812
H	2.39163445926630	-1.13186779452339	0.08937317164185
H	2.02422514509110	3.14138857168339	-0.20397710407263
F	1.45183743355507	3.89175410336215	-0.38530114298415
F	1.40667123380388	-3.91161226888790	-0.39590895146366
H	1.98745816188792	-3.16769314884033	-0.21475565014914
F	2.89995438496169	1.99011265970121	0.07263040352544
H	2.40509881670783	1.10087740453291	0.09550838579020

6HF·py (DFT/LC-PBE/def2-TZVPPD+CPCM(Low dielectric))

C	-3.61867442792682	0.09467342518547	-0.09964561523385
C	-2.99881216374437	-0.38638100614791	1.02992267376954
C	-1.70780583760853	-0.02403780764641	1.27371800158941
C	-1.65121270829179	1.26026501818749	-0.66652631286933
C	-2.93986005960426	0.92699143464870	-0.95884275850117
H	-4.64424426787115	-0.18340559822869	-0.31332275224284
H	-3.50992139594998	-1.04325875573120	1.72038340798120
H	-1.13478756675824	-0.35570323970481	2.12910149056363
H	-1.03636967414113	1.90661078980022	-1.27855539591398
H	-3.40438222446223	1.31853921020653	-1.85360036233069
N	-1.08306671114907	0.78021175119606	0.42847900444136
F	2.76558434623596	-0.82993854395280	0.43203640539882
H	-0.11195505297257	1.04106444564472	0.62579220982920
F	1.75836804712504	-2.59058522166235	-0.71710769200556
H	2.20559866466480	-1.84026449483250	-0.22271663166724
H	1.85485912942854	-0.25346267201594	1.38031132160461
F	1.19792724156908	0.16585631090868	1.95928144829088
F	-0.58261841988886	-2.10657629658705	-1.24041565317390
H	0.33451727124944	-2.29726923185191	-1.03684419709183
F	3.11746053944096	1.04391537381483	-0.88361867654043
H	3.01664949039222	0.20137004110607	-0.32928337337197
F	1.13196552205584	2.29895179101603	-0.32450055939147
H	1.94525023658196	1.83553201373494	-0.57169195704413

7HF·py (DFT/LC-PBE/def2-TZVPPD+CPCM(Low dielectric))

C	-3.83749946249172	0.29705358792293	0.01843332884851
C	-3.23692460009302	-0.02341531762956	-1.17643410247800
C	-2.00978837764407	-0.61591490267076	-1.15527559313224
C	-1.97590166087502	-0.57304106950313	1.17068700360351
C	-3.20193806468386	0.02075811420764	1.20639459865478
H	-4.81315302104032	0.76905404219726	0.02373083346777
H	-3.71483283200178	0.18505944369323	-2.12383491442096
H	-1.45902839653546	-0.90287224865233	-2.04089708233757
H	-1.39974756949985	-0.82910373519326	2.04962008942500
H	-3.65172475622563	0.26414439844850	2.15923335409856
N	-1.42572979720362	-0.87204968026514	0.00443036043032
F	2.91498000580804	0.86172905932119	0.01584279643041
H	-0.50271463228015	-1.31893884579517	0.00083490833778
F	2.58648989959685	-0.48441730759698	1.89493728063865
H	2.74397812521063	0.10729363292287	1.10147798935800
H	2.19073603803257	2.00685741648110	0.00309187345829
F	1.64004659766650	2.83419535839167	-0.00564826716830
F	0.66867309836171	-1.91878433868875	1.51853128755931
H	1.43943716930429	-1.35906291574813	1.67573507141090
F	2.63093580991565	-0.47756114883782	-1.87632175237469
H	2.77135060978733	0.11018169117189	-1.07719414988149
F	-0.77014093425233	2.36659335525848	-0.10367056598963
H	0.16796156338876	2.54928136090654	-0.06571596322521
F	0.66376245198466	-1.86028503303770	-1.56197155239107
H	1.45192384801274	-1.31946818905392	-1.69655188336966

8HF·py (DFT/LC-PBE/def2-TZVPPD+CPCM(Low dielectric))

C	3.14138330255266	1.96065202785459	0.04689855349812
C	2.44693501232440	0.97556677969729	-0.61520881509707
C	1.47748634753775	0.29280444863896	0.05687013469233
C	1.87463355953112	1.52859371600975	1.98533441475979
C	2.85350249545373	2.24266555284323	1.36184752279032
H	3.91588856975370	2.51658615498352	-0.46874635976775
H	2.65173898272850	0.73582607695405	-1.64970523739968
H	0.88203546304552	-0.49414678820590	-0.38906631313417
H	1.58281018269249	1.68481505164100	3.01591930538209
H	3.38261360810271	3.01387900715910	1.90475743894948
N	1.22509723835370	0.58364599829858	1.32367075413454
F	-2.41163223147094	-0.21692224849964	-0.84627263244458
H	0.48295476113508	0.05713363758865	1.81277533138319
F	-2.32350821900719	2.13378292770868	-1.04871120054200
H	-2.36772351002369	1.14941156920070	-0.97072189123726
H	-2.61632857183669	-0.65576209872924	0.39330937112123
F	-2.74773940161371	-0.98035338689048	1.33345763205971
F	-0.43487208544220	3.13304869050464	0.17123500001069
H	-1.16789767954231	2.74461666183672	-0.30278791363905
F	-0.76971452309140	-1.33269440081842	-2.03962918660132
H	-1.50162609651165	-0.85665778581727	-1.52223002389259
F	1.30668030319763	-4.85729530790962	-1.92901896320202
H	0.47024797097660	-4.40776993652591	-2.00734322481914
F	-0.87607091525371	-3.70994073514392	-2.13595366998056
H	-0.84624644891491	-2.73204998446193	-2.08953005816546
F	-0.73452728309144	-0.78361098933371	2.62468238178630
H	-1.55665918918460	-0.86895872545337	2.11043476246439

9HF·py (DFT/LC-PBE/def2-TZVPPD+CPCM(Low dielectric))

C	3.56314248664182	-1.15224213537078	0.12838540789504
C	3.39930547243660	-1.05293622569659	1.49045976879837
C	2.29516603500523	-0.41615275655278	1.97151882559281
C	1.53889503415770	0.01147386230822	-0.18453719742257
C	2.62316398527952	-0.61676627908266	-0.72039784844864
H	4.43367213483442	-1.65513710369631	-0.27655564525371
H	4.12218044763050	-1.46753040948374	2.17964252262834
H	2.08579288438748	-0.29327261495470	3.02638735799664
H	0.75388241120049	0.46143452193663	-0.77748981771624
H	2.72713439798176	-0.68409162387470	-1.79469852333074
N	1.40948050924649	0.09460162282272	1.12990530277418
F	-1.91868839271885	-0.67550181889608	-1.61699877584822
H	0.58145178771412	0.57226008357368	1.51866414060633
F	-0.59942875146752	-2.63198565613561	-1.47475588443427
H	-1.15072482676873	-1.81644286090155	-1.53949760372413
H	-2.87191324249509	-0.50519571982067	-0.74951305883215
F	-3.61654882047449	-0.37839049190079	-0.07147925384365
F	-0.72791620526183	1.33644574152566	2.28060798496865
H	-1.63932425525373	1.04140549661814	2.13362240901235
F	-1.24564799603149	1.11018332834432	-2.94557050186074
H	-1.54284734867192	0.33748213032464	-2.37088975559793
F	0.41322788887964	-2.99400598777554	0.74431958261318
H	0.02243408664076	-2.84924442669106	-0.11427789507026
F	1.13111607500637	3.12309278456951	-0.21127008947511
H	0.40920979908040	3.12572463305857	-0.83477909119179
F	-3.01257618897739	0.63549216518953	1.96745721746021
H	-3.24536056549145	0.20735374027861	1.10510541750257
F	-0.74594547993553	3.14211208208434	-1.81457354488468
H	-0.94867989347903	2.29640832182072	-2.26805655420745

1HF·TEA (DFT/LC-PBE/def2-TZVPPD+CPCM(Low dielectric))

N	-0.00023903995881	0.00041844321281	-0.19390194803294
C	-1.27391917980179	-0.55581501494152	-0.64101378723108
H	-1.30667689941540	-0.50641429245340	-1.73484071176750
H	-2.05733492932346	0.09619163472773	-0.26013412055055
C	1.11820892695564	-0.82511059600564	-0.64034262550831
H	1.09391588892476	-0.87634820152283	-1.73431526052772
H	0.94316892617029	-1.83000882376607	-0.26157235409488
C	0.15451333345177	1.38110298939762	-0.64260926091714
H	1.11272504852438	1.73339197451796	-0.26612424201304
H	0.20928300497229	1.38412899753916	-1.73669397389546
C	-0.92886354332192	2.29821968373231	-0.15258741731705
H	-1.87993261590840	2.13546777937874	-0.65649711613825
H	-1.07797645948609	2.17982161328947	0.92137598682931
H	-0.63178627765547	3.32947740664056	-0.33936385995987
C	-1.52472489403493	-1.95416412025131	-0.15473885370912
H	-1.34345823792379	-2.02815776701032	0.91832101844689
H	-2.56730464755307	-2.21134488347095	-0.33791696825701
H	-0.91070778611024	-2.69489966892795	-0.66401453321602
C	2.45384939729281	-0.34520572986343	-0.14979906995546
H	2.79014743011374	0.55768244227502	-0.65640183925834
H	2.42353809715416	-0.15331098339587	0.92341378688309
H	3.19796292065243	-1.11950895821718	-0.33247243162248
F	0.00560444469007	0.00341216994642	2.20840162771984
H	0.00099934199101	0.00182490396866	1.03235641029309

2HF·TEA (DFT/LC-PBE/def2-TZVPPD+CPCM(Low dielectric))

N	0.60454860337167	0.19674538341171	-0.08199690688787
C	0.33131047490503	-0.61249269915797	1.11231091622292
H	-0.58303465292906	-0.21740774319390	1.55051717441469
H	1.14053214342872	-0.44436283900067	1.82448789717284
C	0.75961913968771	1.62045980385327	0.24692734514425
H	1.03549418892433	2.12007490991806	-0.68185882909362
H	1.59337067714388	1.71781074623573	0.94258286259676
C	1.73596855816093	-0.29992953394639	-0.87926600700111
H	1.44748596319454	-1.27553224223585	-1.26754224672513
H	1.82414110658393	0.37267430827325	-1.73227200478500
C	3.02897150272969	-0.38565038118701	-0.12673145403036
H	2.97462912395192	-1.09320943156467	0.70025588247492
H	3.80292762666914	-0.73580055837172	-0.80823107329453
H	3.34583727029363	0.58141656872814	0.26154132768780
C	0.15634867536385	-2.06732105246870	0.79828123705615
H	1.08814977399274	-2.54364852919523	0.49587809024283
H	-0.20397319006372	-2.57882652833019	1.68946490963887
H	-0.58207497609433	-2.20951505680256	0.00826384674413
C	-0.49049168042104	2.22763247286482	0.81096925860347
H	-0.70518142394813	1.88065378804615	1.82009510879525
H	-0.36473646710165	3.30842727922186	0.85591041762818
H	-1.35020081288029	2.01399838474158	0.17497448408251
F	-1.43127871088347	-0.02284276949888	-1.58208055933130
H	-0.25778489874943	0.11246357447896	-0.71561526242471
F	-3.32457411901265	-0.41041902026247	-0.34714115640357
H	-2.47952297301795	-0.23677841685734	-0.91038490652837

3HF·TEA (DFT/LC-PBE/def2-TZVPPD+CPCM(Low dielectric))

N	0.60176376400337	0.32562586632734	0.26248393130241
C	0.62253863151464	-0.07082646365546	1.67978919736371
H	-0.28328222829965	0.34900779590258	2.11757621026491
H	1.47342724447992	0.42270655757497	2.14976445489757
C	0.50289074060403	1.79136865343688	0.16565441992316
H	1.35486415862274	2.21723127493473	0.69576971180590
H	-0.39737466162723	2.06355296505576	0.71662117066815
C	1.70255389671280	-0.24675351881447	-0.52839218560815
H	1.60214653041953	-1.32874973143934	-0.46619373862094
H	1.51752889846409	0.02711861484075	-1.56539295271604
C	3.06024439578153	0.19942931755042	-0.08292290163936
H	3.80551979617883	-0.26609528208801	-0.72599310082391
H	3.18392891706511	1.27915819893997	-0.16350756585792
H	3.27544927153026	-0.10170277422376	0.94207006686979
C	0.66279545836301	-1.55481459439836	1.87617159321942
H	0.49586563568810	-1.77208593845464	2.93002719006448
H	-0.12214388930849	-2.05090392223036	1.30354253151058
H	1.62459191225961	-1.98432376039316	1.59885498759095
C	0.42500563814339	2.28499345052320	-1.24566250982601
H	-0.35533149385583	1.76580917215414	-1.80364091398545
H	0.17605869900925	3.34493230215078	-1.23095696980704
H	1.36985496195115	2.17459403883796	-1.77618828339537
F	-1.67114763883722	-0.56201781542631	-0.69684677440513
H	-0.28804571106138	-0.05899569376460	-0.14389241860258
F	-2.14875669151853	-2.86545618705175	-0.64433273054066
F	-3.31647011879002	1.11814865010343	-0.76774645149491
H	-1.96152066360097	-1.89707882289912	-0.67007619055245
H	-2.63755575177138	0.40208899198112	-0.74246995299182

4HF·TEA (DFT/LC-PBE/def2-TZVPPD+CPCM(Low dielectric))

N	-0.94743749798226	0.40714383743277	-0.16114925264695
C	-2.19122457040126	0.40909020288979	0.63109221293153
H	-1.89940551191966	0.49166967256827	1.67667079864001
H	-2.63735513895574	-0.57586909766928	0.49563529977170
C	-1.19504869288913	0.02299277501249	-1.56204188271832
H	-1.73977642188037	0.83909077265208	-2.03572196590974
H	-1.84879362244923	-0.84848238449085	-1.52917993123979
C	-0.19159789104147	1.66786577066744	-0.06998028470703
H	0.74654595375634	1.50478139054889	-0.59629021574866
H	-0.75640371120977	2.42611987468617	-0.61279665236124
C	0.08397499309800	2.07793433903792	1.34398817700256
H	0.55209404422592	1.26731125554146	1.90440258768797
H	0.77688645220194	2.91776141825978	1.33375814689431
H	-0.81508779488400	2.39459390478849	1.87103980066273
C	-3.15237317647799	1.49361272046613	0.25365610382241
H	-3.47228327810480	1.42032375606343	-0.78474756264917
H	-4.03937251878823	1.39561906596624	0.87774858171137
H	-2.73881320445994	2.48698286264089	0.42444780410668
C	0.06595280738557	-0.29753571081267	-2.30666575809644
H	0.66399978978816	-1.03479031245942	-1.76956265383170
H	-0.20021662390226	-0.72407985057311	-3.27257447474472
H	0.67855035546901	0.58249993475902	-2.49306689660219
F	0.55831457972727	-1.54199992456853	0.94010267568553
H	-0.34956614758130	-0.33785932203645	0.25578967984378
F	-0.01592676082758	-3.69612458608435	0.08344687178618
F	2.73059852179796	-0.87178722004586	1.03419868023814
H	0.23455323401568	-2.82656515278726	0.44154708990812
H	1.72769193491222	-1.19929838937231	0.99252177273243
F	3.16279911075600	1.03110605253425	-0.36964170693860
H	2.99332969925347	0.26262833017481	0.19624065616400

5HF·TEA (DFT/LC-PBE/def2-TZVPPD+CPCM(Low dielectric))

N	-0.33188517568673	0.96654951522933	0.03688737951389
C	0.84738439366025	1.84610749698320	-0.02759231677644
H	1.63256253665929	1.35240721652562	0.54135149706922
H	0.58899946497142	2.77240439299358	0.48599085102791
C	-0.76758879004304	0.73466598092587	1.42636994139654
H	-1.68837800295872	0.15481429230519	1.36105092557832
H	-1.00328052211677	1.70508057056552	1.86159153624790
C	-1.45386608278224	1.41851533364499	-0.80957272138829
H	-1.11901834000526	1.34796290731829	-1.84314569928531
H	-2.24704352173466	0.68380176152216	-0.67058674246082
C	-1.93248129610304	2.80140632835395	-0.49285748684494
H	-2.28220817130405	2.89286373415152	0.53437449518264
H	-1.16430692610183	3.55329276651113	-0.67099389682107
H	-2.77290965357917	3.02879597475681	-1.14697818115967
C	1.31010508678363	2.09205917765519	-1.43034991774386
H	2.26717071709474	2.61002807249931	-1.39561303801572
H	1.45864023667356	1.15323593950479	-1.96587879901803
H	0.61640148470184	2.71180049805002	-1.99688817611948
C	0.25666551451787	0.00462311527950	2.24156616522037
H	0.58445470318625	-0.90718620932553	1.74030833722288
H	1.12954848228329	0.61504602398743	2.46483033952132
H	-0.19620607458732	-0.28165762952382	3.18960116590846
F	0.26745120548218	-1.53122630355799	-0.95196641632563
H	-0.04961866428690	0.04404087787478	-0.34460555851780
F	2.42916159596665	-2.10151782535598	-0.41037895448723
F	-3.35024809957619	-1.38864314142163	0.36059914440065
H	1.46188322731106	-1.86854103609700	-0.65322905178245
H	-2.63602959751851	-1.97303402362023	0.08052515014942
F	-1.53072128397480	-2.82694402700097	-0.34089399535920
H	-0.71385611832213	-2.27141038837478	-0.61802325772823
F	3.62300328958401	-0.22408869815867	0.56074016772932

H	3.15334916724295	-0.97136716344706	0.17564066040864

5.2 Development of Parameters for CPCM Modelling

A sufficient set of parameters for implicit solvent modelling could not be found in the literature at the time of writing for describing these systems. To do this, the static dielectric constant (ε_{static}) and dynamic dielectric constant ($\varepsilon_{dynamic}$) need to be defined. The calculation of these two properties depends on the dielectric constant and the refractive indices, respectively.

To calculate the static dielectric constant for these mixtures at a given temperature ($\varepsilon_{mix,\ static,\ T}$), **Equation S6** was used:

$$\varepsilon_{static,\ mix,T} = \phi_1 \ln \varepsilon_{1,T} + \phi_2 \ln \varepsilon_{2,T} + \phi_3 \ln \varepsilon_{3,T} - \frac{189.3\phi_1\phi_2}{T} + \frac{1728.1\phi_1\phi_2(\phi_1-\phi_2)}{T} - \frac{1519.8\phi_1\phi_2(\phi_1-\phi_2)^2}{T} + \frac{2074.5\phi_1\phi_3(\phi_1-\phi_3)}{T} - \frac{2571.8\phi_1\phi_3(\phi_1-\phi_3)^2}{T} - \frac{1075.6\phi_2\phi_3}{T} - \frac{311.4\phi_2\phi_3(\phi_2-\phi_3)}{T} + \frac{5552.7\phi_2\phi_3(\phi_2-\phi_3)^2}{T} + \frac{799.3\phi_1\phi_2\phi_3}{T} - \frac{8220.2\phi_1\phi_2\phi_3(\phi_1-\phi_2-\phi_3)}{T} + \frac{6447.0\phi_1\phi_2\phi_3(\phi_1-\phi_2-\phi_3)^2}{T}$$

Equation S6

Where Φ_i represents the mole fraction of component *i* in the mixture and ε_i represents the static dielectric constant for component *i*.[41] To determine $\varepsilon_{dynamic}$, the refractive index of each individual component can be used. The derivation is provided below. The dynamic dielectric constant can be expressed as the ratio of the permittivity of the solution and the permittivity of a vacuum, as shown in **Equation S7**.

$$\varepsilon_{dynamic} = \frac{E_m}{E_0}$$

Equation S7

The refractive index of a material is of a similar nature, as it is defined as the ratio of the speed of light going through a vacuum (c_0) compared to that of a material ($c_{material}$), as shown in **Equation S8**. Through Maxwell equations, the permittivity of a vacuum can be related to the speed of light in a vacuum and the vacuum permeability, according to **Equation S9**.

$$n_{material} = \frac{c_0}{c_{material}}$$

Equation S8

$$c_0 = \frac{1}{\sqrt{\mu_o E_0}}$$

Equation S9

By analogy therefore, the speed of light in a material can be related to the permittivity and permeability of the material (**Equation S10**).

$$c_{material} = \frac{1}{\sqrt{\mu_m E_m}}$$ **Equation S10**

Through combining **Equations S8 – S10**, an expression can be derived that relates the refractive index of a material to the permittivity and permeability of the material, as shown in **Equation S11**.

$$n_{material} = \left(\frac{\mu_0 E_0}{\mu_m E_m}\right)^{-\frac{1}{2}}$$ **Equation S11**

Assuming that the material is non-magnetic, **Equation S11** can be simplified further as $\mu_m = \mu_0$. Combining **Equation S7** and **Equation S11** suggests that the dynamic dielectric constant of a material is therefore the square of the refractive index of the material, as shown in **Equation S12**.

$$\varepsilon_{dynamic} = n_{mix,\,T}^{2}$$ **Equation S12**

To calculate the refractive index of the HF mixture, the refractive indices of the individual components ($n_{i,\,T}$) have been used, along with the volume fraction ($\vartheta_{i,\,T}$) that they occupy in the mixture (**Equation S13**).

$$\varepsilon_{static,\,mix,\,T} = n_{mix,\,T}^{2} = \left(\sum \vartheta_{i,\,T}\, n_{i,\,T}\right)^{2}$$ **Equation S13**

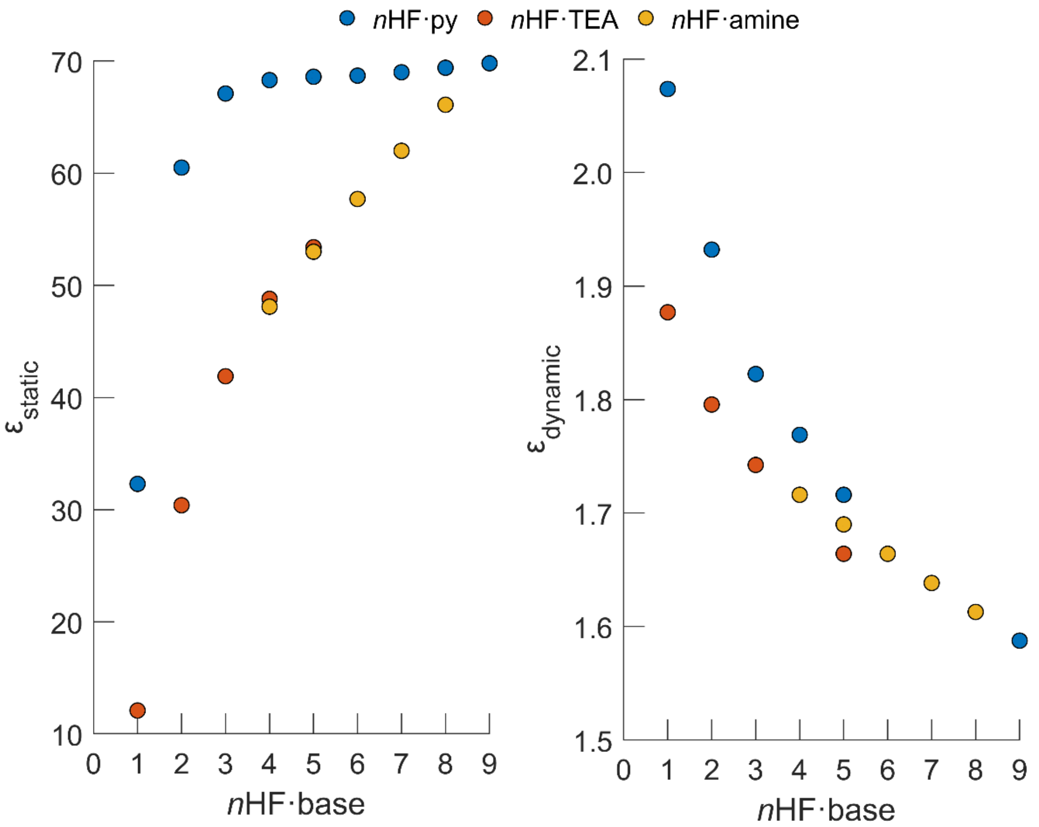

Figure S18: Plots showing how the static dielectric constant and dynamic dielectric constant vary between the different HF mixtures.

5.3 Optimisation of Anionic Clusters $[F(HF)_x]^-$ (x = 1-4)

Computational studies of anionic clusters were carried out in ORCA 6.0.[16] Initial guesses for the fluoride anionic clusters $[F(HF)_x]^-$ (x = 1 – 4) have already been described in the literature at the MP2/aug-cc-pVTZ level of theory, and the coordinates produced at this level of theory was used as an initial guess for all geometry optimisations.[42] Optimisations were then carried out at the CCSD/aug-cc-pVTZ level of theory, and implicit solvent was modelled using the conductor polarisable continuum model (CPCM)[40] framework (see section 4.2 for further details). Stationary points were characterised through their harmonic vibrational frequencies as minima, where ground states can be identified through a lack of imaginary vibrational frequencies.

Cartesian coordinates

$[F(HF)]^-$ (CCSD/aug-cc-pVTZ+CPCM(High dielectric))

F 0.00000000 0.00000000 -1.12770500

F 0.00000000 0.00000000 1.12770500

H 0.00000000 0.00000000 0.00000000

SCF Done: E(CCSD) = -200.26142 Hartree

Zero-point correction = 0.010051 Hartree

Thermal correction to energy = 0.012585 Hartree

Thermal correction to enthalpy = 0.013529 Hartree

Thermal correction to Gibbs Free energy = -0.010549 Hartree

$[F(HF)_2]^-$ (CCSD/aug-cc-pVTZ +CPCM(High dielectric))

F -2.03269300 -0.40154900 0.00000000

F -0.00005400 0.75117200 0.00000000

F 2.03275200 -0.40126500 0.00000000

H -1.16740300 0.08319300 0.00000000

H 1.16739600 0.08335500 0.00000000

SCF Done: E(CCSD) = -300.63374 Hartree

Zero-point correction = 0.023864 Hartree

Thermal correction to energy = 0.028284 Hartree

Thermal correction to enthalpy = 0.029228 Hartree

Thermal correction to Gibbs Free energy = -0.004089 Hartree

$[F(HF)_3]^-$ (CCSD/aug-cc-pVTZ+CPCM(High dielectric))

H -1.26343100 0.68227500 0.00002700

F -2.11262800 1.14090800 0.00004600

H 0.04118500 -1.43386700 -0.00001500

F 0.06933100 -2.39858400 -0.00002800

H 1.22184900 0.75195800 -0.00001800

F 2.04441600 1.25678000 -0.00002300

F -0.00072800 0.00054300 0.00000000

SCF Done: E(CCSD) = -401.00083 Hartree

Zero-point correction = 0.037076 Hartree

Thermal correction to energy = 0.043945 Hartree

Thermal correction to enthalpy = 0.044889 Hartree

Thermal correction to Gibbs Free energy = 0.002911 Hartree

$[F(HF)_4]^-$ (CCSD/aug-cc-pVTZ+CPCM(High dielectric))

H -0.75679000 -1.06389000 0.76088800

F -1.23332300 -1.73316900 1.23986600

H 0.84777000 -0.65070700 -1.06887200

F 1.38110200 -1.06006500 -1.74149400

H 0.88952200 0.76318900 0.95383800

F 1.44911600 1.24363800 1.55420300

H -0.98030700 0.95122800 -0.64595100

F -1.59730000 1.55005400 -1.05233000

F 0.00020300 -0.00026700 -0.00015500

SCF Done: E(CCSD) = -501.36308 Hartree

Zero-point correction = 0.050011 Hartree

Thermal correction to energy = 0.059384 Hartree

Thermal correction to enthalpy = 0.060328 Hartree

Thermal correction to Gibbs Free energy = 0.011776 Hartree

$[F(HF)]^-$ (CCSD/aug-cc-pVTZ+CPCM(Low dielectric))

F 0.00000000 0.00000000 -1.12829800

F 0.00000000 0.00000000 1.12829800

H 0.00000000 0.00000000 0.00000000

SCF Done: E(CCSD) = -200.25383 Hartree

Zero-point correction = 0.010097 Hartree

Thermal correction to energy = 0.012629 Hartree

Thermal correction to enthalpy = 0.013573 Hartree

Thermal correction to Gibbs Free energy = -0.010504 Hartree

$[F(HF)_2]^-$ (CCSD/aug-cc-pVTZ+CPCM(Low dielectric))

F -2.04440700 -0.39389000 0.00000000

F -0.00004700 0.73904000 0.00000000

F 2.04446200 -0.39361300 0.00000000

H -1.17357500 0.08160300 0.00000000

H 1.17356500 0.08176600 0.00000000

SCF Done: E(CCSD) = -300.627255 Hartree

Zero-point correction = 0.023936 Hartree

Thermal correction to energy = 0.028350 Hartree

Thermal correction to enthalpy = 0.029295 Hartree

Thermal correction to Gibbs Free energy = -0.003994 Hartree

$[F(HF)_3]^-$ (CCSD/aug-cc-pVTZ+CPCM(Low dielectric))

H -1.26401400 0.68260100 0.00002400

F -2.11331400 1.14128500 0.00003900

H 0.04108000 -1.43482700 -0.00001500

F 0.06900700 -2.39966600 -0.00002800

H 1.22262700 0.75253900 -0.00001100

F 2.04516400 1.25762800 -0.00002300

F -0.00055400 0.00045400 0.00000400

SCF Done: E(CCSD) = -400.99511 Hartree

Zero-point correction = 0.037186 Hartree

Thermal correction to energy = 0.044039 Hartree

Thermal correction to enthalpy = 0.044983 Hartree

Thermal correction to Gibbs Free energy = 0.003144 Hartree

$[F(HF)_4]^-$ (CCSD/aug-cc-pVTZ+CPCM(Low dielectric))

H -0.75751600 -1.06490700 0.76154900

F -1.23401200 -1.73419900 1.24046500

H 0.84854300 -0.65134600 -1.06980400

F 1.38187100 -1.06070400 -1.74236900

H 0.89034900 0.76393500 0.95467300

F 1.44994900 1.24439500 1.55496100

H -0.98120500 0.95214300 -0.64649700

F -1.59816700 1.55095500 -1.05285300

F 0.00018100 -0.00026200 -0.00013200

SCF Done: E(CCSD) = -501.35779 Hartree

Zero-point correction = 0.050137 Hartree

Thermal correction to energy = 0.059495 Hartree

Thermal correction to enthalpy = 0.060439 Hartree

Thermal correction to Gibbs Free energy = 0.011979 Hartree

5.4 Stability of Anionic Clusters $[F(HF)_x]^-$ (x = 1-4)

There are several methods that can be used to assess this property, such as stability energy (E_{stab}), HOMO-LUMO energy gaps (E_{gap}) and binding energies (E_{bind}), as shown in **Equations S14 – S16**:

$$E_{stab} = E_{[F(HF)_n]^-} - E_{F^-} - nE_{HF}$$ **Equation S14**

$$E_{gap} = E(LUMO)_{[F(HF)_n]^-} - E(HOMO)_{[F(HF)_n]^-}$$ **Equation S15**

$$E_{bind} = E_{[F(HF)_{n-1}]^-} + E_{HF} - E_{[F(HF)_n]^-}$$ **Equation S16**

For the above equations, $E_{[F(HF)_n]^-}$, $E_{[F(HF)_{n-1}]^-}$, E_{F^-}, E_{HF} are energies of $[F(HF)_n]^-$, $[F(HF)_{n-1}]^-$, F^- and HF, respectively, including thermal corrections. Plotted data associated with **Equations S14 – S16** are shown in **Figure S19**.

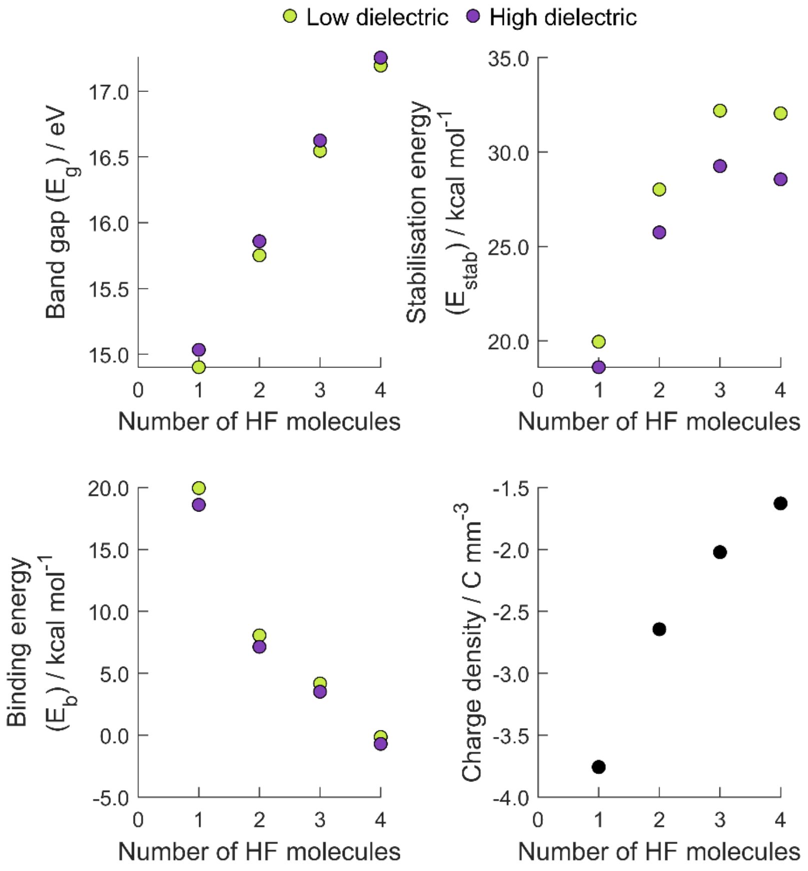

Figure S19: The band gap, stabilisation energy, binding energy, and charge density of $[F(HF)_x]^-$ anions (x = 1-4) using CCSD/aug-cc-pVTZ+CPCM(custom). High dielectric: ε_{static} = 69.8; $\varepsilon_{dynamic}$ = 1.5876. Low dielectric: ε_{static} = 12.1; $\varepsilon_{dynamic}$ = 1.8769.

Though the electronic stability increases with greater degrees of HF coordination to the fluoride anion (**Figure S19;** top left), other indicators related to stability decrease. When considering the individual components that contribute to the binding energy, the trend can be explained. The binding energy of the interaction ($E_{binding}$) will depend on two main factors: i) the electrostatic term describing the attraction between the negatively charged fluoride anionic cluster and the dipole of the HF molecule ($E_{electrostatic}$, **Equation S18**), and ii) the hydrogen bonding interaction term between the hydrogen of the HF molecule and the fluoride anion (E_{HB}, **Equation S19**).[43]

$$E_{binding} = E_{Electrostatic} + E_{HB}$$ **Equation S17**

$$E_{electrostatic} = \sum_{\substack{\alpha\varepsilon A \\ \beta\varepsilon B}} \frac{Z_\alpha Z_\beta}{R_{\alpha\beta}} - \int \sum_{\alpha\varepsilon A} \frac{Z_\alpha \rho_B(\mathbf{r})}{|\mathbf{r}-R_\alpha|} d\mathbf{r} - \int \sum_{\beta\varepsilon B} \frac{Z_\beta \rho_A(\mathbf{r})}{|\mathbf{r}-R_\beta|} d\mathbf{r} + \int \int \frac{\rho_A(\mathbf{r_1}) \rho_B(\mathbf{r_2})}{r_{12}} d\mathbf{r_1} d\mathbf{r_2}$$ **Equation S18**

$$E_{HB} = C \cos^2 \theta \, e^{-\lambda r_{\alpha\beta}}$$ **Equation S19**

In **Equation S18**, Z_α and R_α are the nuclear charge and position of atom α, respectively, in hydrogen fluoride. Z_β and R_β are the nuclear charge and position of atom β, respectively, within the fluoride anionic cluster. Along with the individual atoms that make up both species, the electronic density for both hydrogen fluoride ($\rho_A(\mathbf{r})$) and the fluoride anionic cluster ($\rho_B(\mathbf{r})$) are required.[43] The equation proposed in **Equation S19** is based on recent work that describes hydrogen bonding by considering quantum tunnelling, where C and λ are empirical constants representing the ideal hydrogen bond strength and the rate at which it decays with distance (r_{HF}).[44] The introduction of the hydrogen bonding interaction angle (θ) term is not necessary here, as the F^-…H-F angle is approximately 180°. Through considering **Equation S18** and **Equation S19**, it can be shown that as the charge density of the fluoride anionic cluster decreases (**Figure S19;** bottom right), the electrostatic contribution to the binding energy decreases. As more HF molecules are introduced, steric encumbrance begins to affect the distance between the interacting fluoride anion and the hydrogen of the HF molecule coming in to interact with the anionic cluster, resulting in larger distances between the interacting species and therefore a further decrease in binding energy.

5.5 Non-Covalent Interaction Analysis of *n*HF·py and *n*HF·TEA clusters

When considering the Non-Covalent Interactions (NCIs) of all the *n*HF·base clusters, both reduced density gradient (RDG) and Quantum Theory of Atoms In Molecules (QTAIM) were performed in MultiWFN 3.8.[45] Structures that were obtained at the DFT/LC-PBE/def2-TZVPPD+CPCM(High dielectric) level of theory were used for all analyses. RDG-NCI plots were generated using code provided with MultiWFN 3.8 in the “examples” folder, along with GNUplot. Visualizations of the 3D surfaces of the RDG-NCI plots were achieved through implementing code found in the “examples” folder of MultiWFN 3.8, in conjunction with VMD.[46]

From the QTAIM analysis, several parameters were obtained that are traditionally used to characterise non-covalent interactions. All values were obtained at the bond critical points (BCPs), and properties obtained include the electron density (ρ_{BCP}), energy density (H_{BCP}), the laplacian of electron density ($\nabla^2\rho_{BCP}$), lagrangian kinetic energy (G(r)), hamiltonian kinetic energy (H(r)) and potential energy density (V(r)). From the electron density, the binding energy for neutral hydrogen bonds and ionic hydrogen bonds can be estimated using **Equation S20** and **Equation S21**, respectively:[47]

$$BE_{neutral} = -223.08\ \rho_{BCP} + 0.7423$$ **Equation S20**

$$BE_{charged} = -332.34\ \rho_{BCP} - 1.0661$$ **Equation S21**

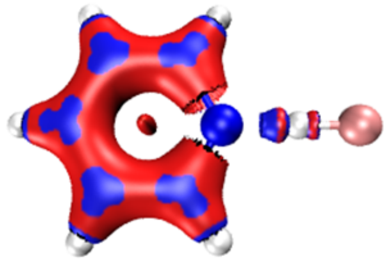

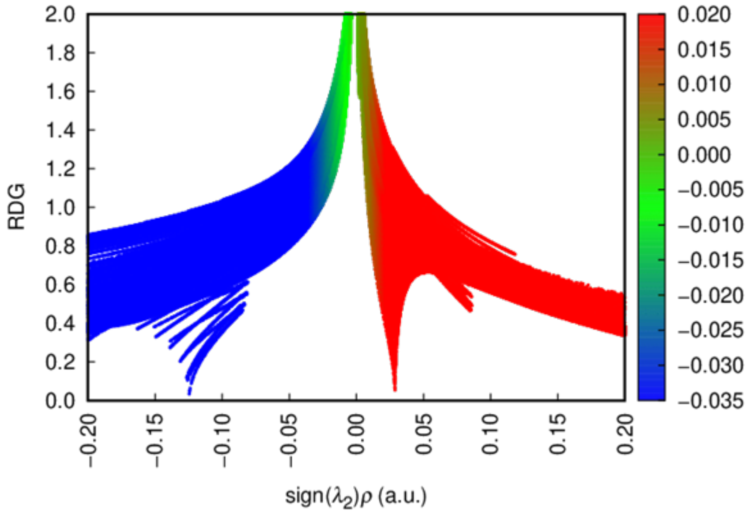

Figure S20: The RDG-NCI plot and RDG-NCI map for 1HF·py. Geometry was optimised at DFT/LC-PBE/def2-TZVPPD+CPCM(High dielectric)

Table S11: The QTAIM properties for non-covalent interactions within the 1HF·py complex. Values were obtained using the DFT/LC-PBE/def2-TZVPPD+CPCM(High dielectric) results.

NCI	ρ_{bcp} / e Å^{-3}	H_{bcp} / Ha Å^{-3}	$\nabla^2\rho_{bcp}$ / e Å^{-5}	G(r)	V(r)	K(r)	sign(λ_2)ρ	BE (neutral)	BE (charged)
N^+-H···F^-	0.12	-0.09	-0.11	0.06	-0.15	0.09	-0.12	-27.0	-

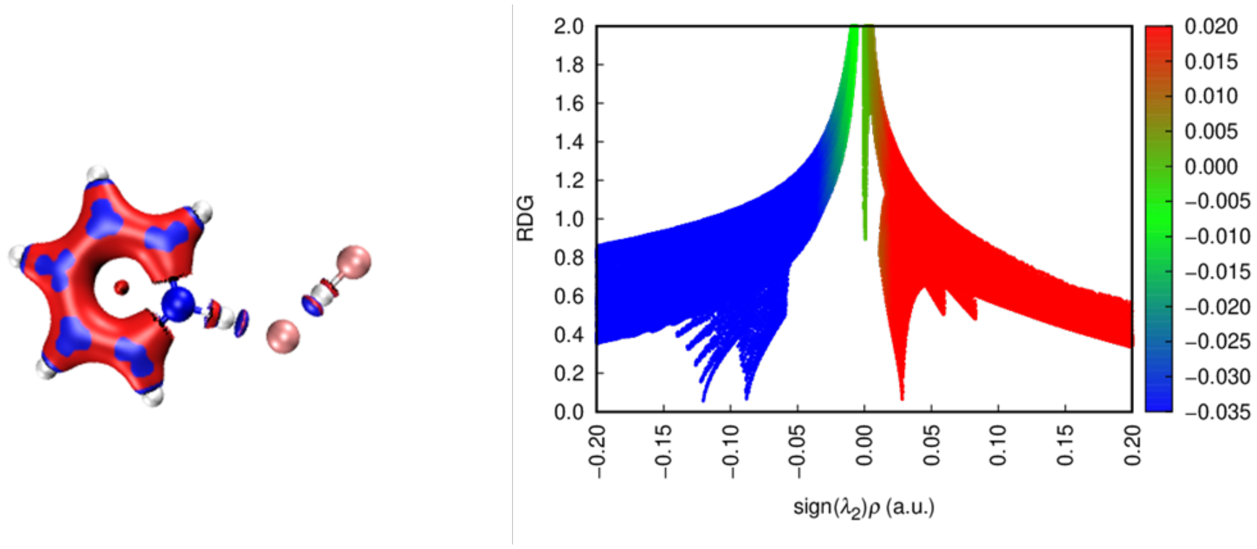

Figure S21: The RDG-NCI plot and RDG-NCI map for 2HF·py. Geometry was optimised at DFT/LC-PBE/def2-TZVPPD+CPCM(High dielectric)

Table S12: The QTAIM properties for non-covalent interactions within the 2HF·py complex. Values were obtained using the DFT/LC-PBE/def2-TZVPPD+CPCM(High dielectric) results.

NCI	ρ_{bcp} / e Å^{-3}	H_{bcp} / Ha Å^{-3}	$\nabla^2\rho_{bcp}$ / e Å^{-5}	G(r)	V(r)	K(r)	sign(λ_2)ρ	BE (neutral)	BE (charged)
N^+-H···F^-	0.09	-0.04	0.14	0.08	-0.12	0.04	-0.09	-	-30.38
F^-···HF	0.12	-0.09	0.04	0.10	-0.19	0.09	-0.12	-	-41.05

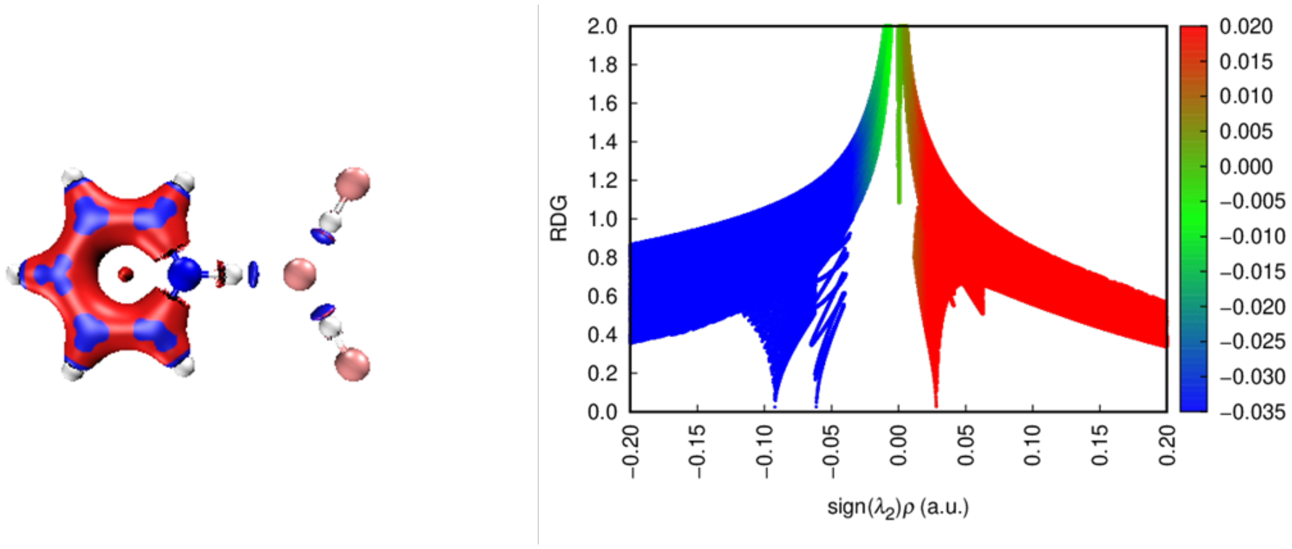

Figure S22: The RDG-NCI plot and RDG-NCI map for 3HF·py. Geometry was optimised at DFT/LC-PBE/def2-TZVPPD+CPCM(High dielectric)

Table S13: The QTAIM properties for non-covalent interactions within the 3HF·py complex. Values were obtained using the DFT/LC-PBE/def2-TZVPPD+CPCM(High dielectric) results.

NCI	ρ_{bcp} / e Å^{-3}	H_{bcp} / Ha Å^{-3}	$\nabla^2\rho_{bcp}$ / e Å^{-5}	G(r)	V(r)	K(r)	sign(λ_2)ρ	BE (neutral)	BE (charged)
N^+-H···F^-	0.06	-0.02	0.14	0.05	-0.07	0.02	-0.06	-	-21.48
F^-···HF	0.09	-0.05	0.13	0.08	-0.13	0.05	-0.09	-	-31.73
F^-···HF	0.09	-0.05	0.13	0.08	-0.13	0.05	-0.09	-	-31.73

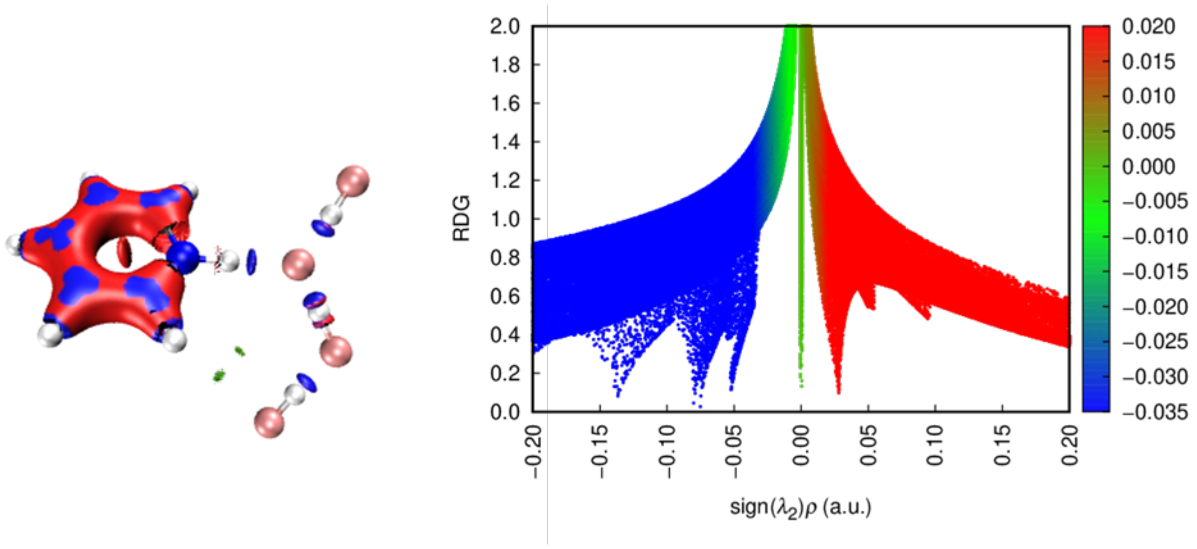

Figure S23: The RDG-NCI plot and RDG-NCI map for 4HF·py. Geometry was optimised at DFT/LC-PBE/def2-TZVPPD+CPCM(High dielectric)

Table S14: The QTAIM properties for non-covalent interactions within the 4HF·py complex. Values were obtained using the DFT/LC-PBE/def2-TZVPPD+CPCM(High dielectric) results.

NCI	ρ_{bcp} / e Å^{-3}	H_{bcp} / Ha Å^{-3}	$\nabla^2\rho_{bcp}$ / e Å^{-5}	G(r)	V(r)	K(r)	sign(λ_2)ρ	BE (neutral)	BE (charged)
N^+-H···F^-	0.05	-0.01	0.14	0.05	-0.06	0.01	-0.05	-	-18.41
F^-···HF	0.14	-0.13	-0.09	0.10	-0.23	0.13	-0.14	-	-46.78
H-F···H-F (secondary)	0.08	-0.03	0.14	0.07	-0.10	0.03	-0.08	-16.04	-
F^-···HF	0.08	-0.04	0.14	0.07	-0.10	0.04	-0.08	-	-27.69

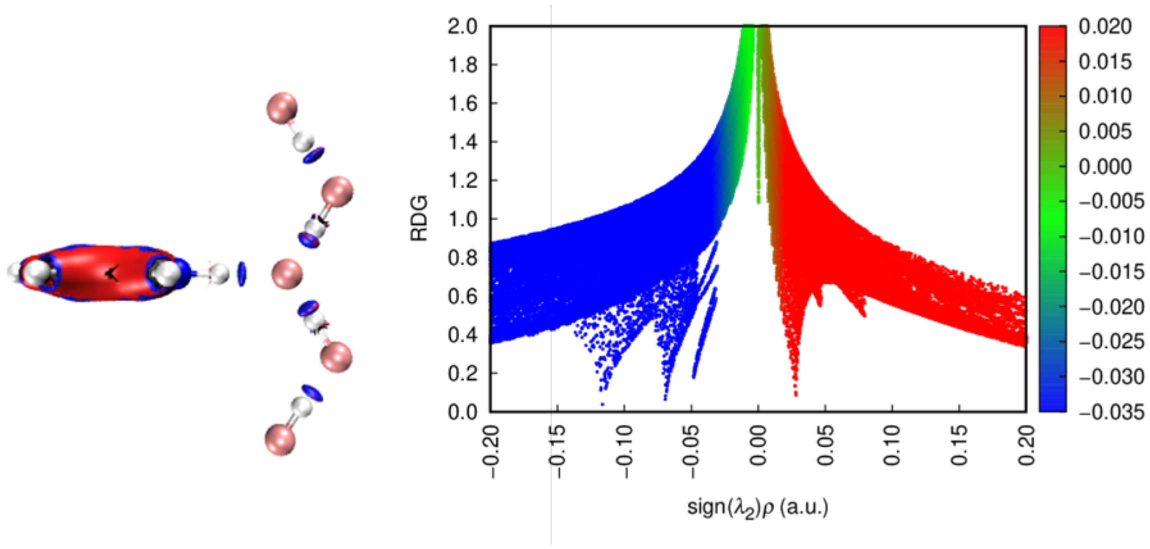

Figure S24: The RDG-NCI plot and RDG-NCI map for 5HF·py. Geometry was optimised at DFT/LC-PBE/def2-TZVPPD+CPCM(High dielectric)

Table S15: The QTAIM properties for non-covalent interactions within the 5HF·py complex. Values were obtained using the DFT/LC-PBE/def2-TZVPPD+CPCM(High dielectric) results.

NCI	ρ_{bcp} / e Å^{-3}	H_{bcp} / Ha Å^{-3}	$\nabla^2\rho_{bcp}$ / e Å^{-5}	G(r)	V(r)	K(r)	sign(λ_2)ρ	BE (neutral)	BE (charged)
N^+-H···F^-	0.05	-0.01	0.13	0.04	-0.05	0.01	-0.05	-	-16.94
F^-···HF	0.12	-0.08	0.04	0.09	-0.18	0.08	-0.12	-	-39.84
H-F···H-F (secondary)	0.07	-0.03	0.14	0.06	-0.09	0.03	-0.07	-14.75	-
F^-···HF	0.12	-0.08	0.04	0.09	-0.18	0.08	-0.12	-	-39.84
H-F···H-F (secondary)	0.07	-0.03	0.14	0.06	-0.09	0.03	-0.07	-14.76	-

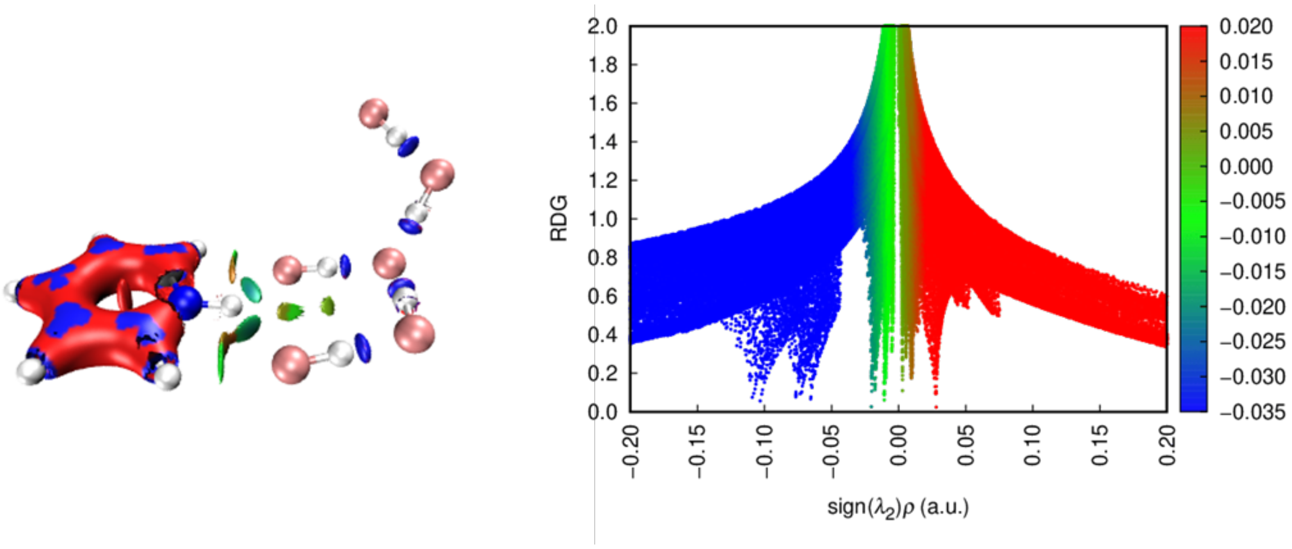

Figure S25: The RDG-NCI plot and RDG-NCI map for 6HF·py. Geometry was optimised at DFT/LC-PBE/def2-TZVPPD+CPCM(High dielectric)

Table S16: The QTAIM properties for non-covalent interactions within the 6HF·py complex. Values were obtained using the DFT/LC-PBE/def2-TZVPPD+CPCM(High dielectric) results.

NCI	ρ_{bcp} / e Å^{-3}	H_{bcp} / Ha Å^{-3}	$\nabla^2\rho_{bcp}$ / e Å^{-5}	G(r)	V(r)	K(r)	sign(λ_2)ρ	BE (neutral)	BE (charged)
N^+-H···F^-	0.02	0.00	0.09	0.02	-0.02	0.00	-0.02	-	-7.85
N^+-H···F^-	0.02	0.00	0.08	0.02	-0.01	0.00	-0.02	-	-7.00
F^-···HF	0.10	-0.06	0.10	0.09	-0.15	0.06	-0.10	-	-35.35
H-F···H-F (secondary)	0.07	-0.02	0.14	0.06	-0.08	0.02	-0.07	-13.96	-
F^-···HF	0.11	-0.07	0.07	0.09	-0.16	0.07	-0.11	-	-37.40
H-F···H-F (secondary)	0.07	-0.03	0.13	0.06	-0.09	0.03	-0.07	-15.59	-
F^-···HF	0.08	-0.03	0.13	0.07	-0.10	0.03	-0.08	-	-26.70

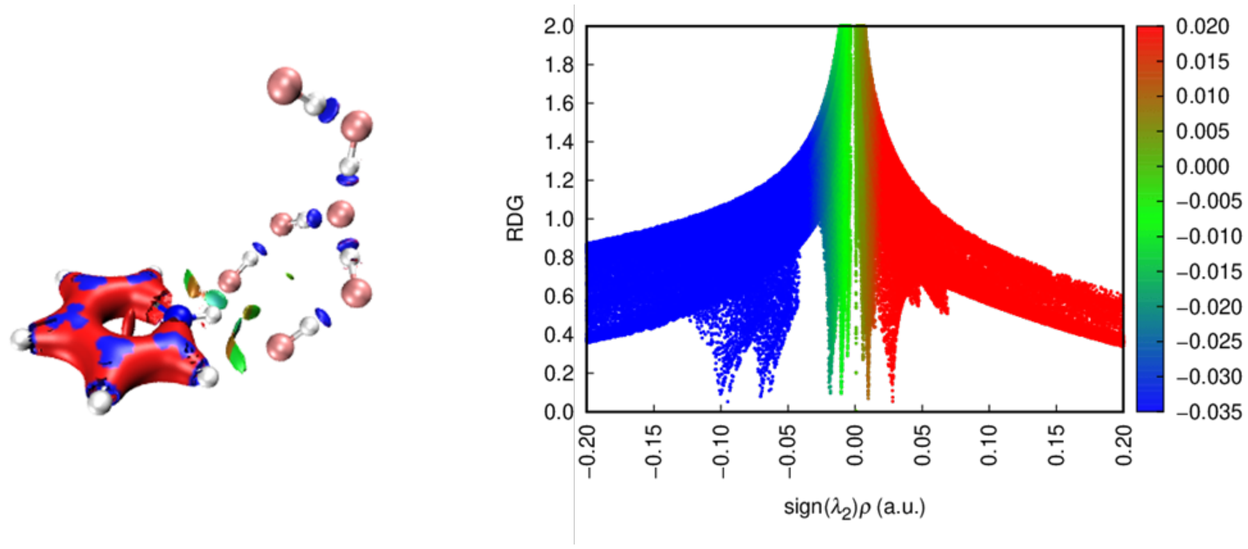

Figure S26: The RDG-NCI plot and RDG-NCI map for 7HF·py. Geometry was optimised at DFT/LC-PBE/def2-TZVPPD+CPCM(High dielectric)

Table S17: The QTAIM properties for non-covalent interactions within the 7HF·py complex. Values were obtained using the DFT/LC-PBE/def2-TZVPPD+CPCM(High dielectric) results.

NCI	ρ_{bcp} / e Å^{-3}	H_{bcp} / Ha Å^{-3}	$\nabla^2\rho_{bcp}$ / e Å^{-5}	G(r)	V(r)	K(r)	sign(λ_2)ρ	BE (neutral)	BE (charged)
N^+-H···F^-	0.02	0.00	0.08	0.02	-0.02	0.00	-0.02	-	-7.25
N^+-H···F^-	0.02	0.00	0.09	0.02	-0.02	0.00	-0.02	-	-7.52
F^-···HF	0.10	-0.06	0.10	0.08	-0.14	0.06	-0.10	-	-34.69
H-F···H-F (secondary)	0.07	-0.03	0.14	0.06	-0.09	0.03	-0.07	-15.03	-
F^-···HF	0.10	-0.06	0.10	0.08	-0.14	0.06	-0.10	-	-34.52
H-F···H-F (secondary)	0.07	-0.03	0.14	0.06	-0.09	0.03	-0.07	-14.99	-
F^-···HF	0.09	-0.05	0.12	0.08	-0.13	0.05	-0.09	-	-32.59
H-F···H-F (secondary)	0.06	-0.02	0.14	0.06	-0.08	0.02	-0.06	-13.51	-

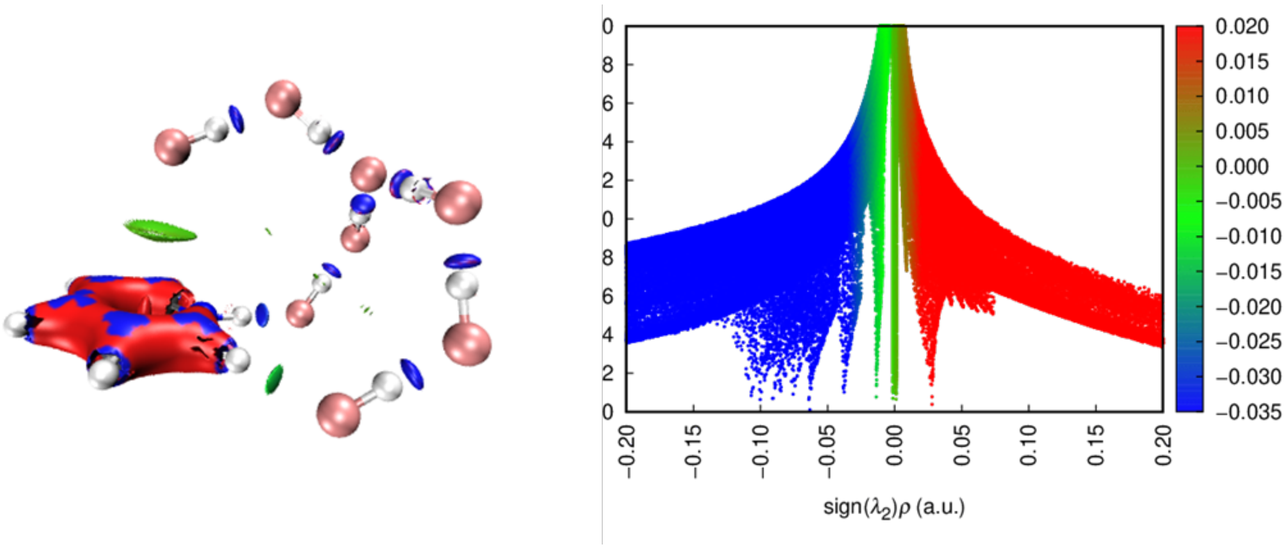

Figure S27: The RDG-NCI plot and RDG-NCI map for 8HF·py. Geometry was optimised at DFT/LC-PBE/def2-TZVPPD+CPCM(High dielectric)

Table S18: The QTAIM properties for non-covalent interactions within the 8HF·py complex. Values were obtained using the DFT/LC-PBE/def2-TZVPPD+CPCM(High dielectric) results.

NCI	ρ_{bcp} / e Å^{-3}	H_{bcp} / Ha Å^{-3}	$\nabla^2\rho_{bcp}$ / e Å^{-5}	G(r)	V(r)	K(r)	sign(λ_2)ρ	BE (neutral)	BE (charged)
N^+-H···F^-	0.04	-0.01	0.12	0.03	-0.04	0.01	-0.04	-	-13.76
F^-···HF	0.11	-0.07	0.07	0.09	-0.16	0.07	-0.11	-	-36.98
H-F···H-F (secondary)	0.09	-0.04	0.13	0.07	-0.12	0.04	-0.09	-18.42	-
H-F···H-F (tertiary)	0.06	-0.02	0.13	0.06	-0.08	0.02	-0.06	-13.57	-
F^-···HF	0.09	-0.05	0.12	0.08	-0.13	0.05	-0.09	-	-31.40
H-F···H-F (secondary)	0.06	-0.02	0.13	0.06	-0.08	0.02	-0.06	-13.38	-
F^-···HF	0.10	-0.06	0.10	0.08	-0.14	0.06	-0.10	-	-34.58
H-F···H-F (secondary)	0.08	-0.03	0.14	0.07	-0.10	0.03	-0.08	-16.25	-

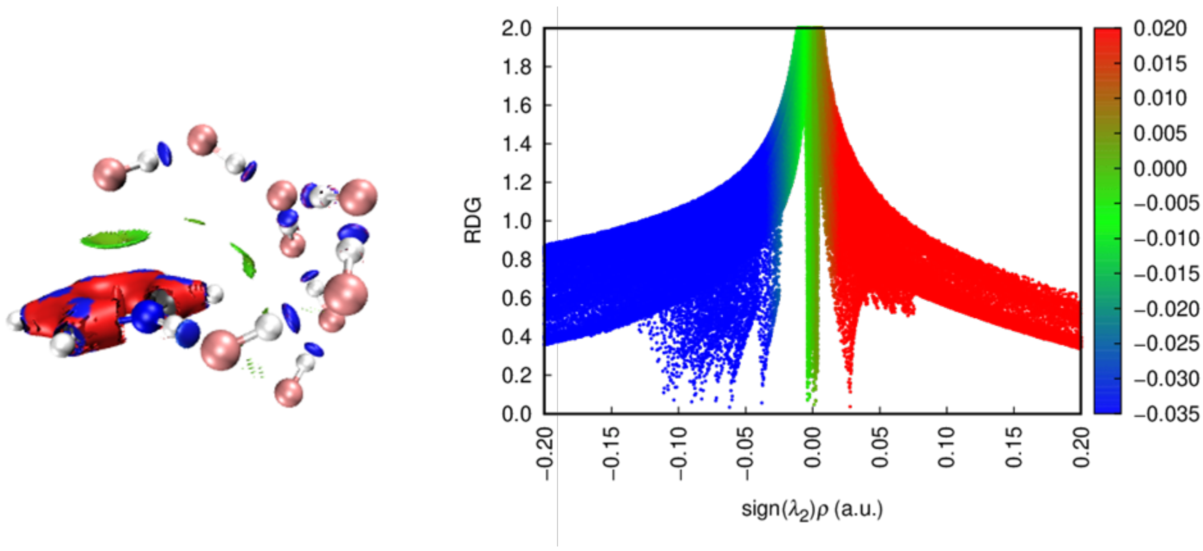

Figure S28: The RDG-NCI plot and RDG-NCI map for 9HF·py. Geometry was optimised at DFT/LC-PBE/def2-TZVPPD+CPCM(High dielectric)

Table S19: The QTAIM properties for non-covalent interactions within the 9HF·py complex. Values were obtained using the DFT/LC-PBE/def2-TZVPPD+CPCM(High dielectric) results.

NCI	ρ_{bcp} / e Å^{-3}	H_{bcp} / Ha Å^{-3}	$\nabla^2\rho_{bcp}$ / e Å^{-5}	G(r)	V(r)	K(r)	sign(λ_2)ρ	BE (neutral)	BE (charged)
N^+-H···F^-	0.04	0.00	0.12	0.03	-0.04	0.00	-0.04	-	-13.69
F^-···HF	0.11	-0.07	0.06	0.09	-0.17	0.07	-0.11	-	-37.75
H-F···H-F (secondary)	0.09	-0.05	0.12	0.08	-0.12	0.05	-0.09	-19.50	-
H-F···H-F (tertiary)	0.07	-0.03	0.14	0.06	-0.09	0.03	-0.07	-15.43	-
F^-···HF	0.10	-0.07	0.09	0.09	-0.15	0.07	-0.10	-	-35.74
H-F···H-F (secondary)	0.08	-0.04	0.13	0.07	-0.11	0.04	-0.08	-17.86	-
H-F···H-F (tertiary)	0.06	-0.02	0.13	0.05	-0.07	0.02	-0.06	-12.75	-
F^-···HF	0.09	-0.04	0.13	0.08	-0.12	0.04	-0.09	-	-30.49
H-F···H-F (secondary)	0.06	-0.02	0.13	0.05	-0.08	0.02	-0.06	-13.09	-

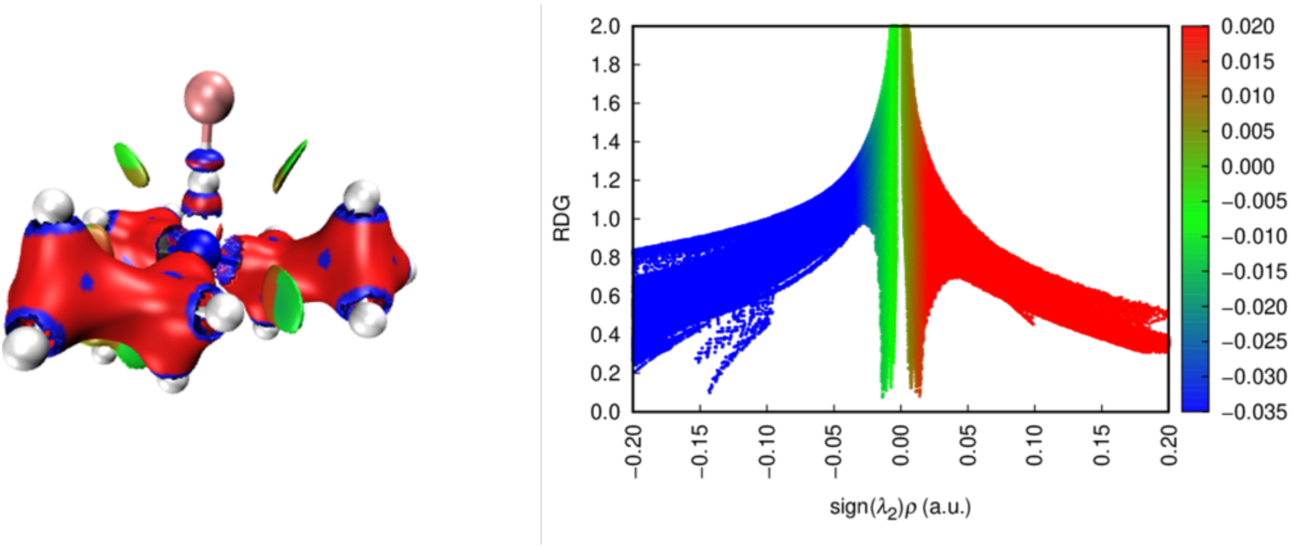

Figure S29: The RDG-NCI plot and RDG-NCI map for 1HF·TEA. Geometry was optimised at DFT/LC-PBE/def2-TZVPPD+CPCM(High dielectric)

Table S20: The QTAIM properties for non-covalent interactions within the 1HF·TEA complex. Values were obtained using the DFT/LC-PBE/def2-TZVPPD+CPCM(High dielectric) results.

NCI	ρ_{bcp} / e Å^{-3}	H_{bcp} / Ha Å^{-3}	$\nabla^2\rho_{bcp}$ / e Å^{-5}	G(r)	V(r)	K(r)	sign(λ_2)ρ	BE (neutral)	BE (charged)
N^+-H···F^-	0.14	-0.12	-0.04	0.11	-0.23	0.12	-0.14	-	-48.38

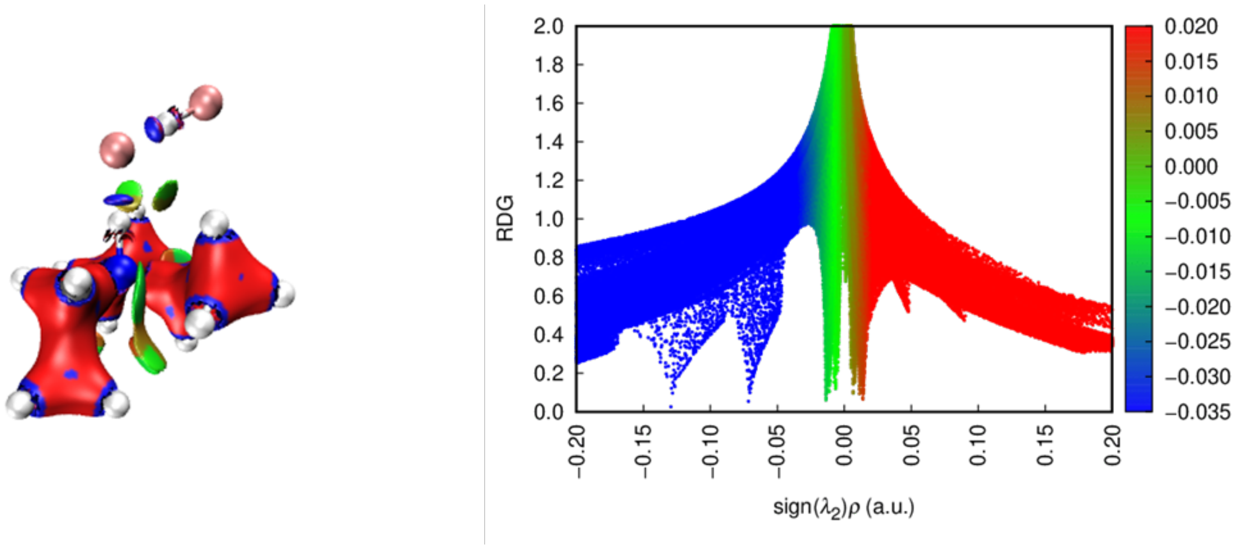

Figure S30: The RDG-NCI plot and RDG-NCI map for 2HF·TEA**.** Geometry was optimised at DFT/LC-PBE/def2-TZVPPD+CPCM(High dielectric)

Table S21: The QTAIM properties for non-covalent interactions within the 2HF·TEA complex. Values were obtained using the DFT/LC-PBE/def2-TZVPPD+CPCM(High dielectric) results.

NCI	ρ_{bcp} / e Å^{-3}	H_{bcp} / Ha Å^{-3}	$\nabla^2\rho_{bcp}$ / e Å^{-5}	G(r)	V(r)	K(r)	sign(λ_2)ρ	BE (neutral)	BE (charged)
N^+-H···F^-	0.07	-0.03	0.15	0.06	-0.09	0.03	-0.07	-	-24.83
F^-···HF	0.13	-0.11	-0.02	0.10	-0.21	0.11	-0.13	-	-44.17

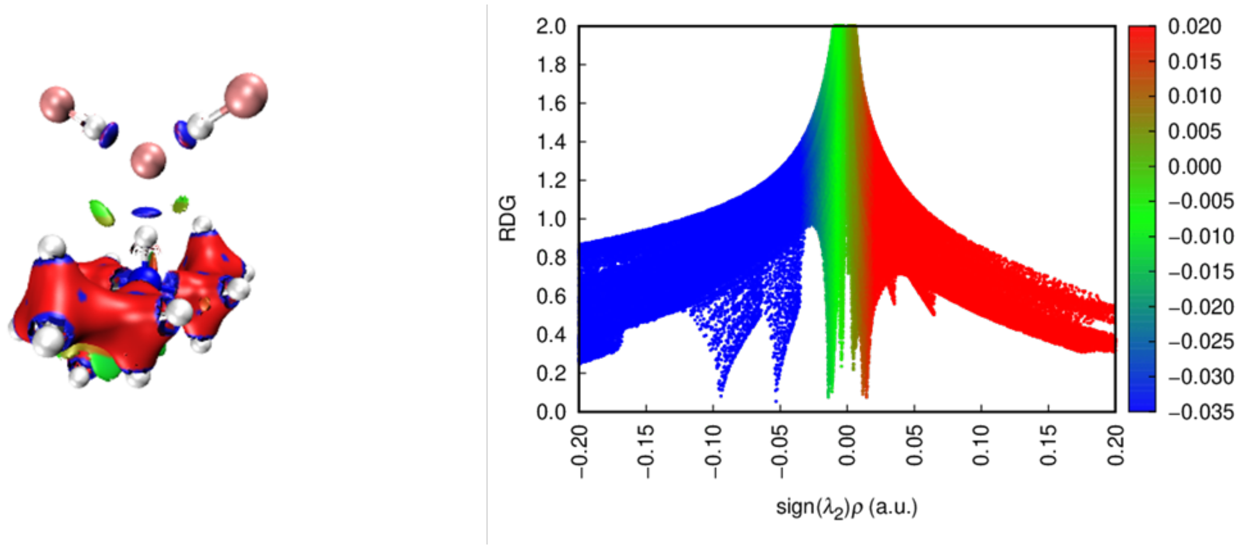

Figure S31: The RDG-NCI plot and RDG-NCI map for 3HF·TEA**.** Geometry was optimised at DFT/LC-PBE/def2-TZVPPD+CPCM(High dielectric)

Table S22: The QTAIM properties for non-covalent interactions within the 3HF·TEA complex. Values were obtained using the DFT/LC-PBE/def2-TZVPPD+CPCM(High dielectric) results.

NCI	ρ_{bcp} / e Å^{-3}	H_{bcp} / Ha Å^{-3}	$\nabla^2\rho_{bcp}$ / e Å^{-5}	G(r)	V(r)	K(r)	sign(λ_2)ρ	BE (neutral)	BE (charged)
N^+-H···F^-	0.05	-0.01	0.14	0.05	-0.06	0.01	-0.05	-	-18.81
F^-···HF	0.09	-0.05	0.12	0.08	-0.13	0.05	-0.09	-	-32.61
F^-···HF	0.10	-0.05	0.12	0.08	-0.13	0.05	-0.10	-	-32.65

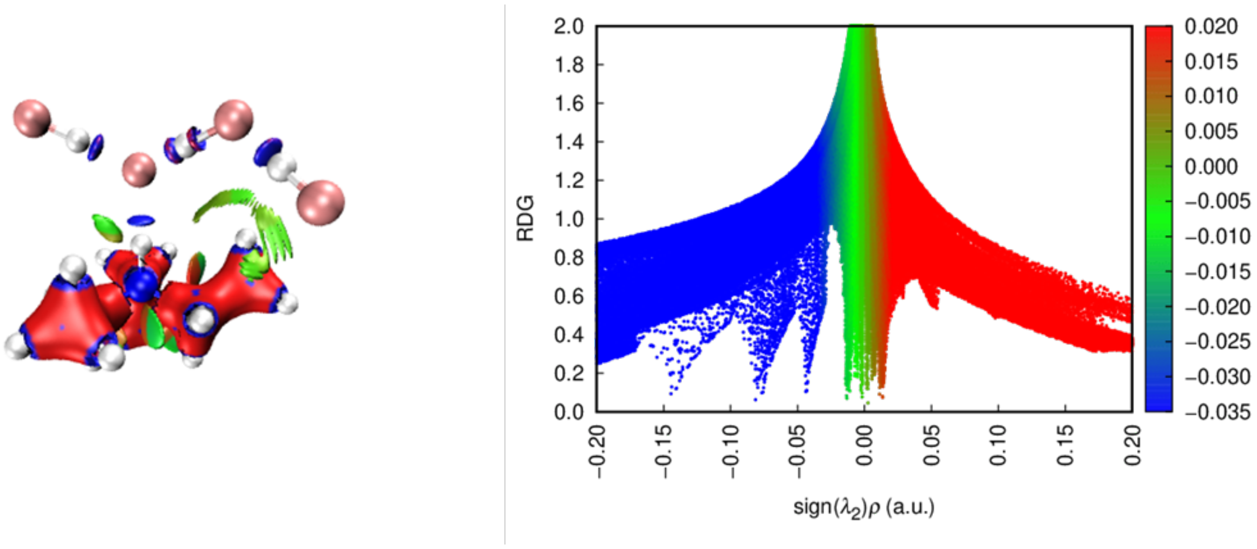

Figure S32: The RDG-NCI plot and RDG-NCI map for 4HF·TEA**.** Geometry was optimised at DFT/LC-PBE/def2-TZVPPD+CPCM(High dielectric)

Table S23: The QTAIM properties for non-covalent interactions within the 4HF·TEA complex. Values were obtained using the DFT/LC-PBE/def2-TZVPPD+CPCM(High dielectric) results.

NCI	ρ_{bcp} / e Å^{-3}	H_{bcp} / Ha Å^{-3}	$\nabla^2\rho_{bcp}$ / e Å^{-5}	G(r)	V(r)	K(r)	sign(λ_2)ρ	BE (neutral)	BE (charged)
N^+-H···F^-	0.04	-0.01	0.13	0.04	-0.05	0.01	-0.04	-	-15.64
F^-···HF	0.14	-0.14	-0.14	0.11	-0.25	0.14	-0.14	-	-48.75
H-F···H-F (secondary)	0.08	-0.03	0.14	0.07	-0.10	0.03	-0.08	-16.41	-
F^-···HF	0.08	-0.04	0.14	0.07	-0.11	0.04	-0.08	-	-28.18

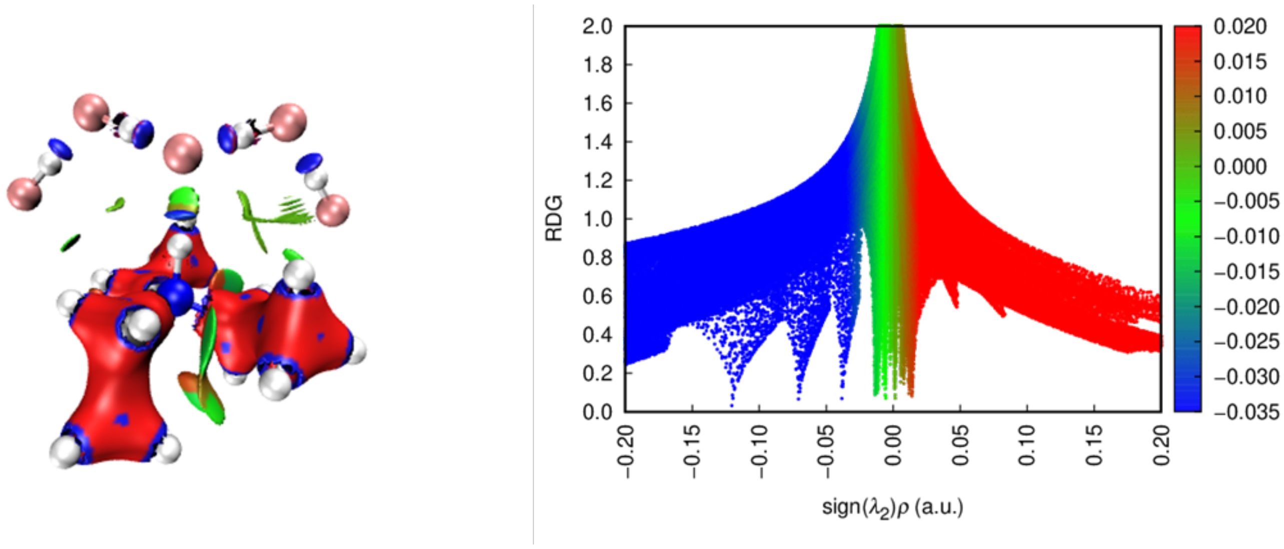

Figure S33: The RDG-NCI plot and RDG-NCI map for 5HF·TEA**.** Geometry was optimised at DFT/LC-PBE/def2-TZVPPD+CPCM(High dielectric)

Table S24: The QTAIM properties for non-covalent interactions within the 5HF·TEA complex. Values were obtained using the DFT/LC-PBE/def2-TZVPPD+CPCM(High dielectric) results.

NCI	ρ_{bcp} / e Å^{-3}	H_{bcp} / Ha Å^{-3}	$\nabla^2\rho_{bcp}$ / e Å^{-5}	G(r)	V(r)	K(r)	sign(λ_2)ρ	BE (neutral)	BE (charged)
N^+-H···F^-	0.04	-0.01	0.12	0.03	-0.04	0.01	-0.04	-	-13.84
F^-···HF	0.12	-0.09	0.02	0.10	-0.19	0.09	-0.12	-	-41.11
H-F···H-F (secondary)	0.07	-0.03	0.14	0.06	-0.09	0.03	-0.07	-15.06	-
F^-···HF	0.12	-0.09	0.02	0.10	-0.19	0.09	-0.12	-	-41.11
H-F···H-F (secondary)	0.07	-0.03	0.14	0.06	-0.09	0.03	-0.07	-15.06	-

5.6 Nucleophilicity of anionic clusters

The nucleophilicity of the anionic clusters can be related to the ionisation potential (I_s) and the electron affinity (A) using several equations, as shown with **Equations S22 – S25.**[48]

$$N = -I_s \tag{Equation S22}$$

$$N' = \frac{2(I_s - A)}{(I_s + A)^2} \tag{Equation S23}$$

$$N'' = \frac{20(I_s - A)}{I_s^2} \tag{Equation S24}$$

$$N''' = \frac{160(I_s - A)}{(3I_s + A)^2} \tag{Equation S25}$$

The value for the ionisation potential can be estimated using Koopman's Theorem, which states that the ionisation potential is the negative value of the HOMO of the molecule.[49] Though not stated in the paper, a common convention is to estimate the electron affinity of the molecule by taking the negative of the LUMO. However, due to the only the fluoride anion reacting rather than the whole anionic cluster, the local ionisation potential ($\bar{I}(\mathbf{r})$) and local electron affinity ($\overline{A_E}(\mathbf{r})$) can also be used to probe the reactivity of the fluoride anion within the cluster, as shown in **Equation S26** and **Equation S27**, respectively.

$$\bar{I}(\mathbf{r}) = \frac{\sum_i \rho_i(\mathbf{r})|\varepsilon_i|}{\rho(\mathbf{r})} \tag{Equation S26}$$

$$\overline{A_E}(\mathbf{r}) = \frac{-\sum_{i \in vir}|\phi_i(\mathbf{r})|^2 \varepsilon_i}{\sum_{i \in vir}|\phi_i(\mathbf{r})|^2} \tag{Equation S27}$$

where $\rho_i(\mathbf{r})$ represents the electronic density of the *i*th molecular orbital at position **r**, ε_i is the orbital energy, $\rho(\mathbf{r})$ is the total electronic density and φ_i is the orbital wavefunction.[50–52] The calculated nucleophilicities in **Equations S22 – S25** are shown in **Figure S34**. When considering ($\bar{I}(\mathbf{r})$) alone, a clear trend can be observed between the nucleophilicity of the fluoride anion and the size of the solvation shell. However, for equations that make use of $\overline{A_E}(\mathbf{r})$, no clear trend is observed between cluster size and nucleophilicity. This is due to the equation used to calculate $\overline{A_E}(\mathbf{r})$, as it incorporates orbitals that are very high in energy and therefore have no chemical relevance. To overcome this the local electron attachment energy ($E_{att}(\mathbf{r})$) could be used as a more robust estimation (**Equation S28**). However, as the CCSD level of theory for these anionic clusters, the value of $E_{attr}(r)$ is zero (See **Table S25**).

$$E_{att}(\mathbf{r}) = \frac{2\sum_{i=LUMO}^{\varepsilon_i<0}|\phi_i(\mathbf{r})|^2 \varepsilon_i}{\rho(\mathbf{r})} \tag{Equation S28}$$

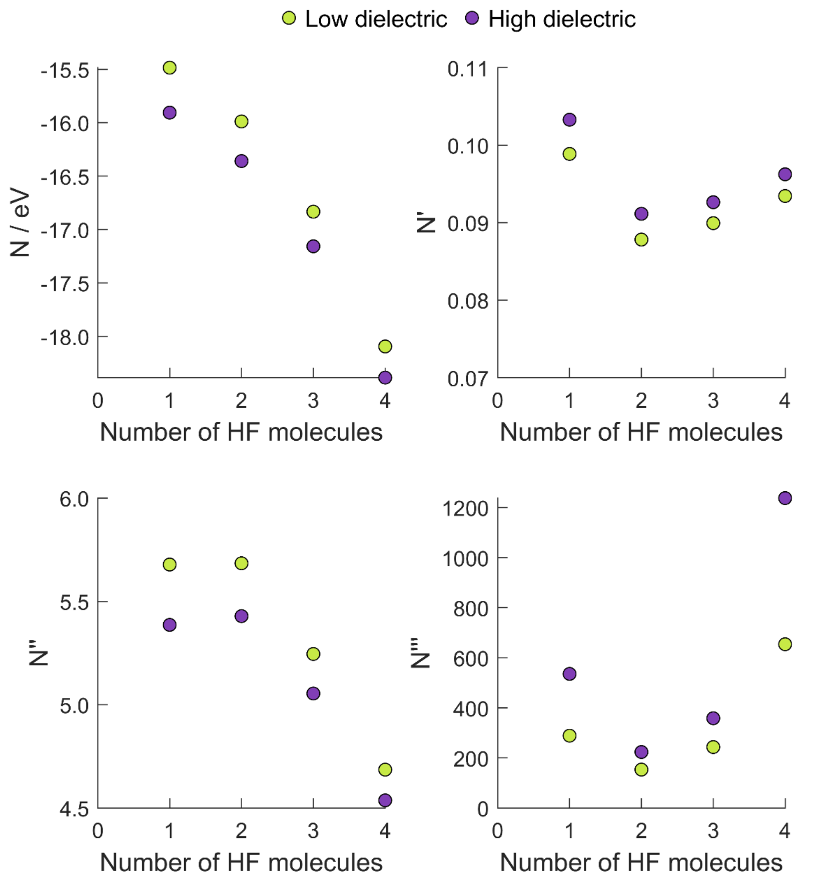

Figure S34: The calculated nucleophilicities of $[F(HF)_x]^-$ anions (x = 1-4) using CCSD/ def2-TZVPPD +CPCM(custom), when the local electron affinity is used. High dielectric: ε_{static} = 69.8; $\varepsilon_{dynamic}$ = 1.5876. Low dielectric: ε_{static} = 12.1; $\varepsilon_{dynamic}$ = 1.8769.

Table S25: The calculated HOMO and LUMO values for $[F(HF)_x]^-$ anions (x = 1-4) using CCSD/ def2-TZVPPD+CPCM(custom). High dielectric: ε_{static} = 69.8; $\varepsilon_{dynamic}$ = 1.5876. Low dielectric: ε_{static} = 12.1; $\varepsilon_{dynamic}$ = 1.8769.

Anionic cluster	HOMO (Low dielectric)	LUMO (Low dielectric)	HOMO (High dielectric)	LUMO (High dielectric)
$[FHF]^-$	-13.27	1.63	-13.69	1.35
$[F(HF)_2]^-$	-14.20	1.55	-14.56	1.30
$[F(HF)_3]^-$	-15.04	1.51	-15.35	1.27
$[F(HF)_4]^-$	-15.73	1.47	-16.01	1.24

5.7 Nucleophilicity of *n*HF·base clusters

The nucleophilicities of the different *n*HF·base clusters were then calculated using **Equations S22 – S25**. Both global and local descriptors were considered to calculate the nucleophilicities, as shown in **Table S26 – S76**. It should be noted that when global descriptions were considered to calculate the nucleophilicities, the opposite trend was observed compared to what could be determined experimentally. Therefore, we chose to use the calculated nucleophilicities using local descriptors.

Table S26: The calculated local and global properties used to calculate the nucleophilicities using DLPNO-CCSD/def2-TZVPPD+CPCM(high) for *n*HF·base clusters. $\bar{I}(\mathbf{r})$: Average local ionisation energy; $\overline{A_E}(\mathbf{r})$: Local electron affinity; $E_{att}(\mathbf{r})$: Local electron attachment energy; HOMO: Highest occupied molecular orbital; LUMO: Lowest unoccupied molecular orbital

	Local properties on fluoride anion in *n*HF·base cluster			Global properties for *n*HF·base cluster	
***n*HF·base**	**$\bar{I}$(r) / eV**	**$\overline{A_E}$(r) / eV**	**E_{att}(r) / eV**	**HOMO / eV**	**LUMO / eV**
1HF·py	17.65	-29.81	0.00	-9.79	1.89
2HF·py	16.62	-31.34	0.00	-10.30	1.68
3HF·py	17.29	-33.48	0.00	-10.43	1.52
4HF·py	17.81	-32.54	0.00	-10.47	1.45
5HF·py	18.09	-33.38	0.00	-10.44	1.48
6HF·py	18.40	-33.55	0.00	-10.41	1.45
7HF·py	18.57	-33.39	0.00	-10.38	1.48
8HF·py	18.75	-34.02	0.00	-10.44	1.42
9HF·py	18.78	-34.05	0.00	-10.42	1.45
1HF·TEA	16.61	-32.57	0.00	-11.17	1.99
2HF·TEA	16.35	-33.13	0.00	-13.14	1.94
3HF·TEA	17.18	-34.27	0.00	-13.50	1.91
4HF·TEA	17.74	-35.11	0.00	-13.32	1.90
5HF·TEA	18.00	-35.23	0.00	-13.31	1.87

Table S27: The calculated nucleophilicities using DLPNO-CCSD/def2-TZVPPD+CPCM(high) for *n*HF·base clusters when using local descriptors.

***n*HF·base**	**N**	**N'**	**N''**	**N'''**
1HF·py	-17.65	0.45	1.13	1.01
2HF·py	-16.62	0.48	1.20	1.07
3HF·py	-17.29	0.46	1.16	1.03
4HF·py	-17.81	0.45	1.12	1.00
5HF·py	-18.09	0.44	1.11	0.98
6HF·py	-18.40	0.43	1.09	0.97
7HF·py	-18.57	0.43	1.08	0.96
8HF·py	-18.75	0.43	1.07	0.95
9HF·py	-18.78	0.43	1.06	0.95
1HF·TEA	-16.61	0.48	1.20	1.07
2HF·TEA	-16.35	0.49	1.22	1.09
3HF·TEA	-17.18	0.47	1.16	1.03
4HF·TEA	-17.74	0.45	1.13	1.00
5HF·TEA	-18.00	0.44	1.11	0.99

Table S28: The calculated nucleophilicities using DLPNO-CCSD/def2-TZVPPD+CPCM(high) for *n*HF·base clusters when using global descriptors.

***n*HF·base**	**N**	**N'**	**N''**	**N'''**
1HF·py	-9.79	1.50	2.44	2.47
2HF·py	-10.30	1.29	2.26	2.24
3HF·py	-10.43	1.20	2.20	2.16
4HF·py	-10.47	1.17	2.18	2.13
5HF·py	-10.44	1.19	2.19	2.14
6HF·py	-10.41	1.18	2.19	2.14
7HF·py	-10.38	1.20	2.20	2.15
8HF·py	-10.44	1.16	2.17	2.12
9HF·py	-10.42	1.18	2.19	2.14
1HF·TEA	-11.17	1.25	2.11	2.12
2HF·TEA	-13.14	0.96	1.75	1.72
3HF·TEA	-13.50	0.92	1.69	1.66
4HF·TEA	-13.32	0.93	1.72	1.68
5HF·TEA	-13.31	0.93	1.71	1.68

Table S29: The calculated local and global properties used to calculate the nucleophilicities using DFT/B2PLYP-D4/def2-TZVPPD+CPCM(high) for *n*HF·base clusters. $\bar{I}(\mathbf{r})$: Average local ionisation energy; $\overline{A_E}(\mathbf{r})$: Local electron affinity; $E_{att}(\mathbf{r})$: Local electron attachment energy; HOMO: Highest occupied molecular orbital; LUMO: Lowest unoccupied molecular orbital

	Local properties on fluoride anion in *n*HF·base cluster			Global properties for *n*HF·base cluster	
***n*HF·base**	**$\bar{I}$(r) / eV**	**$\overline{A_E}$(r) / eV**	**E_{att}(r) / eV**	**HOMO / eV**	**LUMO / eV**
1HF·py	13.91	-26.84	0.00	-8.62	0.04
2HF·py	12.89	-28.16	0.00	-9.15	-0.63
3HF·py	13.64	-29.50	-0.01	-9.23	-0.74
4HF·py	14.10	-28.52	0.00	-9.25	-0.78
5HF·py	14.33	-29.56	0.00	-9.26	-0.80
6HF·py	14.58	-29.49	0.00	-9.27	-0.84
7HF·py	14.73	-29.55	0.00	-9.25	-0.82
8HF·py	14.83	-29.92	0.00	-9.27	-0.83
9HF·py	14.83	-30.83	0.00	-9.28	-0.83
1HF·TEA	11.50	-28.78	0.00	-9.66	1.03
2HF·TEA	12.74	-30.34	0.00	-10.70	0.94
3HF·TEA	13.54	-30.71	0.00	-11.16	0.86
4HF·TEA	14.02	-31.27	0.00	-11.13	0.88
5HF·TEA	14.22	-31.57	0.00	-11.12	0.84

Table S30: The calculated nucleophilicities using DFT/B2PLYP-D4/def2-TZVPPD+CPCM(high) for *n*HF·base clusters when using local descriptors.

***n*HF·base**	**N**	**N'**	**N''**	**N'''**
1HF·py	-13.91	0.58	1.44	1.28
2HF·py	-12.89	0.62	1.55	1.38
3HF·py	-13.64	0.59	1.47	1.30
4HF·py	-14.10	0.57	1.42	1.26
5HF·py	-14.33	0.56	1.40	1.24
6HF·py	-14.58	0.55	1.37	1.22
7HF·py	-14.73	0.54	1.36	1.21
8HF·py	-14.83	0.54	1.35	1.20
9HF·py	-14.83	0.54	1.35	1.20
1HF·TEA	-11.50	0.70	1.74	1.55
2HF·TEA	-12.74	0.63	1.57	1.40
3HF·TEA	-13.54	0.59	1.48	1.31
4HF·TEA	-14.02	0.57	1.43	1.27
5HF·TEA	-14.22	0.56	1.41	1.25

Table S31: The calculated nucleophilicities using DFT/B2PLYP-D4/def2-TZVPPD+CPCM(high) for *n*HF·base clusters when using global descriptors.

***n*HF·base**	**N**	**N'**	**N''**	**N'''**
1HF·py	-8.62	0.94	2.33	2.08
2HF·py	-9.15	0.71	2.04	1.73
3HF·py	-9.23	0.68	1.99	1.68
4HF·py	-9.25	0.67	1.98	1.67
5HF·py	-9.26	0.67	1.97	1.66
6HF·py	-9.27	0.66	1.96	1.65
7HF·py	-9.25	0.66	1.97	1.65
8HF·py	-9.27	0.66	1.96	1.65
9HF·py	-9.28	0.66	1.96	1.64
1HF·TEA	-9.66	1.15	2.29	2.19
2HF·TEA	-10.70	0.98	2.03	1.92
3HF·TEA	-11.16	0.91	1.93	1.81
4HF·TEA	-11.13	0.91	1.94	1.82
5HF·TEA	-11.12	0.91	1.94	1.81

Table S32: The calculated local and global properties used to calculate the nucleophilicities using DFT/B3LYP/def2-TZVPPD+CPCM(high) for *n*HF·base clusters. $\bar{I}(\mathbf{r})$: Average local ionisation energy; $\overline{A_E}(\mathbf{r})$: Local electron affinity; $E_{att}(\mathbf{r})$: Local electron attachment energy; HOMO: Highest occupied molecular orbital; LUMO: Lowest unoccupied molecular orbital

	Local properties on fluoride anion in *n*HF·base cluster			Global properties for *n*HF·base cluster	
***n*HF·base**	**$\bar{I}$(r) / eV**	**$\overline{A_E}$(r) / eV**	**E_{att}(r) / eV**	**HOMO / eV**	**LUMO / eV**
1HF·py	11.13	-24.92	0.00	-7.68	-1.46
2HF·py	10.37	-26.07	-0.01	-8.20	-2.13
3HF·py	11.07	-27.74	-0.02	-8.28	-2.24
4HF·py	11.48	-28.87	-0.04	-8.32	-2.28
5HF·py	11.71	-29.23	-0.12	-8.33	-2.30
6HF·py	11.96	-28.87	-0.05	-8.11	-2.10
7HF·py	12.10	-28.18	-0.10	-8.38	-2.37
8HF·py	12.13	-28.93	-0.28	-8.39	-2.37
9HF·py	12.17	-29.26	-0.22	-8.39	-2.38
1HF·TEA	9.05	-26.89	0.00	-7.78	0.28
2HF·TEA	10.20	-28.67	0.00	-8.80	0.15
3HF·TEA	11.05	-30.82	0.00	-9.36	0.00
4HF·TEA	11.41	-31.20	-0.01	-9.50	-0.01
5HF·TEA	11.61	-31.39	-0.08	-9.51	-0.08

Table S33: The calculated nucleophilicities using DFT/B3LYP/def2-TZVPPD+CPCM(high) for *n*HF·base clusters when using local descriptors.

***n*HF·base**	**N**	**N'**	**N''**	**N'''**
1HF·py	-11.13	0.72	1.80	1.60
2HF·py	-10.37	0.77	1.93	1.72
3HF·py	-11.07	0.73	1.81	1.61
4HF·py	-11.48	0.70	1.75	1.56
5HF·py	-11.71	0.70	1.72	1.54
6HF·py	-11.96	0.68	1.68	1.50
7HF·py	-12.10	0.68	1.67	1.49
8HF·py	-12.13	0.71	1.69	1.52
9HF·py	-12.17	0.69	1.67	1.51
1HF·TEA	-9.05	0.88	2.21	1.96
2HF·TEA	-10.20	0.78	1.96	1.74
3HF·TEA	-11.05	0.72	1.81	1.61
4HF·TEA	-11.41	0.70	1.76	1.56
5HF·TEA	-11.61	0.70	1.73	1.55

Table S34: The calculated nucleophilicities using DFT/B3LYP/def2-TZVPPD+CPCM(high) for *n*HF·base clusters when using global descriptors.

***n*HF·base**	**N**	**N'**	**N''**	**N'''**
1HF·py	-7.68	0.60	2.11	1.66
2HF·py	-8.20	0.46	1.81	1.36
3HF·py	-8.28	0.44	1.76	1.32
4HF·py	-8.32	0.43	1.74	1.30
5HF·py	-8.33	0.43	1.74	1.29
6HF·py	-8.11	0.46	1.83	1.38
7HF·py	-8.38	0.42	1.71	1.27
8HF·py	-8.39	0.42	1.71	1.27
9HF·py	-8.39	0.41	1.71	1.27
1HF·TEA	-7.78	1.15	2.66	2.42
2HF·TEA	-8.80	0.96	2.31	2.08
3HF·TEA	-9.36	0.85	2.14	1.90
4HF·TEA	-9.50	0.84	2.10	1.87
5HF·TEA	-9.51	0.82	2.09	1.84

Table S35: The calculated local and global properties used to calculate the nucleophilicities using DFT/B3LYP-D4/def2-TZVPPD+CPCM(high) for *n*HF·base clusters. $\bar{I}(\mathbf{r})$: Average local ionisation energy; $\overline{A_E}(\mathbf{r})$: Local electron affinity; $E_{att}(\mathbf{r})$: Local electron attachment energy; HOMO: Highest occupied molecular orbital; LUMO: Lowest unoccupied molecular orbital

	Local properties on fluoride anion in *n*HF·base cluster			Global properties for *n*HF·base cluster	
***n*HF·base**	**$\bar{I}$(r) / eV**	**$\overline{A_E}$(r) / eV**	**E_{att}(r) / eV**	**HOMO / eV**	**LUMO / eV**
1HF·py	11.14	-24.94	0.00	-7.68	-1.45
2HF·py	10.40	-26.31	-0.01	-8.20	-2.11
3HF·py	11.11	-27.69	-0.02	-8.28	-2.22
4HF·py	11.58	-27.19	-0.01	-8.31	-2.27
5HF·py	11.82	-27.73	-0.03	-8.31	-2.28
6HF·py	12.04	-27.61	-0.08	-8.32	-2.32
7HF·py	12.19	-27.57	-0.12	-8.32	-2.31
8HF·py	12.25	-28.12	-0.15	-8.11	-2.12
9HF·py	12.25	-29.04	-0.17	-8.10	-2.13
1HF·TEA	9.12	-26.98	0.00	-7.77	0.29
2HF·TEA	10.28	-28.62	0.00	-8.78	0.16
3HF·TEA	11.06	-28.32	0.00	-9.32	0.04
4HF·TEA	11.52	-29.42	0.00	-9.47	0.05
5HF·TEA	11.72	-29.81	0.00	-9.46	0.00

Table S36: The calculated nucleophilicities using DFT/B3LYP-D4/def2-TZVPPD+CPCM(high) for *n*HF·base clusters when using local descriptors.

***n*HF·base**	**N**	**N'**	**N''**	**N'''**
1HF·py	-11.14	0.72	1.80	1.60
2HF·py	-10.40	0.77	1.93	1.71
3HF·py	-11.11	0.72	1.80	1.61
4HF·py	-11.58	0.69	1.73	1.54
5HF·py	-11.82	0.68	1.70	1.51
6HF·py	-12.04	0.68	1.67	1.49
7HF·py	-12.19	0.68	1.66	1.48
8HF·py	-12.25	0.68	1.65	1.48
9HF·py	-12.25	0.68	1.66	1.48
1HF·TEA	-9.12	0.88	2.19	1.95
2HF·TEA	-10.28	0.78	1.95	1.73
3HF·TEA	-11.06	0.72	1.81	1.61
4HF·TEA	-11.52	0.69	1.74	1.54
5HF·TEA	-11.72	0.68	1.71	1.52

Table S37: The calculated nucleophilicities using DFT/B3LYP-D4/def2-TZVPPD+CPCM(high) for *n*HF·base clusters when using global descriptors.

***n*HF·base**	**N**	**N'**	**N''**	**N'''**
1HF·py	-7.68	0.60	2.11	1.66
2HF·py	-8.20	0.46	1.81	1.36
3HF·py	-8.28	0.44	1.77	1.32
4HF·py	-8.31	0.43	1.75	1.31
5HF·py	-8.31	0.43	1.75	1.30
6HF·py	-8.32	0.42	1.73	1.29
7HF·py	-8.32	0.43	1.74	1.29
8HF·py	-8.11	0.46	1.82	1.37
9HF·py	-8.10	0.46	1.82	1.37
1HF·TEA	-7.77	1.15	2.67	2.43
2HF·TEA	-8.78	0.96	2.32	2.09
3HF·TEA	-9.32	0.87	2.15	1.92
4HF·TEA	-9.47	0.86	2.12	1.89
5HF·TEA	-9.46	0.85	2.11	1.88

Table S38: The calculated local and global properties used to calculate the nucleophilicities using DFT/BP86/def2-TZVPPD+CPCM(high) for *n*HF·base clusters. $\bar{I}(\mathbf{r})$: Average local ionisation energy; $\overline{A_E}(\mathbf{r})$: Local electron affinity; $E_{att}(\mathbf{r})$: Local electron attachment energy; HOMO: Highest occupied molecular orbital; LUMO: Lowest unoccupied molecular orbital

	Local properties on fluoride anion in *n*HF·base cluster			Global properties for *n*HF·base cluster	
***n*HF·base**	**$\bar{I}(r)$ / eV**	**$\overline{A_E}(r)$ / eV**	**$E_{att}(r)$ / eV**	**HOMO / eV**	**LUMO / eV**
1HF·py	9.24	-24.14	-0.01	-7.13	-2.46
2HF·py	9.03	-26.01	-0.05	-7.56	-3.00
3HF·py	9.67	-27.35	-0.20	-7.67	-3.14
4HF·py	10.09	-28.71	-0.31	-7.71	-3.20
5HF·py	10.33	-29.09	-0.39	-7.73	-3.22
6HF·py	10.56	-29.26	-0.34	-7.53	-2.99
7HF·py	10.71	-29.64	-0.35	-7.55	-3.02
8HF·py	10.77	-29.29	-0.42	-7.77	-3.28
9HF·py	10.81	-29.31	-0.40	-7.78	-3.28
1HF·TEA	8.04	-26.61	0.00	-6.62	0.05
2HF·TEA	8.84	-28.20	-0.07	-7.52	-7.67
3HF·TEA	9.64	-30.15	-0.27	-8.14	-0.28
4HF·TEA	10.03	-30.69	-0.31	-8.41	-0.27
5HF·TEA	10.24	-30.88	-0.44	-8.43	-0.34

Table S39: The calculated nucleophilicities using DFT/BP86/def2-TZVPPD+CPCM(high) for *n*HF·base clusters when using local descriptors.

***n*HF·base**	**N**	**N'**	**N''**	**N'''**
1HF·py	-9.24	0.87	2.17	1.93
2HF·py	-9.03	0.90	2.23	1.99
3HF·py	-9.67	0.88	2.11	1.90
4HF·py	-10.09	0.87	2.04	1.85
5HF·py	-10.33	0.87	2.01	1.83
6HF·py	-10.56	0.84	1.96	1.78
7HF·py	-10.71	0.82	1.93	1.75
8HF·py	-10.77	0.83	1.93	1.76
9HF·py	-10.81	0.83	1.92	1.75
1HF·TEA	-8.04	1.01	2.49	2.21
2HF·TEA	-8.84	0.97	2.28	2.04
3HF·TEA	-9.64	0.91	2.13	1.93
4HF·TEA	-10.03	0.90	2.06	1.87
5HF·TEA	-10.24	0.81	2.04	1.86

Table S40: The calculated nucleophilicities using DFT/BP86/def2-TZVPPD+CPCM(high) for *n*HF·base clusters when using global descriptors.

***n*HF·base**	**N**	**N'**	**N''**	**N'''**
1HF·py	-7.13	0.41	1.84	0.13
2HF·py	-7.56	0.33	1.59	0.11
3HF·py	-7.67	0.31	1.54	0.10
4HF·py	-7.71	0.30	1.52	0.09
5HF·py	-7.73	0.30	1.51	0.09
6HF·py	-7.53	0.33	1.60	0.09
7HF·py	-7.55	0.32	1.59	0.09
8HF·py	-7.77	0.29	1.49	0.09
9HF·py	-7.78	0.29	1.49	0.09
1HF·TEA	-6.62	1.24	3.05	0.17
2HF·TEA	-7.52	-0.01	-0.05	0.00
3HF·TEA	-8.14	0.89	2.37	0.15
4HF·TEA	-8.41	0.86	2.30	0.15
5HF·TEA	-8.43	0.84	2.28	0.15

Table S41: The calculated local and global properties used to calculate the nucleophilicities using DFT/cam-B3LYP/def2-TZVPPD+CPCM(high) for *n*HF·base clusters. $\overline{I}(\mathbf{r})$: Average local ionisation energy; $\overline{A_E}(\mathbf{r})$: Local electron affinity; $E_{att}(\mathbf{r})$: Local electron attachment energy; HOMO: Highest occupied molecular orbital; LUMO: Lowest unoccupied molecular orbital

	Local properties on fluoride anion in *n*HF·base cluster			Global properties for *n*HF·base cluster	
***n*HF·base**	**$\overline{I}$(r) / eV**	**$\overline{A_E}$(r) / eV**	**E_{att}(r) / eV**	**HOMO / eV**	**LUMO / eV**
1HF·py	13.17	-26.00	0.00	-9.24	-0.23
2HF·py	12.51	-27.27	0.00	-9.75	-0.88
3HF·py	13.25	-28.94	-0.01	-9.84	-0.99
4HF·py	13.69	-29.76	-0.01	-9.85	-1.02
5HF·py	13.92	-30.40	0.00	-9.89	-1.06
6HF·py	14.19	-28.97	0.00	-9.65	-0.90
7HF·py	14.35	-29.27	0.00	-9.91	-1.11
8HF·py	14.44	-30.59	0.00	-9.65	-0.92
9HF·py	14.46	-30.37	-0.01	-9.90	-1.09
1HF·TEA	11.16	-28.27	0.00	-9.58	1.00
2HF·TEA	12.36	-29.92	0.00	-10.63	0.89
3HF·TEA	13.20	-30.97	0.00	-11.16	0.76
4HF·TEA	13.64	-31.82	0.00	-11.21	0.75
5HF·TEA	13.80	-30.86	0.00	-11.22	0.73

Table S42: The calculated nucleophilicities using DFT/cam-B3LYP/def2-TZVPPD+CPCM(high) for *n*HF·base clusters when using local descriptors.

***n*HF·base**	**N**	**N'**	**N''**	**N'''**
1HF·py	-13.17	0.61	1.52	1.35
2HF·py	-12.51	0.64	1.60	1.42
3HF·py	-13.25	0.60	1.51	1.34
4HF·py	-13.69	0.59	1.46	1.30
5HF·py	-13.92	0.57	1.44	1.28
6HF·py	-14.19	0.56	1.41	1.25
7HF·py	-14.35	0.56	1.39	1.24
8HF·py	-14.44	0.55	1.39	1.23
9HF·py	-14.46	0.55	1.38	1.23
1HF·TEA	-11.16	0.72	1.79	1.59
2HF·TEA	-12.36	0.65	1.62	1.44
3HF·TEA	-13.20	0.61	1.51	1.35
4HF·TEA	-13.64	0.59	1.47	1.30
5HF·TEA	-13.80	0.58	1.45	1.29

Table S43: The calculated nucleophilicities using DFT/cam-B3LYP/def2-TZVPPD+CPCM(high) for *n*HF·base clusters when using global descriptors.

***n*HF·base**	**N**	**N'**	**N''**	**N'''**
1HF·py	-9.24	0.80	2.11	1.85
2HF·py	-9.75	0.63	1.87	1.56
3HF·py	-9.84	0.60	1.83	1.52
4HF·py	-9.85	0.60	1.82	1.51
5HF·py	-9.89	0.59	1.81	1.50
6HF·py	-9.65	0.63	1.88	1.57
7HF·py	-9.91	0.58	1.79	1.48
8HF·py	-9.65	0.62	1.87	1.56
9HF·py	-9.90	0.58	1.80	1.49
1HF·TEA	-9.58	1.15	2.31	2.20
2HF·TEA	-10.63	0.97	2.04	1.92
3HF·TEA	-11.16	0.88	1.91	1.78
4HF·TEA	-11.21	0.88	1.90	1.77
5HF·TEA	-11.22	0.87	1.90	1.77

Table S44: The calculated local and global properties used to calculate the nucleophilicities using DFT/cam-B3LYP-D4/def2-TZVPPD+CPCM(high) for *n*HF·base clusters. $\bar{I}(\mathbf{r})$: Average local ionisation energy; $\overline{A_E}(\mathbf{r})$: Local electron affinity; $E_{att}(\mathbf{r})$: Local electron attachment energy; HOMO: Highest occupied molecular orbital; LUMO: Lowest unoccupied molecular orbital

	Local properties on fluoride anion in *n*HF·base cluster			Global properties for *n*HF·base cluster	
***n*HF·base**	**$\bar{I}(r)$ / eV**	**$\overline{A_E}(r)$ / eV**	**$E_{att}(r)$ / eV**	**HOMO / eV**	**LUMO / eV**
1HF·py	13.18	-26.01	0.00	-9.24	-0.22
2HF·py	12.53	-27.50	0.00	-9.75	-0.87
3HF·py	13.27	-29.00	-0.01	-9.84	-0.99
4HF·py	13.75	-28.27	-0.01	-9.84	-1.01
5HF·py	14.01	-28.97	0.00	-9.86	-1.04
6HF·py	14.26	-28.88	0.00	-9.86	-1.07
7HF·py	14.42	-28.74	0.00	-9.85	-1.05
8HF·py	14.52	-29.26	0.00	-9.86	-1.06
9HF·py	14.54	-30.03	0.00	-9.87	-1.08
1HF·TEA	11.19	-28.33	0.00	-9.57	1.00
2HF·TEA	12.41	-29.81	0.00	-10.62	0.90
3HF·TEA	13.22	-29.52	0.00	-11.14	0.80
4HF·TEA	13.69	-30.62	0.00	-11.19	0.81
5HF·TEA	13.92	-30.62	0.00	-11.17	0.77

Table S45: The calculated nucleophilicities using DFT/cam-B3LYP-D4/def2-TZVPPD+CPCM(high) for *n*HF·base clusters when using local descriptors.

***n*HF·base**	**N**	**N’**	**N”**	**N’’’**
1HF·py	-13.18	0.61	1.52	1.35
2HF·py	-12.53	0.64	1.60	1.42
3HF·py	-13.27	0.60	1.51	1.34
4HF·py	-13.75	0.58	1.46	1.29
5HF·py	-14.01	0.57	1.43	1.27
6HF·py	-14.26	0.56	1.40	1.25
7HF·py	-14.42	0.55	1.39	1.23
8HF·py	-14.52	0.55	1.38	1.22
9HF·py	-14.54	0.55	1.38	1.22
1HF·TEA	-11.19	0.72	1.79	1.59
2HF·TEA	-12.41	0.64	1.61	1.43
3HF·TEA	-13.22	0.61	1.51	1.34
4HF·TEA	-13.69	0.58	1.46	1.30
5HF·TEA	-13.92	0.57	1.44	1.28

Table S46: The calculated nucleophilicities using DFT/cam-B3LYP-D4/def2-TZVPPD+CPCM(high) for *n*HF·base clusters when using global descriptors.

***n*HF·base**	**N**	**N'**	**N''**	**N'''**
1HF·py	-9.24	0.81	2.11	1.85
2HF·py	-9.75	0.63	1.87	1.56
3HF·py	-9.84	0.60	1.83	1.52
4HF·py	-9.84	0.60	1.82	1.51
5HF·py	-9.86	0.59	1.81	1.51
6HF·py	-9.86	0.59	1.81	1.50
7HF·py	-9.85	0.59	1.81	1.50
8HF·py	-9.86	0.59	1.81	1.50
9HF·py	-9.87	0.59	1.80	1.49
1HF·TEA	-9.57	1.15	2.31	2.20
2HF·TEA	-10.62	0.98	2.04	1.92
3HF·TEA	-11.14	0.89	1.92	1.80
4HF·TEA	-11.19	0.89	1.92	1.79
5HF·TEA	-11.17	0.88	1.91	1.78

Table S47: The calculated local and global properties used to calculate the nucleophilicities using DFT/LC-PBE/def2-TZVPPD+CPCM(high) for *n*HF·base clusters. $\bar{I}(\mathbf{r})$: Average local ionisation energy; $\overline{A_E}(\mathbf{r})$: Local electron affinity; $E_{att}(\mathbf{r})$: Local electron attachment energy; HOMO: Highest occupied molecular orbital; LUMO: Lowest unoccupied molecular orbital

	Local properties on fluoride anion in *n*HF·base cluster			Global properties for *n*HF·base cluster	
***n*HF·base**	**$\bar{I}$(r) / eV**	**$\overline{A_E}$(r) / eV**	**E_{att}(r) / eV**	**HOMO / eV**	**LUMO / eV**
1HF·py	15.15	-27.56	0.00	-10.95	0.78
2HF·py	14.97	-29.65	0.00	-11.39	0.22
3HF·py	15.72	-31.18	0.00	-11.50	0.08
4HF·py	16.18	-31.38	0.00	-11.54	0.02
5HF·py	16.46	-32.80	0.00	-11.56	0.00
6HF·py	16.79	-31.02	0.00	-11.57	-0.03
7HF·py	16.94	-31.86	0.00	-11.56	-0.03
8HF·py	17.03	-32.72	0.00	-11.27	0.04
9HF·py	17.06	-33.38	0.00	-11.56	-0.03
1HF·TEA	13.86	-29.84	0.00	-11.15	1.85
2HF·TEA	14.82	-31.86	0.00	-12.32	1.77
3HF·TEA	15.69	-33.10	0.00	-12.87	1.67
4HF·TEA	16.16	-33.28	0.00	-12.84	1.70
5HF·TEA	16.45	-32.97	0.00	-12.84	1.69

Table S48: The calculated nucleophilicities using DFT/LC-PBE/def2-TZVPPD+CPCM(high) for *n*HF·base clusters when using local descriptors.

***n*HF·base**	**N**	**N'**	**N''**	**N'''**
1HF·py	-15.15	0.53	1.32	1.17
2HF·py	-14.97	0.53	1.34	1.19
3HF·py	-15.72	0.51	1.27	1.13
4HF·py	-16.18	0.49	1.24	1.10
5HF·py	-16.46	0.49	1.22	1.08
6HF·py	-16.79	0.48	1.19	1.06
7HF·py	-16.94	0.47	1.18	1.05
8HF·py	-17.03	0.47	1.17	1.04
9HF·py	-17.06	0.47	1.17	1.04
1HF·TEA	-13.86	0.58	1.44	1.28
2HF·TEA	-14.82	0.54	1.35	1.20
3HF·TEA	-15.69	0.51	1.28	1.13
4HF·TEA	-16.16	0.50	1.24	1.10
5HF·TEA	-16.45	0.49	1.22	1.08

Table S49: The calculated nucleophilicities using DFT/LC-PBE/def2-TZVPPD+CPCM(high) for *n*HF·base clusters when using global descriptors.

***n*HF·base**	**N**	**N'**	**N''**	**N'''**
1HF·py	-10.95	0.91	1.96	1.83
2HF·py	-11.39	0.74	1.79	1.61
3HF·py	-11.50	0.71	1.75	1.56
4HF·py	-11.54	0.70	1.74	1.55
5HF·py	-11.56	0.69	1.73	1.54
6HF·py	-11.57	0.69	1.72	1.53
7HF·py	-11.56	0.69	1.73	1.53
8HF·py	-11.27	0.72	1.78	1.59
9HF·py	-11.56	0.69	1.73	1.53
1HF·TEA	-11.15	1.20	2.09	2.08
2HF·TEA	-12.32	1.01	1.86	1.82
3HF·TEA	-12.87	0.93	1.76	1.71
4HF·TEA	-12.84	0.94	1.76	1.72
5HF·TEA	-12.84	0.94	1.76	1.71

Table S50: The calculated local and global properties used to calculate the nucleophilicities using DFT/M06-2X/def2-TZVPPD+CPCM(high) for *n*HF·base clusters. $\bar{I}(\mathbf{r})$: Average local ionisation energy; $\overline{A_E}(\mathbf{r})$: Local electron affinity; $E_{att}(\mathbf{r})$: Local electron attachment energy; HOMO: Highest occupied molecular orbital; LUMO: Lowest unoccupied molecular orbital

	Local properties on fluoride anion in *n*HF·base cluster			Global properties for *n*HF·base cluster	
***n*HF·base**	**$\bar{I}$(r) / eV**	**$\overline{A_E}$(r) / eV**	**E_{att}(r) / eV**	**HOMO / eV**	**LUMO / eV**
1HF·py	14.05	-26.29	0.00	-9.10	-0.45
2HF·py	13.32	-27.68	-0.01	-9.61	-1.09
3HF·py	13.94	-29.14	-0.02	-9.74	-1.26
4HF·py	14.43	-28.57	-0.01	-9.79	-1.33
5HF·py	14.72	-29.70	0.00	-9.77	-1.35
6HF·py	15.04	-29.88	0.00	-9.75	-1.35
7HF·py	15.17	-29.45	0.00	-9.72	-1.31
8HF·py	15.37	-30.31	0.00	-9.79	-1.38
9HF·py	15.39	-30.08	0.00	-9.76	-1.35
1HF·TEA	13.24	-29.08	0.00	-9.08	0.54
2HF·TEA	13.07	-29.56	0.00	-10.79	0.43
3HF·TEA	13.89	-30.42	0.00	-11.27	0.35
4HF·TEA	14.38	-31.26	0.00	-11.14	0.34
5HF·TEA	14.64	-31.32	0.00	-11.14	0.30

Table S51: The calculated nucleophilicities using DFT/M06-2X/def2-TZVPPD+CPCM(high) for *n*HF·base clusters when using local descriptors.

***n*HF·base**	**N**	**N’**	**N’’**	**N’’’**
1HF·py	-14.05	0.57	1.42	1.27
2HF·py	-13.32	0.60	1.50	1.34
3HF·py	-13.94	0.58	1.44	1.28
4HF·py	-14.43	0.55	1.39	1.23
5HF·py	-14.72	0.54	1.36	1.21
6HF·py	-15.04	0.53	1.33	1.18
7HF·py	-15.17	0.53	1.32	1.17
8HF·py	-15.37	0.52	1.30	1.16
9HF·py	-15.39	0.52	1.30	1.16
1HF·TEA	-13.24	0.60	1.51	1.34
2HF·TEA	-13.07	0.61	1.53	1.36
3HF·TEA	-13.89	0.58	1.44	1.28
4HF·TEA	-14.38	0.56	1.39	1.24
5HF·TEA	-14.64	0.55	1.37	1.21

Table S52: The calculated nucleophilicities using DFT/M06-2X/def2-TZVPPD+CPCM(high) for *n*HF·base clusters when using global descriptors.

***n*HF·base**	**N**	**N'**	**N''**	**N'''**
1HF·py	-9.10	0.76	2.09	1.80
2HF·py	-9.61	0.60	1.85	1.52
3HF·py	-9.74	0.56	1.79	1.46
4HF·py	-9.79	0.55	1.76	1.44
5HF·py	-9.77	0.55	1.77	1.43
6HF·py	-9.75	0.55	1.77	1.44
7HF·py	-9.72	0.55	1.78	1.45
8HF·py	-9.79	0.54	1.76	1.42
9HF·py	-9.76	0.54	1.76	1.43
1HF·TEA	-9.08	1.05	2.33	2.16
2HF·TEA	-10.79	0.84	1.93	1.76
3HF·TEA	-11.27	0.78	1.83	1.66
4HF·TEA	-11.14	0.79	1.85	1.68
5HF·TEA	-11.14	0.78	1.84	1.67

Table S53: The calculated local and global properties used to calculate the nucleophilicities using DFT/RevDSD-PBEP86-D4/def2-TZVPPD+CPCM(high) for *n*HF·base clusters. $\bar{I}(\mathbf{r})$: Average local ionisation energy; $\overline{A_E}(\mathbf{r})$: Local electron affinity; $E_{att}(\mathbf{r})$: Local electron attachment energy; HOMO: Highest occupied molecular orbital; LUMO: Lowest unoccupied molecular orbital

	Local properties on fluoride anion in *n*HF·base cluster			Global properties for *n*HF·base cluster	
***n*HF·base**	**$\bar{I}$(r) / eV**	**$\overline{A_E}$(r) / eV**	**E_{att}(r) / eV**	**HOMO / eV**	**LUMO / eV**
1HF·py	15.30	-28.05	0.00	-9.08	0.74
2HF·py	14.11	-29.24	0.00	-9.62	0.07
3HF·py	14.85	-31.05	0.00	-9.71	-0.05
4HF·py	15.31	-29.70	0.00	-9.73	-0.08
5HF·py	15.53	-30.81	0.00	-9.74	-0.11
6HF·py	15.79	-30.90	0.00	-9.76	-0.16
7HF·py	15.95	-30.75	0.00	-9.73	-0.13
8HF·py	16.02	-31.44	0.00	-9.76	-0.15
9HF·py	16.06	-31.95	0.00	-9.79	-0.18
1HF·TEA	12.74	-29.85	0.00	-10.51	1.42
2HF·TEA	13.94	-31.29	0.00	-11.56	1.35
3HF·TEA	14.74	-31.30	0.00	-11.97	1.27
4HF·TEA	15.23	-32.42	0.00	-11.88	1.29
5HF·TEA	15.43	-32.81	0.00	-11.86	1.26

Table S54: The calculated nucleophilicities using DFT/RevDSD-PBEP86-D4/def2-TZVPPD+CPCM(high) for *n*HF·base clusters when using local descriptors.

***n*HF·base**	**N**	**N'**	**N''**	**N'''**
1HF·py	-15.30	0.52	1.31	1.16
2HF·py	-14.11	0.57	1.42	1.26
3HF·py	-14.85	0.54	1.35	1.20
4HF·py	-15.31	0.52	1.31	1.16
5HF·py	-15.53	0.52	1.29	1.14
6HF·py	-15.79	0.51	1.27	1.13
7HF·py	-15.95	0.50	1.25	1.11
8HF·py	-16.02	0.50	1.25	1.11
9HF·py	-16.06	0.50	1.25	1.11
1HF·TEA	-12.74	0.63	1.57	1.40
2HF·TEA	-13.94	0.57	1.43	1.28
3HF·TEA	-14.74	0.54	1.36	1.21
4HF·TEA	-15.23	0.53	1.31	1.17
5HF·TEA	-15.43	0.52	1.30	1.15

Table S55: The calculated nucleophilicities using DFT/RevDSD-PBEP86-D4/def2-TZVPPD+CPCM(high) for *n*HF·base clusters when using global descriptors.

***n*HF·base**	**N**	**N'**	**N''**	**N'''**
1HF·py	-9.08	1.13	2.38	2.24
2HF·py	-9.62	0.85	2.09	1.87
3HF·py	-9.71	0.81	2.05	1.82
4HF·py	-9.73	0.80	2.04	1.80
5HF·py	-9.74	0.79	2.03	1.79
6HF·py	-9.76	0.78	2.01	1.77
7HF·py	-9.73	0.79	2.03	1.78
8HF·py	-9.76	0.78	2.02	1.78
9HF·py	-9.79	0.77	2.01	1.76
1HF·TEA	-10.51	1.15	2.16	2.10
2HF·TEA	-11.56	0.99	1.93	1.86
3HF·TEA	-11.97	0.93	1.85	1.77
4HF·TEA	-11.88	0.94	1.87	1.79
5HF·TEA	-11.86	0.93	1.87	1.78

Table S56: The calculated local and global properties used to calculate the nucleophilicities using DFT/RevPBE/def2-TZVPPD+CPCM(high) for *n*HF·base clusters. $\bar{I}(\mathbf{r})$: Average local ionisation energy; $\overline{A_E}(\mathbf{r})$: Local electron affinity; $E_{att}(\mathbf{r})$: Local electron attachment energy; HOMO: Highest occupied molecular orbital; LUMO: Lowest unoccupied molecular orbital

	Local properties on fluoride anion in *n*HF·base cluster			Global properties for *n*HF·base cluster	
***n*HF·base**	**$\bar{I}$(r) / eV**	**$\overline{A_E}$(r) / eV**	**E_{att}(r) / eV**	**HOMO / eV**	**LUMO / eV**
1HF·py	9.13	-24.14	-0.12	-6.95	-2.29
2HF·py	8.67	-25.69	-0.20	-7.42	-2.89
3HF·py	9.25	-26.84	-0.41	-7.52	-3.02
4HF·py	9.70	-27.07	-0.49	-7.57	-3.07
5HF·py	9.91	-27.28	-0.58	-7.59	-3.10
6HF·py	10.10	-27.53	-0.43	-7.62	-3.15
7HF·py	10.23	-27.33	-0.47	-7.39	-2.86
8HF·py	10.29	-27.61	-0.55	-7.63	-3.16
9HF·py	10.28	-28.56	-0.46	-7.64	-3.17
1HF·TEA	7.64	-26.40	-0.09	-6.44	-0.15
2HF·TEA	8.46	-27.84	-0.20	-7.30	-0.31
3HF·TEA	9.21	-28.21	-0.41	-7.87	-0.46
4HF·TEA	9.63	-30.01	-0.48	-8.22	-0.48
5HF·TEA	9.77	-29.12	-0.48	-8.32	-0.55

Table S57: The calculated nucleophilicities using DFT/RevPBE/def2-TZVPPD+CPCM(high) for *n*HF·base clusters when using local descriptors.

***n*HF·base**	**N**	**N'**	**N''**	**N'''**
1HF·py	-9.13	0.91	2.22	1.99
2HF·py	-8.67	0.99	2.36	2.13
3HF·py	-9.25	0.99	2.26	2.07
4HF·py	-9.70	0.96	2.17	1.99
5HF·py	-9.91	0.96	2.14	1.97
6HF·py	-10.10	0.90	2.06	1.89
7HF·py	-10.23	0.90	2.04	1.87
8HF·py	-10.29	0.91	2.05	1.89
9HF·py	-10.28	0.89	2.03	1.86
1HF·TEA	-7.64	1.08	2.65	2.37
2HF·TEA	-8.46	1.01	2.42	2.18
3HF·TEA	-9.21	0.99	2.27	2.08
4HF·TEA	-9.63	0.97	2.18	2.00
5HF·TEA	-9.77	0.95	2.15	1.97

Table S58: The calculated nucleophilicities using DFT/RevPBE/def2-TZVPPD+CPCM(high) for *n*HF·base clusters when using global descriptors.

***n*HF·base**	**N**	**N'**	**N''**	**N'''**
1HF·py	-6.95	0.44	1.93	1.39
2HF·py	-7.42	0.34	1.65	1.15
3HF·py	-7.52	0.32	1.59	1.10
4HF·py	-7.57	0.32	1.57	1.08
5HF·py	-7.59	0.31	1.56	1.07
6HF·py	-7.62	0.31	1.54	1.06
7HF·py	-7.39	0.35	1.66	1.16
8HF·py	-7.63	0.31	1.53	1.05
9HF·py	-7.64	0.31	1.53	1.05
1HF·TEA	-6.44	1.16	3.03	2.65
2HF·TEA	-7.30	0.97	2.63	2.27
3HF·TEA	-7.87	0.86	2.39	2.05
4HF·TEA	-8.22	0.82	2.29	1.96
5HF·TEA	-8.32	0.79	2.25	1.91

Table S59: The calculated local and global properties used to calculate the nucleophilicities using DFT/RevPBE0/def2-TZVPPD+CPCM(high) for *n*HF·base clusters. $\bar{I}(\mathbf{r})$: Average local ionisation energy; $\overline{A_E}(\mathbf{r})$: Local electron affinity; $E_{att}(\mathbf{r})$: Local electron attachment energy; HOMO: Highest occupied molecular orbital; LUMO: Lowest unoccupied molecular orbital

	Local properties on fluoride anion in *n*HF·base cluster			Global properties for *n*HF·base cluster	
***n*HF·base**	**$\bar{I}$(r) / eV**	**$\overline{A_E}$(r) / eV**	**E_{att}(r) / eV**	**HOMO / eV**	**LUMO / eV**
1HF·py	11.67	-25.83	0.00	-7.92	-1.27
2HF·py	10.77	-26.91	-0.01	-8.46	-1.94
3HF·py	11.41	-28.72	-0.02	-8.56	-2.07
4HF·py	11.80	-29.62	-0.01	-8.59	-2.12
5HF·py	12.03	-30.01	-0.01	-8.61	-2.14
6HF·py	12.21	-30.29	-0.01	-8.39	-1.95
7HF·py	12.33	-30.04	-0.01	-8.41	-1.97
8HF·py	12.44	-29.58	-0.03	-8.67	-2.22
9HF·py	12.46	-29.95	-0.14	-8.38	-1.98
1HF·TEA	9.71	-27.69	0.00	-8.00	0.44
2HF·TEA	10.57	-29.38	0.00	-9.07	0.31
3HF·TEA	11.35	-29.93	0.00	-9.63	0.18
4HF·TEA	11.73	-31.89	0.00	-9.77	0.18
5HF·TEA	11.91	-31.97	0.00	-9.78	0.13

Table S60: The calculated nucleophilicities using DFT/RevPBE0/def2-TZVPPD+CPCM(high) for *n*HF·base clusters when using local descriptors.

***n*HF·base**	**N**	**N'**	**N''**	**N'''**
1HF·py	-11.67	0.69	1.71	1.52
2HF·py	-10.77	0.75	1.86	1.65
3HF·py	-11.41	0.70	1.76	1.56
4HF·py	-11.80	0.68	1.70	1.51
5HF·py	-12.03	0.67	1.66	1.48
6HF·py	-12.21	0.66	1.64	1.46
7HF·py	-12.33	0.65	1.62	1.45
8HF·py	-12.44	0.65	1.61	1.43
9HF·py	-12.46	0.66	1.62	1.45
1HF·TEA	-9.71	0.82	2.06	1.83
2HF·TEA	-10.57	0.76	1.89	1.68
3HF·TEA	-11.35	0.70	1.76	1.57
4HF·TEA	-11.73	0.68	1.70	1.52
5HF·TEA	-11.91	0.67	1.68	1.49

Table S61: The calculated nucleophilicities using DFT/RevPBE0/def2-TZVPPD+CPCM(high) for *n*HF·base clusters when using global descriptors.

***n*HF·base**	**N**	**N'**	**N''**	**N'''**
1HF·py	-7.92	0.63	2.12	1.70
2HF·py	-8.46	0.48	1.82	1.40
3HF·py	-8.56	0.46	1.77	1.35
4HF·py	-8.59	0.45	1.75	1.33
5HF·py	-8.61	0.45	1.75	1.32
6HF·py	-8.39	0.48	1.83	1.40
7HF·py	-8.41	0.48	1.82	1.39
8HF·py	-8.67	0.43	1.71	1.29
9HF·py	-8.38	0.48	1.82	1.39
1HF·TEA	-8.00	1.18	2.64	2.43
2HF·TEA	-9.07	0.98	2.28	2.08
3HF·TEA	-9.63	0.88	2.12	1.91
4HF·TEA	-9.77	0.86	2.08	1.88
5HF·TEA	-9.78	0.85	2.07	1.86

Table S62: The calculated local and global properties used to calculate the nucleophilicities using DFT/RevPBE0-D4/def2-TZVPPD+CPCM(high) for *n*HF·base clusters. $\bar{I}(\mathbf{r})$: Average local ionisation energy; $\overline{A_E}(\mathbf{r})$: Local electron affinity; $E_{att}(\mathbf{r})$: Local electron attachment energy; HOMO: Highest occupied molecular orbital; LUMO: Lowest unoccupied molecular orbital

	Local properties on fluoride anion in *n*HF·base cluster			Global properties for *n*HF·base cluster	
***n*HF·base**	**$\bar{I}(r)$ / eV**	**$\overline{A_E}(r)$ / eV**	**$E_{att}(r)$ / eV**	**HOMO / eV**	**LUMO / eV**
1HF·py	11.66	-25.83	0.00	-7.93	-1.26
2HF·py	10.81	-27.15	-0.01	-8.45	-1.92
3HF·py	11.46	-28.88	-0.02	-8.55	-2.05
4HF·py	11.92	-28.07	-0.01	-8.59	-2.10
5HF·py	12.15	-28.30	-0.01	-8.61	-2.12
6HF·py	12.38	-29.38	0.00	-8.39	-1.95
7HF·py	12.47	-28.48	0.00	-8.63	-2.18
8HF·py	12.55	-30.21	0.00	-8.39	-1.97
9HF·py	12.56	-29.51	-0.02	-8.63	-2.17
1HF·TEA	9.84	-27.63	0.00	-7.95	0.44
2HF·TEA	10.66	-29.39	0.00	-9.04	0.33
3HF·TEA	11.42	-29.26	0.00	-9.58	0.22
4HF·TEA	11.85	-30.29	0.00	-9.74	0.23
5HF·TEA	12.05	-30.42	0.00	-9.74	0.19

Table S63: The calculated nucleophilicities using DFT/RevPBE0-D4/def2-TZVPPD+CPCM(high) for *n*HF·base clusters when using local descriptors.

***n*HF·base**	**N**	**N'**	**N''**	**N'''**
1HF·py	-11.66	0.69	1.72	1.53
2HF·py	-10.81	0.74	1.85	1.65
3HF·py	-11.46	0.70	1.75	1.56
4HF·py	-11.92	0.67	1.68	1.49
5HF·py	-12.15	0.66	1.65	1.46
6HF·py	-12.38	0.65	1.62	1.44
7HF·py	-12.47	0.64	1.60	1.43
8HF·py	-12.55	0.64	1.59	1.42
9HF·py	-12.56	0.64	1.60	1.42
1HF·TEA	-9.84	0.81	2.03	1.81
2HF·TEA	-10.66	0.75	1.88	1.67
3HF·TEA	-11.42	0.70	1.75	1.56
4HF·TEA	-11.85	0.67	1.69	1.50
5HF·TEA	-12.05	0.66	1.66	1.48

Table S64: The calculated nucleophilicities using DFT/RevPBE0-D4/def2-TZVPPD+CPCM(high) for *n*HF·base clusters when using global descriptors.

***n*HF·base**	**N**	**N'**	**N''**	**N'''**
1HF·py	-7.93	0.63	2.12	1.70
2HF·py	-8.45	0.49	1.83	1.40
3HF·py	-8.55	0.46	1.78	1.36
4HF·py	-8.59	0.45	1.76	1.34
5HF·py	-8.61	0.45	1.75	1.33
6HF·py	-8.39	0.48	1.83	1.40
7HF·py	-8.63	0.44	1.73	1.31
8HF·py	-8.39	0.48	1.83	1.40
9HF·py	-8.63	0.44	1.74	1.31
1HF·TEA	-7.95	1.19	2.65	2.45
2HF·TEA	-9.04	0.99	2.29	2.09
3HF·TEA	-9.58	0.90	2.14	1.93
4HF·TEA	-9.74	0.88	2.10	1.90
5HF·TEA	-9.74	0.87	2.09	1.89

Table S65: The calculated local and global properties used to calculate the nucleophilicities using DFT/RevPBE38/def2-TZVPPD+CPCM(high) for *n*HF·base clusters. $\bar{I}(\mathbf{r})$: Average local ionisation energy; $\overline{A_E}(\mathbf{r})$: Local electron affinity; $E_{att}(\mathbf{r})$: Local electron attachment energy; HOMO: Highest occupied molecular orbital; LUMO: Lowest unoccupied molecular orbital

	Local properties on fluoride anion in *n*HF·base cluster			Global properties for *n*HF·base cluster	
***n*HF·base**	**$\bar{I}(r)$ / eV**	**$\overline{A_E}(r)$ / eV**	**$E_{att}(r)$ / eV**	**HOMO / eV**	**LUMO / eV**
1HF·py	12.94	-26.66	0.00	-8.37	-0.69
2HF·py	11.84	-27.70	-0.01	-8.97	-1.46
3HF·py	12.53	-29.59	-0.02	-9.07	-1.60
4HF·py	12.92	-30.79	-0.01	-9.11	-1.64
5HF·py	13.15	-31.00	-0.01	-9.13	-1.67
6HF·py	13.33	-31.11	0.00	-8.89	-1.52
7HF·py	13.46	-31.13	0.00	-8.91	-1.54
8HF·py	13.53	-31.02	-0.02	-9.19	-1.75
9HF·py	13.59	-30.75	-0.04	-9.19	-1.76
1HF·TEA	12.06	-29.20	0.00	-8.36	0.73
2HF·TEA	11.64	-30.16	0.00	-9.93	0.60
3HF·TEA	12.45	-32.01	0.00	-10.46	0.47
4HF·TEA	12.84	-32.79	0.00	-10.50	0.48
5HF·TEA	13.03	-31.44	0.00	-10.51	0.44

Table S66: The calculated nucleophilicities using DFT/RevPBE38/def2-TZVPPD+CPCM(high) for *n*HF·base clusters when using local descriptors.

***n*HF·base**	**N**	**N'**	**N''**	**N'''**
1HF·py	-12.94	0.62	1.55	1.37
2HF·py	-11.84	0.68	1.69	1.50
3HF·py	-12.53	0.64	1.60	1.42
4HF·py	-12.92	0.62	1.55	1.38
5HF·py	-13.15	0.61	1.52	1.35
6HF·py	-13.33	0.60	1.50	1.33
7HF·py	-13.46	0.59	1.49	1.32
8HF·py	-13.53	0.59	1.48	1.32
9HF·py	-13.59	0.59	1.48	1.32
1HF·TEA	-12.06	0.66	1.66	1.47
2HF·TEA	-11.64	0.69	1.72	1.53
3HF·TEA	-12.45	0.64	1.61	1.43
4HF·TEA	-12.84	0.62	1.56	1.38
5HF·TEA	-13.03	0.61	1.54	1.36

Table S67: The calculated nucleophilicities using DFT/RevPBE38/def2-TZVPPD+CPCM(high) for *n*HF·base clusters when using global descriptors.

***n*HF·base**	**N**	**N'**	**N''**	**N'''**
1HF·py	-8.37	-1.68	2.19	1.85
2HF·py	-8.97	-1.26	1.87	1.49
3HF·py	-9.07	-1.16	1.82	1.44
4HF·py	-9.11	-1.10	1.80	1.42
5HF·py	-9.13	-1.11	1.79	1.42
6HF·py	-8.89	-1.13	1.87	1.48
7HF·py	-8.91	-1.14	1.86	1.48
8HF·py	-9.19	-1.16	1.76	1.38
9HF·py	-9.19	-1.20	1.76	1.38
1HF·TEA	-8.36	-1.12	2.60	2.45
2HF·TEA	-9.93	-0.98	2.14	1.98
3HF·TEA	-10.46	-0.93	2.00	1.83
4HF·TEA	-10.50	-0.92	1.99	1.83
5HF·TEA	-10.51	-1.05	1.98	1.81

Table S68: The calculated local and global properties used to calculate the nucleophilicities using DFT/RevPBE38-D4/def2-TZVPPD+CPCM(high) for *n*HF·base clusters. $\bar{I}(\mathbf{r})$: Average local ionisation energy; $\overline{A_E}(\mathbf{r})$: Local electron affinity; $E_{att}(\mathbf{r})$: Local electron attachment energy; HOMO: Highest occupied molecular orbital; LUMO: Lowest unoccupied molecular orbital

	Local properties on fluoride anion in *n*HF·base cluster			Global properties for *n*HF·base cluster	
***n*HF·base**	**$\bar{I}(r)$ / eV**	**$\overline{A_E}(r)$ / eV**	**$E_{att}(r)$ / eV**	**HOMO / eV**	**LUMO / eV**
1HF·py	12.92	-26.65	0.00	-8.43	-0.76
2HF·py	11.89	-27.79	-0.01	-8.97	-1.45
3HF·py	12.56	-29.77	-0.02	-9.07	-1.58
4HF·py	13.01	-28.98	-0.01	-9.11	-1.63
5HF·py	13.25	-29.24	-0.01	-9.12	-1.65
6HF·py	13.49	-29.28	0.00	-8.89	-1.52
7HF·py	13.58	-29.36	0.00	-9.14	-1.71
8HF·py	13.62	-30.21	0.00	-9.15	-1.70
9HF·py	13.68	-30.64	-0.02	-9.14	-1.71
1HF·TEA	10.86	-28.26	0.00	-8.75	0.71
2HF·TEA	11.72	-30.12	0.00	-9.89	0.61
3HF·TEA	12.50	-30.04	0.00	-10.41	0.52
4HF·TEA	12.93	-31.08	0.00	-10.48	0.53
5HF·TEA	13.14	-31.27	0.00	-10.48	0.50

Table S69: The calculated nucleophilicities using DFT/RevPBE38-D4/def2-TZVPPD+CPCM(high) for *n*HF·base clusters when using local descriptors.

***n*HF·base**	**N**	**N’**	**N’’**	**N’’’**
1HF·py	-12.92	0.62	1.55	1.38
2HF·py	-11.89	0.67	1.68	1.50
3HF·py	-12.56	0.64	1.59	1.42
4HF·py	-13.01	0.62	1.54	1.37
5HF·py	-13.25	0.60	1.51	1.34
6HF·py	-13.49	0.59	1.48	1.32
7HF·py	-13.58	0.59	1.47	1.31
8HF·py	-13.62	0.59	1.47	1.31
9HF·py	-13.68	0.59	1.46	1.30
1HF·TEA	-10.86	0.74	1.84	1.64
2HF·TEA	-11.72	0.68	1.71	1.52
3HF·TEA	-12.50	0.64	1.60	1.42
4HF·TEA	-12.93	0.62	1.55	1.37
5HF·TEA	-13.14	0.61	1.52	1.35

Table S70: The calculated nucleophilicities using DFT/RevPBE38-D4/def2-TZVPPD+CPCM(high) for *n*HF·base clusters when using global descriptors.

***n*HF·base**	**N**	**N'**	**N''**	**N'''**
1HF·py	-8.43	0.73	2.16	1.81
2HF·py	-8.97	0.55	1.87	1.49
3HF·py	-9.07	0.53	1.82	1.45
4HF·py	-9.11	0.52	1.80	1.43
5HF·py	-9.12	0.51	1.79	1.42
6HF·py	-8.89	0.54	1.87	1.48
7HF·py	-9.14	0.50	1.78	1.40
8HF·py	-9.15	0.51	1.78	1.40
9HF·py	-9.14	0.51	1.78	1.40
1HF·TEA	-8.75	1.17	2.47	2.32
2HF·TEA	-9.89	0.98	2.15	1.99
3HF·TEA	-10.41	0.89	2.02	1.85
4HF·TEA	-10.48	0.89	2.01	1.84
5HF·TEA	-10.48	0.88	2.00	1.84

Table S71: The calculated local and global properties used to calculate the nucleophilicities using DFT/RevPBE-D4/def2-TZVPPD+CPCM(high) for *n*HF·base clusters. $\bar{I}(\mathbf{r})$: Average local ionisation energy; $\overline{A_E}(\mathbf{r})$: Local electron affinity; $E_{att}(\mathbf{r})$: Local electron attachment energy; HOMO: Highest occupied molecular orbital; LUMO: Lowest unoccupied molecular orbital

	Local properties on fluoride anion in *n*HF·base cluster			Global properties for *n*HF·base cluster	
***n*HF·base**	**$\bar{I}(r)$ / eV**	**$\overline{A_E}(r)$ / eV**	**$E_{att}(r)$ / eV**	**HOMO / eV**	**LUMO / eV**
1HF·py	9.17	-24.19	-0.12	-6.95	-2.27
2HF·py	8.74	-25.67	-0.19	-7.42	-2.86
3HF·py	9.31	-27.17	-0.42	-7.52	-2.99
4HF·py	9.83	-26.48	-0.40	-7.56	-3.04
5HF·py	10.07	-26.96	-0.45	-7.57	-3.07
6HF·py	10.32	-28.95	-0.43	-7.38	-2.83
7HF·py	10.35	-27.03	-0.43	-7.39	-2.86
8HF·py	10.43	-27.52	-0.53	-7.58	-3.10
9HF·py	10.39	-27.23	-0.44	-7.36	-2.86
1HF·TEA	7.80	-26.33	-0.09	-6.41	-0.15
2HF·TEA	8.59	-27.96	-0.18	-7.31	-0.28
3HF·TEA	9.33	-27.83	-0.32	-7.86	-0.41
4HF·TEA	9.77	-28.74	-0.35	-8.17	-0.41
5HF·TEA	9.97	-28.94	-0.39	-8.27	-0.47

Table S72: The calculated nucleophilicities using DFT/RevPBE-D4/def2-TZVPPD+CPCM(high) for *n*HF·base clusters when using local descriptors.

***n*HF·base**	**N**	**N'**	**N''**	**N'''**
1HF·py	-9.17	0.91	2.21	1.98
2HF·py	-8.74	0.98	2.34	2.11
3HF·py	-9.31	0.99	2.25	2.06
4HF·py	-9.83	0.92	2.12	1.93
5HF·py	-10.07	0.91	2.08	1.90
6HF·py	-10.32	0.88	2.02	1.84
7HF·py	-10.35	0.88	2.01	1.84
8HF·py	-10.43	0.90	2.02	1.85
9HF·py	-10.39	0.88	2.01	1.84
1HF·TEA	-7.80	1.06	2.59	2.32
2HF·TEA	-8.59	0.99	2.38	2.14
3HF·TEA	-9.33	0.95	2.22	2.02
4HF·TEA	-9.77	0.91	2.12	1.93
5HF·TEA	-9.97	0.90	2.08	1.90

Table S73: The calculated nucleophilicities using DFT/RevPBE-D4/def2-TZVPPD+CPCM(high) for *n*HF·base clusters when using global descriptors.

***n*HF·base**	**N**	**N’**	**N’’**	**N’’’**
1HF·py	-6.95	0.44	1.94	1.40
2HF·py	-7.42	0.35	1.66	1.16
3HF·py	-7.52	0.33	1.60	1.11
4HF·py	-7.56	0.32	1.58	1.09
5HF·py	-7.57	0.32	1.57	1.08
6HF·py	-7.38	0.35	1.67	1.17
7HF·py	-7.39	0.35	1.66	1.16
8HF·py	-7.58	0.31	1.56	1.07
9HF·py	-7.36	0.35	1.66	1.16
1HF·TEA	-6.41	1.17	3.05	2.67
2HF·TEA	-7.31	0.98	2.63	2.28
3HF·TEA	-7.86	0.87	2.41	2.07
4HF·TEA	-8.17	0.84	2.32	2.00
5HF·TEA	-8.27	0.82	2.28	1.95

Table S74: The calculated local and global properties used to calculate the nucleophilicities using DFT/ωB97X-D4/def2-TZVPPD+CPCM(high) for *n*HF·base clusters. $\bar{I}(\mathbf{r})$: Average local ionisation energy; $\overline{A_E}(\mathbf{r})$: Local electron affinity; $E_{att}(\mathbf{r})$: Local electron attachment energy; HOMO: Highest occupied molecular orbital; LUMO: Lowest unoccupied molecular orbital

	Local properties on fluoride anion in *n*HF·base cluster			Global properties for *n*HF·base cluster	
***n*HF·base**	**$\bar{I}(r)$ / eV**	**$\overline{A_E}(r)$ / eV**	**$E_{att}(r)$ / eV**	**HOMO / eV**	**LUMO / eV**
1HF·py	14.51	-27.53	0.00	-10.20	0.76
2HF·py	13.57	-28.55	0.00	-10.74	0.07
3HF·py	14.26	-29.98	0.00	-10.83	-0.04
4HF·py	14.69	-29.60	0.00	-10.85	-0.07
5HF·py	14.94	-30.03	0.00	-10.86	-0.10
6HF·py	15.17	-30.29	0.00	-10.89	-0.16
7HF·py	15.29	-29.99	0.00	-10.86	-0.14
8HF·py	15.36	-30.32	0.00	-10.65	0.05
9HF·py	15.41	-31.11	0.00	-10.90	-0.16
1HF·TEA	12.29	-29.58	0.00	-10.61	1.75
2HF·TEA	13.46	-30.84	0.00	-11.66	1.68
3HF·TEA	14.21	-30.84	0.00	-12.19	1.61
4HF·TEA	14.66	-31.68	0.00	-12.31	1.60
5HF·TEA	14.86	-32.13	0.00	-12.30	1.57

Table S75: The calculated nucleophilicities using DFT/ωB97X-D4/def2-TZVPPD+CPCM(high) for *n*HF·base clusters when using local descriptors.

***n*HF·base**	**N**	**N'**	**N''**	**N'''**
1HF·py	-14.51	0.55	1.38	1.23
2HF·py	-13.57	0.59	1.47	1.31
3HF·py	-14.26	0.56	1.40	1.25
4HF·py	-14.69	0.54	1.36	1.21
5HF·py	-14.94	0.54	1.34	1.19
6HF·py	-15.17	0.53	1.32	1.17
7HF·py	-15.29	0.52	1.31	1.16
8HF·py	-15.36	0.52	1.30	1.16
9HF·py	-15.41	0.52	1.30	1.15
1HF·TEA	-12.29	0.65	1.63	1.45
2HF·TEA	-13.46	0.59	1.49	1.32
3HF·TEA	-14.21	0.56	1.41	1.25
4HF·TEA	-14.66	0.55	1.36	1.21
5HF·TEA	-14.86	0.54	1.35	1.20

Table S76: The calculated nucleophilicities using DFT/ωB97X-D4/def2-TZVPPD+CPCM(high) for *n*HF·base clusters when using global descriptors.

***n*HF·base**	**N**	**N'**	**N''**	**N'''**
1HF·py	-10.20	0.98	2.11	1.97
2HF·py	-10.74	0.76	1.87	1.67
3HF·py	-10.83	0.73	1.84	1.63
4HF·py	-10.85	0.72	1.83	1.62
5HF·py	-10.86	0.72	1.83	1.61
6HF·py	-10.89	0.70	1.81	1.59
7HF·py	-10.86	0.71	1.82	1.60
8HF·py	-10.65	0.76	1.89	1.68
9HF·py	-10.90	0.70	1.81	1.59
1HF·TEA	-10.61	1.26	2.20	2.19
2HF·TEA	-11.66	1.07	1.96	1.92
3HF·TEA	-12.19	0.99	1.86	1.81
4HF·TEA	-12.31	0.97	1.84	1.78
5HF·TEA	-12.30	0.96	1.83	1.78

5.8 Basicity of the Fluoride Anion in *n*HF·py and *n*HF·TEA clusters

When calculating the basicity, both the proton affinity and the gas phase basicity were considered to determine trends. For the proton affinity, defined as the negative of the enthalpy change for the reaction of proton addition to the fluoride anion, **Equation S29** was used:

$$PA = H_{nHF\cdot base} + H_{proton} - H_{protonated\ nHF\cdot base}$$

Equation S29

Where H_x is the enthalpy of chemical species x. The enthalpy of the proton can be calculated using the ideal gas equation, and equates to $\frac{5}{2}RT$. When calculating the gas phase basicity, the entropy of the proton is also required. This can be calculated using the Sackur−Tetrode equation (**Equation S30**):

$$S(H^+) = R\ln\left(\frac{e^{\frac{5}{2}}k_BT}{p\Lambda^3}\right)$$

Equation S30

Where k_B is the Boltzmann constant, and Λ is the thermal de Broglie wavelength, calculated to be 1 Å under standard state conditions. If the enthalpy change is $\frac{5}{2}RT$ (1.48 kcal mol^{-1}), and the entropy is 26.02 cal mol^{-1} K^{-1}, then the Gibbs energy change is -6.28 kcal mol^{-1}. The gas phase basicity (GPB) is analogous to proton affinity, but instead considers the Gibbs energy of the chemical species involved in protonation (**Equation S31**):

$$GBP = G_{nHF\cdot base} + G_{proton} - G_{protonated\ nHF\cdot base}$$

Equation S31

To determine the geometries of the protonated *n*HF·base clusters, protons were placed 1 Å away from the fluoride anion in each of the clusters. Then an optimisation calculation was performed at the DFT/LC-PBE/def2-TZVPPD+CPCM(high) level of theory. As well as a full optimisation, a set of constrained optimisations were performed where all the *n*HF·base was frozen, but the proton added to the cluster was allowed to move freely. Thermodynamic data was obtained and is reported in **Table S77 – S79**. Both proton affinity and gas phase basicity calculations generally show that the fluoride basicity is greater when considering *n*HF·TEA compared to *n*HF·py for a given *n*. This can be explained by considering the charge associated with the fluoride anion. When considering *n*HF·TEA complexes, the fluoride anion interacts less with the protonated base compared to *n*HF·py (see NCI analysis). However, it should be noted that for *n*HF·py, the trend is not monotonic when considering values of *n* that are greater

than 4. This may be due to inappropriate modelling of solvent interactions with the *n*HF·base, resulting to anomalous behaviour. Ideally, the CPCM parameters derived for each *n*HF·base should be used, which may increase their accuracy. However, the general trend can be observed that as the number of HF molecules increase within the cluster, the basicity of the fluoride anion decreases, which aligns with experimental observation.

Table S77: Thermodynamic data obtained from the optimised *n*HF·base clusters at the DFT/LC-PBE/def2-TZVPPD+CPCM(high) level of theory.

*n*HF·base	Total thermal energy (E) / E_h	Total enthalpy term (H) / E_h	Total entropy term (S) / E_h	Final Gibbs energy / E_h
1HF·py	-347.70	-347.69	0.04	-347.73
2HF·py	-447.97	-447.97	0.04	-448.01
3HF·py	-548.25	-548.25	0.04	-548.30
4HF·py	-648.54	-648.54	0.05	-648.58
5HF·py	-748.82	-748.82	0.05	-748.87
6HF·py	-849.11	-849.11	0.06	-849.17
7HF·py	-949.39	-949.39	0.06	-949.45
8HF·py	-1049.68	-1049.67	0.07	-1049.74
9HF·py	-1149.95	-1149.95	0.07	-1150.02
1HF·TEA	-391.58	-391.58	0.05	-391.63
2HF·TEA	-491.87	-491.86	0.05	-491.91
3HF·TEA	-592.15	-592.15	0.05	-592.20
4HF·TEA	-692.44	-692.44	0.05	-692.49
5HF·TEA	-792.73	-792.73	0.07	-792.79

Table S78: Thermodynamic data obtained from the optimised protonated *n*HF·base clusters at the DFT/LC-PBE/def2-TZVPPD+CPCM(high) level of theory.

*n*HF·base	Total thermal energy (E) / E_h	Total enthalpy term (H) / E_h	Total entropy term (S) / E_h	Final Gibbs energy / E_h
1HF·py	-348.04	-348.04	0.04	-348.08
2HF·py	-448.32	-448.32	0.04	-448.37
3HF·py	-548.60	-548.60	0.05	-548.65
4HF·py	-648.88	-648.88	0.06	-648.93
5HF·py	-749.17	-749.17	0.06	-749.23
6HF·py	-849.44	-849.44	0.06	-849.50
7HF·py	-949.72	-949.72	0.07	-949.79
8HF·py	-1050.00	-1050.00	0.07	-1050.08
9HF·py	-1150.28	-1150.28	0.08	-1150.36
1HF·TEA	-391.94	-391.94	0.05	-391.99
2HF·TEA	-492.22	-492.22	0.05	-492.28
3HF·TEA	-592.51	-592.51	0.06	-592.56
4HF·TEA	-692.78	-692.78	0.06	-692.84
5HF·TEA	-793.05	-793.05	0.07	-793.12

Table S79: Thermodynamic data obtained from the constrained optimised protonated *n*HF·base clusters at the DFT/LC-PBE/def2-TZVPPD+CPCM(high) level of theory.

*n*HF·base	Total thermal energy (E) / E_h	Total enthalpy term (H) / E_h	Total entropy term (S) / E_h	Final Gibbs energy / E_h
1HF·py	-347.99	-347.99	0.04	-348.03
2HF·py	-448.27	-448.27	0.04	-448.31
3HF·py	-548.51	-548.51	0.04	-548.54
4HF·py	-648.77	-648.77	0.04	-648.81
5HF·py	-749.02	-749.02	0.04	-749.07
6HF·py	-849.34	-849.34	0.05	-849.39
7HF·py	-949.61	-949.61	0.05	-949.66
8HF·py	-1049.88	-1049.88	0.06	-1049.93
9HF·py	-1150.14	-1150.13	0.06	-1150.13
1HF·TEA	-391.92	-391.92	0.04	-391.96
2HF·TEA	-492.17	-492.17	0.05	-492.22
3HF·TEA	-592.41	-592.41	0.05	-592.46
4HF·TEA	-692.68	-692.68	0.05	-692.72
5HF·TEA	-792.95	-792.95	0.05	-793.00

Table S80: The calculated proton affinities (PA) and gas phase basicity (GPB) values for *n*HF·base based on the data shown in **Table S77 – S79**.

*n*HF·base	PA for full optimised protonated structure / kcal mol^{-1}	PA for constrained optimised protonated structure / kcal mol^{-1}	GPB for full optimised protonated structure / kcal mol^{-1}	GBP for constrained optimised protonated structure / kcal mol^{-1}
1HF·py	219.76	179.11	213.24	187.60
2HF·py	224.84	181.08	219.40	189.85
3HF·py	218.85	150.18	214.89	162.02
4HF·py	215.22	135.26	213.62	146.97
5HF·py	219.22	117.55	218.78	129.05
6HF·py	209.85	134.09	205.42	147.49
7HF·py	207.46	126.11	205.33	140.16
8HF·py	206.73	115.60	204.15	129.13
9HF·py	207.79	65.55	205.99	115.86
1HF·TEA	229.15	202.57	224.24	212.16
2HF·TEA	226.73	183.30	221.42	193.73
3HF·TEA	223.77	154.81	218.37	165.39
4HF·TEA	213.45	138.54	211.65	150.24
5HF·TEA	204.54	126.66	198.20	141.99

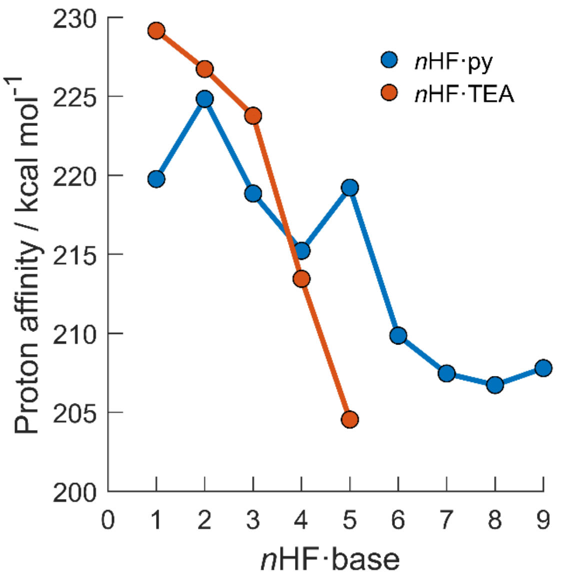

Figure S35: The calculated proton affinities for the fluoride anion in *n*HF·base when considering the fully optimised geometry for protonated *n*HF·base.

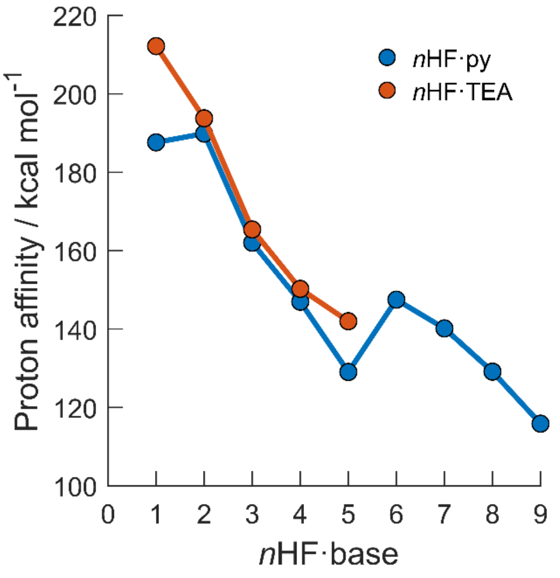

Figure S36: The calculated proton affinities for the fluoride anion in *n*HF·base when considering the constrained optimised geometry for protonated *n*HF·base.

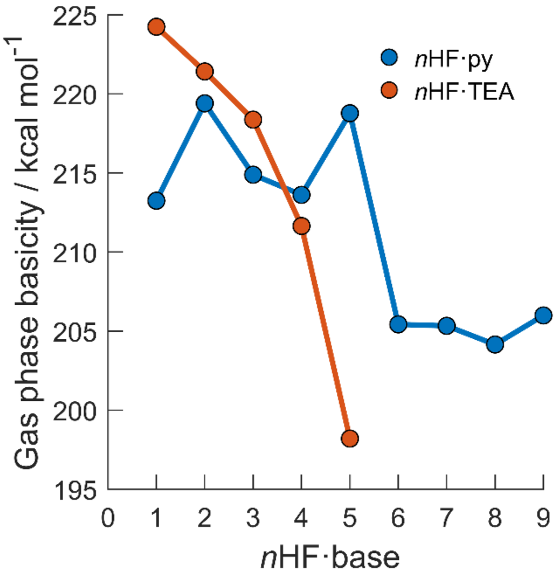

Figure S37: The calculated gas phase basicity for the fluoride anion in *n*HF·base when considering the fully optimised geometry for protonated *n*HF·base.

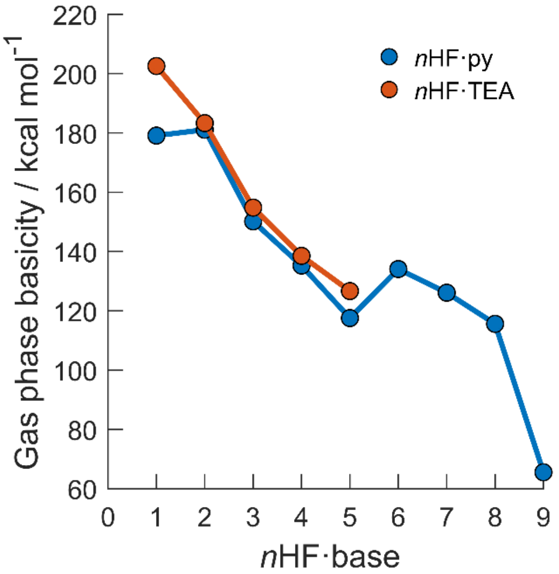

Figure S38: The calculated proton affinities for the fluoride anion in *n*HF·base when considering the constrained optimised geometry for protonated *n*HF·base.

6. VT-NMR for *n*HF·base Mixtures

For VT-NMR studies, 50 µL aliquots of the *n*HF·base mixture was mixed into 0.5 mL of DCM-d_2 in HDPE vials and mixed thoroughly. The solution was then transferred into an FEP NMR liner, which was then inserted into an NMR tube that contained 0.1 mL trichlorofluoromethane (internal reference for ^{19}F NMR). Both the ^{19}F and the ^{1}H NMR spectra were recorded using the Jeol ECS300 NMR spectrometer (300 MHz) between 293 K and 213 K in 20 K intervals. For each temperature, the NMR sample was left for three minutes to ensure that the sample was at equilibrium for the temperature being applied.

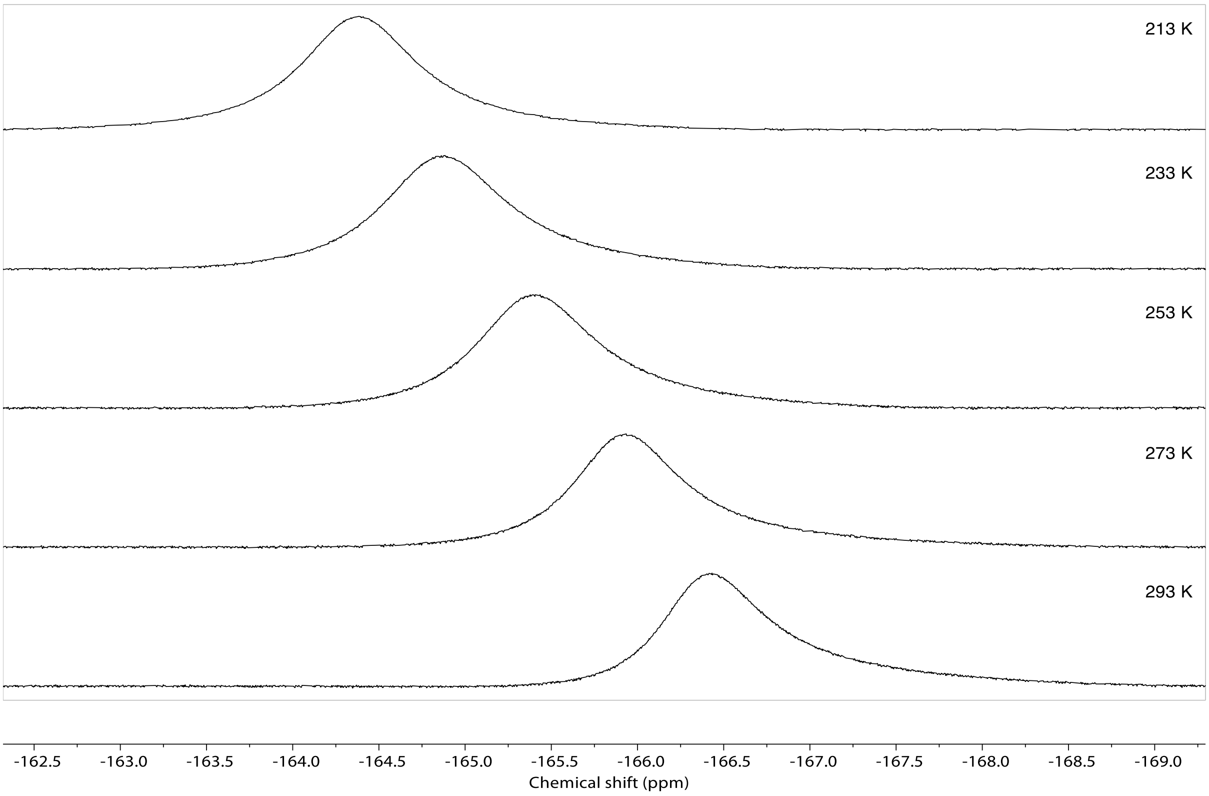

Figure S39: VT-^{19}F NMR for 3HF·TEA in DCM-d_2 in the temperature range 293 K and 213 K in 20 K intervals.

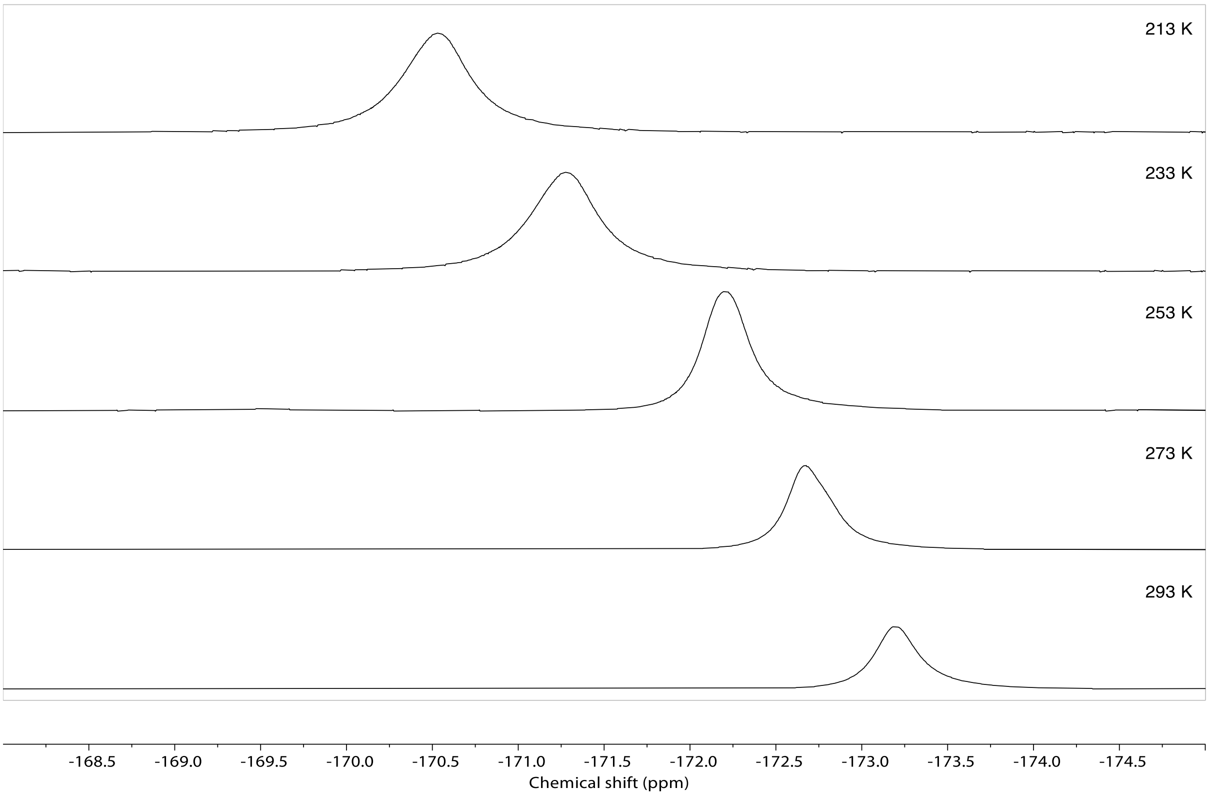

Figure S40: VT-^{19}F NMR for 4HF·TEA in DCM-d_2 in the temperature range 293 K and 213 K in 20 K intervals.

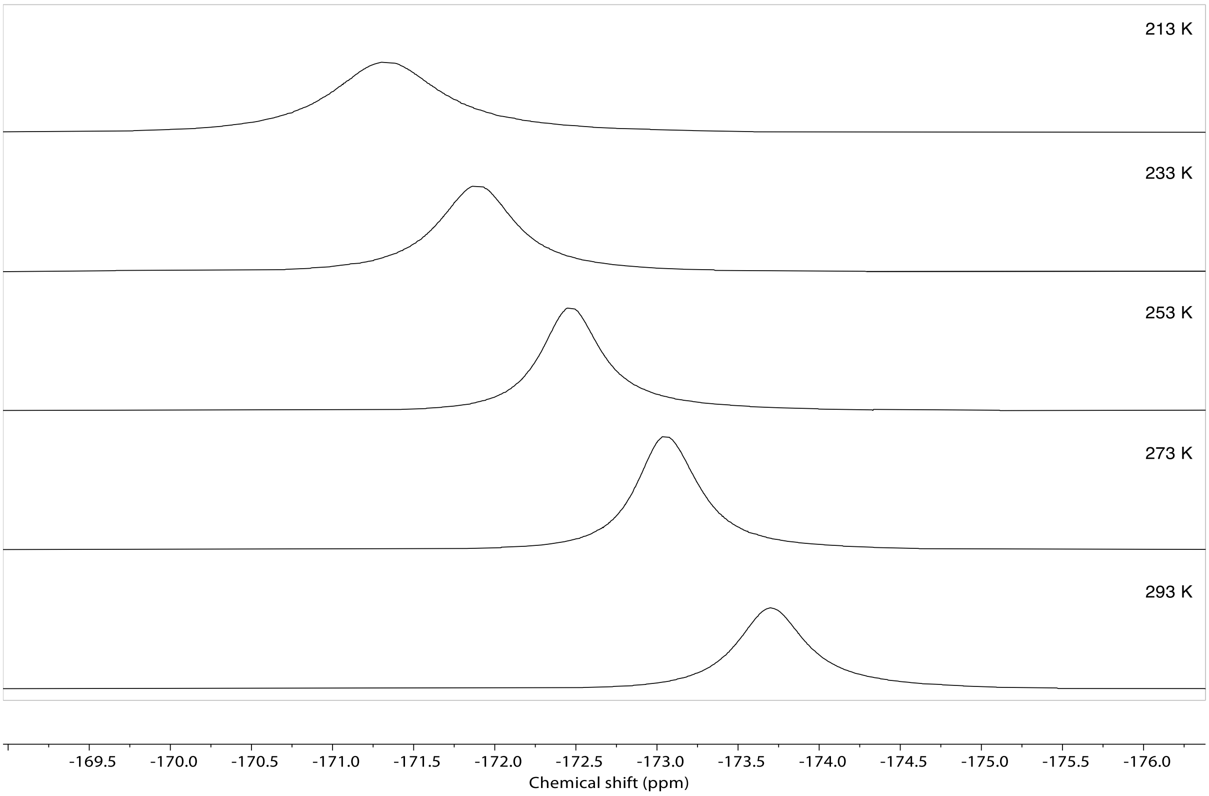

Figure S41: VT-^{19}F NMR for 5HF·TEA in DCM-d_2 in the temperature range 293 K and 213 K in 20 K intervals.

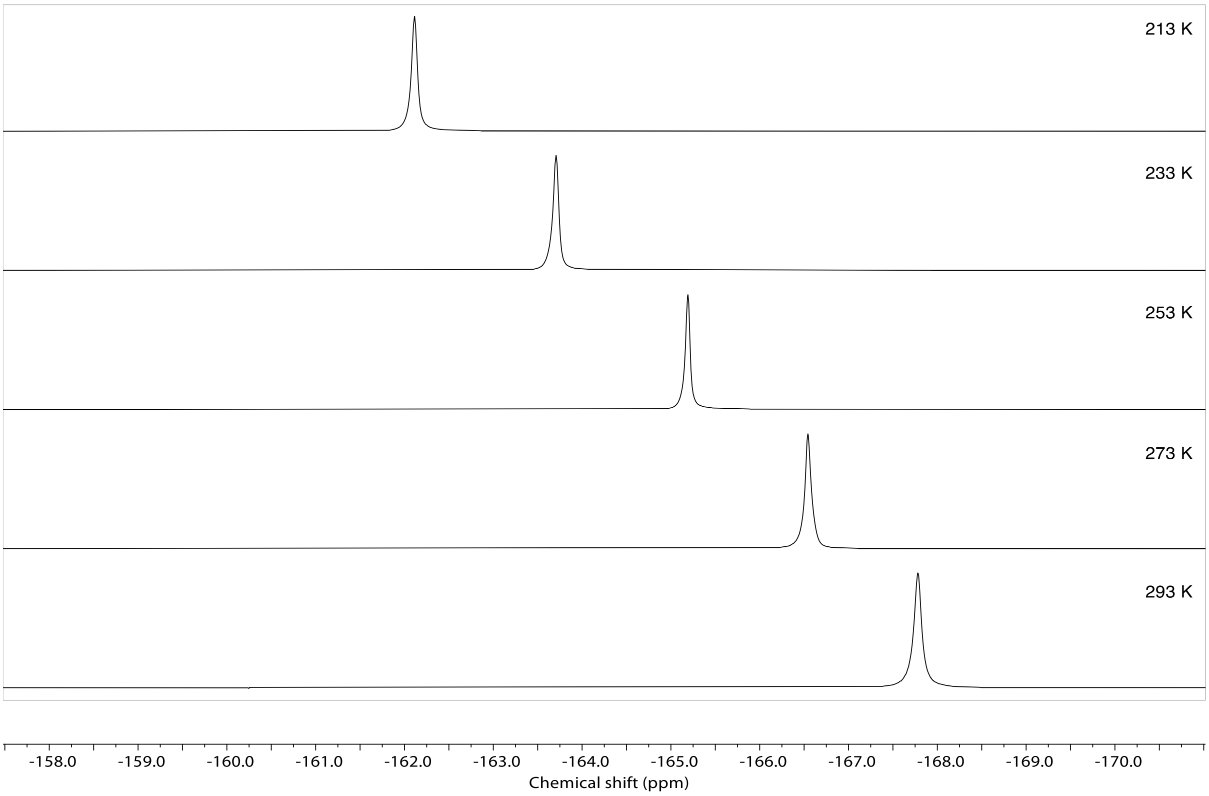

Figure S42: VT-^{19}F NMR for 1HF·py in DCM-d_2 in the temperature range 293 K and 213 K in 20 K intervals.

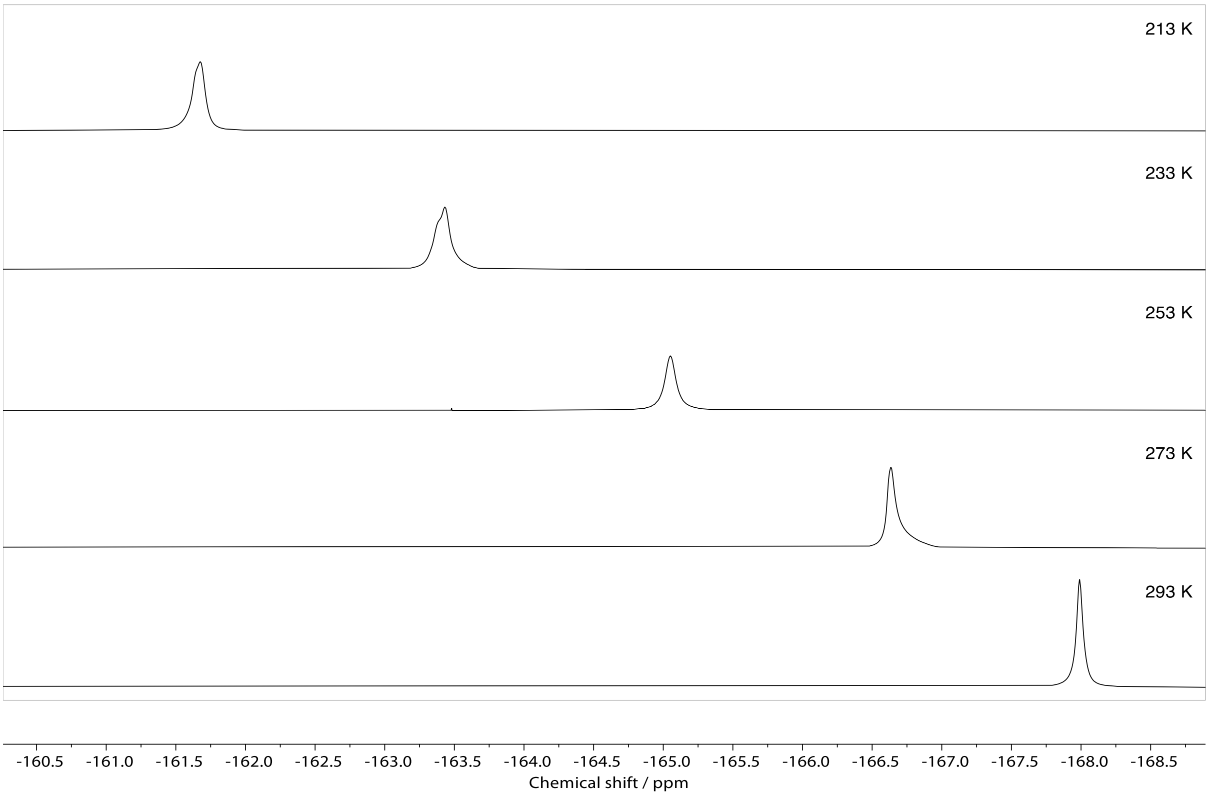

Figure S43: VT-^{19}F NMR for 2HF·py in DCM-d_2 in the temperature range 293 K and 213 K in 20 K intervals.

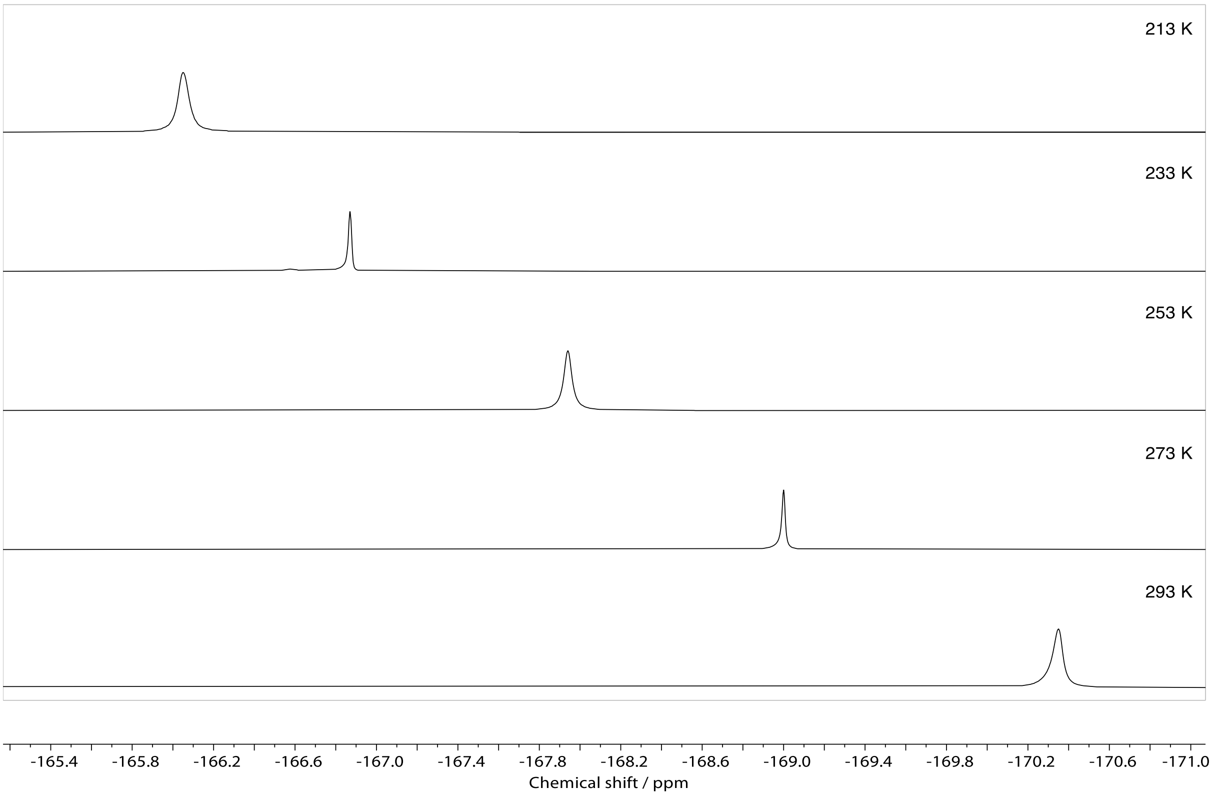

Figure S44: VT-^{19}F NMR for 3HF·py in DCM-d_2 in the temperature range 293 K and 213 K in 20 K intervals.

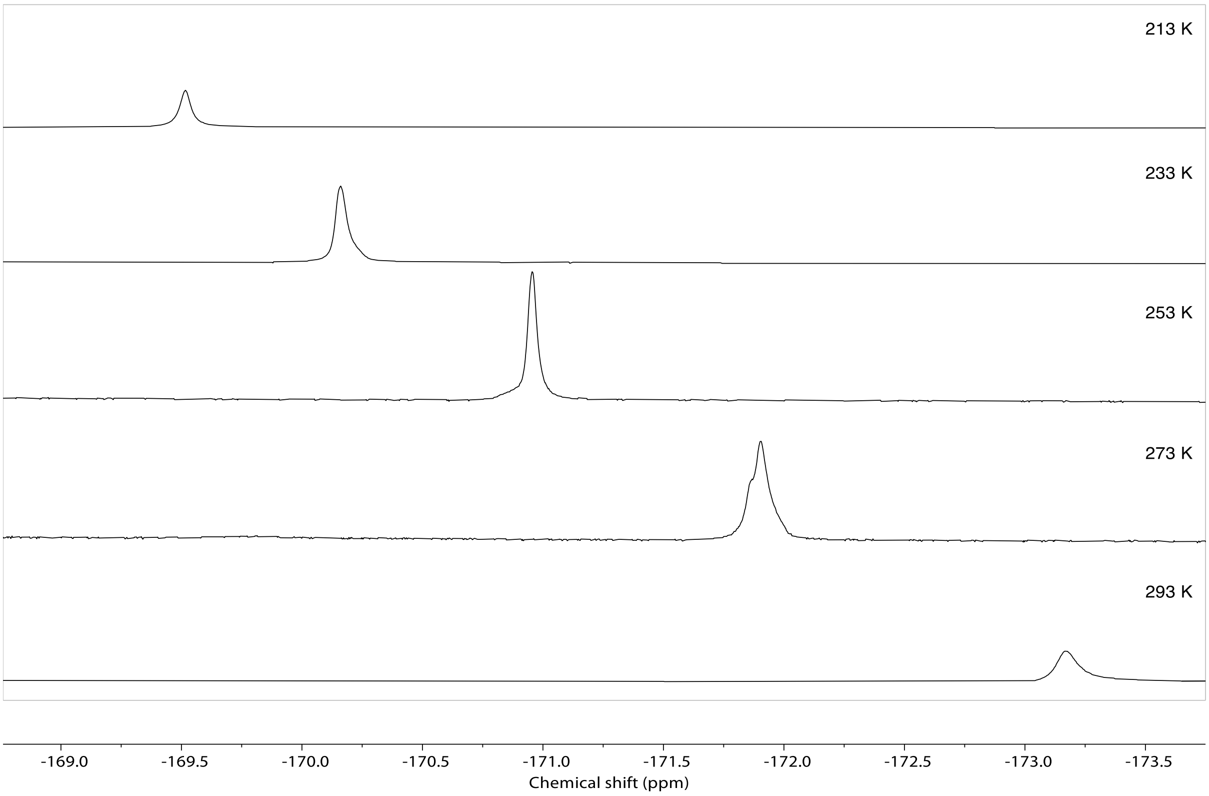

Figure S45: VT-^{19}F NMR for 4HF·py in DCM-d_2 in the temperature range 293 K and 213 K in 20 K intervals.

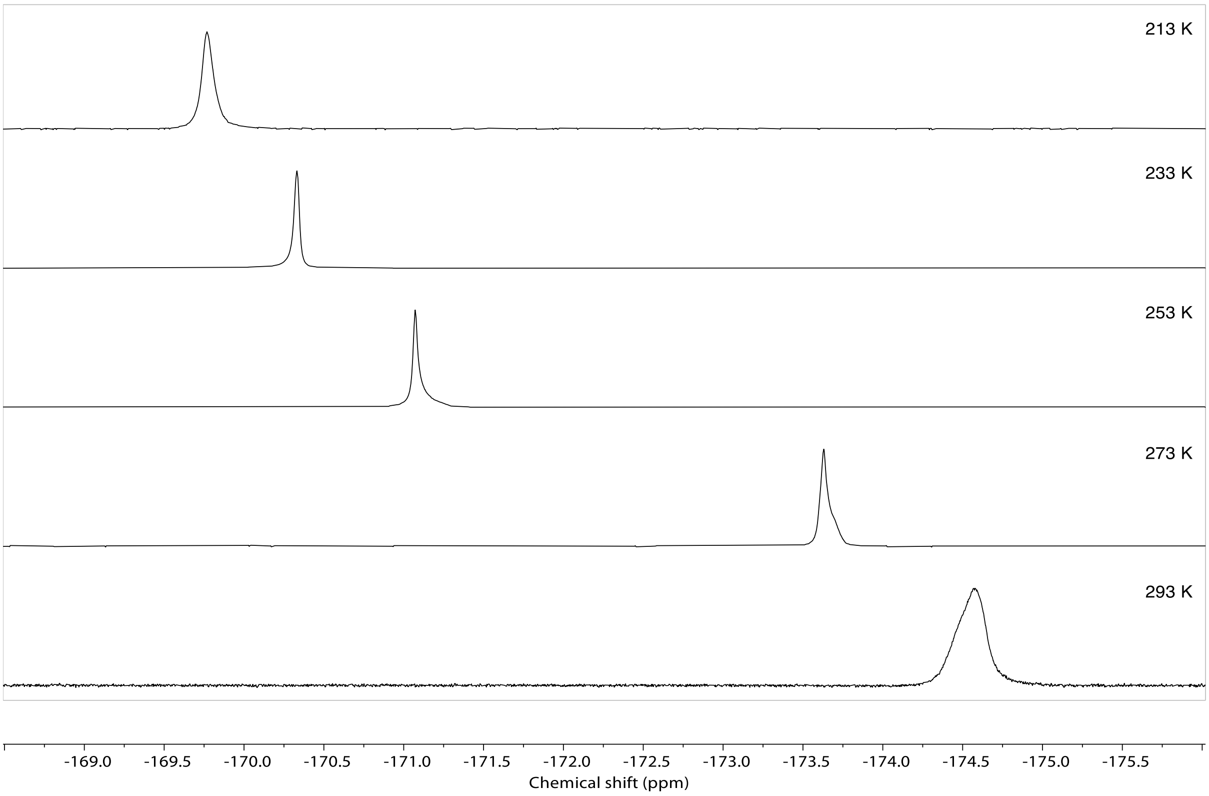

Figure S46: VT-^{19}F NMR for 5HF·py in DCM-d_2 in the temperature range 293 K and 213 K in 20 K intervals.

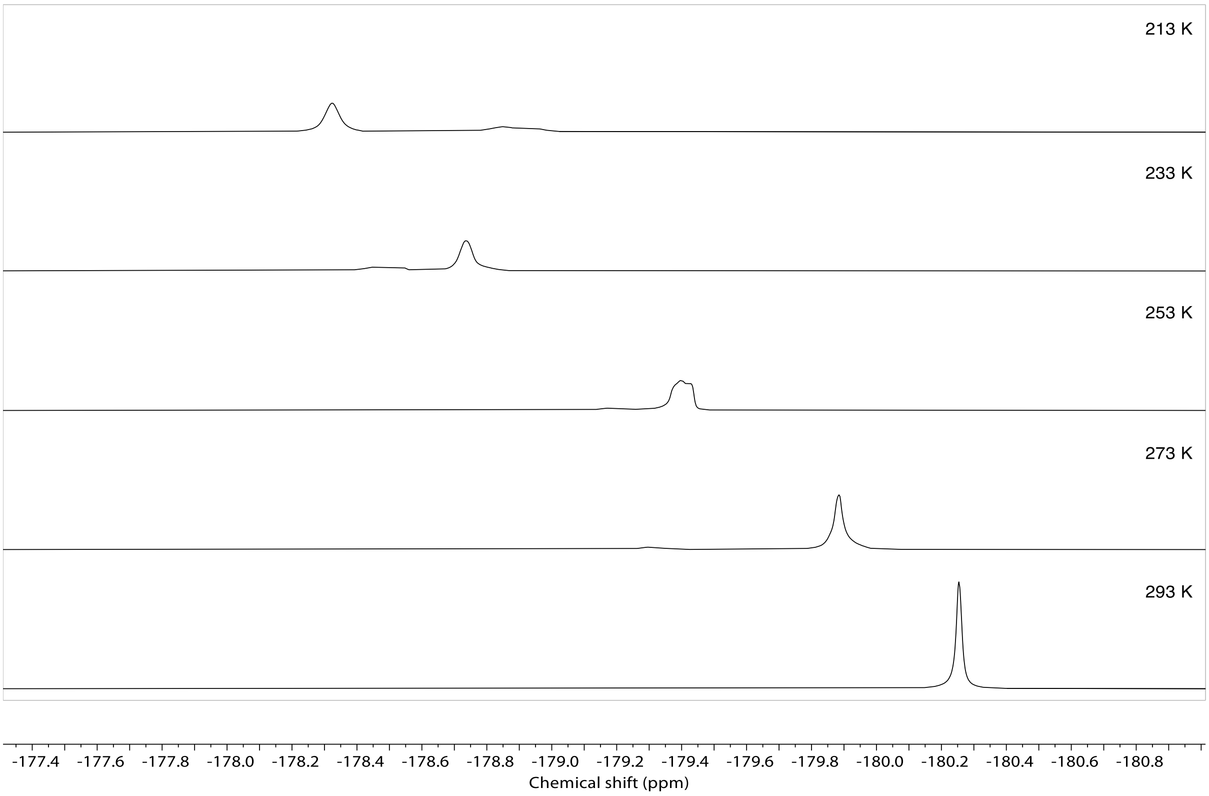

Figure S47: VT-^{19}F NMR for 6HF·py in DCM-d_2 in the temperature range 293 K and 213 K in 20 K intervals.

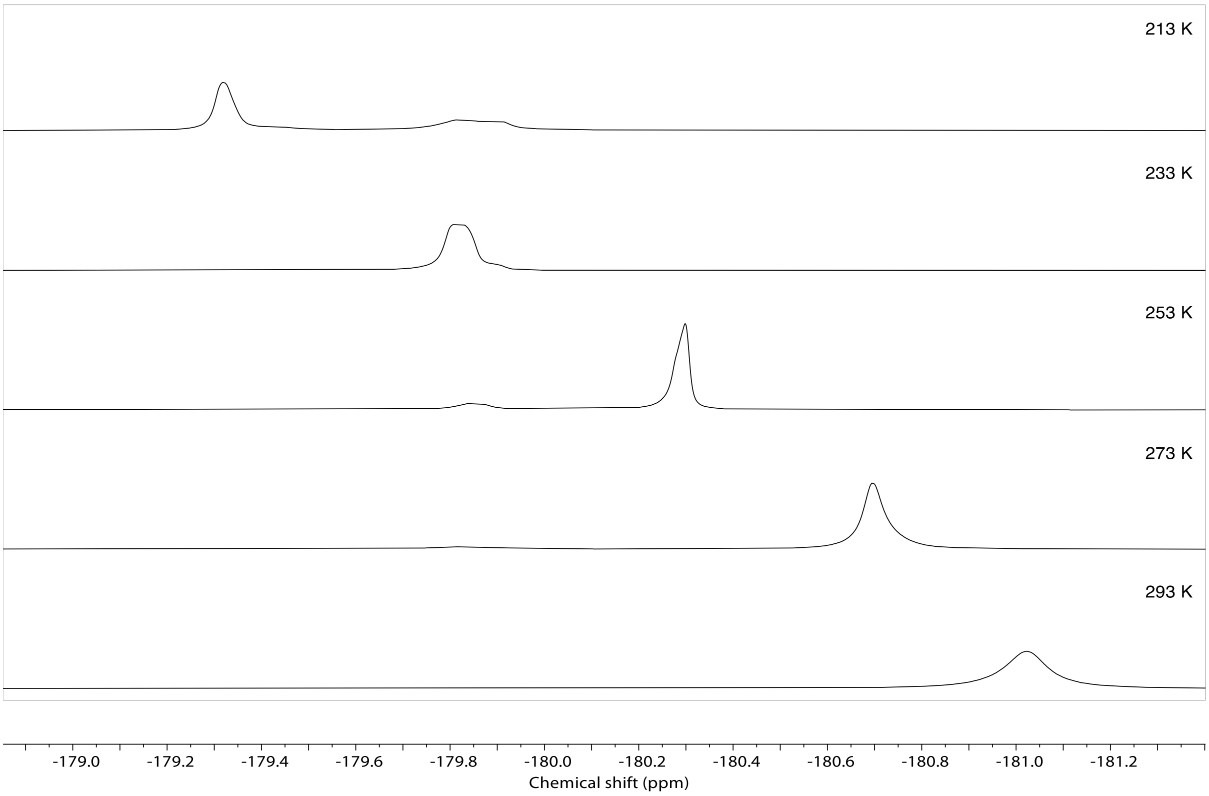

Figure S48: VT-^{19}F NMR for 7HF·py in DCM-d_2 in the temperature range 293 K and 213 K in 20 K intervals.

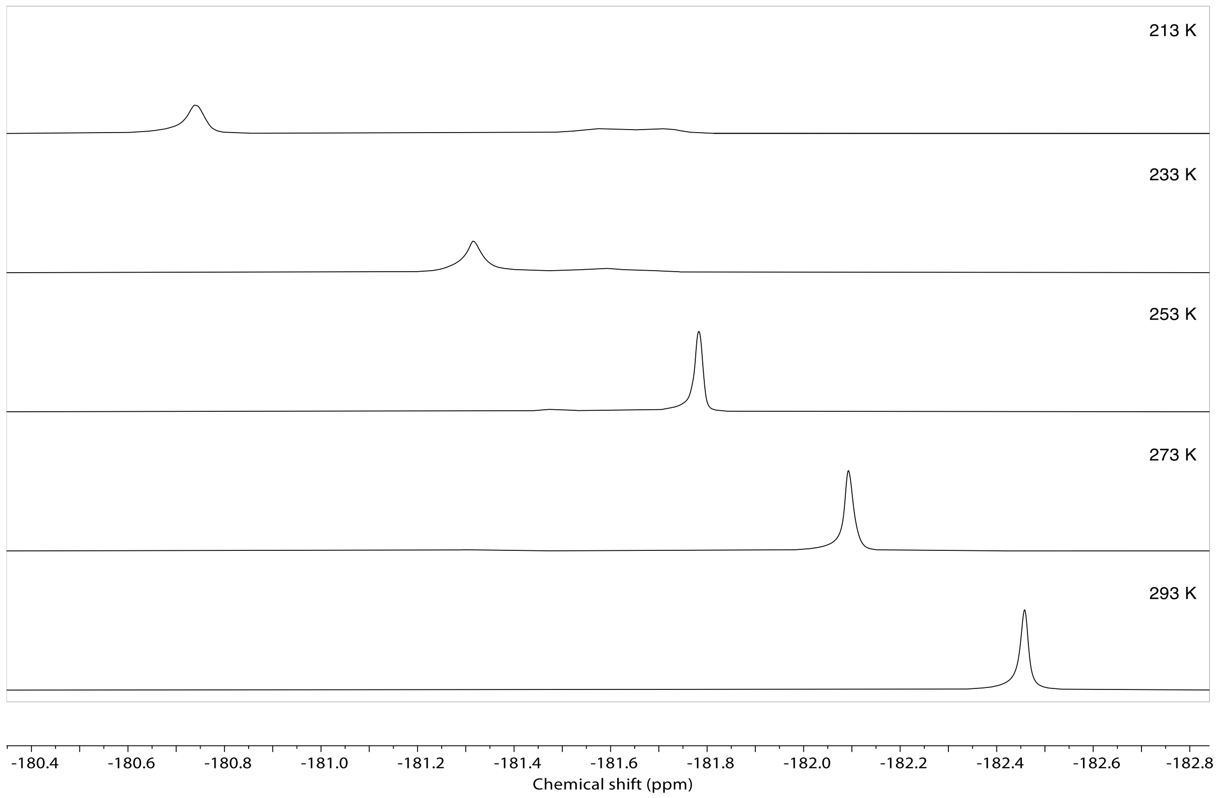

Figure S49: VT-^{19}F NMR for 8HF·py in DCM-d_2 in the temperature range 293 K and 213 K in 20 K intervals.

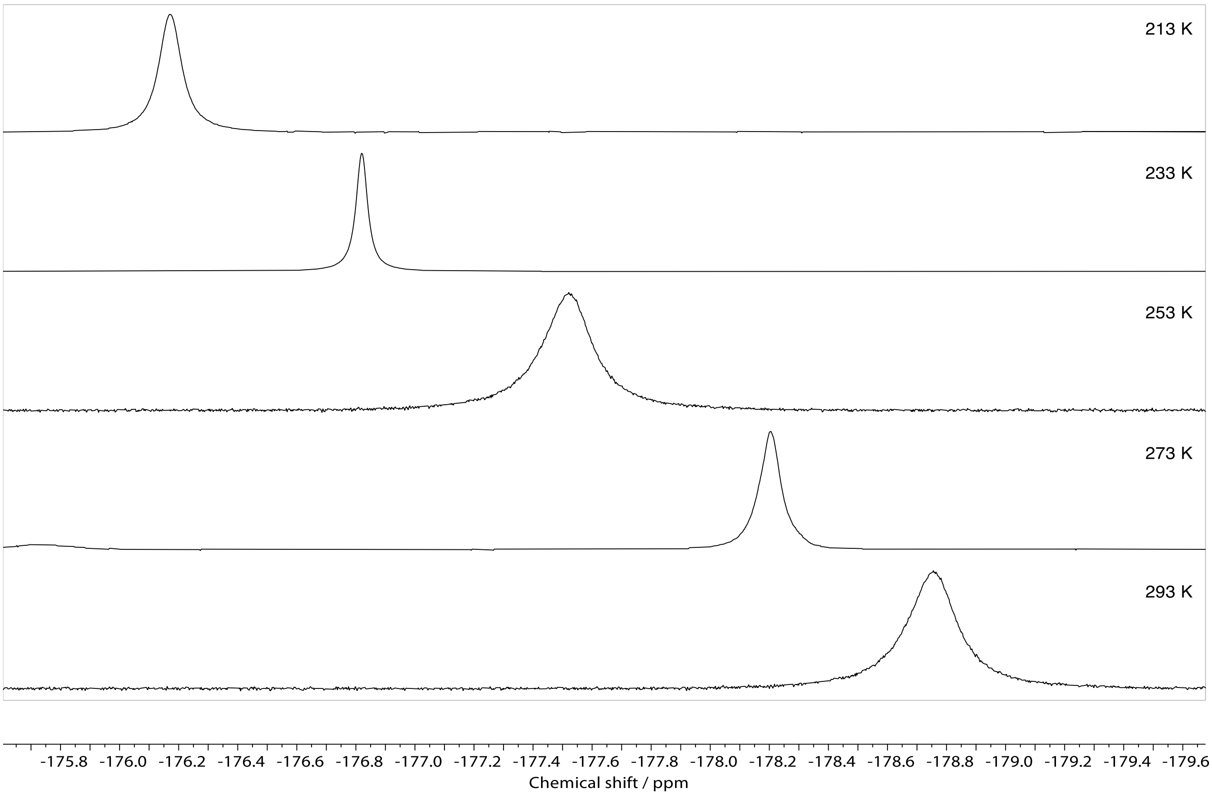

Figure S50: VT-^{19}F NMR for 9HF·py in DCM-d_2 in the temperature range 293 K and 213 K in 20 K intervals.

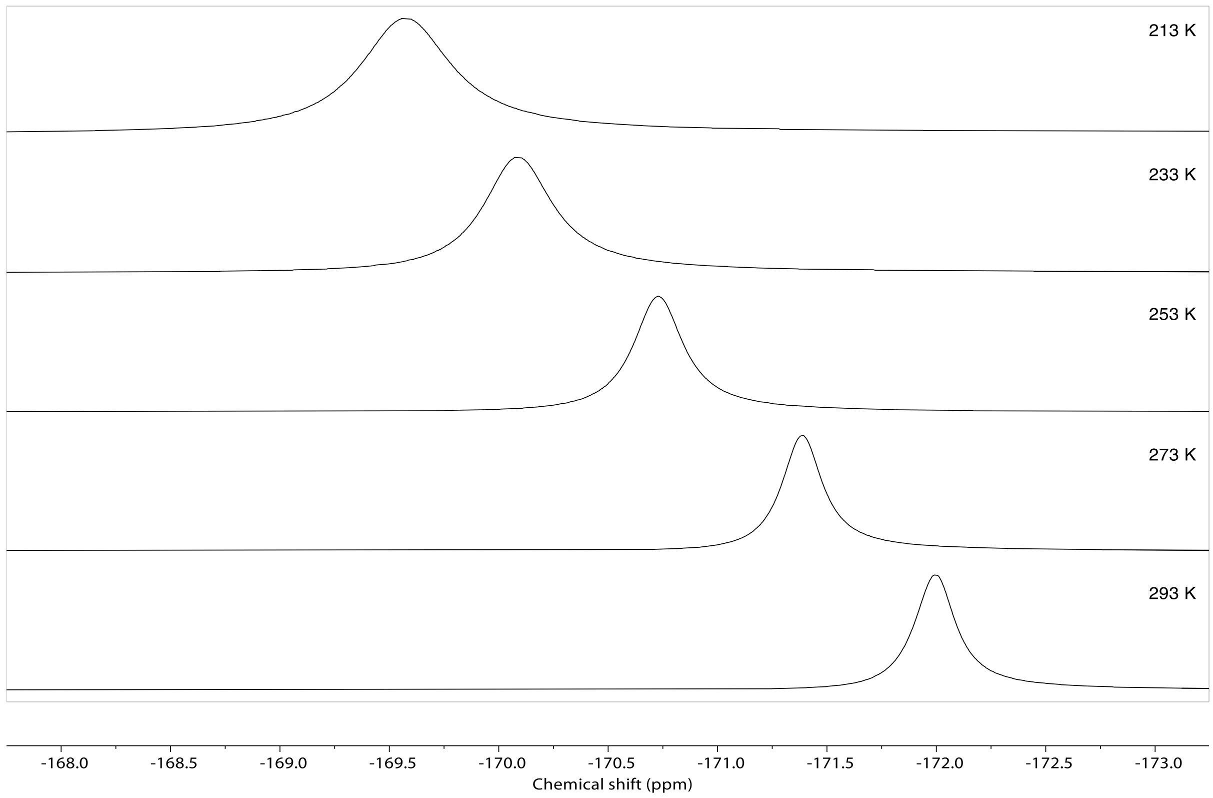

Figure S51: VT-^{19}F NMR for 4HF·amine in DCM-d_2 in the temperature range 293 K and 213 K in 20 K intervals.

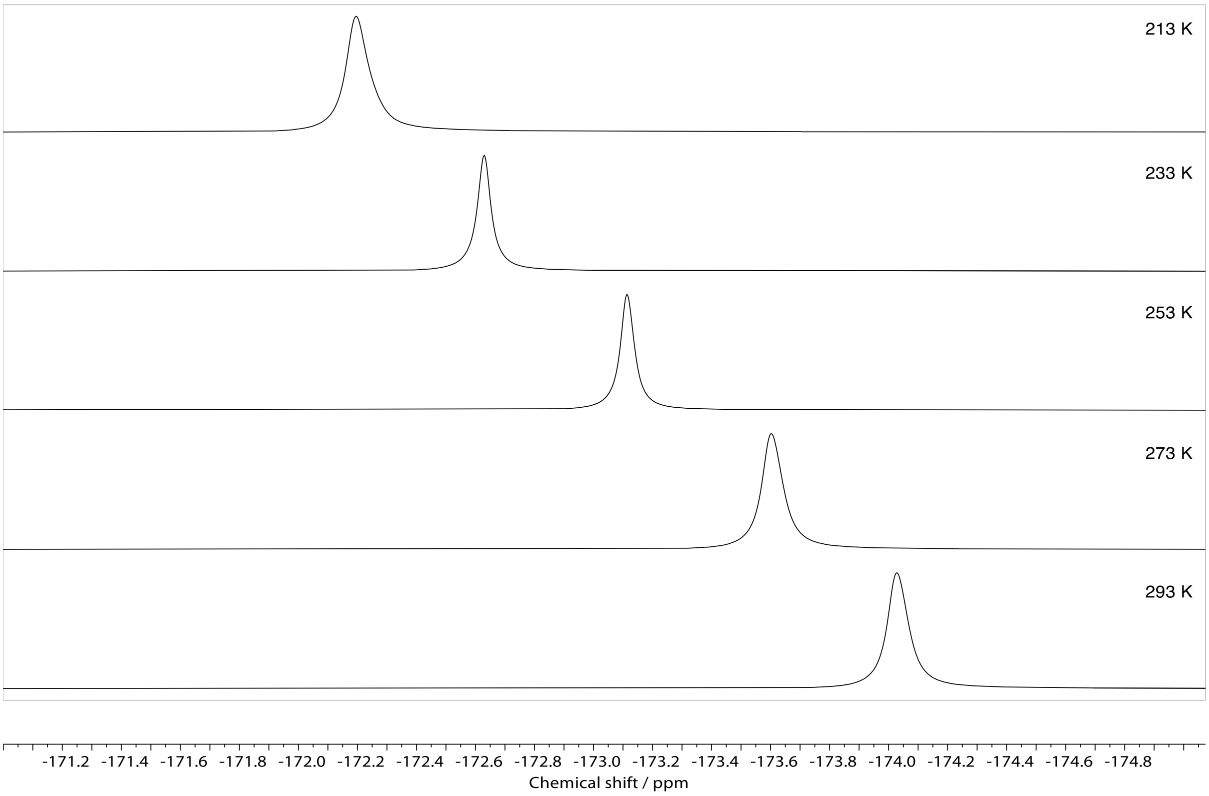

Figure S52: VT-^{19}F NMR for 5HF·amine in DCM-d_2 in the temperature range 293 K and 213 K in 20 K intervals.

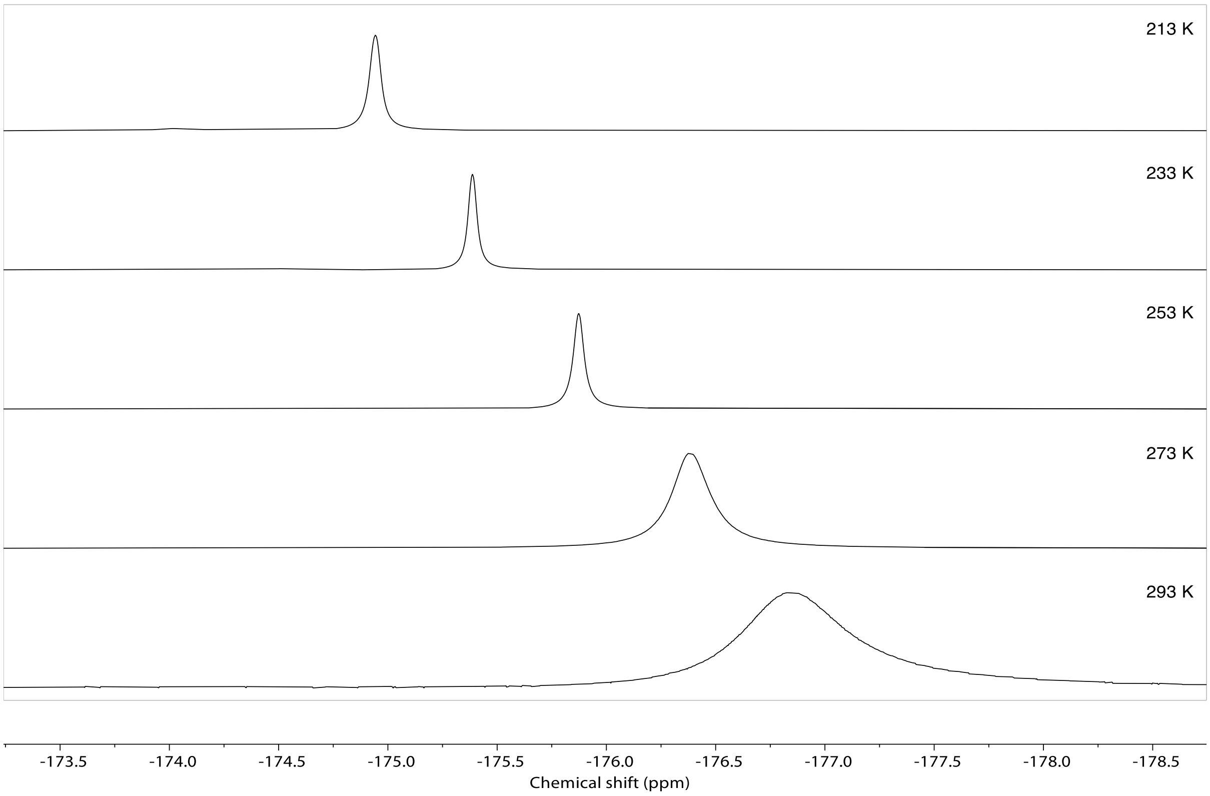

Figure S53: VT-^{19}F NMR for 6HF·amine in DCM-d_2 in the temperature range 293 K and 213 K in 20 K intervals.

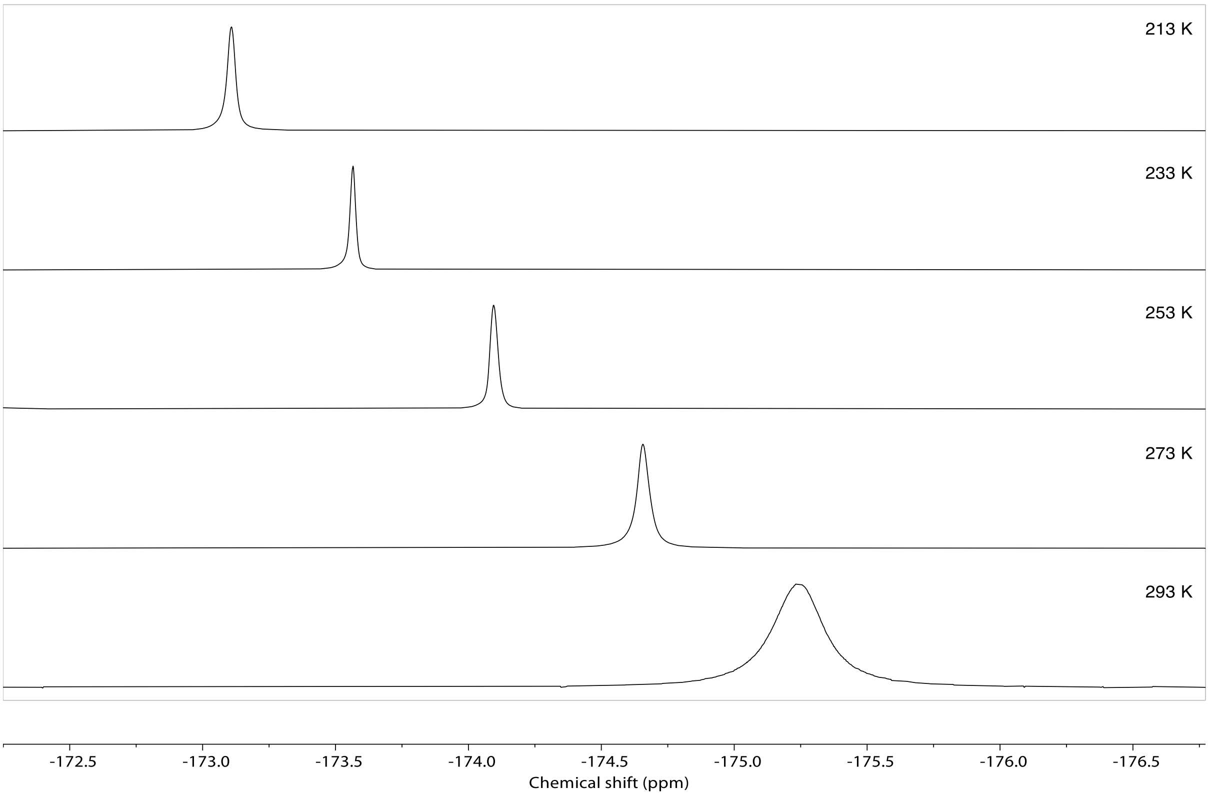

Figure S54: VT-^{19}F NMR for 7HF·amine in DCM-d_2 in the temperature range 293 K and 213 K in 20 K intervals.

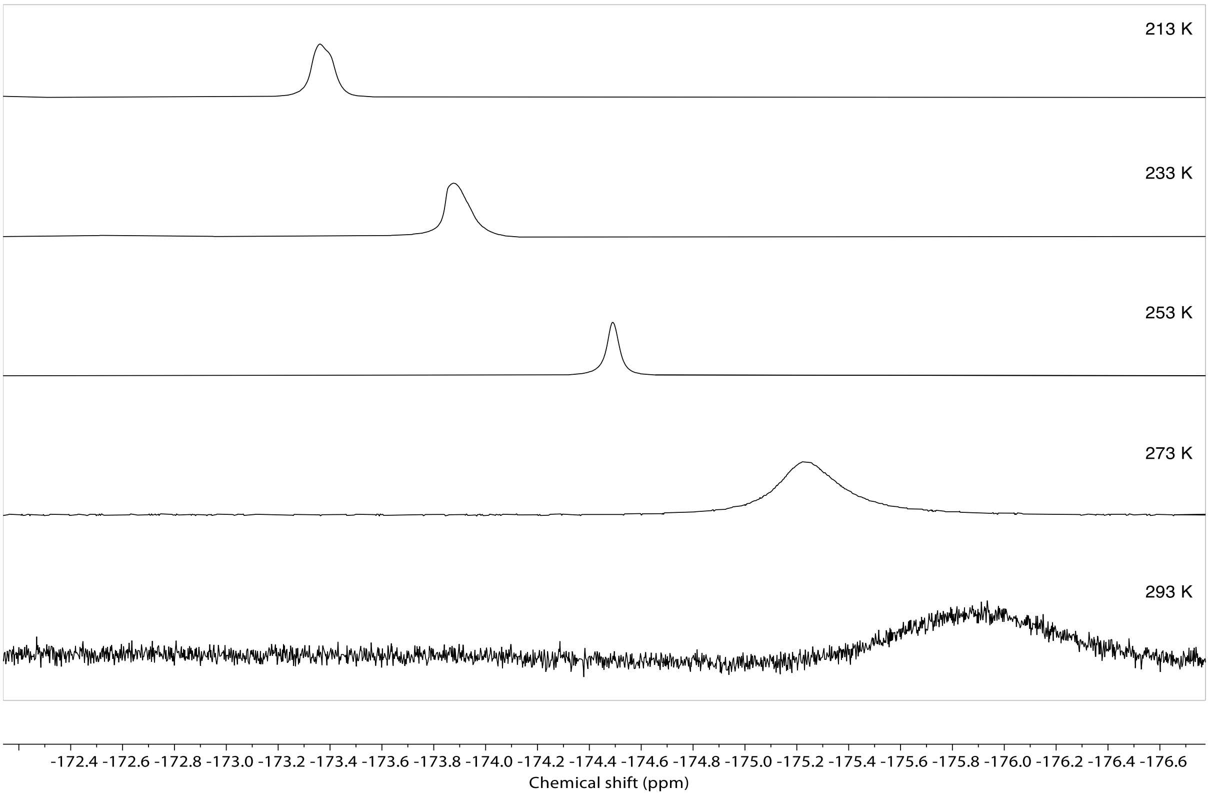

Figure S55: VT-^{19}F NMR for 8HF·amine in DCM-d_2 in the temperature range 293 K and 213 K in 20 K intervals.

7. Determining H_0 for *n*HF·base Mixtures

Acidity measurements were conducted using a series of Hammett bases, with their pK_{BH^+} values known, as shown in **Table S81**. To use the Hammett acidity function, the chemical shift for both the base and protonated bases were first required. Unprotonated carbon signals were obtained by producing 50 mᴍ solutions of each indicator in DCM-d_2, using the Bruker Avance III HD spectrometer Prodigy 500 at 0 °C. This temperature was used as it has been shown to lead to greater accuracy for NMR titrations involving HF mixtures previously in the literature.[53]

Table S81: Properties of the five Hammett bases used within this study.

Hammett base	pK_{BH^+}(from literature)	^{13}C NMR Chemical shift for H_2N-C (DCM-d_2) / ppm	^{13}C NMR Chemical shift for H_3N^+-C (aHF) / ppm
3-Nitroaniline	2.50	149.3	135.5
4-Nitroaniline	0.99	154.5	140.2
2-Nitroaniline	-0.29	148.0	136.0
2-Chloro-6-nitroaniline	-2.46	142.2	133.5
2,4-Dinitroaniline	-4.48	151.0	139.6

Chemical shifts for the protonated bases were obtained by dissolving the Hammett bases in anhydrous hydrogen fluoride (aHF). *Note: this was performed using our specially designed apparatus to safely handle aHF. This should not be repeated by anyone who does not have the correct apparatus and expertise to safely handle aHF.* To obtain these NMR values, the desired amount of Hammett base was first added to a FEP liner. Hydrogen fluoride gas was then slowly condensed into the FEP liner up to a predefined mark, to obtain a solution with a concentration of 50 mᴍ. The reaction between the Hammett base and the aHF was extremely vigorous. When the solution was produced, the FEP liner was then heat sealed so that the aHF was contained and remained liquid, allowing for ^{13}C NMR analysis to be performed. Before samples were analysed, samples were allowed to warm up to room temperature before being removed from the fumehood. This is to ensure that the seal does not fail during the analysis. ^{13}C NMR chemical shifts associated with the H_2N-C / H_3N^+-C are recorded in **Table S81** for both DCM-d_2 and aHF, respectively.

To verify the concentration of indicator that was in the sealed FEP NMR liner was 50 mᴍ, quantitative ^{1}H NMR spectrum was recorded for the Hammett base alongside an internal reference that was added coaxially (internal reference was tetramethylsilane in $CDCl_3$; 50 mᴍ). The internal reference was added coaxially to avoid interactions between the Hammett base and the indicator, which would affect the chemical shift of the indicator.

With the protonated and unprotonated Hammett base chemical shifts known, **Equation S32** can be used to determine the Hammett acidity (H_0) of each of the different *n*HF·base mixtures being studied:

$$H_0 = pK_{BH^+} - \log_{10}\left(\frac{\delta_B - \delta_{obs}}{\delta_{obs} - \delta_{BH^+}}\right) \quad \textbf{Equation S32}$$

where pK_{BH}^{+} is the pK_a of its conjugate acid, δ_B and $\delta_{BH}+$ are the ^{13}C NMR chemical shifts of the Hammett base and conjugate acid, respectively, and δ_{obs} is the effective chemical shift of the base in the solution being measured, which is the weighted average of both the indicator chemical shifts used. Solutions of each *n*HF·base mixture being studied were produced as described previously, and the desired amount of Hammett base was added to produce a 50 mᴍ solution. ^{13}C NMR measurements were then taken to determine δ_{obs} for each solution. The values of δ_{obs} for *n*HF·py, *n*HF·TEA and *n*HF·amine is shown in **Tables S82 – S84**.

Table S82: The chemical shifts for the five Hammett bases for each of the different *n*HF·py mixtures. **HB1**: 3-Nitroaniline; **HB2**: 4-Nitroaniline; **HB3**: 2-Nitroaniline; **HB4**: 2-Chloro-6-nitroaniline; **HB5**: 2,4-Dinitroaniline

nHF·py	**HB1** / ppm	**HB2** / ppm	**HB3** / ppm	**HB4** / ppm	**HB5** / ppm
1	135.8	146.6	147.3	-	-
2	-	140.5	139.4	142.1	-
3	-	140.3	137.3	141.7	-
4	-	-	136.7	141.3	-
5	-	-	136.4	140.6	-
6	-	-	136.1	139.1	150.9
7	-	-	-	135.1	150.5
8	-	-	-	133.7	147.1
9	-	-	-	-	141.8

Table S83: The chemical shifts for the five Hammett bases for each of the different *n*HF·TEA mixtures. **HB1**: 3-Nitroaniline; **HB2**: 4-Nitroaniline; **HB3**: 2-Nitroaniline; **HB4**: 2-Chloro-6-nitroaniline; **HB5**: 2,4-Dinitroaniline

*n*HF·TEA	**HB1** / ppm	**HB2** / ppm	**HB3** / ppm	**HB4** / ppm	**HB5** / ppm
3	-	140.8	141.2	142.1	-
4	-	140.6	139.9	142.1	-
5	-	140.4	138.8	142.0	-

Table S84: The chemical shifts for the five Hammett bases for each of the different *n*HF·amine mixtures. **HB1**: 3-Nitroaniline; **HB2**: 4-Nitroaniline; **HB3**: 2-Nitroaniline; **HB4**: 2-Chloro-6-nitroaniline; **HB5**: 2,4-Dinitroaniline

*n*HF·amine	**HB1** / ppm	**HB2** / ppm	**HB3** / ppm	**HB4** / ppm	**HB5** / ppm
4	-	140.4	138.8	142.0	-
5	-	140.3	137.6	141.8	-
6	-	140.3	136.9	141.5	-
7	-	-	136.2	140.0	-
8	-	-	-	134.4	150.1

Table S85: The H_0 values for nHF·py recorded according to concentration of HF and weight percentage.

*n*HF·py	H_0	HF wt%	[HF] / mol dm^{-3}
1HF·py	0.9	20.2	11.1
2HF·py	-0.7	33.6	18.5
3HF·py	-1.2	43.2	23.7
4HF·py	-1.5	50.3	27.7
5HF·py	-1.8	55.8	30.7
6HF·py	-2.2	60.3	33.1
7HF·py	-3.1	63.9	35.1
8HF·py	-4.2	66.9	36.8
9HF·py	-5.1	69.5	38.2

Table S86: The H_0 values for nHF·TEA recorded according to concentration of HF and weight percentage.

*n*HF·py	H_0	HF wt%	[HF] / mol dm^{-3}
3HF·py	-0.4	37.2	18.4
4HF·py	-0.6	44.2	21.8
5HF·py	-0.8	49.7	24.6

Table S87: The H_0 values for nHF·amine recorded according to concentration of HF and weight percentage.

*n*HF·py	H_0	HF wt%	[HF] / mol dm^{-3}
4HF·py	-0.8	45.1	22.8
5HF·py	-1.1	51.6	26.7
6HF·py	-1.4	57.1	30.1
7HF·py	-2.0	61.8	33.1
8HF·py	-3.4	65.9	35.8

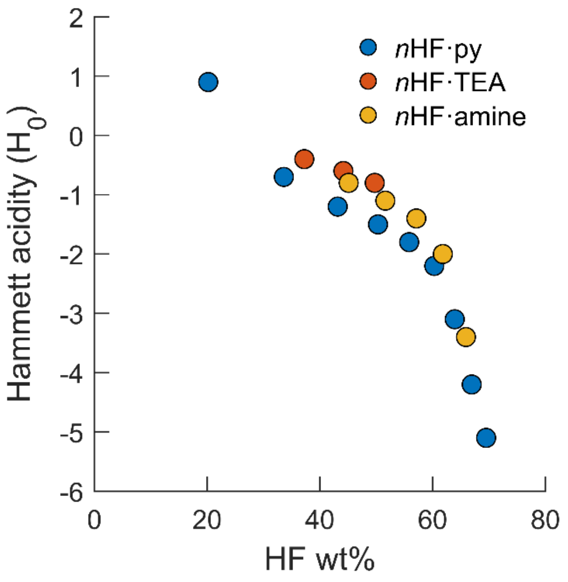

Figure S56: Hammett acidity functions (H_0) of each n in each nHF·base mixture plotted against concentration of HF.

To validate the method that we are proposing to determine the acidity of these mixtures, we replicated the method provided by Thibaudeau *et al.*[53] Recorded within the SI of this work, the acidity for *n*HF·py (n = 7 – 9) is reported. The values have been reported in **Table S88**, and

our experimentally determined values using our method agrees with the values obtained by Thibaudeau *et al*. These are the only values that we could compare, as Thibaudeau *et al*. measure *n*HF·py between 63 – 90 HF wt%, which covers 6.7HF·py to 35.5HF·py. Their method is only valid when mixtures have -2 > H_0 > -10 and was not able to measure 5HF·TEA, as suitable *N*-trifluoromethylanilines were shown to be volatile and unstable.[53] We therefore conclude that through the data available, that our method is complementary and allows the scope of H_0 in HF mixtures to be expanded to a greater range.

Table S88: Comparison of H_0 values obtained using our method and the method proposed by Thibaudeau *et al*.

*n*HF·Pyr	Our method	Thibaudeau method
7HF·Pyr	-3.1	-3.2
8HF·Pyr	-4.2	-4.1
9HF·Pyr	-5.1	-5.1

8. Kinetics

The HF mixture was first prepared in a cooled 20 mL HDPE vial (7.5 mL). The mixture was mixed for thirty minutes. Then a pre-prepared 1.0 M solution of 4-nitrobenzyl alcohol in DCM was added to the solution (2.5 mL). Aliquots of the mixture every five minutes for the first thirty minutes, followed by an aliquot for every thirty minutes for ninety minutes. Internal standard is separate from the reaction mixture and is achieved using a coaxial system (illustrated in **Figure S57**). ^{1}H NMR were recorded at each time point, and the rate was calculated from the change in the integrated area of a signal associated with both the substrate and the product. The two signals that were chosen were the benzylic protons for both the substrate and the product. The chemical shift for the benzylic proton for 4-nitrobenzyl bromide is 4.51 ppm (singlet), and for 4-nitrobenzyl fluoride it is 5.32 ppm (doublet)

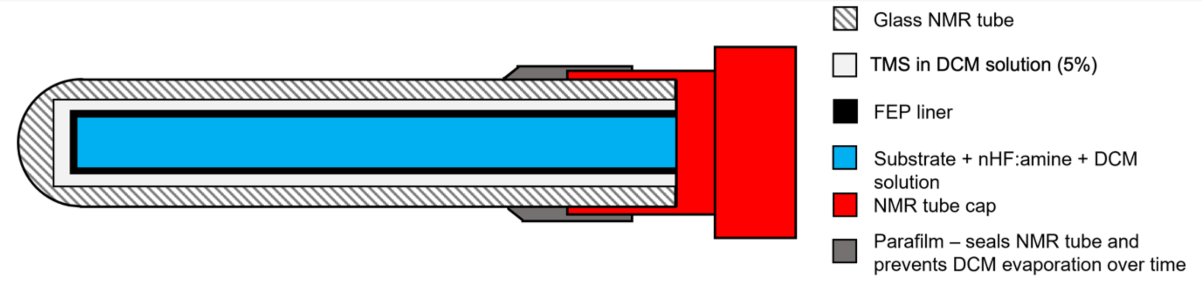

Figure S57: A cross section of an NMR tube used for monitoring the kinetics of a HF reaction.

The concentration of any given species at a given time is calculated using **Equation S33**

$$C_x = \frac{I_x}{I_{int}} * \frac{N_{int}}{N_x} * C_{int}$$ **Equation S33**

where C_x is the concentration of species x, I_x is the integrated area of the signal belonging to the compound of interest, I_{int} is the integrated area of the signal associated with the internal standard (5.0% TMS solution in DCM; chemical shift of 0.0ppm), N_{int} is the number of nuclei associated with the signal of the internal standard (N_{int} = 12 for all kinetic runs recorded within), N_x is the number of nuclei associated with the signal of the desired species (N_x = 2 for non-deuterated substrates; N_x = 1 for deuterated substrates) and C_{int} is the concentration of the internal standard.

The initial rate for each of the reactions was calculated graphically from the slope of each kinetic plot. The rate was also calculated using a genetic algorithm provided by the software

package COPASI when wanting to model reversibility and is shown as the solid line in **Figure S58 – S77**.[54]

Table S89: The concentrations of all the different chemical species involved in the reaction between 4-nitrobenzyl bromide and the fluoride anion in 1HF·py over a two-hour period.

Time	Measured concentration of substrate / mol dm^{-3}	Calculated concentration of fluoride / mol dm^{-3}	Measured concentration of product / mol dm^{-3}	Calculated concentration of bromide / mol dm^{-3}
0	0.254	7.501	0.000	0.000
300	0.006	7.253	0.248	0.248
600	0.005	7.252	0.249	0.249
900	0.005	7.252	0.249	0.249
1200	0.005	7.252	0.249	0.249
1500	0.005	7.252	0.249	0.249
1800	0.005	7.252	0.249	0.249
3600	0.005	7.252	0.249	0.249
5400	0.005	7.252	0.249	0.249
7200	0.005	7.252	0.249	0.249

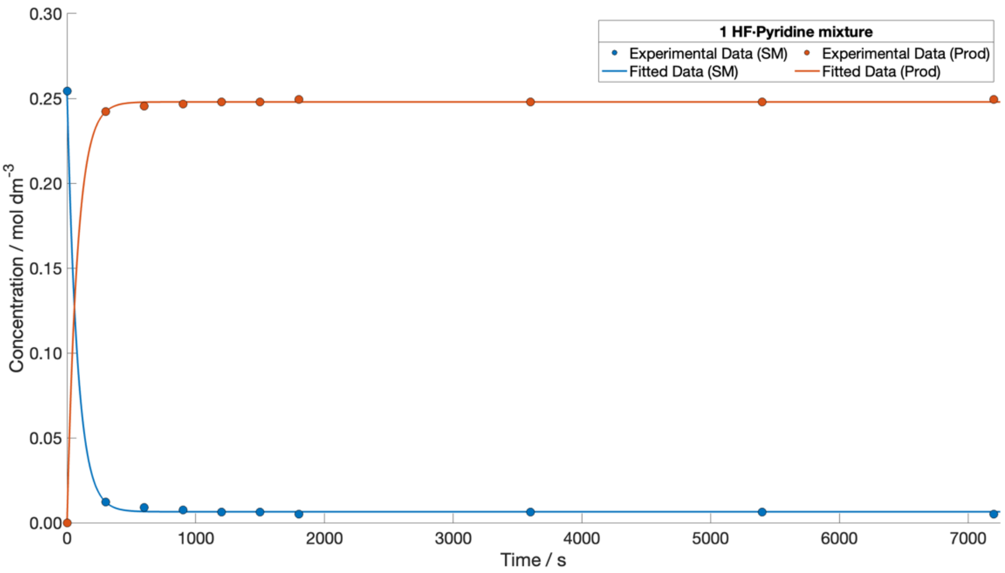

Figure S58: The kinetic run involving 1HF·py as a source of fluoride for the nucleophilic substitution of 4-nitrobenzyl bromide (SM) to 4-nitrobenzyl fluoride (Prod). Fluoride anion and bromide anion concentrations are omitted from this plot.

Table S90: The concentrations of all the different chemical species involved in the reaction between 4-nitrobenzyl bromide and the fluoride anion in 2HF·py over a two-hour period.

Time	Measured concentration of substrate / mol dm^{-3}	Calculated concentration of fluoride / mol dm^{-3}	Measured concentration of product / mol dm^{-3}	Calculated concentration of bromide / mol dm^{-3}
0	0.254	6.368	0.000	0.000
300	0.060	6.174	0.194	0.194
600	0.023	6.137	0.231	0.231
900	0.016	6.130	0.238	0.238
1200	0.015	6.129	0.239	0.239
1500	0.015	6.129	0.239	0.239
1800	0.015	6.129	0.239	0.239
3600	0.015	6.129	0.239	0.239
5400	0.015	6.129	0.239	0.239
7200	0.015	6.129	0.239	0.239

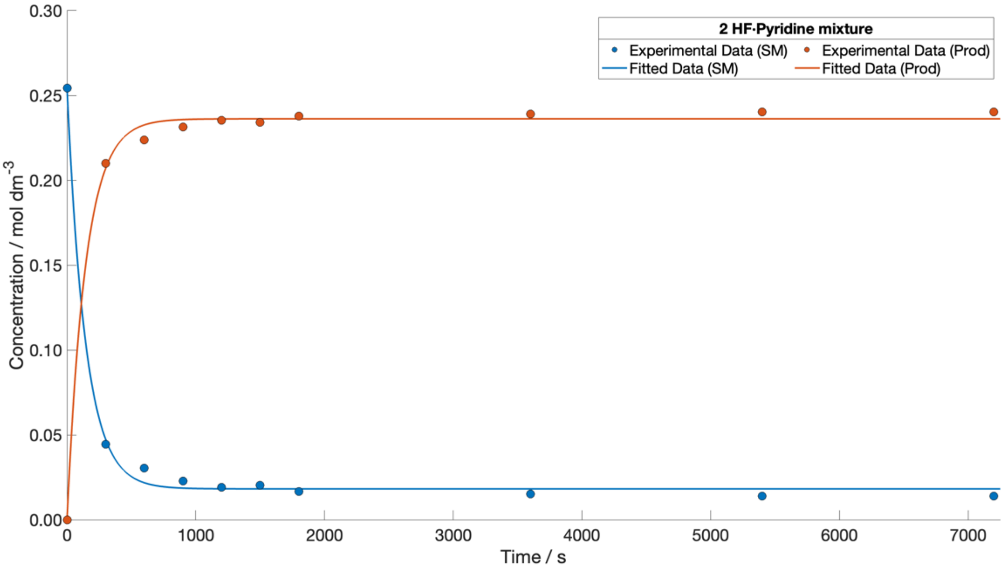

Figure S59: The kinetic run involving 2HF·py as a source of fluoride for the nucleophilic substitution of 4-nitrobenzyl bromide (SM) to 4-nitrobenzyl fluoride (Prod). Fluoride anion and bromide anion concentrations are omitted from this plot.

Table S91: The concentrations of all the different chemical species involved in the reaction between 4-nitrobenzyl bromide and the fluoride anion in 3HF·py over a two-hour period.

Time	Measured concentration of substrate / mol dm^{-3}	Calculated concentration of fluoride / mol dm^{-3}	Measured concentration of product / mol dm^{-3}	Calculated concentration of bromide / mol dm^{-3}
0	0.254	5.632	0.000	0.000
300	0.146	5.524	0.108	0.108
600	0.098	5.476	0.156	0.156
900	0.065	5.442	0.190	0.190
1200	0.043	5.421	0.211	0.211
1500	0.034	5.412	0.220	0.220
1800	0.031	5.408	0.224	0.224
3600	0.025	5.403	0.229	0.229
5400	0.024	5.402	0.230	0.230
7200	0.024	5.402	0.230	0.230

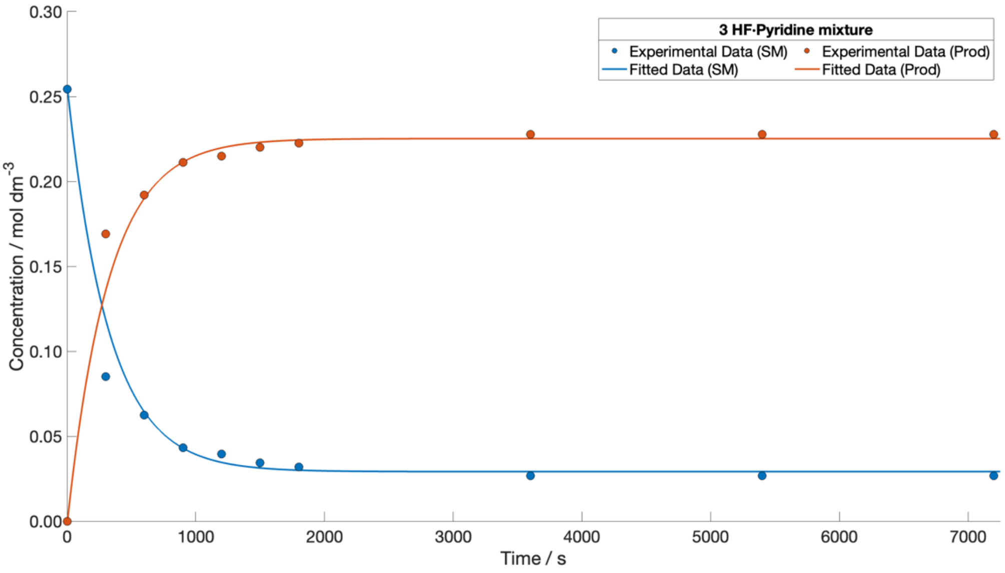

Figure S60: The kinetic run involving 3HF·py as a source of fluoride for the nucleophilic substitution of 4-nitrobenzyl bromide (SM) to 4-nitrobenzyl fluoride (Prod). Fluoride anion and bromide anion concentrations are omitted from this plot.

Table S92: The concentrations of all the different chemical species involved in the reaction between 4-nitrobenzyl bromide and the fluoride anion in 4HF·py over a two-hour period.

Time	Measured concentration of substrate / mol dm^{-3}	Calculated concentration of fluoride / mol dm^{-3}	Measured concentration of product / mol dm^{-3}	Calculated concentration of bromide / mol dm^{-3}
0	0.254	4.981	0.000	0.000
300	0.181	4.908	0.073	0.073
600	0.121	4.848	0.133	0.133
900	0.085	4.812	0.169	0.169
1200	0.063	4.790	0.191	0.191
1500	0.049	4.776	0.205	0.205
1800	0.041	4.768	0.213	0.213
3600	0.029	4.756	0.225	0.225
5400	0.028	4.755	0.226	0.226
7200	0.028	4.755	0.226	0.226

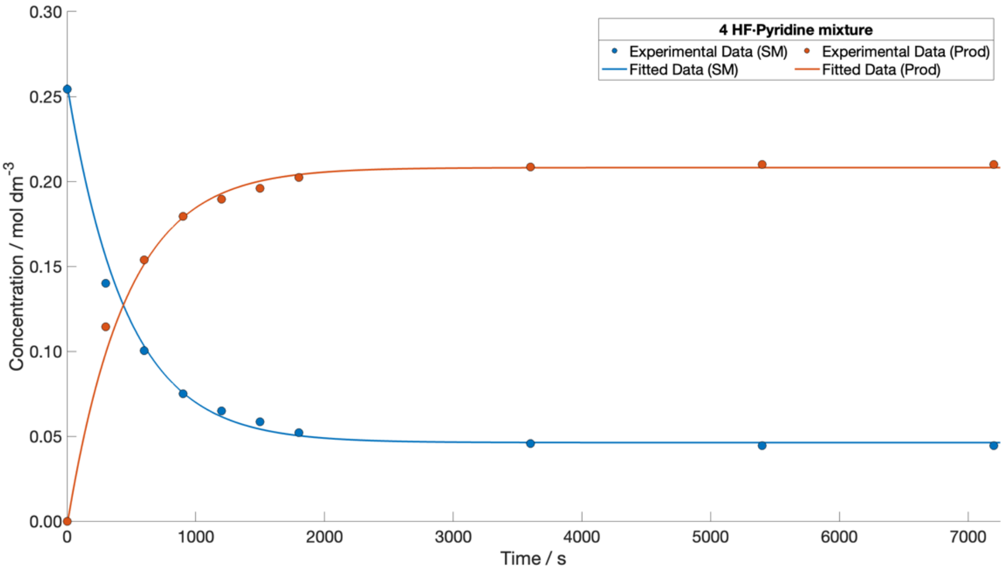

Figure S61: The kinetic run involving 4HF·py as a source of fluoride for the nucleophilic substitution of 4-nitrobenzyl bromide (SM) to 4-nitrobenzyl fluoride (Prod). Fluoride anion and bromide anion concentrations are omitted from this plot.

Table S93: The concentrations of all the different chemical species involved in the reaction between 4-nitrobenzyl bromide and the fluoride anion in 5HF·py over a two-hour period.

Time	Measured concentration of substrate / mol dm^{-3}	Calculated concentration of fluoride / mol dm^{-3}	Measured concentration of product / mol dm^{-3}	Calculated concentration of bromide / mol dm^{-3}
0	0.254	4.405	0.000	0.000
300	0.195	4.347	0.059	0.059
600	0.133	4.285	0.121	0.121
900	0.099	4.251	0.155	0.155
1200	0.080	4.232	0.174	0.174
1500	0.070	4.222	0.184	0.184
1800	0.064	4.216	0.190	0.190
3600	0.057	4.209	0.197	0.197
5400	0.057	4.209	0.197	0.197
7200	0.057	4.209	0.197	0.197

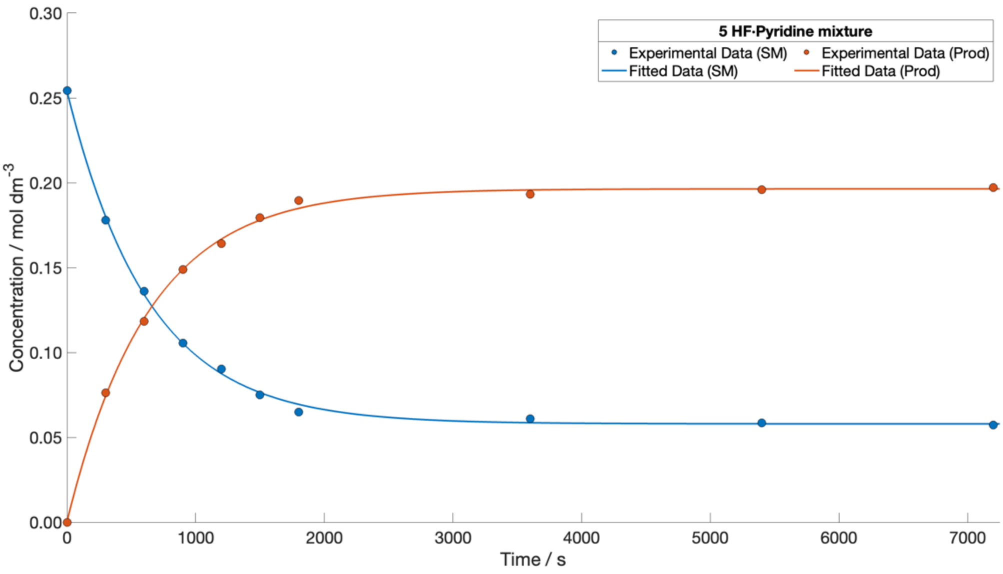

Figure S62: The kinetic run involving 5HF·py as a source of fluoride for the nucleophilic substitution of 4-nitrobenzyl bromide (SM) to 4-nitrobenzyl fluoride (Prod). Fluoride anion and bromide anion concentrations are omitted from this plot.

Table S94: The concentrations of all the different chemical species involved in the reaction between 4-nitrobenzyl bromide and the fluoride anion in 6HF·py over a two-hour period.

Time	Measured concentration of substrate / mol dm^{-3}	Calculated concentration of fluoride / mol dm^{-3}	Measured concentration of product / mol dm^{-3}	Calculated concentration of bromide / mol dm^{-3}
0	0.254	4.059	0.000	0.000
300	0.200	4.005	0.054	0.054
600	0.139	3.944	0.115	0.115
900	0.104	3.909	0.150	0.150
1200	0.083	3.888	0.171	0.171
1500	0.072	3.877	0.182	0.182
1800	0.065	3.870	0.189	0.189
3600	0.056	3.861	0.198	0.198
5400	0.056	3.861	0.198	0.198
7200	0.056	3.861	0.198	0.198

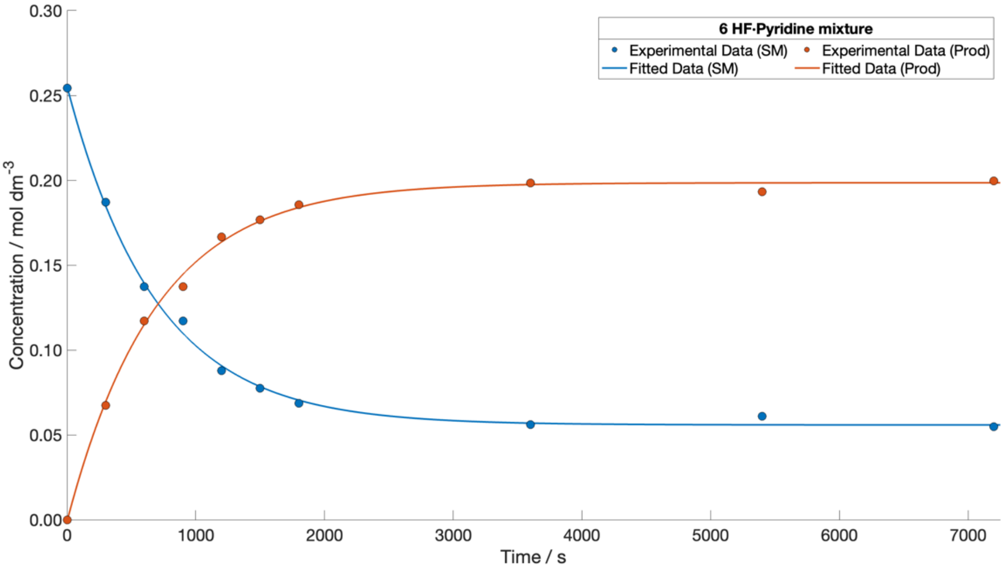

Figure S63: The kinetic run involving 6HF·py as a source of fluoride for the nucleophilic substitution of 4-nitrobenzyl bromide (SM) to 4-nitrobenzyl fluoride (Prod). Fluoride anion and bromide anion concentrations are omitted from this plot.

Table S95: The concentrations of all the different chemical species involved in the reaction between 4-nitrobenzyl bromide and the fluoride anion in 7HF·py over a two-hour period.

Time	Measured concentration of substrate / mol dm^{-3}	Calculated concentration of fluoride / mol dm^{-3}	Measured concentration of product / mol dm^{-3}	Calculated concentration of bromide / mol dm^{-3}
0	0.254	3.590	0.000	0.000
300	0.211	3.547	0.043	0.043
600	0.151	3.487	0.103	0.103
900	0.115	3.451	0.139	0.139
1200	0.093	3.429	0.161	0.161
1500	0.080	3.416	0.174	0.174
1800	0.072	3.408	0.182	0.182
3600	0.061	3.396	0.193	0.193
5400	0.060	3.396	0.194	0.194
7200	0.060	3.396	0.194	0.194

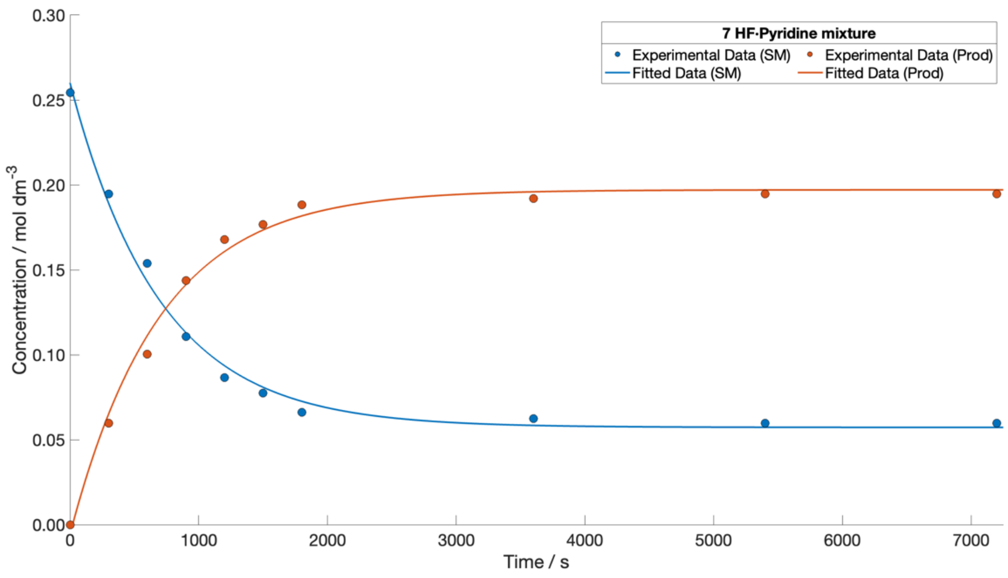

Figure S64: The kinetic run involving 7HF·py as a source of fluoride for the nucleophilic substitution of 4-nitrobenzyl bromide (SM) to 4-nitrobenzyl fluoride (Prod). Fluoride anion and bromide anion concentrations are omitted from this plot.

Table S96: The concentrations of all the different chemical species involved in the reaction between 4-nitrobenzyl bromide and the fluoride anion in 8HF·py over a two-hour period.

Time	Measured concentration of substrate / mol dm^{-3}	Calculated concentration of fluoride / mol dm^{-3}	Measured concentration of product / mol dm^{-3}	Calculated concentration of bromide / mol dm^{-3}
0	0.254	3.308	0.000	0.000
300	0.217	3.270	0.037	0.037
600	0.158	3.212	0.096	0.096
900	0.122	3.175	0.132	0.132
1200	0.099	3.152	0.155	0.155
1500	0.084	3.138	0.170	0.170
1800	0.075	3.129	0.179	0.179
3600	0.061	3.115	0.193	0.193
5400	0.060	3.114	0.194	0.194
7200	0.060	3.114	0.194	0.194

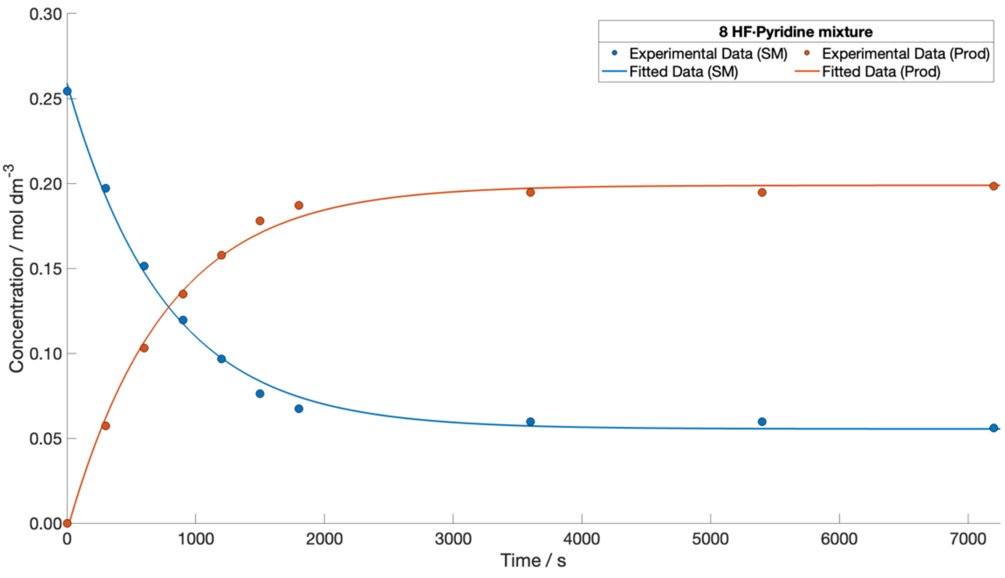

Figure S65: The kinetic run involving 8HF·py as a source of fluoride for the nucleophilic substitution of 4-nitrobenzyl bromide (SM) to 4-nitrobenzyl fluoride (Prod). Fluoride anion and bromide anion concentrations are omitted from this plot.

Table S97: The concentrations of all the different chemical species involved in the reaction between 9-nitrobenzyl bromide and the fluoride anion in 9HF·py over a two-hour period.

Time	Measured concentration of substrate / mol dm^{-3}	Calculated concentration of fluoride / mol dm^{-3}	Measured concentration of product / mol dm^{-3}	Calculated concentration of bromide / mol dm^{-3}
0	0.254	3.008	0.000	0.000
300	0.221	2.975	0.033	0.033
600	0.168	2.922	0.086	0.086
900	0.132	2.885	0.122	0.122
1200	0.106	2.860	0.148	0.148
1500	0.088	2.842	0.166	0.166
1800	0.076	2.829	0.178	0.178
3600	0.050	2.804	0.204	0.204
5400	0.047	2.801	0.207	0.207
7200	0.047	2.801	0.207	0.207

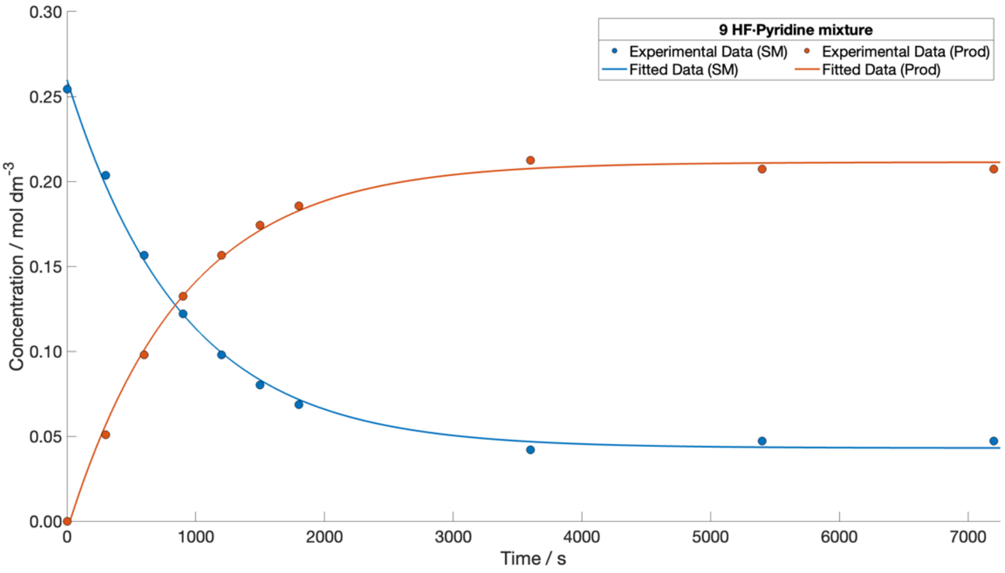

Figure S66: The kinetic run involving 9HF·py as a source of fluoride for the nucleophilic substitution of 4-nitrobenzyl bromide (SM) to 4-nitrobenzyl fluoride (Prod). Fluoride anion and bromide anion concentrations are omitted from this plot.

Table S98: The concentrations of all the different chemical species involved in the reaction between 4-nitrobenzyl bromide and the fluoride anion in 4HF·amine over a two-hour period.

Time	Measured concentration of substrate / mol dm^{-3}	Calculated concentration of fluoride / mol dm^{-3}	Measured concentration of product / mol dm^{-3}	Calculated concentration of bromide / mol dm^{-3}
0	0.254	4.059	0.000	0.000
300	0.156	3.961	0.098	0.098
600	0.094	3.899	0.160	0.160
900	0.066	3.871	0.188	0.188
1200	0.054	3.859	0.200	0.200
1500	0.048	3.853	0.206	0.206
1800	0.045	3.850	0.209	0.209
3600	0.043	3.848	0.211	0.211
5400	0.043	3.848	0.211	0.211
7200	0.043	3.848	0.211	0.211

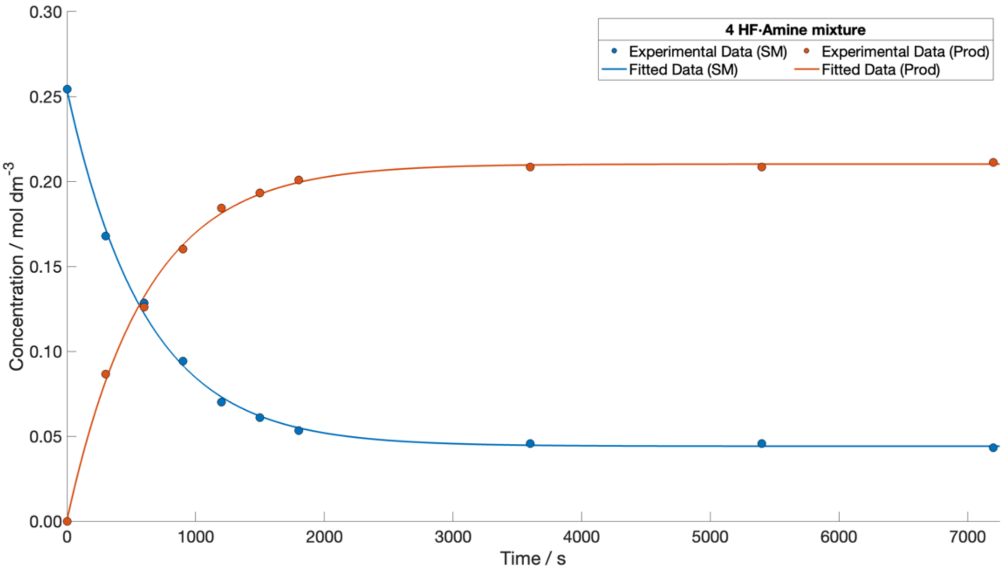

Figure S67: The kinetic run involving 4HF·amine as a source of fluoride for the nucleophilic substitution of 4-nitrobenzyl bromide (SM) to 4-nitrobenzyl fluoride (Prod). Fluoride anion and bromide anion concentrations are omitted from this plot.

Table S99: The concentrations of all the different chemical species involved in the reaction between 4-nitrobenzyl bromide and the fluoride anion in 5HF·amine over a two-hour period.

Time	Measured concentration of substrate / mol dm^{-3}	Calculated concentration of fluoride / mol dm^{-3}	Measured concentration of product / mol dm^{-3}	Calculated concentration of bromide / mol dm^{-3}
0	0.254	3.904	0.000	0.000
300	0.180	3.830	0.074	0.074
600	0.119	3.769	0.135	0.135
900	0.082	3.732	0.172	0.172
1200	0.058	3.708	0.196	0.196
1500	0.043	3.693	0.211	0.211
1800	0.034	3.684	0.220	0.220
3600	0.019	3.669	0.235	0.235
5400	0.019	3.668	0.235	0.235
7200	0.019	3.668	0.235	0.235

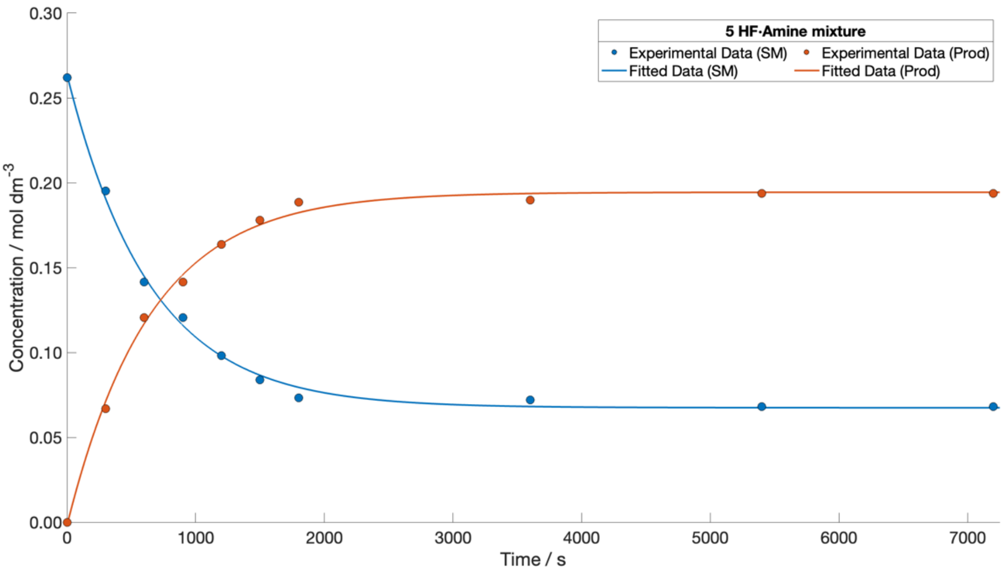

Figure S68: The kinetic run involving 5HF·amine as a source of fluoride for the nucleophilic substitution of 4-nitrobenzyl bromide (SM) to 4-nitrobenzyl fluoride (Prod). Fluoride anion and bromide anion concentrations are omitted from this plot.

Table S100: The concentrations of all the different chemical species involved in the reaction between 4-nitrobenzyl bromide and the fluoride anion in 6HF·amine over a two-hour period.

Time	Measured concentration of substrate / mol dm^{-3}	Calculated concentration of fluoride / mol dm^{-3}	Measured concentration of product / mol dm^{-3}	Calculated concentration of bromide / mol dm^{-3}
0	0.254	3.740	0.000	0.000
300	0.188	3.673	0.066	0.066
600	0.125	3.611	0.129	0.129
900	0.095	3.581	0.159	0.159
1200	0.081	3.566	0.173	0.173
1500	0.074	3.559	0.180	0.180
1800	0.070	3.556	0.184	0.184
3600	0.067	3.553	0.187	0.187
5400	0.067	3.553	0.187	0.187
7200	0.067	3.553	0.187	0.187

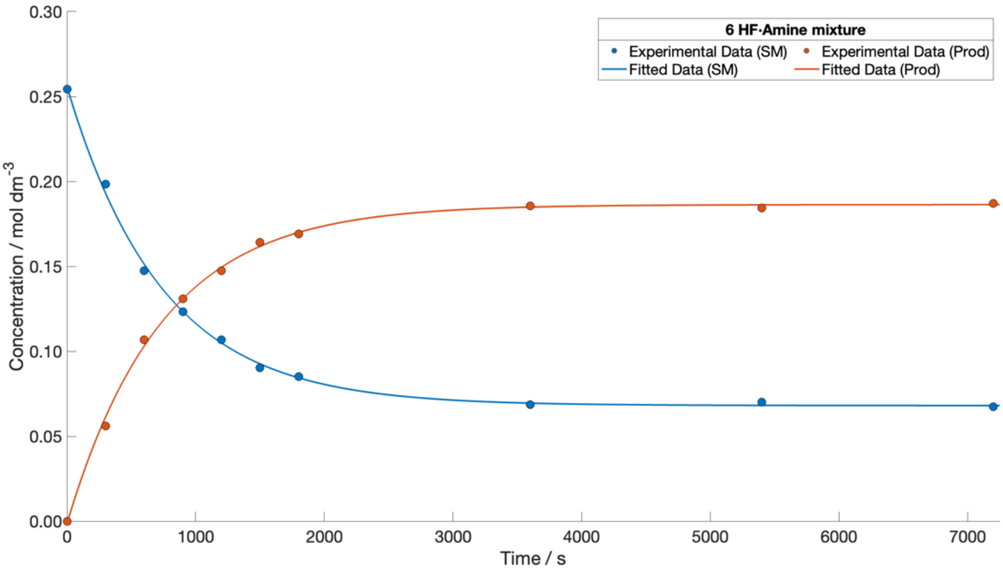

Figure S69: The kinetic run involving 6HF·amine as a source of fluoride for the nucleophilic substitution of 4-nitrobenzyl bromide (SM) to 4-nitrobenzyl fluoride (Prod). Fluoride anion and bromide anion concentrations are omitted from this plot.

Table S101: The concentrations of all the different chemical species involved in the reaction between 4-nitrobenzyl bromide and the fluoride anion in 7HF·amine over a two-hour period.

Time	Measured concentration of substrate / mol dm^{-3}	Calculated concentration of fluoride / mol dm^{-3}	Measured concentration of product / mol dm^{-3}	Calculated concentration of bromide / mol dm^{-3}
0	0.254	3.446	0.000	0.000
300	0.203	3.395	0.051	0.051
600	0.168	3.359	0.086	0.086
900	0.139	3.330	0.116	0.116
1200	0.120	3.311	0.135	0.135
1500	0.103	3.295	0.151	0.151
1800	0.094	3.286	0.160	0.160
3600	0.074	3.265	0.181	0.181
5400	0.070	3.261	0.184	0.184
7200	0.069	3.260	0.186	0.186

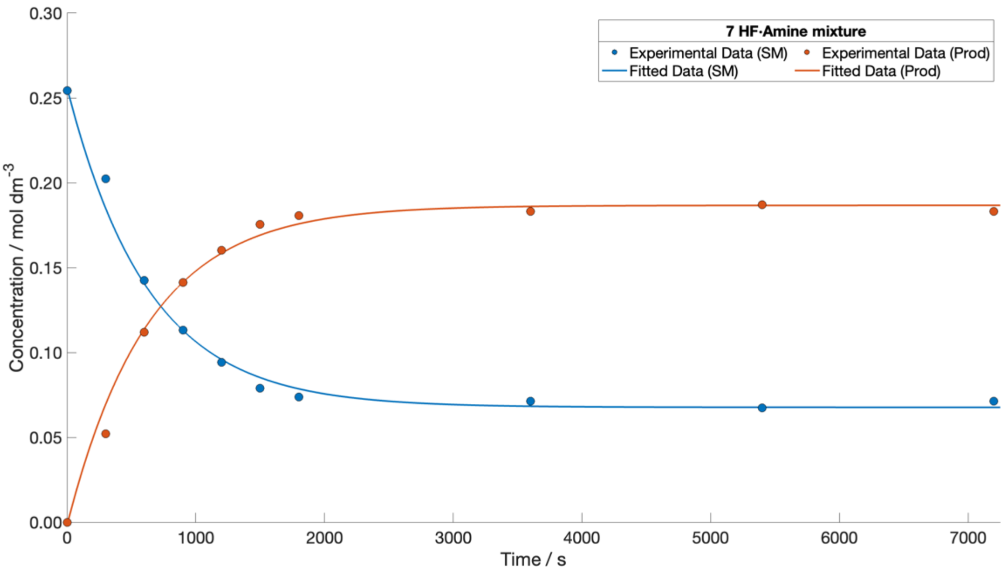

Figure S70: The kinetic run involving 7HF·amine as a source of fluoride for the nucleophilic substitution of 4-nitrobenzyl bromide (SM) to 4-nitrobenzyl fluoride (Prod). Fluoride anion and bromide anion concentrations are omitted from this plot.

Table S102: The concentrations of all the different chemical species involved in the reaction between 4-nitrobenzyl bromide and the fluoride anion in 8HF·amine over a two-hour period.

Time	Measured concentration of substrate / mol dm^{-3}	Calculated concentration of fluoride / mol dm^{-3}	Measured concentration of product / mol dm^{-3}	Calculated concentration of bromide / mol dm^{-3}
0	0.254	3.308	0.000	0.000
300	0.209	3.262	0.046	0.046
600	0.173	3.226	0.081	0.081
900	0.142	3.196	0.112	0.112
1200	0.122	3.175	0.132	0.132
1500	0.106	3.159	0.149	0.149
1800	0.095	3.149	0.159	0.159
3600	0.074	3.127	0.181	0.181
5400	0.070	3.123	0.184	0.184
7200	0.069	3.122	0.186	0.186

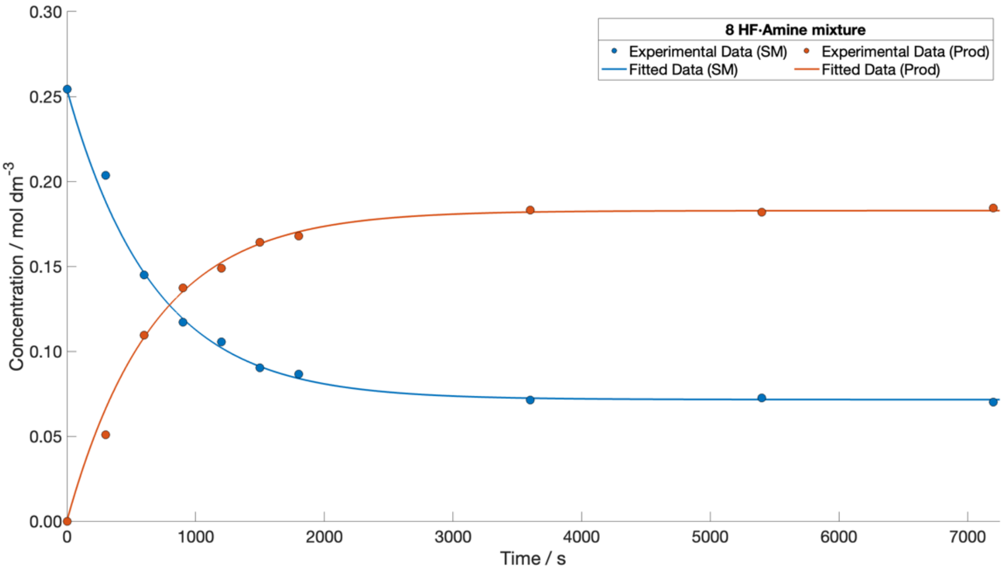

Figure S71: The kinetic run involving 8HF·amine as a source of fluoride for the nucleophilic substitution of 4-nitrobenzyl bromide (SM) to 4-nitrobenzyl fluoride (Prod). Fluoride anion and bromide anion concentrations are omitted from this plot.

Table S103: The concentrations of all the different chemical species involved in the reaction between 4-nitrobenzyl bromide and the fluoride anion in 1HF·TEA over a two-hour period.

Time	Measured concentration of substrate / mol dm^{-3}	Calculated concentration of fluoride / mol dm^{-3}	Measured concentration of product / mol dm^{-3}	Calculated concentration of bromide / mol dm^{-3}
0	0.254	5.189	0.000	0.000
300	0.012	4.947	0.242	0.242
600	0.009	4.944	0.245	0.245
900	0.008	4.942	0.247	0.247
1200	0.006	4.941	0.248	0.248
1500	0.006	4.941	0.248	0.248
1800	0.005	4.940	0.249	0.249
3600	0.006	4.941	0.248	0.248
5400	0.006	4.941	0.248	0.248
7200	0.005	4.940	0.249	0.249

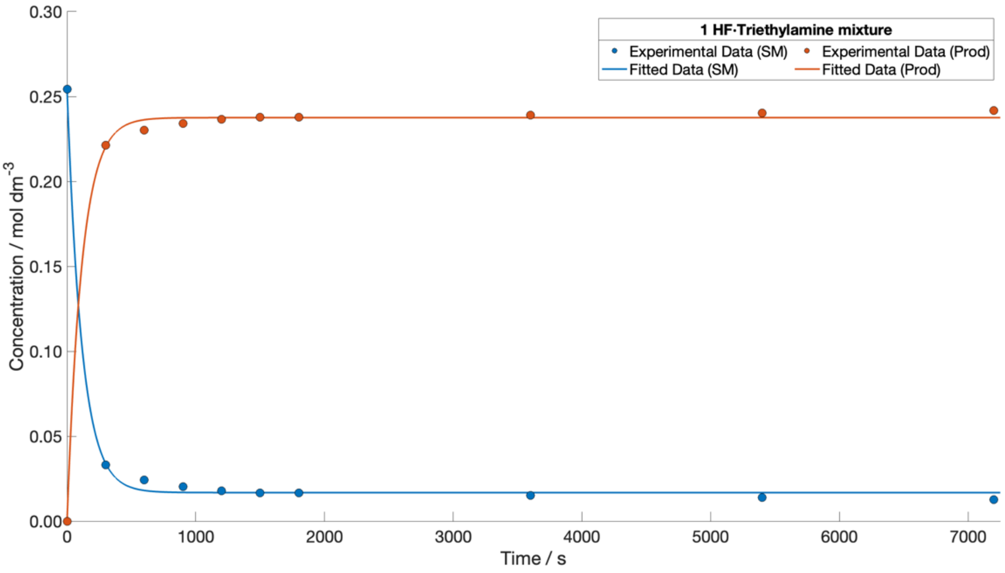

Figure S72: The kinetic run involving 1HF·TEA as a source of fluoride for the nucleophilic substitution of 4-nitrobenzyl bromide (SM) to 4-nitrobenzyl fluoride (Prod). Fluoride anion and bromide anion concentrations are omitted from this plot.

Table S104: The concentrations of all the different chemical species involved in the reaction between 4-nitrobenzyl bromide and the fluoride anion in 2HF·TEA over a two-hour period.

Time	Measured concentration of substrate / mol dm^{-3}	Calculated concentration of fluoride / mol dm^{-3}	Measured concentration of product / mol dm^{-3}	Calculated concentration of bromide / mol dm^{-3}
0	0.254	4.981	0.000	0.000
300	0.045	4.771	0.210	0.210
600	0.031	4.757	0.224	0.224
900	0.023	4.750	0.231	0.231
1200	0.019	4.746	0.235	0.235
1500	0.020	4.747	0.234	0.234
1800	0.017	4.743	0.238	0.238
3600	0.015	4.742	0.239	0.239
5400	0.014	4.741	0.240	0.240
7200	0.014	4.741	0.240	0.240

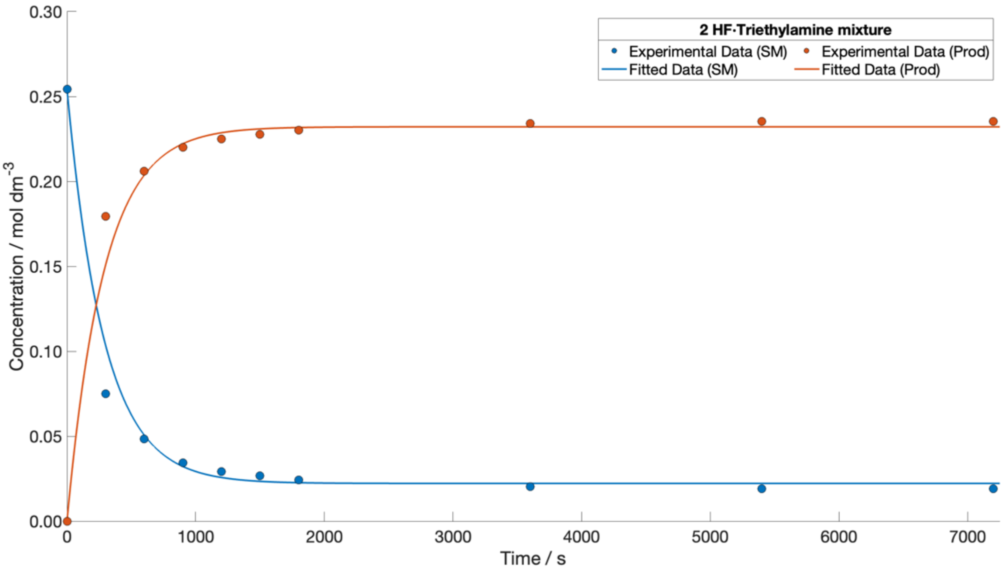

Figure S73: The kinetic run involving 2HF·TEA as a source of fluoride for the nucleophilic substitution of 4-nitrobenzyl bromide (SM) to 4-nitrobenzyl fluoride (Prod). Fluoride anion and bromide anion concentrations are omitted from this plot.

Table S105: The concentrations of all the different chemical species involved in the reaction between 4-nitrobenzyl bromide and the fluoride anion in 3HF·TEA over a two-hour period.

Time	Measured concentration of substrate / mol dm^{-3}	Calculated concentration of fluoride / mol dm^{-3}	Measured concentration of product / mol dm^{-3}	Calculated concentration of bromide / mol dm^{-3}
0	0.254	4.781	0.000	0.000
300	0.085	4.612	0.169	0.169
600	0.062	4.589	0.192	0.192
900	0.043	4.570	0.211	0.211
1200	0.039	4.566	0.215	0.215
1500	0.034	4.561	0.220	0.220
1800	0.032	4.558	0.223	0.223
3600	0.027	4.553	0.228	0.228
5400	0.027	4.553	0.228	0.228
7200	0.027	4.553	0.228	0.228

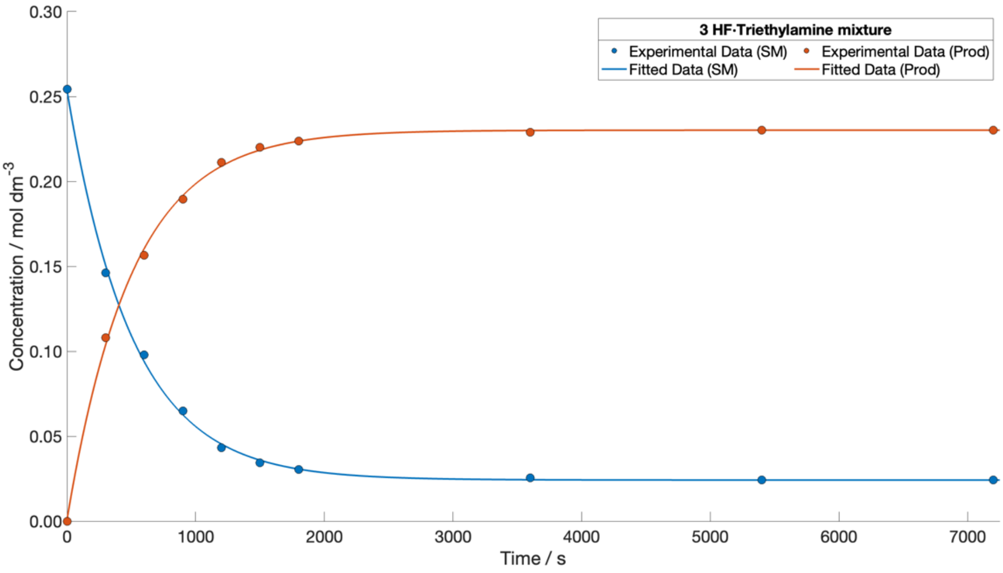

Figure S74: The kinetic run involving 3HF·TEA as a source of fluoride for the nucleophilic substitution of 4-nitrobenzyl bromide (SM) to 4-nitrobenzyl fluoride (Prod). Fluoride anion and bromide anion concentrations are omitted from this plot.

Table S106: The concentrations of all the different chemical species involved in the reaction between 4-nitrobenzyl bromide and the fluoride anion in 4HF·TEA over a two-hour period.

Time	Measured concentration of substrate / mol dm^{-3}	Calculated concentration of fluoride / mol dm^{-3}	Measured concentration of product / mol dm^{-3}	Calculated concentration of bromide / mol dm^{-3}
0	0.254	4.781	0.000	0.000
300	0.127	4.654	0.127	0.127
600	0.072	4.599	0.182	0.182
900	0.054	4.581	0.200	0.200
1200	0.048	4.575	0.206	0.206
1500	0.046	4.573	0.208	0.208
1800	0.045	4.572	0.209	0.209
3600	0.045	4.572	0.209	0.209
5400	0.045	4.572	0.209	0.209
7200	0.045	4.572	0.209	0.209

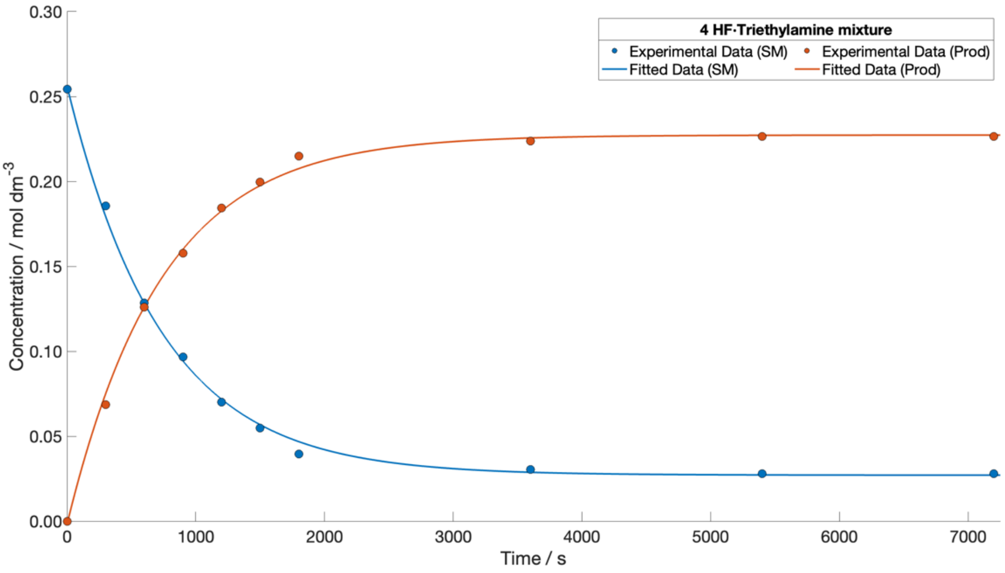

Figure S75: The kinetic run involving 4HF·TEA as a source of fluoride for the nucleophilic substitution of 4-nitrobenzyl bromide (SM) to 4-nitrobenzyl fluoride (Prod). Fluoride anion and bromide anion concentrations are omitted from this plot.

Table S107: The concentrations of all the different chemical species involved in the reaction between 4-nitrobenzyl bromide and the fluoride anion in 5HF·TEA over a two-hour period.

Time	Measured concentration of substrate / mol dm^{-3}	Calculated concentration of fluoride / mol dm^{-3}	Measured concentration of product / mol dm^{-3}	Calculated concentration of bromide / mol dm^{-3}
0	0.254	4.590	0.000	0.000
300	0.144	4.479	0.110	0.110
600	0.083	4.418	0.171	0.171
900	0.056	4.392	0.198	0.198
1200	0.045	4.380	0.209	0.209
1500	0.040	4.375	0.214	0.214
1800	0.038	4.373	0.216	0.216
3600	0.036	4.372	0.218	0.218
5400	0.036	4.372	0.218	0.218
7200	0.036	4.372	0.218	0.218

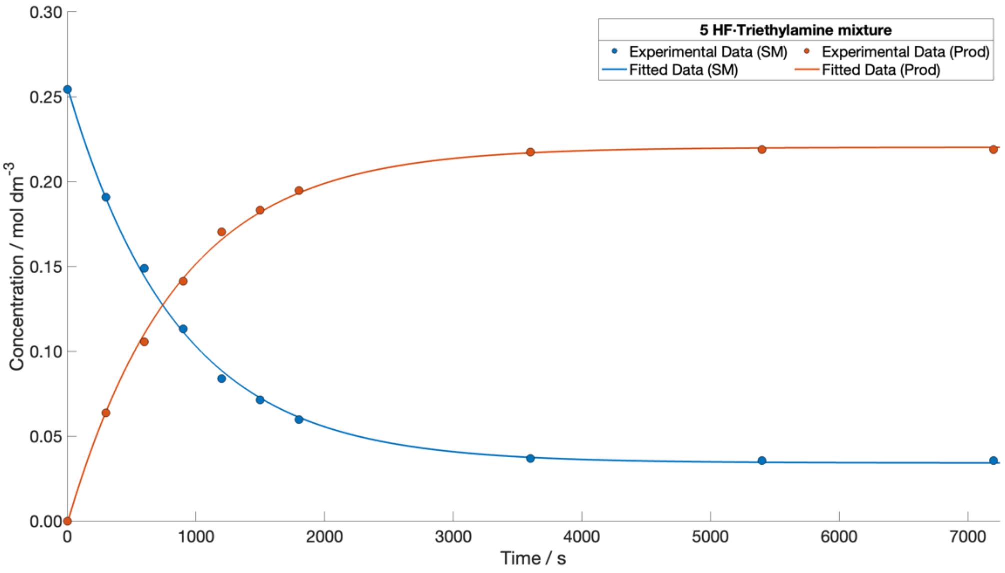

Figure S76: The kinetic run involving 5HF·TEA as a source of fluoride for the nucleophilic substitution of 4-nitrobenzyl bromide (SM) to 4-nitrobenzyl fluoride (Prod). Fluoride anion and bromide anion concentrations are omitted from this plot.

Table S108: The concentrations of all the different chemical species involved in the reaction between (*R*)-(-)-1-(bromomethyl-*d*)-4-nitrobenzene and the fluoride anion in 5HF·TEA over a two-hour period.

Time	Measured concentration of substrate / mol dm^{-3}	Calculated concentration of fluoride / mol dm^{-3}	Measured concentration of product / mol dm^{-3}	Calculated concentration of bromide / mol dm^{-3}
0	0.246	4.590	0.000	0.000
300	0.143	4.486	0.103	0.103
600	0.111	4.454	0.135	0.135
900	0.089	4.432	0.157	0.157
1200	0.071	4.415	0.175	0.175
1500	0.061	4.405	0.184	0.184
1800	0.057	4.400	0.189	0.189
3600	0.042	4.385	0.204	0.204
5400	0.037	4.380	0.209	0.209
7200	0.034	4.378	0.212	0.212

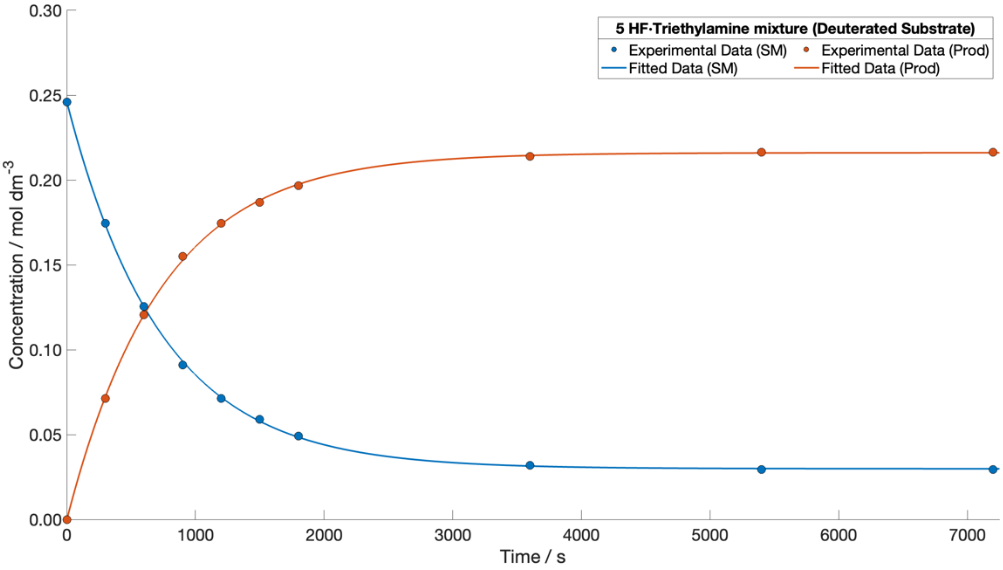

Figure S77: The kinetic run involving 5HF·TEA as a source of fluoride for the nucleophilic substitution of (*R*)-(-)-1-(bromomethyl-*d*)-4-nitrobenzene (SM) to (*S*)-(+)-1-(fluoromethyl-*d*)-4-nitrobenzene (Prod). Fluoride anion and bromide anion concentrations are omitted from this plot.

Table 109: The calculated second-order rate constants calculated through using COPASI[55]

nHF·base	k_2 / 10^3 M^{-1} s^{-1} when base = py	k_2 / 10^3 M^{-1} s^{-1} when base = amine	k_2 / 10^3 M^{-1} s^{-1} when base = TEA
1	1.67	-	-
2	0.76	-	-
3	0.33	-	0.76
4	0.23	0.40	0.49
5	0.20	0.30	0.41
6	0.20	0.27	-
7	0.17	0.22	-
8	0.16	0.20	-
9	0.15	-	-

9. Mechanistic Investigations for the Reaction of 4-Nitrobenzyl Bromide in *n*HF·base Mixtures

9.1 Synthesis of chiral 1-(bromomethyl-*d*)-4-nitrobenzenes

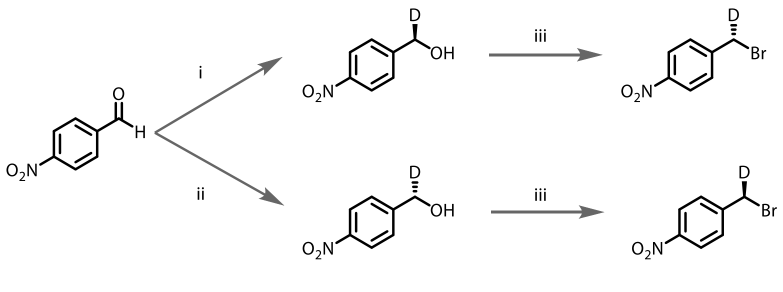

Scheme S1: The syntheses of both (*R*) and (*S*)-1-(bromomethyl-*d*)-4-nitrobenzene. Reagents and conditions: (i): RuCl(*p*-cymene)[(S,*S*-Ts-DPEN], [$^2H_2$]-formic acid, TEA, acetonitrile; (ii): RuCl(*p*-cymene)[(R,*R*-Ts-DPEN], [$^2H_2$]-formic acid, TEA, acetonitrile; (iii) Phosphorous tribromide, DCM.

9.1.1 Synthesis of (4-nitrophenyl)methan-*d*-ol

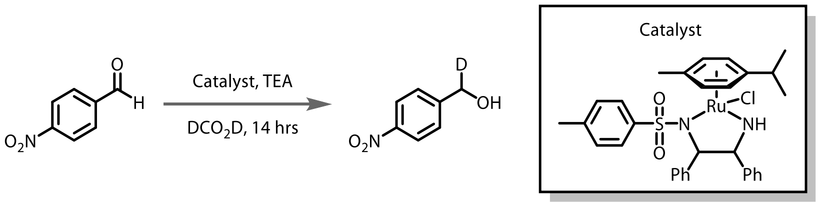

To a solution of ruthenium catalyst (58 mg, 0.09 mmol, 0.5 mol%), [$^2H_2$]-formic acid (0.94 g, 0.78 mL, 20.8 mmol, 1 eqv) and triethylamine (2.05 g, 2.82 mL, 20.2 mmol, 1 eqv) in dry, degassed acetonitrile (20 mL) was stirred at room temperature for fifteen minutes. 4-Nitrobenzaldehyde (3.05 g, 20.2 mmol, 1 eqv) was added to the reaction, which was allowed to stir for 14 hours at room temperature. The reaction was then diluted with water (30 mL) and extracted into ethyl acetate (3 x 40 mL). The organic layer was dried with magnesium sulfate, filtered, and concentrated *en vacuo*. The product was then purified using silica gel column chromatography using an eluent of pentane:ethyl acetate (30:70). All organics containing the product were then combined, dried with magnesium sulfate, filtered, and concentrated *en vacuo*, to produce a pale-yellow solid.

(*R*)-(-)-(4-nitrophenyl)methan-*d*-ol

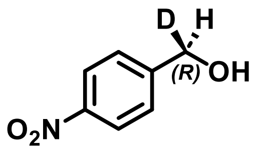

Using the ruthenium catalyst RuCl(*p*-cymene)[(S,*S*-Ts-DPEN] in the procedure for asymmetric deuteration gives the title compound as a pale-yellow solid (2.21 g, 14.3 mmol, 71% yield).

^{1}H NMR (400 MHz, CDCl$_3$): δ = 8.22 (d, *J* = 8.5 Hz, 2H), 7.47 (d, *J* = 8.5 Hz, 2H), 4.81 (s, 1H).

^{13}C {^{1}H} NMR (101 MHz, CDCl$_3$): δ = 147.91, 144.38, 129.46, 124.08, 44.37 (t, *J* = 23.5 Hz).

^{2}H NMR (61 MHz, CHCl$_3$): δ = 4.81 (d, *J* = 2.2 Hz).

HRMS (EI$^+$) calc: $[M]^+$ ($C_7H_6DNO_3$) 154.0483; measured: 154.0482 = 0.65 ppm difference.

IR (neat) v_{max}/cm^{-1}: 3513, 2976, 2867, 1601, 1505, 1409, 1334, 1107, 1061, 914, 859, 819, 728, 623, 529

$[\alpha]^{20}_{D}$ -2.45 deg mL g^{-1} dm^{-1} (*c* 6.1, THF)

(*S*)-(+)-(4-nitrophenyl)methan-*d*-ol

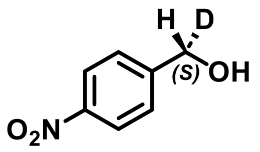

Using the ruthenium catalyst RuCl(*p*-cymene)[(S,*S*-Ts-DPEN] in the procedure for asymmetric deuteration gives the title compound as a pale-yellow solid (2.21 g, 14.3 mmol, 71% yield).

^{1}H NMR (400 MHz, CDCl$_3$): δ = 8.22 (d, *J* = 8.5 Hz, 2H), 7.47 (d, *J* = 8.5 Hz, 2H), 4.81 (s, 1H).

^{13}C {^{1}H} NMR (101 MHz, CDCl$_3$): δ = 147.91, 144.38, 129.46, 124.08, 44.37 (t, *J* = 23.5 Hz).

^{2}H NMR (61 MHz, CHCl$_3$): δ = 4.81 (d, *J* = 2.2 Hz).

$[\alpha]^{20}_{D}$ +2.45 deg mL g^{-1} dm^{-1} (*c* 6.1, THF)

Data is consistent with what is found in the literature.[56]

9.1.2 Synthesis of 1-(bromomethyl-*d*)-4-nitrobenzene

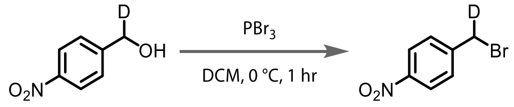

(4-Nitrophenyl)methan-*d*-ol (1.00 g, 6.5 mmol, 1 eqv) was dissolved in 30 mL of anhydrous DCM. The solution was then cooled to 0 °C, to which phosphorous tribromide (0.73 mL, 7.8 mmol, 1.2 eqv) was added dropwise over five minutes. The reaction was then allowed to occur for one hour. Saturated aqueous sodium hydrogen carbonate solution was added to the reaction mixture until the solution was neutral. The solution was then extracted using DCM (2 x 50 mL). All organic layers were then combined and dried with magnesium sulfate. Solvent was then removed *en vacuo*.

(*R*)-(-)-1-(bromomethyl-*d*)-4-nitrobenzene

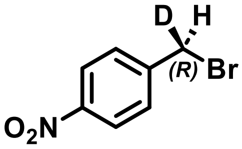

Subjecting (*S*)-(4-Nitrophenyl)methan-*d*-ol to the conditions described above to provide a pale-yellow solid (1.35 g, 6.2 mmol, 96% yield).

^{1}H NMR (400 MHz, $CDCl_3$): δ = 8.21 (d, *J* = 8.7 Hz, 2H), 7.56 (d, *J* = 8.6 Hz, 2H), 4.50 (s, 1H).

^{13}C {^{1}H} NMR (101 MHz, $CDCl_3$): δ = 147.81, 144.85, 130.06, 124.20, 31.06 (t, *J* = 23.4 Hz).

^{2}H NMR (61 MHz, $CHCl_3$): δ = 4.51 (d, *J* = 1.6 Hz).

HRMS (EI$^+$) calc: $[M]^+$ ($C_7H_5DNO_2Br$) 215.9639; measured: 215.9634 = 2.31 ppm difference.

IR (neat) v_{max}/cm^{-1}: 3080, 1937, 1724, 1609, 1531, 1346, 1205, 1159, 1103, 1012, 850, 789, 745, 582, 524, 471.

$[\alpha]^{20}_D$: -1.2 deg mL g^{-1} dm^{-1} (*c* 10.0, $CHCl_3$).

(*S*)-(+)-1-(bromomethyl-*d*)-4-nitrobenzene

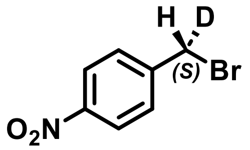

Subjecting (R)-(4-Nitrophenyl)methan-*d*-ol to the conditions described above to provide a pale-yellow solid (1.31 g, 6.1 mmol, 93% yield).

^{1}H NMR (400 MHz, $CDCl_3$): δ = 8.20 (d, *J* = 8.7 Hz, 2H), 7.54 (d, *J* = 8.6 Hz, 2H), 4.51 (s, 1H).

^{13}C {^{1}H} NMR (101 MHz, $CDCl_3$): δ = 147.80, 144.85, 130.06, 124.20, 31.06 (t, *J* = 23.4 Hz).

^{2}H NMR (61 MHz, $CHCl_3$): δ = 4.50 (d, *J* = 1.6 Hz).

HRMS (EI$^+$) calc: $[M]^+$ ($C_7H_5DNO_2Br$) 215.9639; measured: 215.9634 = 2.31 ppm difference.

IR (neat) v_{max}/cm^{-1}: 3082, 1935, 1724, 1606, 1531, 1346, 1207, 1159, 1103, 1009, 850, 789, 746, 586, 524, 475.

$[\alpha]^{20}_D$: +1.3 deg mL g^{-1} dm^{-1} (*c* 10.0, $CHCl_3$)

9.2 Synthesis of chiral 1-(fluoromethyl-*d*)-4-nitrobenzenes

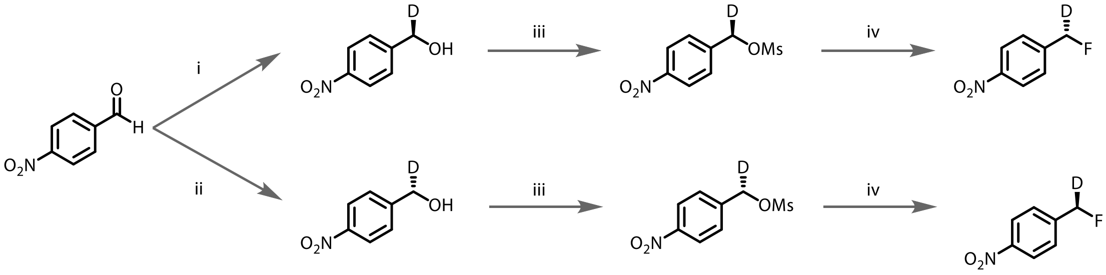

Scheme S2: The syntheses of both (*R*) and (*S*)-1-(fluoromethyl-d)-4-nitrobenzene. Reagents and conditions: (i): RuCl(*p*-cymene)[(S,*S*-Ts-DPEN], [$^{2}H_2$]-formic acid, TEA, acetonitrile; (ii): RuCl(*p*-cymene)[(R,*R*-Ts-DPEN], [$^{2}H_2$]-formic acid, TEA, acetonitrile; (iii) Methanesulfonyl chloride, TEA, DCM; (iv): caesium fluoride, *tert*-butanol.

9.2.1 Synthesis of (4-nitrophenyl)methyl-*d* methanesulfonate

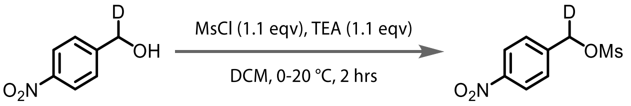

To a two-necked round-bottomed flask, alcohol (1 eqv) and triethylamine (1.1 eqv) were combined and allowed to mix for ten minutes under a nitrogen atmosphere in anhydrous DCM (0.3 M with respect to alcohol substrate). Methanesulfonyl chloride (1.1 eqv) was then added dropwise so that the temperature of the solution did not rise above 5 °C. The mixture was stirred under cooling for 30 minutes and then allowed to warm up and stir for the remaining ninety minutes. Water (30 mL) was carefully added, followed by saturated sodium hydrogen carbonate solution until solution was neutral. The organic layer was separated and washed with water (2 x 50 mL), followed by brine (1 x 50 mL). Organic layer was then separated and dried using magnesium sulfate, filtered and concentrated *en vacuo*, leaving the final mesylate product.

(*R*)-(+)-(4-nitrophenyl)methyl-*d* methanesulfonate

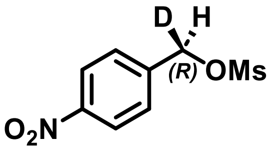

Subjecting (*R*)-(-)-(4-nitrophenyl)methan-*d*-ol to the mesylate general procedure on a 5 mmol scale, the title material was produced as a pale-yellow solid (0.81 g, 3.5 mmol, 70%). No further purification required.

^{1}H NMR (400 MHz, CDCl$_3$): δ = 8.25 (d, *J* = 8.4 Hz, 2H), 7.59 (d, *J* = 8.4 Hz, 2H), 5.31 (s, 1H), 3.05 (s, 3H).

^{13}C {^{1}H} NMR (101 MHz, CDCl$_3$): δ = 148.39, 140.68, 129.00, 124.14, 68.48 (t, *J* = 32.1 Hz), 38.53.

^{2}H NMR (61 MHz, CHCl$_3$): δ = 5.31 (d, *J* = 1.9 Hz).

HRMS (EI^{+}) calc: $[M]^{+}$ ($C_7H_6DNO_3$) 232.0264; measured: 232.0263 = 1.65 ppm difference

IR (neat) v_{max}/cm^{-1}: 3015, 1602, 1517, 1345, 1170, 1110, 975, 846, 727, 714, 520, 491, 450

$[\alpha]^{20}_{D}$ +1.01 deg mL g^{-1} dm^{-1} (*c* 9.9, DCM)

(*S*)-(+)-(4-nitrophenyl)methyl-*d* methanesulfonate

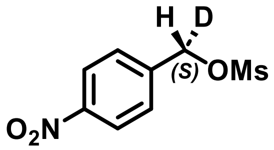

Subjecting (*S*)-(+)-(4-nitrophenyl)methan-*d*-ol to the mesylate general procedure on a 5 mmol scale, the title material was produced as a pale-yellow solid (0.96 g, 4.15 mmol, 83%). No further purification required.

^{1}H NMR (400 MHz, $CDCl_3$): δ = 8.25 (d, *J* = 8.4 Hz, 2H), 7.59 (d, *J* = 8.4 Hz, 2H), 5.31 (s, 1H), 3.05 (s, 3H).

^{13}C {^{1}H} NMR (101 MHz, $CDCl_3$): δ = 148.39, 140.68, 129.00, 124.14, 68.48 (*J* = 32.1 Hz), 38.53.

^{2}H NMR (61 MHz, $CHCl_3$): δ = 5.31 (d, *J* = 1.9 Hz).

HRMS (EI$^+$) calc: $[M]^+$ ($C_7H_6DNO_3$) 232.0264; measured: 232.0262 = 1.78 ppm difference

IR (neat) v_{max}/cm^{-1}: 3015, 1600, 1517, 1347, 1173, 1110, 971, 846, 729, 714, 528, 492, 452

$[\alpha]^{20}_D$ -1.01 deg mL g^{-1} dm^{-1} (*c* 9.9, DCM)

9.2.2 Synthesis of 1-(fluoromethyl-*d*)-4-nitrobenzene

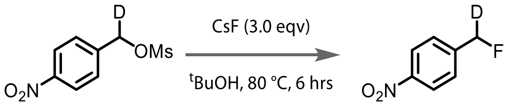

Mesylate (1 eqv) was dissolved in tert-butanol (0.25 M with respect to mesylate) and heated to 80 °C. Once reached, then caesium fluoride (3 eqv) was added with vigorous stirring. The solution was heated at 80 °C for six hours. The solution was then dried over magnesium sulfate, filtered, and dried *en vacuo* to produce a yellow oil.

(*R*)-(-)-1-(fluoromethyl-*d*)-4-nitrobenzene

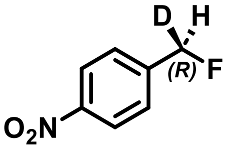

Subjecting (*S*)-(+)-1-(bromomethyl-*d*)-4-nitrobenzene to the reaction conditions on a 5 mmol scale yields the title compound as a yellow oil (0.69 g, 4.5 mmol, 89% yield).

^{1}H NMR (400 MHz, $CDCl_3$): δ = 8.26 (d, *J* = 8.6 Hz, 2H), 7.54 (d, *J* = 8.6 Hz, 2H), 5.49 (dt, *J* = 46.9 Hz, 1.78 Hz, 1H).

^{13}C {^{1}H} NMR (101 MHz, $CDCl_3$): δ = 148.16, 143.55 (d, *J* = 17.6 Hz), 127.22 (d, *J* = 7.2 Hz), 124.00, 82.68 (dt, *J* = 170.1 Hz, 23.0 Hz).

^{19}F NMR (377 MHz, $CDCl_3$): δ = -215.91 (dt, *J* = 46.8 Hz, 7.4 Hz).

^{2}H NMR (61 MHz, $CHCl_3$): δ = 5.51 (dd, *J* = 7.2 Hz, 1.87 Hz).

HRMS (EI^+) calc: $[M]^+$ ($C_7H_5DNO_2F$) 156.0440; measured: 156.0438 = 1.28 ppm difference.

IR (neat) v_{max}/cm^{-1}: 3118, 3086, 2977, 2863, 1607, 1517, 1346, 1210, 1109, 1035, 1014, 944, 857, 829, 785, 730, 718, 638, 601, 477.

$[\alpha]^{20}_{D}$: -2.06 deg mL g^{-1} dm^{-1} (*c* 9.7, $CHCl_3$).

(S)-(+)-1-(fluoromethyl-d)-4-nitrobenzene

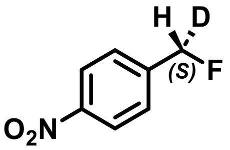

Subjecting (R)-(-)-1-(bromomethyl-d)-4-nitrobenzene to the reaction conditions on a 5 mmol scale yields the title compound as a yellow oil (0.69 g, 4.5 mmol, 89% yield).

^{1}H NMR (400 MHz, $CDCl_3$): δ = 8.27 (d, J = 8.6 Hz, 2H), 7.54 (d, J = 8.6 Hz, 2H), 5.51 (dt, J = 46.9 Hz, 1.78 Hz, 1H).

^{13}C {^{1}H} NMR (101 MHz, $CDCl_3$): δ = 148.09, 143.58 (d, J = 17.6 Hz), 127.18 (d, J = 7.2 Hz), 124.03, 82.78 (dt, J = 170.3 Hz, 22.9 Hz).

^{19}F NMR (377 MHz, $CDCl_3$): δ = -215.91 (dt, J = 47.0 Hz, 7.3 Hz).

^{2}H NMR (61 MHz, $CHCl_3$): δ = 5.51 (dd, J = 7.2 Hz, 1.87 Hz).

HRMS (EI$^+$) calc: $[M]^+$ ($C_7H_5DNO_2F$) 156.0440; measured: 156.0438 = 1.28 ppm difference.

IR (neat) v_{max}/cm^{-1}: 3118, 3086, 2977, 2863, 1607, 1517, 1346, 1210, 1109, 1035, 1014, 944, 857, 829, 785, 730, 718, 638, 601, 477.

$[\alpha]^{20}_{D}$: +1.98 deg mL g^{-1} dm^{-1} (c 9.7, $CHCl_3$).

9.3 Polarimetry measurements

The reaction between (R)-1-(bromomethyl-*d*)-4-nitrobenzene and 5HF·TEA was monitored through polarimetry. The same reaction as reported in section 7 was followed, but with slight modification. For each measurement, the aliquot was quenched in saturated aqueous sodium hydrogen carbonate. The organic layer was then separated, dried over magnesium sulfate, and concentrated *en vacuo*. The residual was then dissolved in chloroform (1 mL), and the optical rotation was measured.

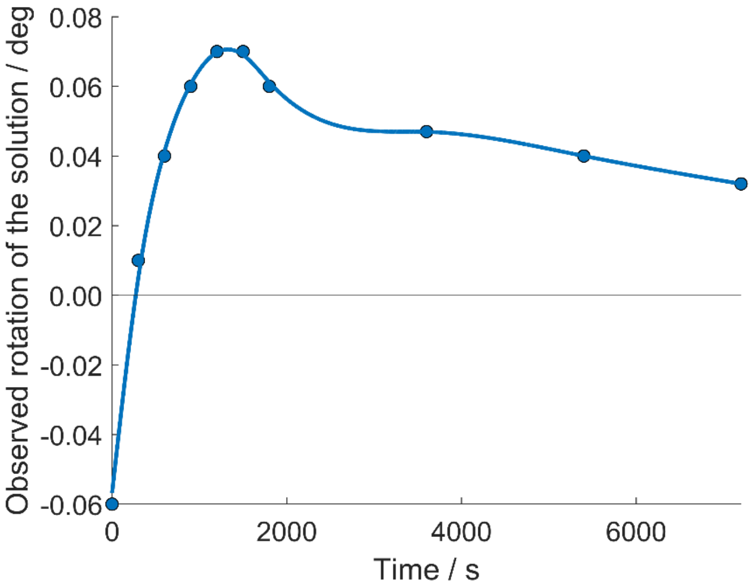

Figure S78: The observed optical rotation determined for the reaction between (R)-1-(bromomethyl-*d*)-4-nitrobenzene and the fluoride anion in the 5HF·TEA mixture.

9.4 Kinetic isotope effect measurement

Further evidence to support the proposed bimolecular substitution mechanism was obtained by measuring the kinetic isotope effect (KIE). The rate obtained when using both 4-nitrobenzyl bromide and (R)-1-(bromomethyl-*d*)-4-nitrobenzene in a reaction with fluoride anion in the 5HF·TEA mixture. The kinetic plots for the two substrates are shown in **Figure 78**. By taking the ratio of the initial rates obtained for the reaction, a KIE of 1.04 is obtained, which is indicative of an S_N2 mechanism occurring when using 4-nitrobenzyl bromide.

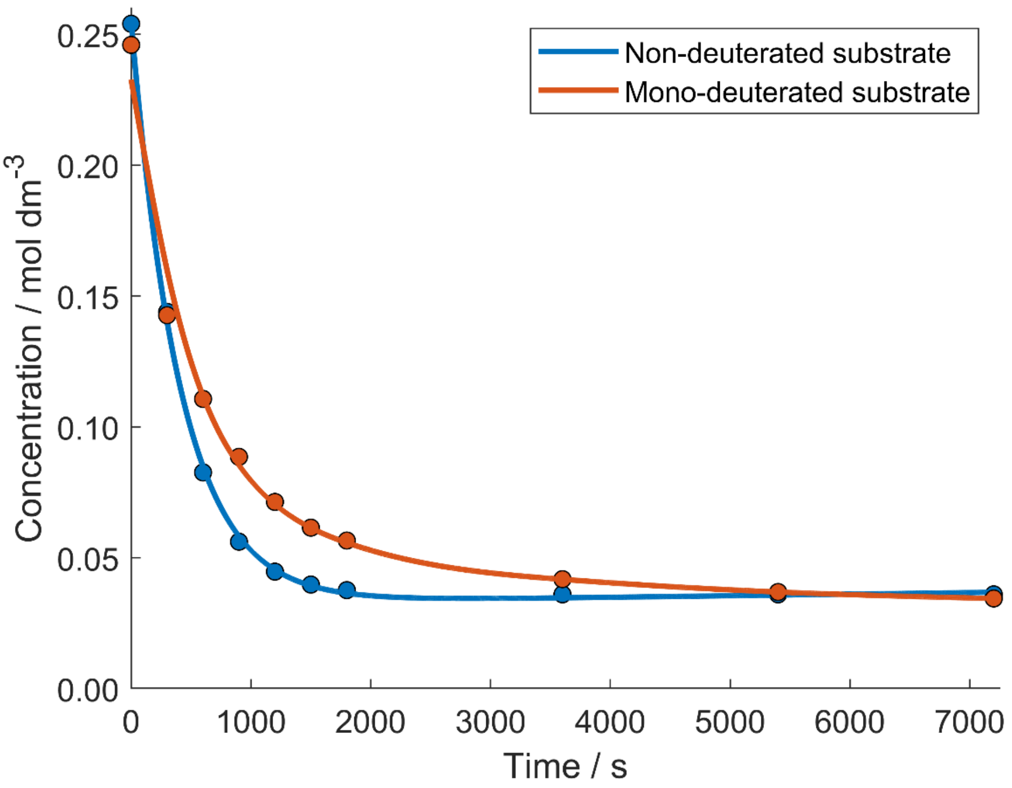

Figure S79: The two kinetic profiles obtained using 4-nitrobenzyl bromide (non-deuterated substrate) and (R)-1-(bromomethyl-*d*)-4-nitrobenzene (mono-deuterated substrate).

10. pK_a Measurements of Bases in *n*HF·base Mixtures

The method for determining the pK_{BH^+} has been reported previously in the literature.[57] A stock solution of perfluorobenzoic acid (PFBA; 0.0661 м in DCM) was first produced, and an ^{19}F NMR of this solution was recorded. Once recorded, the 1HF·base solutions were produced, as described earlier in the SI. Along with these two mixtures, a 1:1 combination of these two mixtures was also investigated, which is labelled "1HF·amine". Each of the three 1HF·base mixtures were combined with DCM (0.4 mL) to produce a 0.083 м (5 equiv) solution, and this was added to a dry and clean NMR tube. To this, 0.15 mL of the PFBA stock solution was added, and the solutions were thoroughly mixed to obtain a homogenous solution. The ^{19}F NMR of this solution was then recorded, and the signal associated with the *para* fluorine of PFBA was compared to the same signal recorded for the stock solution. The change in fluorine chemical shift for the fluorine signal (Δδ) can be related to the pK_{BH^+} according to **Equation S34,** as defined in the literature:[57]

$$pK_a = 1.0333(\Delta\delta) - 2.3732 \qquad \textbf{Equation S34}$$

11. Comparing Basicity and Nucleophilicity of Fluoride Anion

11.1 Synthesis of 3-(4-fluorophenyl)propyl 4-nitrobenzenesulfonate

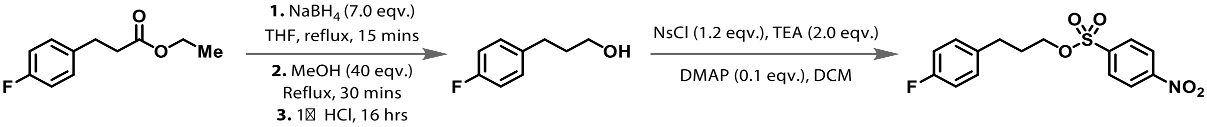

Sodium borohydride (8.10g, 7 equiv, 213 mmol) was suspended in THF (130 mL) and the respective methyl ester (6.00 g, 1 equiv, 31 mmol) was added dropwise over 20 minutes. The resulting mixture was heated to reflux and stirred for 15 minutes. Then, methanol (45 mL) was slowly added dropwise. Effervescence was observed as the methanol was initially added to the THF solution. After the completion of addition, the reaction was heated under reflux and stirred for 30 minutes. Solvent was the removed by rotary evaporation and a white paste left in the flask that was then dissolved in 150 mL of 1.0 м HCl, and the solution stirred overnight. The resulting liquid was neutralized by addition of 2.0 м NaOH and then extracted with DCM and organic layer. The solution was then dried over magnesium sulfate, filtered, and concentrated *en vacuo* to produce the corresponding alcohol, which was used to the next step without further purification.

The alcohol was dissolved in DCM, and to this triethylamine (4.5 mL, 71 mmol, 2.0 equiv) and DMAP (0.37g, 3.1 mmol, 0.1 equiv) were added. The solution was cooled to 0 °C and allowed to stir for 10 minutes. After this 4-nitrobenzenesulfonyl chloride (8.13g, 37 mmol, 1.2 equiv) was added portion wise over 15 minutes. The reaction mixture was stirred at room temperature for 12 hours and then quenched with water. The organic layer was washed by brine, dried over magnesium sulfate, filtered, and concentrated under reduced pressure to produce a yellow oil. Ethanol was added to the solution, resulting in crystals forming in the bottom of the flask. The solution was filtered and washed with ethanol to obtain white crystals of the product (5.7g, 16.8 mmol, 55%).

^{1}H NMR (400 MHz, CD_2Cl_2): δ = 8.40 (d, *J* = 8.9 Hz, 2H), 8.10 (d, *J* = 8.9 Hz, 2H), 7.14 – 7.04 (m, 2H), 6.99 – 6.89 (m, 2H), 4.12 (t, *J* = 6.2 Hz, 2H), 2.70 – 2.62 (m, 2H), 2.05 – 1.93 (m, 2H).

^{13}C {^{1}H} NMR (101 MHz, CD_2Cl_2): δ = 161.83 (d, *J* = 243.6 Hz), 151.22, 142.12, 136.41 (d, *J* = 3.2 Hz), 130.21 (d, *J* = 7.8 Hz), 129.64, 124.89, 115.54 (d, *J* = 21.1 Hz), 71.34, 30.91, 30.83.

^{19}F NMR (377 MHz, CD_2Cl_2): δ = -119.88 (tt, *J* = 8.8, 5.4 Hz).

HRMS (EI$^+$) calc: $[M]^+$ ($C_{15}H_{14}NO_5SF$) 339.0571; measured: 339.0565 = 1.77 ppm difference.

IR (neat) ν_{max}/cm^{-1}: 3121, 3105, 3072, 3047, 3042, 2992, 2946, 2922, 2892, 2865, 1608, 1531, 1509, 1467, 1446, 1403, 1360, 1320, 1303, 1292, 1218, 1182, 1109, 1095, 1051, 1013, 956, 868, 845, 831, 791, 738, 716, 703, 682, 615, 567, 554, 530, 509, 466, 426, 417.

11.2 Selectivity reaction

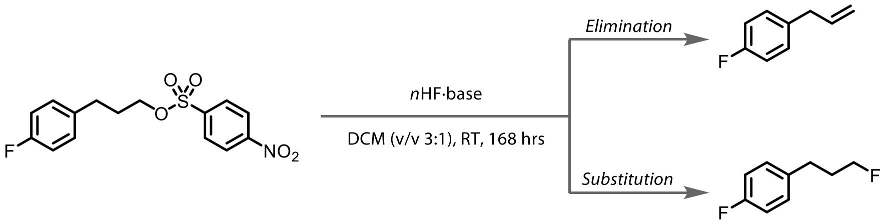

The HF mixtures were first prepared in cooled 20 mL HDPE vial (3 mL). The mixture was mixed for thirty minutes to ensure the solutions were homogenous. Then a pre-prepared 0.5 M solution of 3-(4-fluorophenyl)propyl 4-nitrobenzenesulfonate in DCM was added to each HF mixture (1 mL). Each HDPE vial was then allowed to stir for 168 hours. A long reaction time was used, as the rate of reaction was slow for this substrate. After 168 hours, an aliquot (0.3 mL) was taken from each HDPE vial and placed into an NMR liner, which was then placed inside an NMR tube charged with a solution of hexafluorobenzene in DCM (0.1 M). ^{19}F NMR spectra were then recorded to obtain the reaction profile. Concentrations of each species were then calculated using the integral of the signals for both 1-allyl-4-fluorobenzene and 1-fluoro-4-(3-fluoropropyl)benzene. The conversion for each reaction is low (see **Table S110 – S112**), which is to be expected for this type of substrate.

Table S110: The conversion of 3-(4-fluorophenyl)propyl 4-nitrobenzenesulfonate in *n*HF·py to both 1-fluoro-4-(3-fluoropropyl)benzene and 1-allyl-4-fluorobenzene.

***n*HF·py**	**% yield for substitution product**	**% yield for elimination product**	**% selectivity for substitution product**	**% selectivity for elimination product**
1HF·py	2.5	1.5	62	38
2HF·py	1.4	0.3	84	16
3HF·py	1.0	0.0	99	1
4HF·py	0.9	0.0	100	0
5HF·py	0.8	0.0	100	0
6HF·py	0.6	0.0	100	0
7HF·py	0.5	0.0	100	0
8HF·py	0.5	0.0	100	0
9HF·py	0.4	0.0	100	0

Table S111: The conversion of 3-(4-fluorophenyl)propyl 4-nitrobenzenesulfonate in *n*HF·TEA to both 1-fluoro-4-(3-fluoropropyl)benzene and 1-allyl-4-fluorobenzene.

***n*HF·TEA**	**% yield for substitution product**	**% yield for elimination product**	**% selectivity for substitution product**	**% selectivity for elimination product**
3HF·TEA	5.7	0.8	87	13
4HF·TEA	3.0	0.1	97	3
5HF·TEA	1.6	0.0	100	0

Table S112: The conversion of 3-(4-fluorophenyl)propyl 4-nitrobenzenesulfonate in *n*HF·amine to both 1-fluoro-4-(3-fluoropropyl)benzene and 1-allyl-4-fluorobenzene.

***n*HF·amine**	**% yield for substitution product**	**% yield for elimination product**	**% selectivity for substitution product**	**% selectivity for elimination product**
4HF·py	0.98	0.02	98	2
5HF·py	0.80	0.00	100	0
6HF·py	0.80	0.00	100	0
7HF·py	0.70	0.00	100	0
8HF·py	0.60	0.00	100	0

12. Experiments Involving Tetramethylammonium Fluoride (TMAF)

12.1 Nucleophilicity of fluoride anion in TMAF

We calculated the nucleophilicity of tetramethylammonium fluoride (TMAF) to compare to the HF mixtures being considered. We chose to use TMAF as a source of "naked" fluoride compared to other tetraalkylammonium fluoride salts to avoid degradation pathways of the cation. For example, it is known that the tetrabutylammonium cation can undergo a Hoffman elimination at room temperature, generating the bifluoride anion.[58] This would affect the value obtained when comparing the rate constants to the HF mixtures, and not truly represent the nucleophilicity of the fluoride anion. With TMAF, no such elimination can occur.

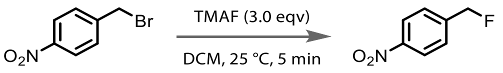

To an oven dried Schlenk tube in a glove box, anhydrous tetramethylammonium fluoride (0.14 g, 1.5 mmol, 3.0 eqv) was added with 4-nitrobenzyl bromide (0.11 g, 0.5 mmol, 1.0 eqv) and a magnetic stir bar. The Schlenk tube was then sealed and transferred to a Schlenk line, and once purged anhydrous DCM (0.5 mL) was added. After 5 minutes, an aliquot was taken for ^{1}H NMR analysis and a quantitative yield was obtained of the benzyl fluoride.

Based on the result obtained, the high nucleophilicity of the fluoride anion results in a rate constant that cannot be accurately obtained using this method. No equilibrium was observed, which is expected as the nucleophilicity of the bromide anion will be lower than that of the fluoride anion when considering polar aprotic solvents such as DCM.

The minimum value of the rate constant can be obtained, however. Through a dilution study, the NMR instrument that was used had a minimum detection threshold of 0.375 mᴍ. Using COPASI an irreversible reaction can be simulated with different rate constants (see **Figure S80**), and it was found that for a solution to have 4-nitrobenzyl bromide at a concentration of 0.375 mᴍ, the minimum value for the calculated constant is 5.2×10^{-3} $\mathrm{M}^{-1}\mathrm{s}^{-1}$. As no 4-nitrobenzyl bromide was detected, the reaction was modelled as an irreversible reaction in the COPASI software.

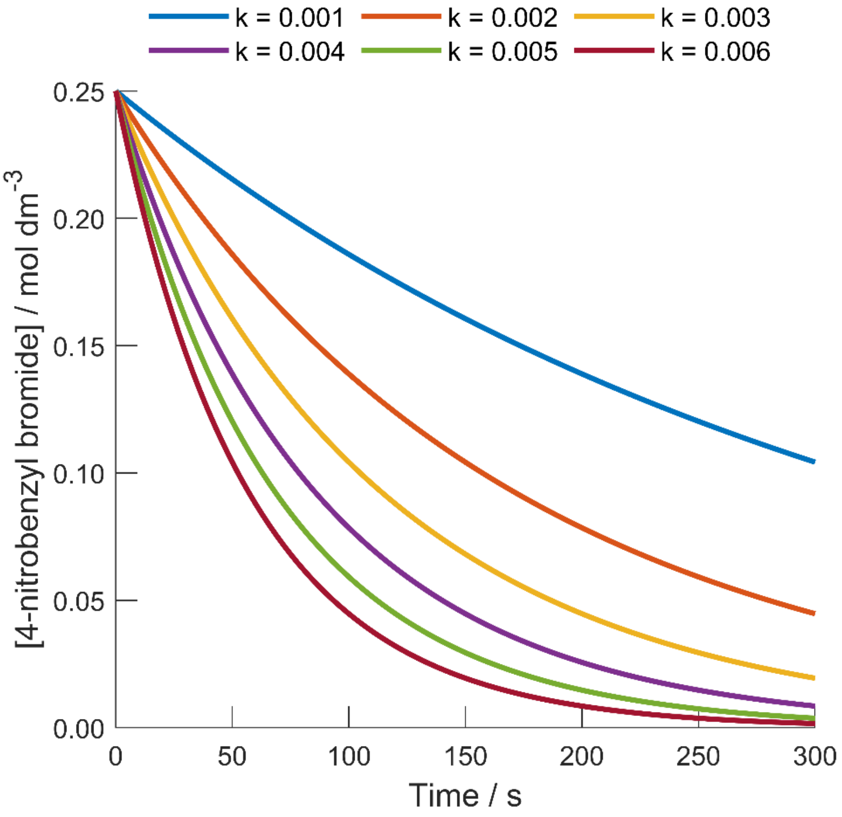

Figure S80: The simulated curves obtained for different rate constants calculated by COPASI for the depletion of 4-nitrobenzyl bromide.

We also calculated the nucleophilicity of the fluoride anion according to **Equations S22 – S25**. The structure of TMAF was optimised at the DFT/LC-PBE/def2-TZVPPD+CPCM(DCM) level of theory. Stationary points after the optimisation calculation were characterised through the harmonic vibrational frequencies, where ground states can be identified through a lack of imaginary vibrational frequencies. The parameters required to calculate the nucleophilicity of the fluoride anion were obtained as described in Section 4.7. **Table S113** and **Table S114** shows that for all computational methods for calculating nucleophilicity, TMAF features far more nucleophilic fluoride (compare with **Table S48** and **Table S49**) This supports the proposed rate constant for the S_N2 reaction being greater than for any of the HF mixtures being considered within. We rationalise that the increase in nucleophilic character comes from weaker interactions with the cation in solution.

Table S113: The calculated nucleophilicities of TMAF at the DFT/LC-PBE/def2-TZVPPD+CPCM(DCM) level of theory using local descriptors. For TMAF, $\bar{I}(r)$ = 12.03 eV; $E_{att}(r)$ = 0 eV

N	N’	N’’	N’’’
-12.03	0.67	1.66	1.48

Table S114: The calculated nucleophilicities of TMAF at the DFT/LC-PBE/def2-TZVPPD+CPCM(DCM) level of theory using global descriptors. For TMAF, HOMO = -10.91 eV; LUMO = 1.61 eV

N	N’	N’’	N’’’
-10.91	1.16	2.10	2.07

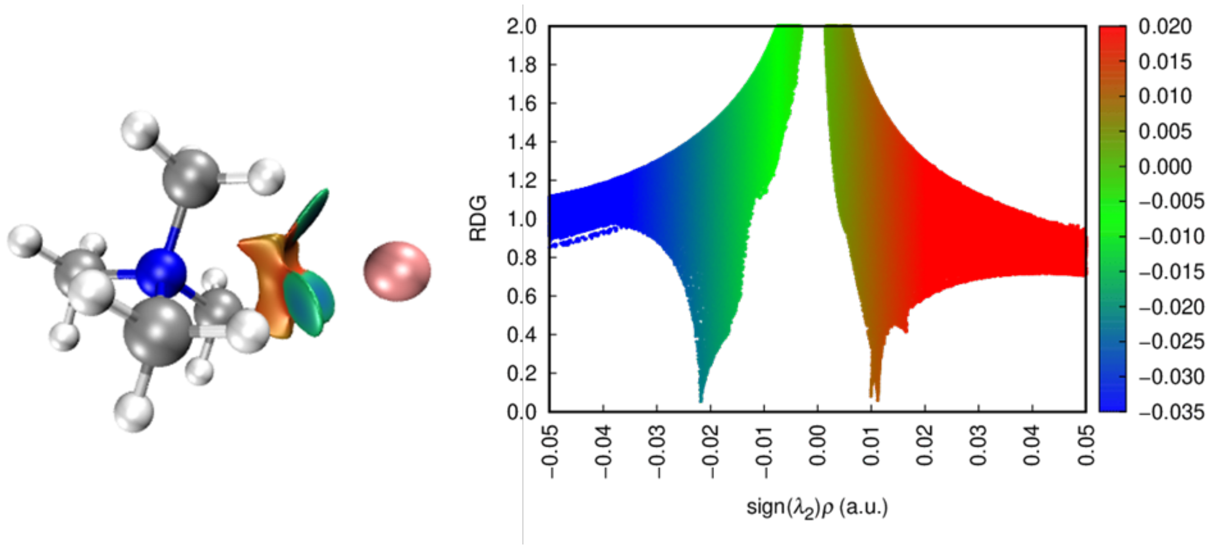

Figure S81: The RDG-NCI plot and RDG-NCI map for TMAF. Geometry was optimised at DFT/LC-PBE/def2-TZVPPD+CPCM(DCM)

Table S115: The calculated total binding energy associated with the fluoride interacting with the protonated base.

Compound	Total binding energy / kcal mol^{-1}
TMAF	-24.9
1HF·TEA	-48.4
1HF·py	-51.3*

***** For 1HF·py, the fluorine is modelled as HF rather than fluoride, and so the interaction energy of the H-F bond is reported here (ρBCP = 0.233 e $Å^{-3}$)

12.2 Basicity of fluoride anion in TMAF

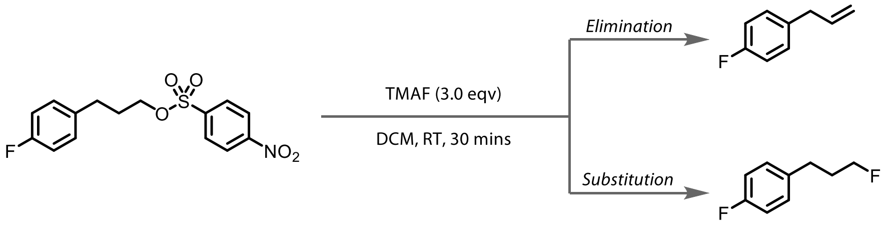

To an oven dried Schlenk tube in a glove box, anhydrous tetramethylammonium fluoride (0.14 g, 1.5 mmol, 3.0 eqv) was added with 3-(4-fluorophenyl)propyl 4-nitrobenzenesulfonate (0.17 g, 0.5 mmol, 1.0 eqv) and a magnetic stir bar. The Schlenk tube was then sealed and transferred to a Schlenk line, and once purged anhydrous DCM (2 mL) was added. After 30 minutes, an aliquot was taken for ^{19}F NMR analysis, and a quantitative yield was obtained of the substitution product 1-fluoro-4-(3-fluoropropyl)benzene. It should be noted that over this period, DCM also reacts with TMAF to produce fluorochloromethane (δ_F = -171.41 ppm, t J = 48 Hz) and difluoromethane (δ_F = -144.58, t, J = 52 Hz).

"Naked" fluoride sources are known to be highly basic as well as nucleophilic, however the basic character is not observed with this substrate. Low ratios of *n*HF·base mixtures however do show the basic nature of the fluoride anion. Our reasoning for this apparent contradiction is due to the low activation barrier for the S_N2 reaction to occur when using TMAF compared to that of the elimination reaction. When considering less nucleophilic sources of fluoride, the activation barrier increases for the S_N2 reaction and is now comparable with that of the elimination pathway, resulting in both elimination and substitution pathways becoming viable. However, it is still energetically favourable to undergo the S_N2 reaction, which is why in selectivity experiments involving this substrate, the S_N2 product is the major component.

We also calculated the basicity of the fluoride anion in TMAF, as shown in **Table S112**. The calculated metrics for basicity indicate that the fluoride anion in TMAF is more basic than any *n*HF·base combination, which is to be expected from a "naked" source of fluoride.

Table S112: The calculated proton affinity (PA) and gas phase basicity (GPB) for TMAF when allowing for a complete optimisation and constrained optimisation of the protonated fluoride interacting with the tetramethylammonium cation.

	Full optimisation	Constrained optimisation
Proton affinity / kcal mol^{-1}	279.12	270.88
Gas phase basicity / kcal mol^{-1}	273.84	263.77

13. Cost Analysis of Nucleophilic Fluorination Reagents

Costs reported in the analysis reported in **Table S113** were recorded on the 31st of March 2025. When performing the cost analysis, the largest available amounts were used for each nucleophilic fluorination reagent.

Table S113: Cost comparison of different nucleophilic fluorinating reagents

Compound	Amount / g	Supplier	Price per mol of fluoride / £
DAST	25	Sigma Aldrich	2030.99
TMAF	5	Sigma Aldrich	5197.21
TBAF·$3H_2O$	250	Sigma Aldrich	837.99
TBAF (1.0 M in THF)	2257.5	Sigma Aldrich	113.39
CsF	100	Sigma Aldrich	186.84
AgF	25	Sigma Aldrich	1715.23
LiF	50	Sigma Aldrich	191.96
KF	12000	Sigma Aldrich	4.89
KHF_2	2500	Sigma Aldrich	3.80
NH_4F	25000	Sigma Aldrich	7.81
Hydrogen fluoride gas (water < 200 ppm)	42000	Air Liquide	0.58
Olah's reagent	100	Sigma Aldrich	29.66
TREAT-HF	500	FluoroChem	79.85

14. NMR Spectra for Novel Compounds

(4-Nitrophenyl)methan-*d*-ol

^{1}H NMR (400 MHz, $CDCl_3$)

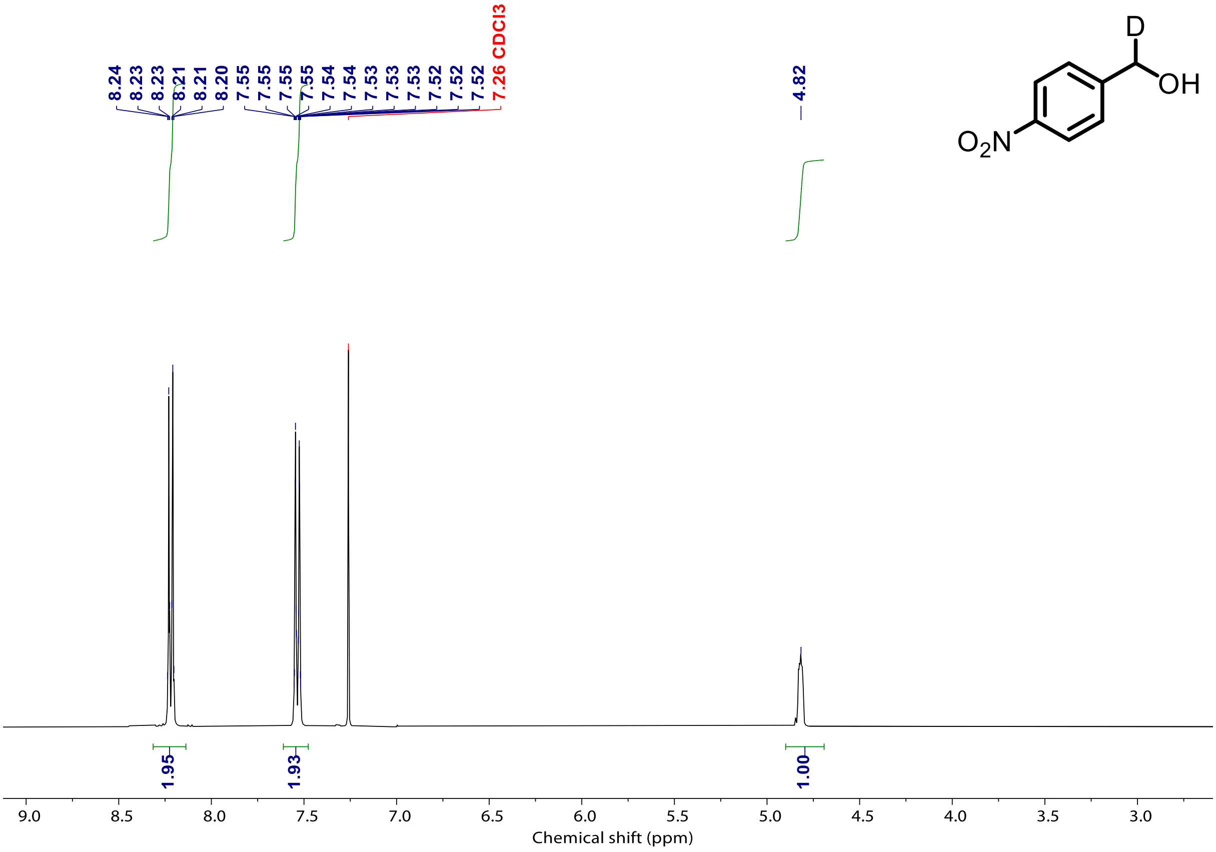

(4-Nitrophenyl)methan-*d*-ol

^{13}C NMR (101 MHz, $CDCl_3$)

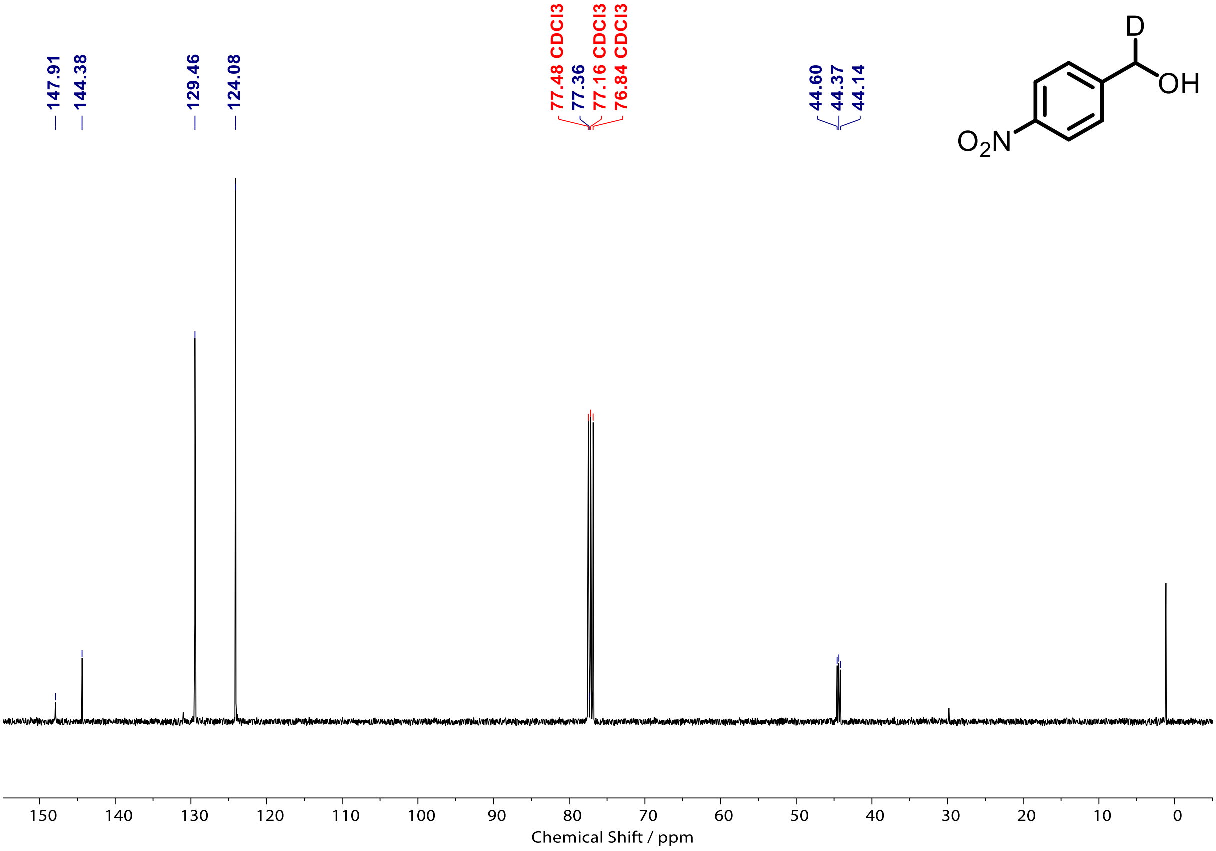

(4-Nitrophenyl)methan-*d*-ol

^{2}H NMR (61 MHz, $CHCl_3$)

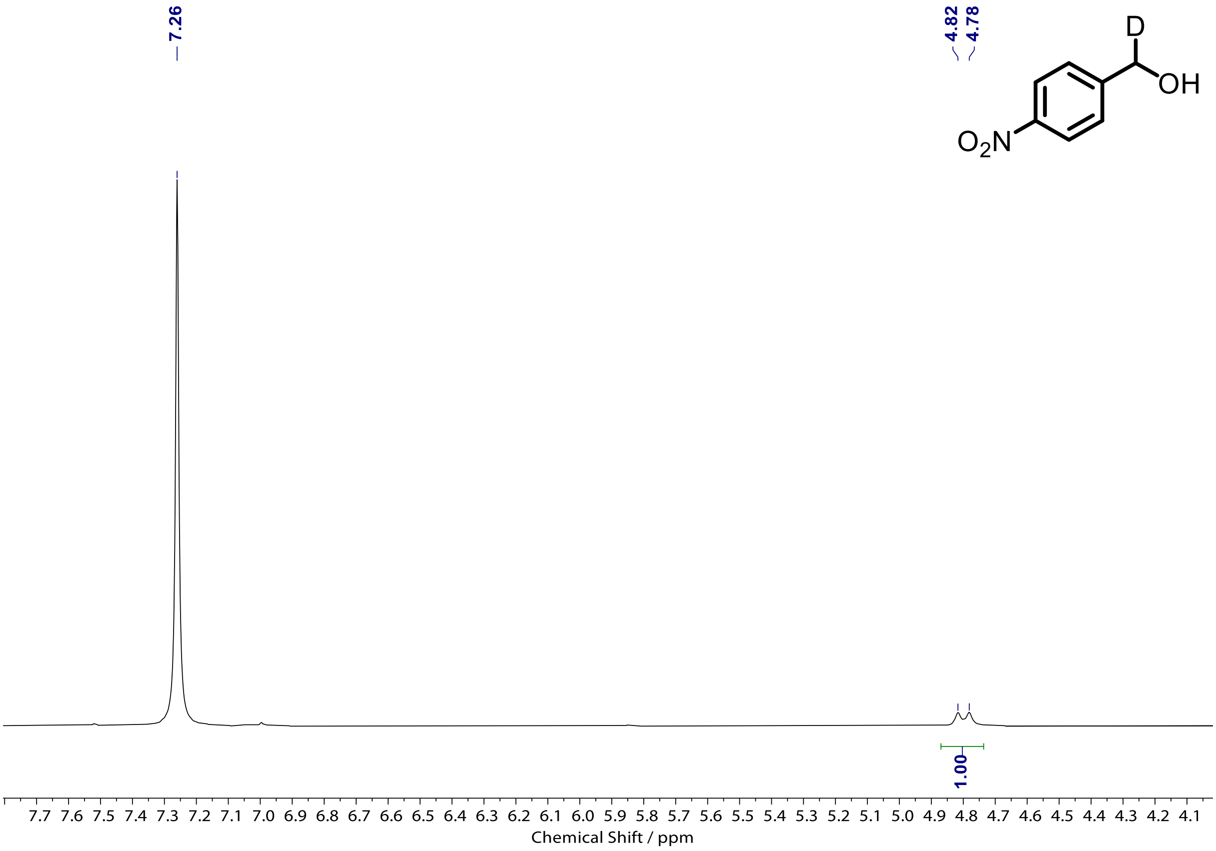

1-(Bromomethyl-*d*)-4-nitrobenzene

^{1}H NMR (400 MHz, $CDCl_3$)

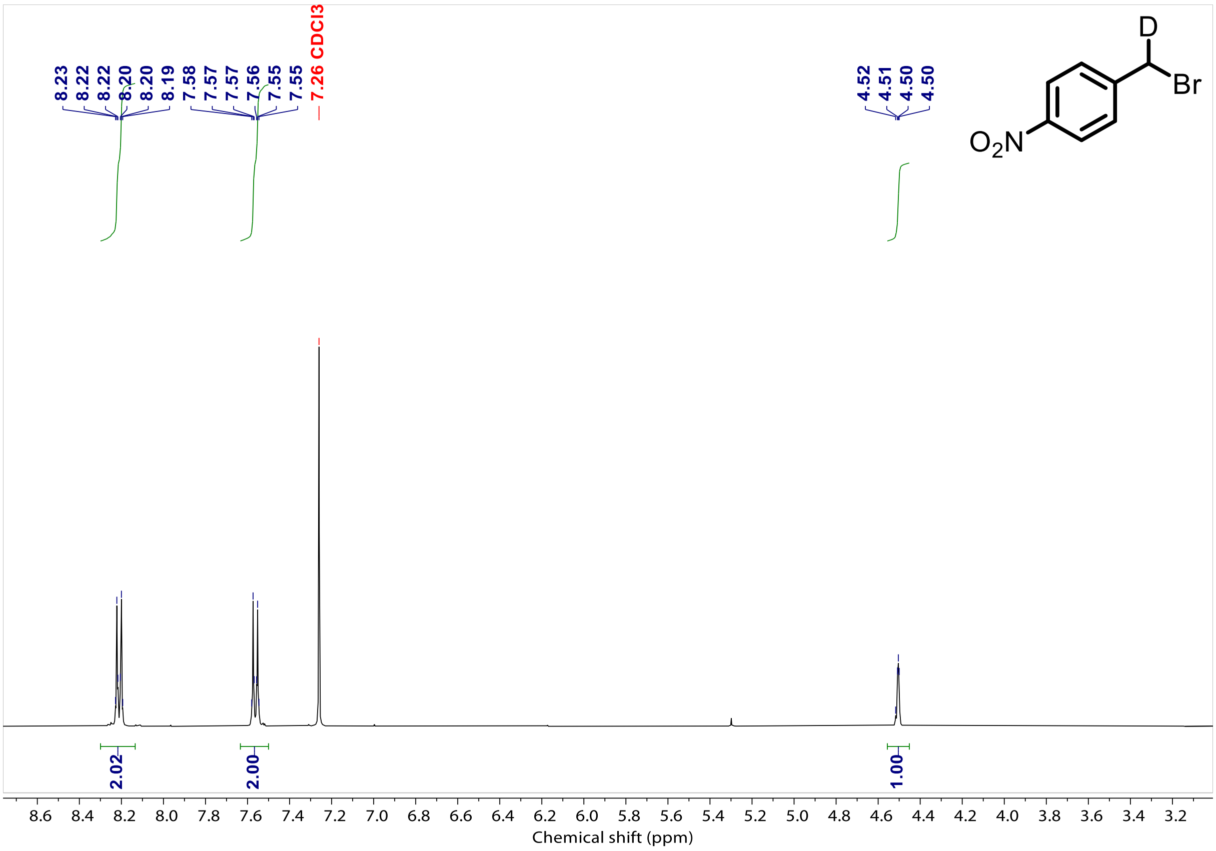

1-(Bromomethyl-*d*)-4-nitrobenzene

^{13}C NMR (101 MHz, $CHCl_3$)

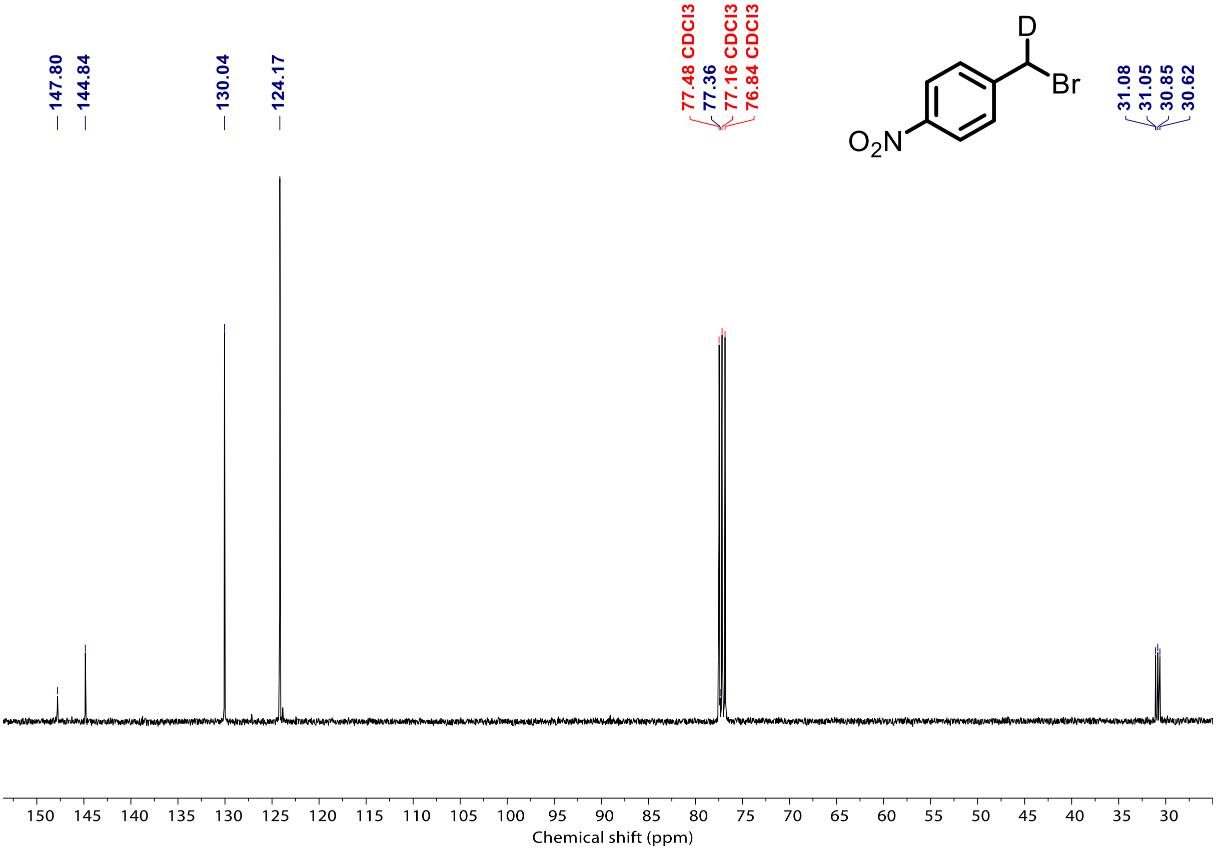

1-(Bromomethyl-*d*)-4-nitrobenzene

^{2}H NMR (61 MHz, $CHCl_3$)

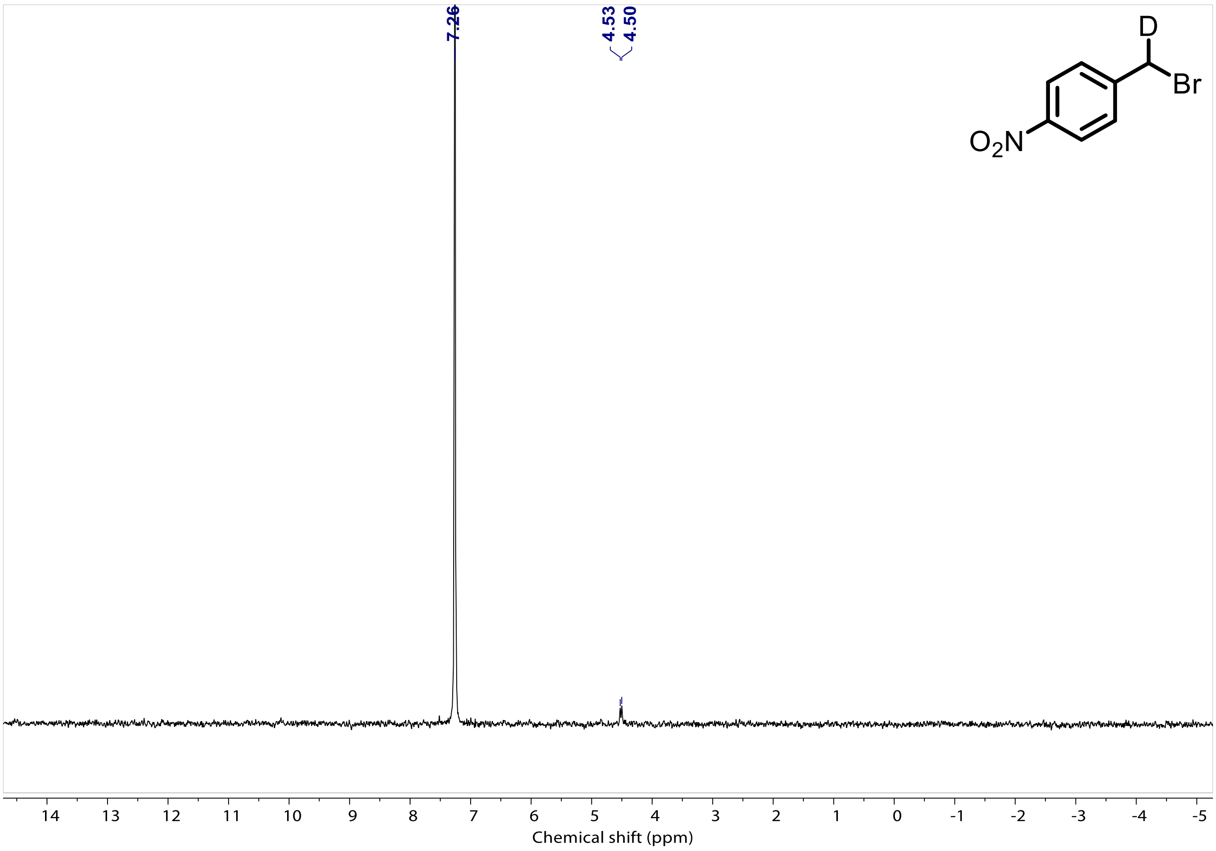

(4-Nitrophenyl)methyl-*d* methanesulfonate

^{1}H NMR (400 MHz, $CDCl_3$)

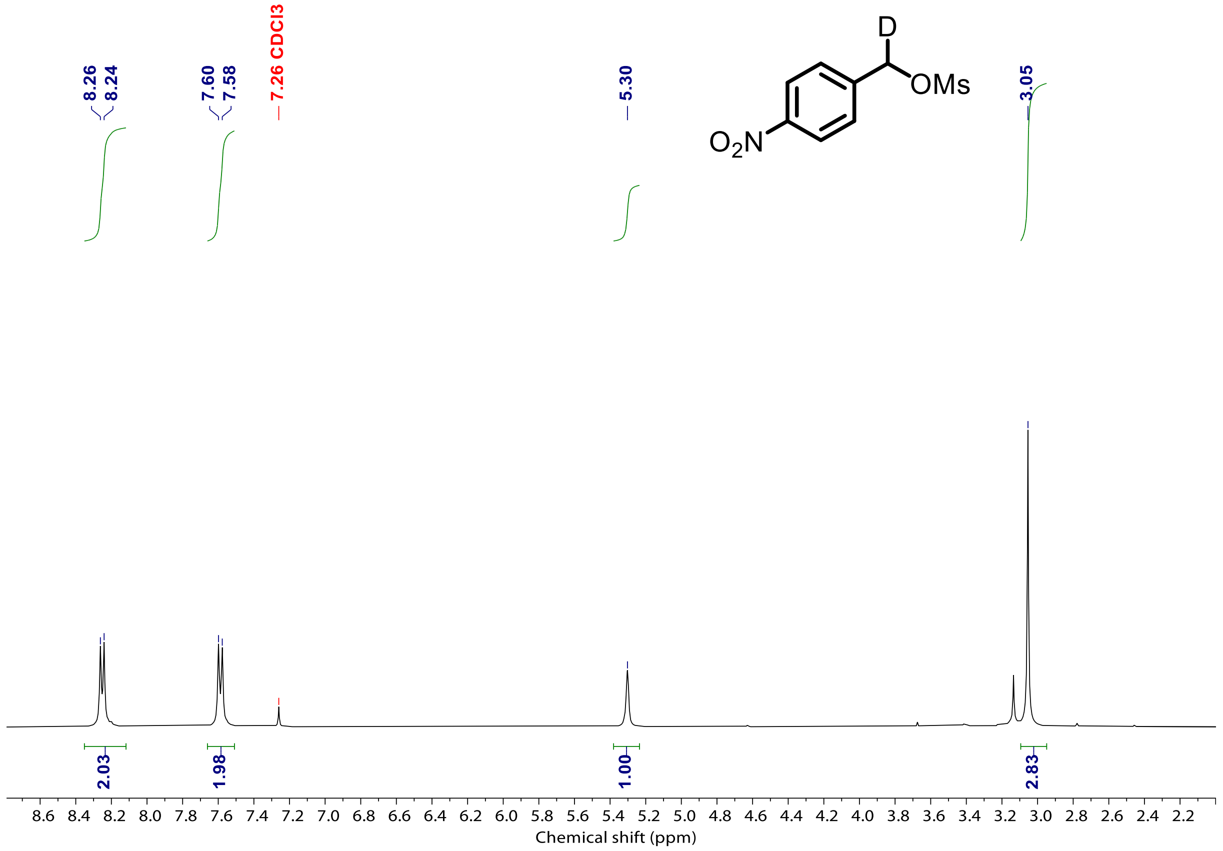

(4-Nitrophenyl)methyl-*d* methanesulfonate

^{13}C NMR (101 MHz, $CHCl_3$)

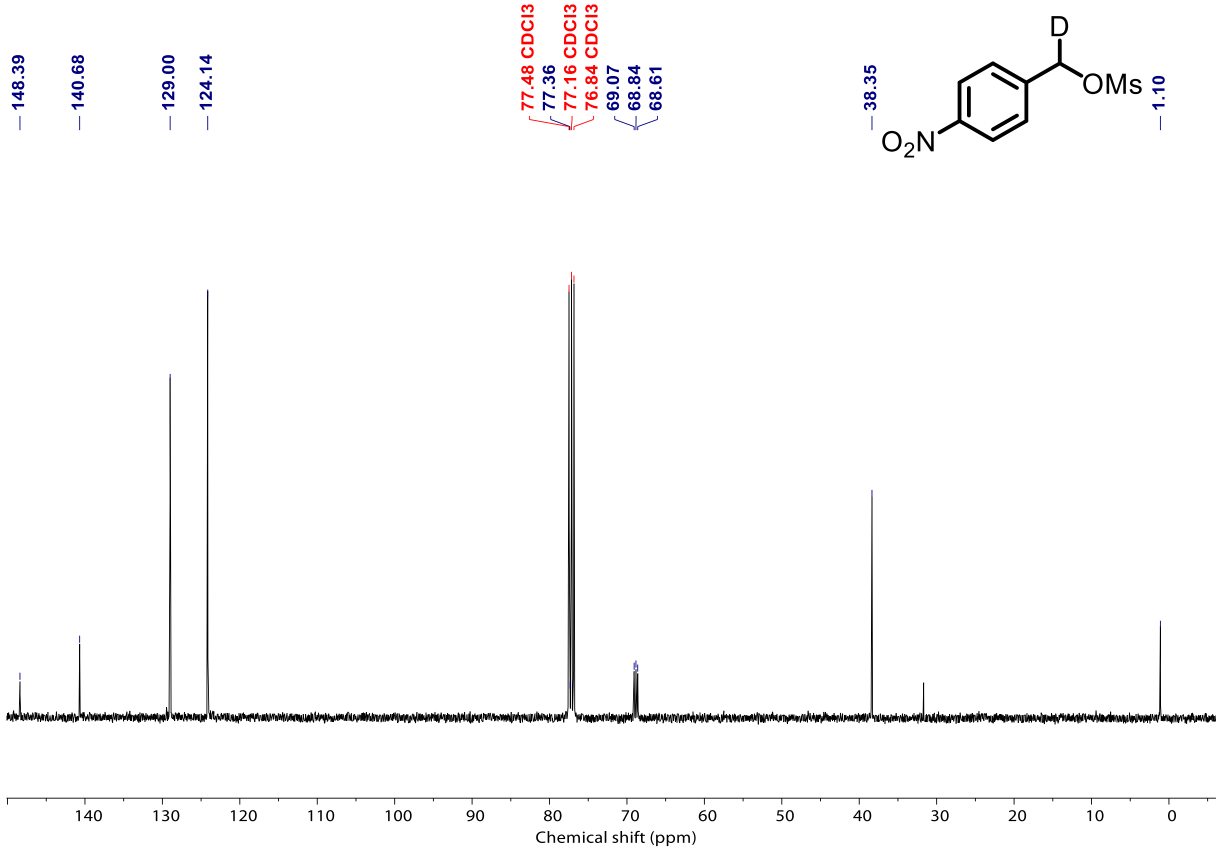

(4-Nitrophenyl)methyl-*d* methanesulfonate

^{2}H NMR (61 MHz, $CHCl_3$)

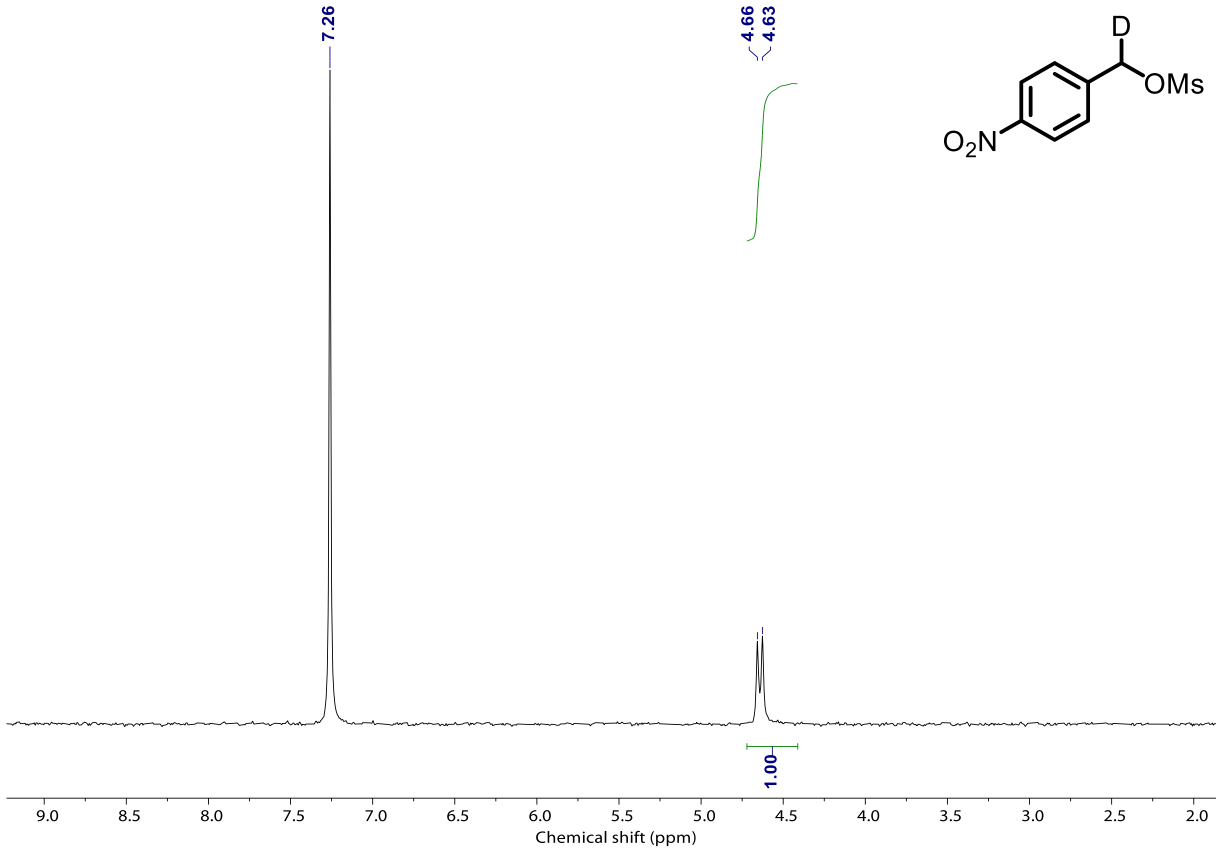

1-(Fluoromethyl-*d*)-4-nitrobenzene

^{1}H NMR (400 MHz, $CDCl_3$)

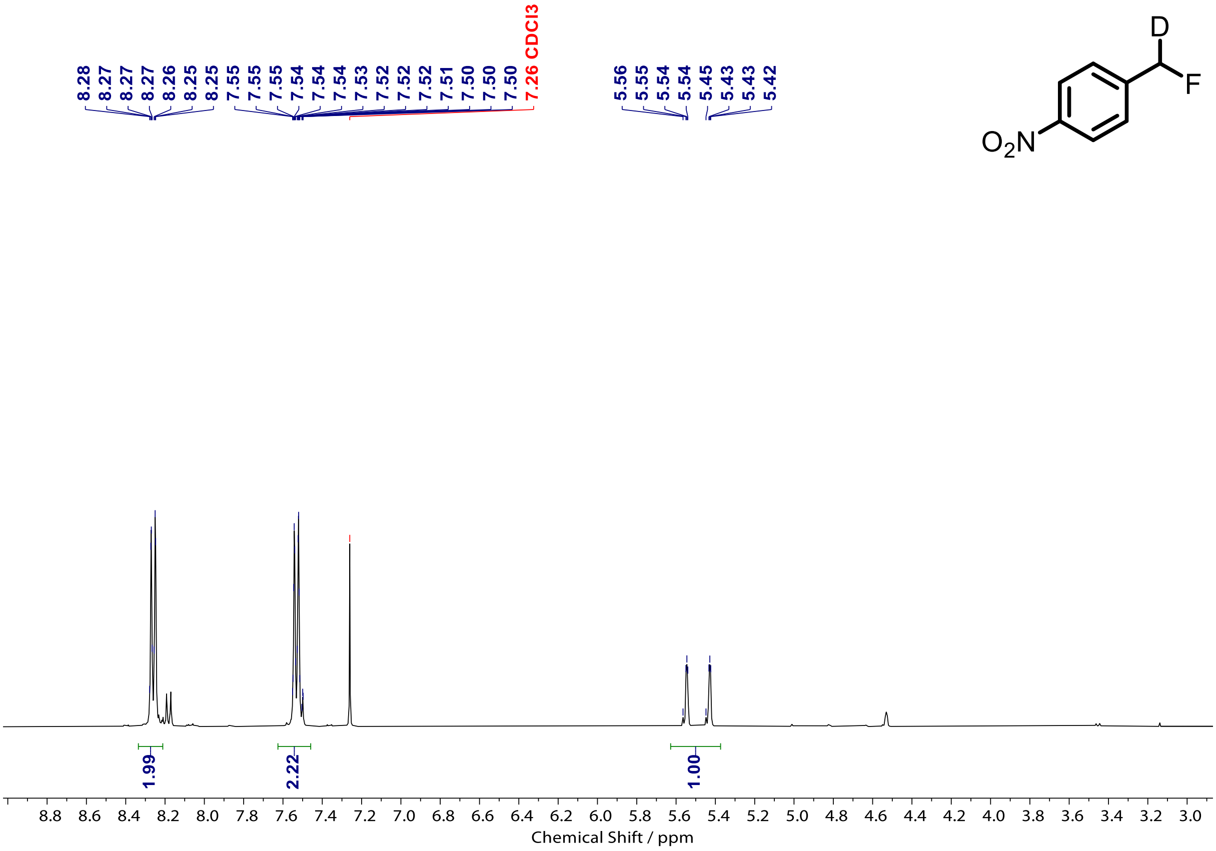

1-(Fluoromethyl-*d*)-4-nitrobenzene

^{13}C NMR (101 MHz, $CHCl_3$)

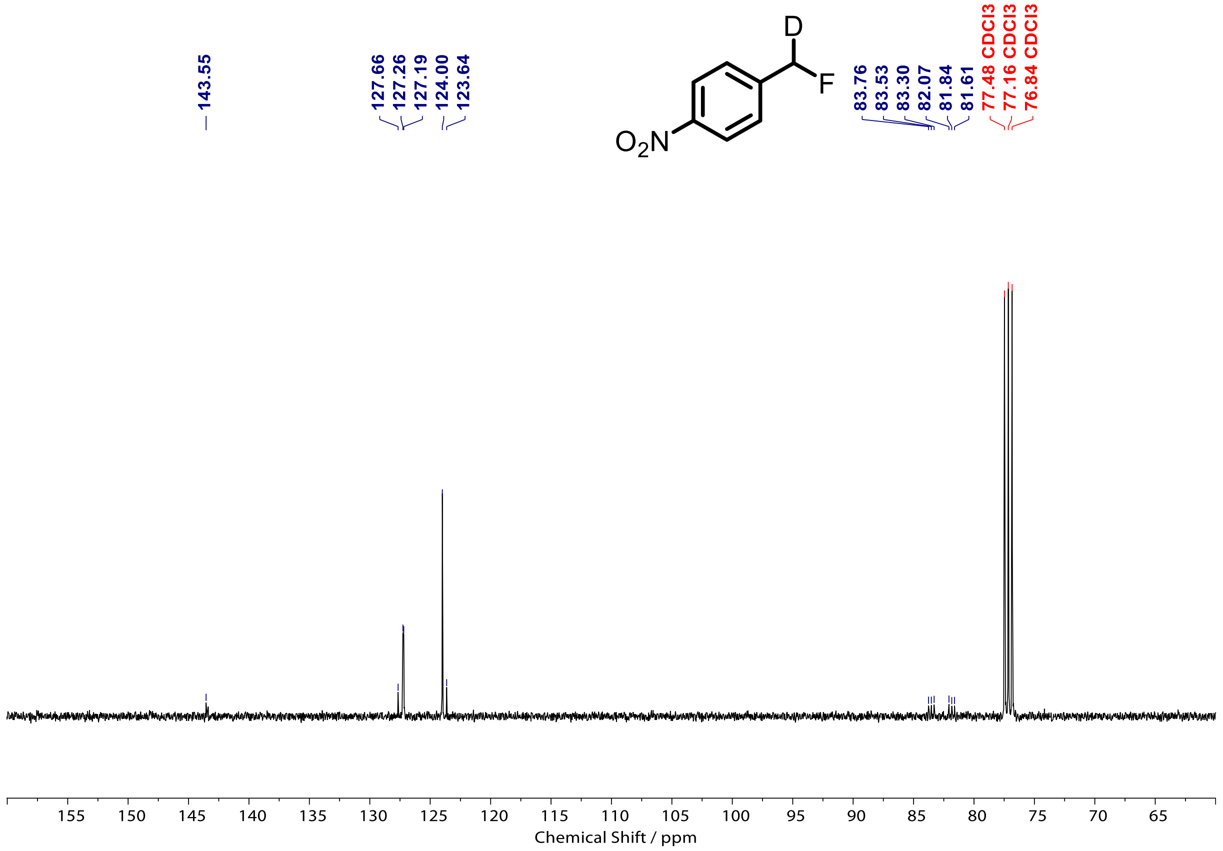

1-(Fluoromethyl-*d*)-4-nitrobenzene

^{2}H NMR (61 MHz, $CHCl_3$)

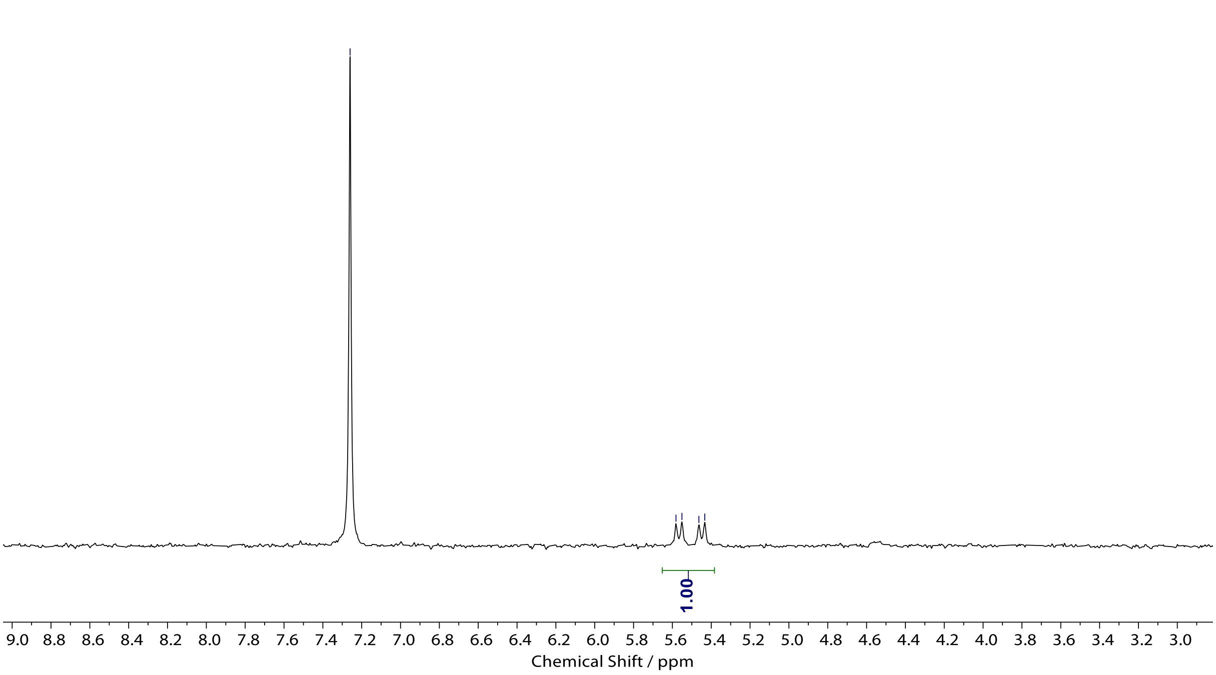

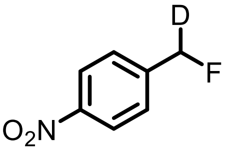

1-(Fluoromethyl-)-4-nitrobenzene

^{19}F NMR (377 MHz, $CDCl_3$)

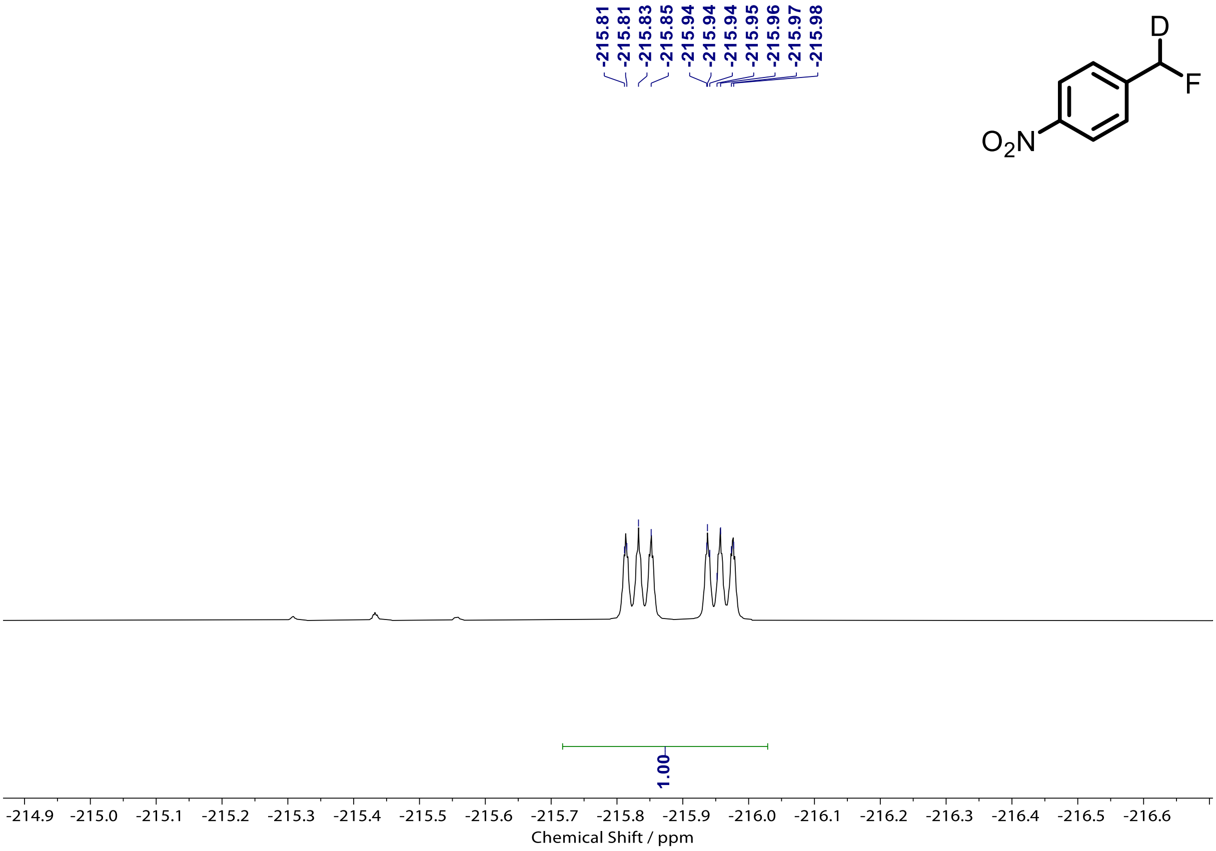

3-(4-Fluorophenyl)propyl 4-nitrobenzenesulfonate

¹H NMR (400 MHz, CD_2Cl_2)

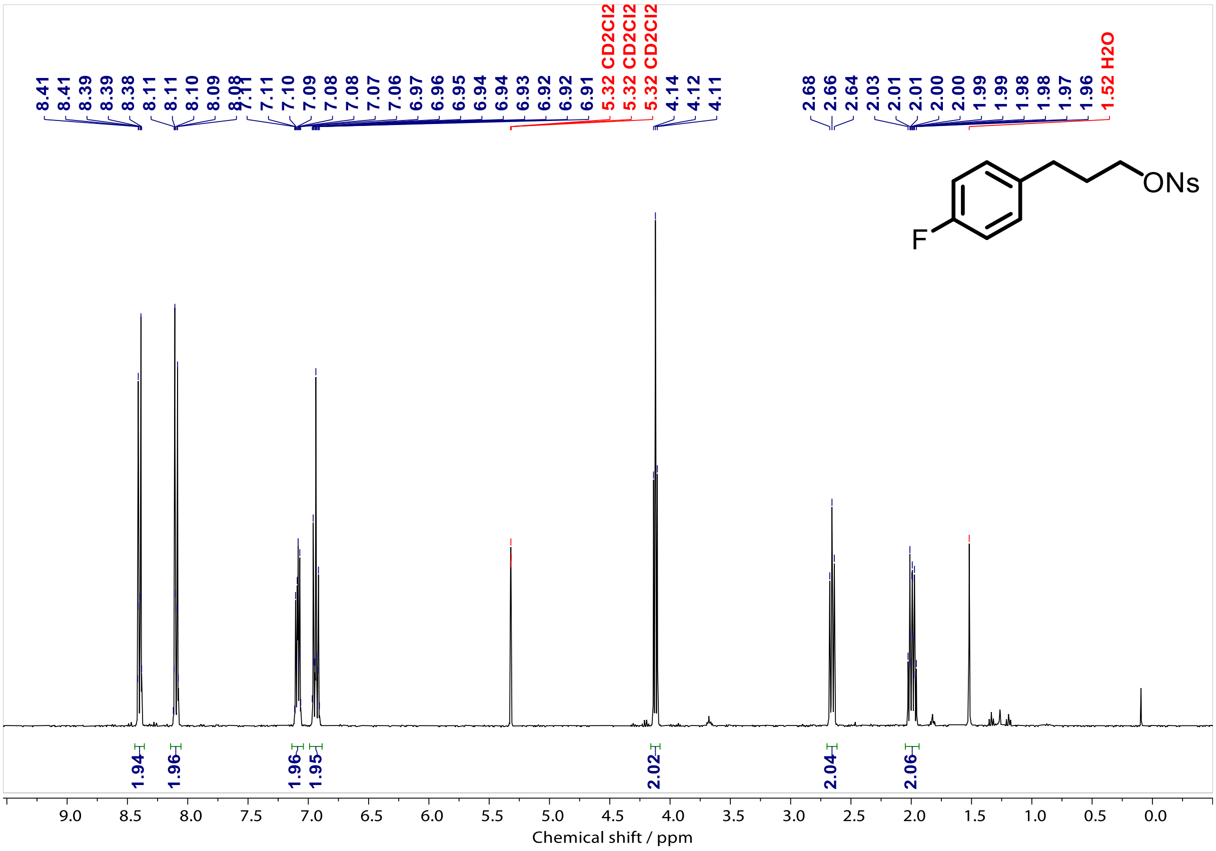

3-(4-Fluorophenyl)propyl 4-nitrobenzenesulfonate

^{13}C NMR (101 MHz, CD_2Cl_2)

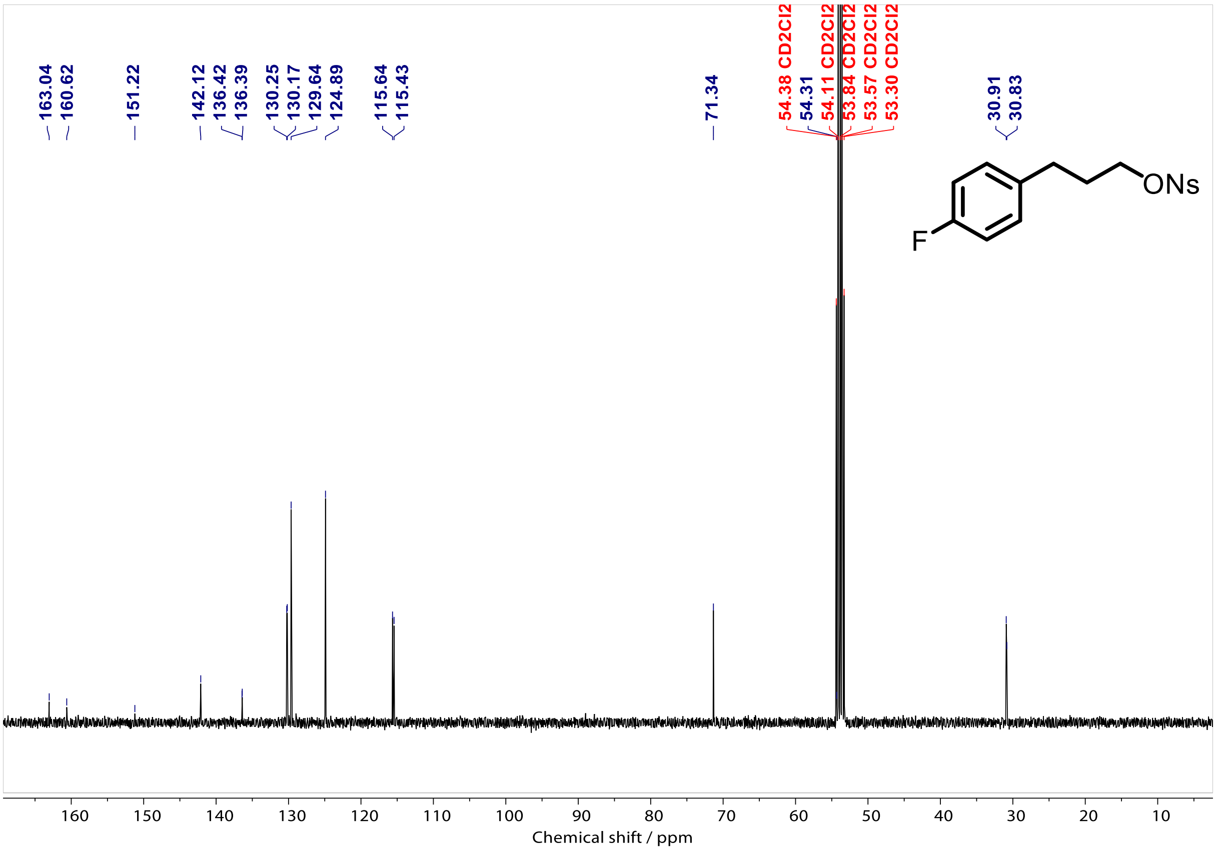

3-(4-Fluorophenyl)propyl 4-nitrobenzenesulfonate

^{19}F NMR (377 MHz, CD_2Cl_2)

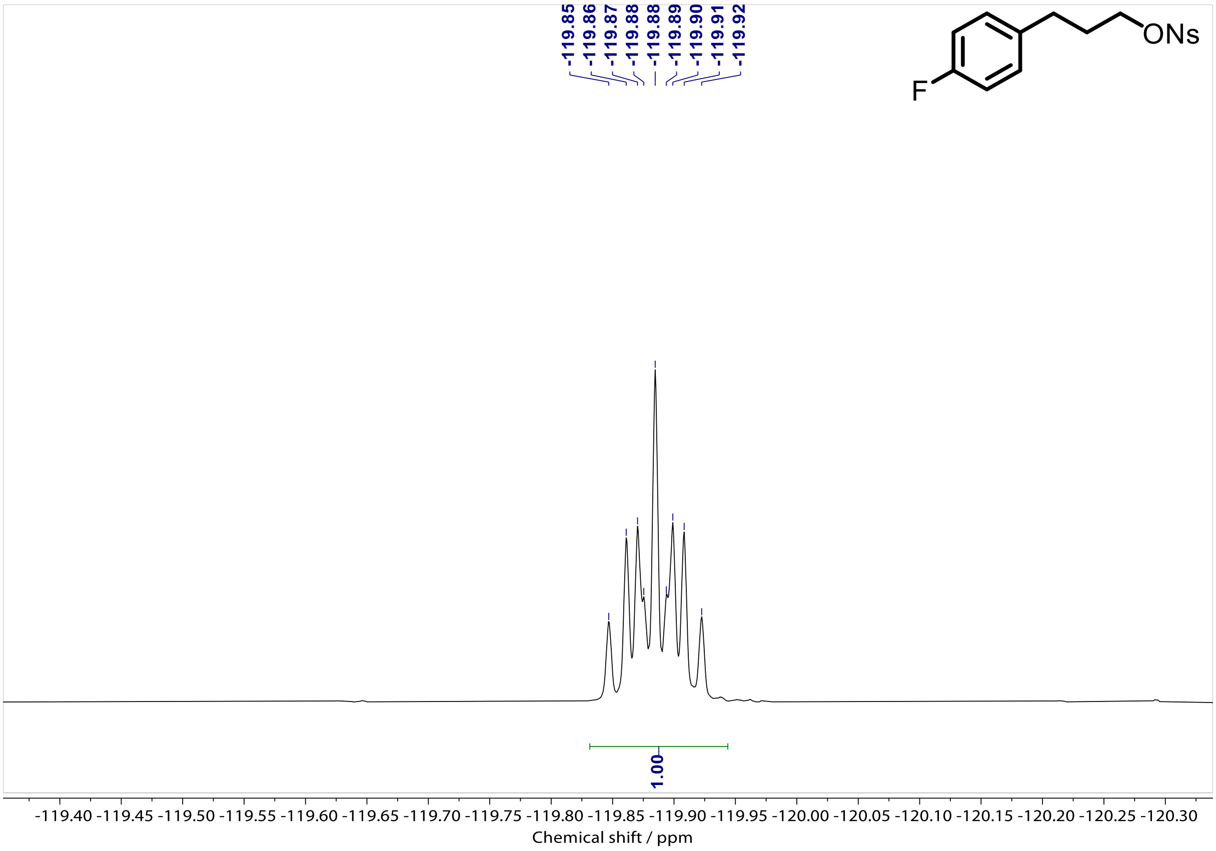

15. References

(1) Stünkel, T.; Siebold, K.; Okumatsu, D.; Murata, K.; Ruyet, L.; Daniliuc, C. G.; Gilmour, R. Para -Selective Dearomatization of Phenols by I(I)/I(III) Catalysis-Based Fluorination. *Chem. Sci.* **2023**, *14* (46), 13574–13580.

(2) Yu, Y.; Häfliger, J.; Wang, Z.; Daniliuc, C. G.; Gilmour, R. Forging Medium Rings via I(I)/I(III)-Catalyzed Diene Carbofunctionalization. *Angew. Chem. Int. Ed.* **2023**, *62* (38).

(3) Yu, Y.; Schäfer, M.; Daniliuc, C. G.; Gilmour, R. Catalytic, Regioselective 1,4-Fluorodifunctionalization of Dienes. *Angew. Chem. Int. Ed.* **2023**, *62* (1).

(4) Ruyet, L.; Roblick, C.; Häfliger, J.; Wang, Z.; Stoffels, T. J.; Daniliuc, C. G.; Gilmour, R. Catalytic Ring Expanding Difluorination: An Enantioselective Platform to Access β,Β-Difluorinated Carbocycles. *Angew. Chem. Int. Ed.* **2024**, *63* (22), e202403957.

(5) Dean, A. C.; Randle, E. H.; Lacey, A. J. D.; Marczak Giorio, G. A.; Doobary, S.; Cons, B. D.; Lennox, A. J. J. Alkene 1,3-Difluorination via Transient Oxonium Intermediates. *Angew. Chem. Int. Ed.* **2024**, *63* (30).

(6) Livingstone, K.; Siebold, K.; Meyer, S.; Martín-Heras, V.; Daniliuc, C. G.; Gilmour, R. Skeletal Ring Contractions via I(I)/I(III) Catalysis: Stereoselective Synthesis of Cis -α,α-Difluorocyclopropanes. *ACS Catal.* **2022**, *12* (23), 14507–14516.

(7) Doobary, S.; Sedikides, A. T.; Caldora, H. P.; Poole, D. L.; Lennox, A. J. J. Electrochemical Vicinal Difluorination of Alkenes: Scalable and Amenable to Electron-Rich Substrates. *Angew. Chem. Int. Ed.* **2020**, *59* (3), 1155–1160.

(8) Neufeld, J.; Daniliuc, C. G.; Gilmour, R. Fluorocyclisation of Oximes to Isoxazolines Through I(I)/I(III) Catalysis. *Helv. Chim. Acta* **2023**, *106* (3).

(9) Sarie, J. C.; Thiehoff, C.; Neufeld, J.; Daniliuc, C. G.; Gilmour, R. Enantioselective Synthesis of 3-Fluorochromanes via Iodine(I)/Iodine(III) Catalysis. *Angew. Chem. Int. Ed.* **2020**, *59* (35), 15069–15075.

(10) Doobary, S.; Lacey, A. J. D.; Sweeting, S. G.; Coppock, S. B.; Caldora, H. P.; Poole, D. L.; Lennox, A. J. J. Diastereodivergent Nucleophile–Nucleophile Alkene Chlorofluorination. *Nat. Chem.* **2024**, *16* (10), 1647–1655.

(11) Li, Z.; Hammond, G. B.; Xu, B. Quantification of Hydrogen Fluoride-Based Reagents Using a Bifunctional NMR Internal Standard. *J. Fluor. Chem.* **2016**, *184*, 72–74.

(12) Doobary, S.; Lacey, A. J. D.; Sweeting, S. G.; Coppock, S. B.; Caldora, H. P.; Poole, D. L.; Lennox, A. J. J. Diastereodivergent Nucleophile–Nucleophile Alkene Chlorofluorination. *Nat. Chem.* **2024**, *16* (10), 1647–1655.

(13) Yokoyama, Y.; Kano, K.; Kondo, Y.; Miyahara, Y.; Miyazaki, K.; Abe, T. Fluoride Ion-Selective Electrode for Organic Solutions. *Anal. Chem.* **2021**, *93* (45), 15058–15062.

(14) Pracht, P.; Grimme, S.; Bannwarth, C.; Bohle, F.; Ehlert, S.; Feldmann, G.; Gorges, J.; Müller, M.; Neudecker, T.; Plett, C.; Spicher, S.; Steinbach, P.; Wesołowski, P. A.; Zeller, F. CREST—A Program for the Exploration of Low-Energy Molecular Chemical Space. *J. Chem. Phys.* **2024**, *160* (11), 114110.

(15) Bannwarth, C.; Ehlert, S.; Grimme, S. GFN2-XTB - An Accurate and Broadly Parametrized Self-Consistent Tight-Binding Quantum Chemical Method with Multipole Electrostatics and Density-Dependent Dispersion Contributions. *J. Chem. Theory Comput.* **2019**, *15* (3), 1652–1671.

(16) Neese, F. Software Update: The ORCA Program System—Version 5.0. *Wiley Interdisciplinary Reviews: Computational Molecular Science* **2022**, *12* (5), e1606.

(17) Neese, F.; Schwabe, T.; Grimme, S. Analytic Derivatives for Perturbatively Corrected "Double Hybrid" Density Functionals: Theory, Implementation, and Applications. *J. Chem. Phys.* **2007**, *126* (12), 124115.

(18) Caldeweyher, E.; Ehlert, S.; Hansen, A.; Neugebauer, H.; Spicher, S.; Bannwarth, C.; Grimme, S. A Generally Applicable Atomic-Charge Dependent London Dispersion Correction. *J. Chem. Phys.* **2019**, *150* (15), 154122.

(19) Caldeweyher, E.; Mewes, J. M.; Ehlert, S.; Grimme, S. Extension and Evaluation of the D4 London-Dispersion Model for Periodic Systems. *Phys. Chem. Chem. Phys.* **2020**, *22* (16), 8499–8512.

(20) Becke, A. D. Density-Functional Thermochemistry. III. The Role of Exact Exchange. *The J. Chem. Phys.* **1993**, *98* (7), 5648–5652.

(21) Lee, C.; Yang, W.; Parr, R. G. Development of the Colle-Salvetti Correlation-Energy Formula into a Functional of the Electron Density. *Phys. Rev. B* **1988**, *37* (2), 785–789.

(22) Becke, A. D. Density-Functional Exchange-Energy Approximation with Correct Asymptotic Behavior. *Phys. Rev. A* **1988**, *38* (6), 3098–3100.

(23) Perdew, J. P. Density-Functional Approximation for the Correlation Energy of the Inhomogeneous Electron Gas. *Phys. Rev. B* **1986**, *33* (12), 8822–8824.

(24) Yanai, T.; Tew, D. P.; Handy, N. C. A New Hybrid Exchange–Correlation Functional Using the Coulomb-Attenuating Method (CAM-B3LYP). *Chem. Phys. Lett.* **2004**, *393* (1–3), 51–57.

(25) Lange, A. W.; Rohrdanz, M. A.; Herbert, J. M. Charge-Transfer Excited States in a π-Stacked Adenine Dimer, as Predicted Using Long-Range-Corrected Time-Dependent Density Functional Theory. *J. Phys. Chem. B* **2008**, *112* (20), 6304–6308.

(26) Iikura, H.; Tsuneda, T.; Yanai, T.; Hirao, K. A Long-Range Correction Scheme for Generalized-Gradient-Approximation Exchange Functionals. *J. Chem. Phys.* **2001**, *115* (8), 3540–3544.

(27) Perdew, J. P.; Burke, K.; Ernzerhof, M. Generalized Gradient Approximation Made Simple. *Phys. Rev. Lett.* **1996**, *77* (18), 3865–3868.

(28) Perdew, J. P.; Burke, K.; Ernzerhof, M. Erratum: Generalized Gradient Approximation Made Simple (Physical Review Letters (1996) 77 (3865)). *Phys. Rev. Lett.* **1997**, 1396.

(29) Zhao, Y.; Truhlar, D. G. The M06 Suite of Density Functionals for Main Group Thermochemistry, Thermochemical Kinetics, Noncovalent Interactions, Excited States, and Transition Elements: Two New Functionals and Systematic Testing of Four M06-

Class Functionals and 12 Other Functionals. *Theor. Chem. Acc.* **2008**, *120* (1–3), 215–241.

(30) Santra, G.; Sylvetsky, N.; Martin, J. M. L. Minimally Empirical Double-Hybrid Functionals Trained against the GMTKN55 Database: RevDSD-PBEP86-D4, RevDOD-PBE-D4, and DOD-SCAN-D4. *J. Phys. Chem. A* **2019**, *123* (24), 5129–5143.

(31) Ernzerhof, M.; Perdew, J. P. Generalized Gradient Approximation to the Angle- and System-Averaged Exchange Hole. *J. Chem. Phys.* **1998**, *109* (9), 3313–3320.

(32) Adamo, C.; Barone, V. Toward Reliable Density Functional Methods without Adjustable Parameters: The PBE0 Model. *J. Chem. Phys.* **1999**, *110* (13).

(33) Goerigk, L.; Grimme, S. A Thorough Benchmark of Density Functional Methods for General Main Group Thermochemistry, Kinetics, and Noncovalent Interactions. *Phys. Chem. Chem. Phys.* **2011**, *13* (14), 6670.

(34) Zhang, Y.; Yang, W. Comment on "Generalized Gradient Approximation Made Simple." *Phys. Rev. Lett.* **1998**, *80* (4), 890.

(35) Grimme, S.; Antony, J.; Ehrlich, S.; Krieg, H. A Consistent and Accurate Ab Initio Parametrization of Density Functional Dispersion Correction (DFT-D) for the 94 Elements H-Pu. *J. Chem. Phys.* **2010**, *132* (15), 154104.

(36) Ernzerhof, M.; Scuseria, G. E. Assessment of the Perdew–Burke–Ernzerhof Exchange-Correlation Functional. *J. Chem. Phys.* **1999**, *110* (11), 5029–5036.

(37) Chai, J. Da; Head-Gordon, M. Systematic Optimization of Long-Range Corrected Hybrid Density Functionals. *J. Chem. Phys.* **2008**, *128* (8), 84106.

(38) Weigend, F.; Ahlrichs, R. Balanced Basis Sets of Split Valence, Triple Zeta Valence and Quadruple Zeta Valence Quality for H to Rn: Design and Assessment of Accuracy. *Phys. Chem. Chem. Phys.* **2005**, *7* (18), 3297–3305.

(39) Weigend, F. Accurate Coulomb-Fitting Basis Sets for H to Rn. *Phys. Chem. Chem. Phys.* **2006**, *8* (9), 1057–1065.

(40) Garcia-Ratés, M.; Neese, F. Effect of the Solute Cavity on the Solvation Energy and Its Derivatives within the Framework of the Gaussian Charge Scheme. *J. Comput. Chem.* **2020**, *41* (9), 922–939.

(41) Jouyban, A.; Soltanpour, S.; Chan, H.-K. A Simple Relationship between Dielectric Constant of Mixed Solvents with Solvent Composition and Temperature. *Int. J. Pharm.* **2004**, *269* (2), 353–360.

(42) Tupikina, E. Yu.; Tokhadze, K. G.; Karpov, V. V.; Denisov, G. S.; Tolstoy, P. M. Stretching Force Constants as Descriptors of Energy and Geometry of F···HF Hydrogen Bonds. *Spectrochim. Acta A Mol. Biomol. Spectrosc.* **2020**, *241*, 118677.

(43) van der Lubbe, S. C. C.; Fonseca Guerra, C. The Nature of Hydrogen Bonds: A Delineation of the Role of Different Energy Components on Hydrogen Bond Strengths and Lengths. *Chem. Asian J.* **2019**, *14* (16), 2760–2769.

(44) Wager, J. F. New Perspective on Hydrogen Bonding. *ACS Omega* **2023**, *8* (44).

(45) Lu, T.; Chen, F. Multiwfn: A Multifunctional Wavefunction Analyzer. *J. Comput. Chem.* **2012**, *33* (5), 580–592.

(46) Humphrey, W.; Dalke, A.; Schulten, K. VMD: Visual Molecular Dynamics. *J. Mol. Graph.* **1996**, *14* (1), 33–38.

(47) Emamian, S.; Lu, T.; Kruse, H.; Emamian, H. Exploring Nature and Predicting Strength of Hydrogen Bonds: A Correlation Analysis Between Atoms-in-Molecules Descriptors, Binding Energies, and Energy Components of Symmetry-Adapted Perturbation Theory. *J. Comput. Chem.* **2019**, *40* (32), 2868–2881.

(48) Domingo, L. R.; Ríos-Gutiérrez, M.; Pérez, P. *Applications of the Conceptual Density Functional Theory Indices to Organic Chemistry Reactivity*; 2016; Vol. 21, p 748.

(49) Koopmans, T. Über Die Zuordnung von Wellenfunktionen Und Eigenwerten Zu Den Einzelnen Elektronen Eines Atoms. *Physica* **1934**, *1* (1–6), 104–113.

(50) Politzer, P.; Murray, J. S.; Bulat, F. A. Average Local Ionization Energy: A Review. *J. Mol. Model* **2010**, *16* (11), 1731–1742.

(51) Ehresmann, B.; Martin, B.; Horn, A. H. C.; Clark, T. Local Molecular Properties and Their Use in Predicting Reactivity. *J. Mol. Model* **2003**, *9* (5), 342–347.

(52) Sjoberg, P.; Murray, J. S.; Brinck, T.; Politzer, P. Average Local Ionization Energies on the Molecular Surfaces of Aromatic Systems as Guides to Chemical Reactivity. *Can. J. Chem.* **1990**, *68* (8), 1440–1443.

(53) Longuet, M.; Vitse, K.; Martin-Mingot, A.; Michelet, B.; Guégan, F.; Thibaudeau, S. Determination of the Hammett Acidity of HF/Base Reagents. *J. Am. Chem. Soc.* **2024**, *146* (17), 12167–12173.

(54) Hoops, S.; Sahle, S.; Gauges, R.; Lee, C.; Pahle, J.; Simus, N.; Singhal, M.; Xu, L.; Mendes, P.; Kummer, U. COPASI—a COmplex PAthway SImulator. *Bioinformatics* **2006**, *22* (24), 3067–3074.

(55) Hoops, S.; Sahle, S.; Gauges, R.; Lee, C.; Pahle, J.; Simus, N.; Singhal, M.; Xu, L.; Mendes, P.; Kummer, U. COPASI—a COmplex PAthway SImulator. *Bioinformatics* **2006**, *22* (24), 3067–3074.

(56) Barnett, D. W.; Refaei, M. S.; Curley, R. W. Chirally Deuterated Benzyl Chlorides from Benzyl Alcohols via Hexachloroacetone/Polymer-supported Triphenylphosphine: Synthesis of Protected (2 S , 3 S)-[3- 2 H, 15 N]-tyrosine. *J. Label. Compd. Radiopharm.* **2013**, *56* (1), 6–11.

(57) Milic, M.; Targos, K.; Tellez Chavez, M.; Thompson, M. A. M.; Jennings, J. J.; Franz, A. K. NMR Quantification of Hydrogen-Bond-Accepting Ability for Organic Molecules. *J. Org. Chem.* **2021**, *86* (9), 6031–6043.

(58) Iashin, V.; Wirtanen, T.; Perea-Buceta, J. E. Tetramethylammonium Fluoride: Fundamental Properties and Applications in C-F Bond-Forming Reactions and as a Base. *Catalysts* **2022**, *12* (2), 233–291.
